# Associations between dimensions of the social environment and cardiometabolic risk factors: Systematic review and meta-analysis

**DOI:** 10.1016/j.ssmph.2023.101559

**Published:** 2023-11-25

**Authors:** Taymara C. Abreu, Joreintje D. Mackenbach, Fleur Heuvelman, Linda J. Schoonmade, Joline W.J. Beulens

**Affiliations:** a Department of Epidemiology & Data Science, Amsterdam UMC - location VUmc, Amsterdam, Noord-Holland, the Netherlands; b Upstream Team, the Netherlands; c University Library, Vrije Universiteit Amsterdam, Amsterdam, Noord-Holland, the Netherlands; d Julius Center for Health Sciences and Primary Care, University Medical Center Utrecht, Utrecht, Utrecht, the Netherlands; e Amsterdam Public Health, Amsterdam Cardiovascular Sciences, Amsterdam, Noord-Holland, the Netherlands

**Keywords:** Social epidemiology, Social context, Social networking, Socioeconomic factors, Social determinants of health, Cardiovascular diseases, Metabolic diseases, Deprivation, Exposome

## Abstract

**Aim:**

The social environment (SE), including social contacts, norms and support, is an understudied element of the living environment which impacts health. We aim to comprehensively summarize the evidence on the association between the SE and risk factors of cardiometabolic disease (CMD).

**Methods:**

We performed a systematic review and meta-analysis based on studies published in PubMed, Scopus and Web of Science Core Collection from inception to 16 February 2021. Studies that used a risk factor of CMD, e.g., HbA1c or blood pressure, as outcome and social environmental factors such as area-level deprivation or social network size as independent variables were included. Titles and abstracts were screened in duplicate. Study quality was assessed using the Newcastle-Ottawa Scale. Data appraisal and extraction were based on the study protocol published in PROSPERO. Data were synthesized through vote counting and meta-analyses.

**Results:**

From the 7521 records screened, 168 studies reported 1050 associations were included in this review. Four meta-analyses based on 24 associations suggested that an unfavorable social environment was associated with increased risk of cardiometabolic risk factors, with three of them being statistically significant. For example, individuals that experienced more economic and social disadvantage had a higher “CVD risk scores” (OR = 1.54, 95%CI: 1.35 to 1.84). Of the 458 associations included in the vote counting, 323 (71%) pointed towards unfavorable social environments being associated with higher CMD risk.

**Conclusion:**

Higher economic and social disadvantage seem to contribute to unfavorable CMD risk factor profiles, while evidence for other dimensions of the social environment is limited.

## Highlights

• Comprehensive systematic review and meta-analysis on the link between social environment and cardiometabolic risk factors.
• Data synthesis from 168 studies reporting 1,050 associations.
• Vote counting: 71% of associations pointed towards unfavourable social environments linked to higher CMD risk.
• Meta-analysis: more economic and social disadvantage associated with higher “CVD risk scores”.
• Substantial heterogeneity among the studies.

## Introduction

Cardiometabolic diseases (CMDs), including cardiovascular diseases (CVDs) and type 2 diabetes mellitus (T2DM) are the number one cause of death worldwide (World Health Organization (WHO)). Although some CMDs are highly heritable, e.g., familial hypercholesterolemia (Ison et al.), the non-genetic nature of CMDs is reflected by the close association between lifestyle behaviors and CMD risk. In fact, lifestyle risk factors such as an unhealthy diet or physical inactivity account for more than 70% of total cardiovascular events, 80% of coronary heart disease events and 90% of incidence of T2DM (Mozaffarian et al., 2007).

Alterations of lifestyle risk factors can therefore have a major impact on the prevention of CMDs. Several landmark trials indeed showed that adhering to a Mediterranean diet (Grosso et al., 2017) or a combined lifestyle intervention (Diabetes Prevention Program Research Group, 2002; Maruthur et al., 2009) reduces the risk of CMDs substantially with up to 58% risk reduction of type 2 diabetes. These lifestyle risk factors not only influence established risk markers such as elevated blood pressure, blood lipids, and glucose-insulin homeostasis but also other pathways such as endothelial function, oxidative stress, inflammation (e.g., C-reactive protein), thrombosis/coagulation, arrhythmia, and other intermediary pathways (e.g., psychosocial stress) (Appel et al., 2005; Folsom, 2013; Man & Xia, 2020; Netz et al., 2005; Upadhyay, 2015; Vandercappellen et al., 2022; Wang et al., 2017). Indeed, clinical trials have demonstrated the effects of lifestyle interventions on CMD risk factors (Hochsmann et al., 2021; Wister et al., 2007). On the basis of population-wide benefits and minimizing adverse drug effects, changes in lifestyle are crucially important for primary prevention and may also have beneficial effects for secondary prevention. However, many individuals cannot maintain healthy lifestyle behaviors in the long term (Burgess et al., 2017; Middleton et al., 2013).

That is because many lifestyle programs and interventions do not address the upstream risk factors of unhealthy lifestyle (Lakerveld & Mackenbach, 2017), i.e., the causes-of-the-causes of CMD. One important upstream factor that drives lifestyle behaviors is the social environment, i.e., the social relationships and social context in which groups of people live and interact. The social environment encompasses several concepts organized in dimensions; however, there is no consensus in the literature on which dimensions are included in the social environment. In this work, we adopt the dimensions proposed by Kepper et al., 2019, namely: Economic and Social Disadvantage, Discrimination and Segregation, Crime and Safety, Social Cohesion and Social Capital, Disorders and Incivilities, Social Relationships and Norms, and Civic Participation and Engagement. These concepts and dimensions were found to be related to CVD risk, for example, the extent of connectedness and solidarity in a community – often labeled “social cohesion” – has been associated with a reduced likelihood of CVDs and related risk factors, such as myocardial infarction, stroke and hypertension (Kim et al., 2013, 2014; Lagisetty et al., 2016). Social cohesion may protect against CMD through multiple pathways, including better coping abilities, healthier lifestyle behaviors and positive psychological effects. Similarly, a meta-analysis showed that poor social relationships are associated with 29% increased risk of coronary heart disease and 32% increased risk of stroke (Valtorta et al., 2016). Another important factor which can have a detrimental impact on an individual’s lifestyle, and in turn on CVD health, is social isolation - the absence of social connections and interactions (Leigh-Hunt et al., 2017). Social isolation is related to feelings of loneliness, which seems to result in a chronic social stress response (Xia & Li, 2018). These feelings of stress are able to activate different mechanisms in the body which may negatively impact CVD risk. In addition, the results from a global study suggest that civic participation, such as voting, being a volunteer and participating in recreational and sporting activities “strengthens existing social networks, increases social cohesion, creates a common sense of goals and purpose, and improves overall health and wellbeing” (Kim et al., 2015). And a US study that aimed to investigate how the benefits of volunteering get “under the skin” found that middle-aged and older volunteers were less likely to have central adiposity, lipid dysregulation and hypertension than their non-volunteering peers (Burr et al., 2016). In summary, it seems that a variety of aspects related to the social environment may influence CVD risk in different ways. There is even tentative evidence that social network interventions may reduce HbA1c levels in T2DM patients (Spencer-Bonilla, Ponce, Rodriguez-Gutierrez, et al., 2017). Yet, to understand the causes-of-the-causes of CMDs, and to develop effective intervention and prevention strategies, it is important to evaluate the existing evidence on the associations between dimensions of the social environment and risk factors of CMD. Therefore, it was our aim to systematically summarize and meta-analyze the available evidence.

## Methods

This is a systematic literature review of English-language scientific articles on the association between different dimensions of the social environment and cardiometabolic risk factors in adults. This systematic literature review is the result of a review protocol that was prospectively registered with the International Prospective Register of Systematic Reviews (PROSPERO) database (registration number CRD42021223035). Because the search resulted in exceptionally large numbers of relevant articles, the reporting is split according to outcome measures: this review focuses on cardiometabolic risk factors and a twin review focuses on cardiometabolic disease endpoints (Abreu et al. submitted elsewhere). This review is written according to the latest Preferred Reporting Items for Systematic Reviews and Meta-Analysis PRISMA guidelines for systematic reviews (Page et al., 2021a, 2021b).

### Search strategy and study selection

To identify all relevant publications, we conducted systematic searches in the bibliographic databases PubMed, Scopus and Web of Science Core Collection from inception to 16 February 2021, in collaboration with a medical information specialist (LS). We used free-text terms in all databases. For PubMed, the search terms also included indexed terms from MeSH. The search comprised a block for “cardiometabolic diseases” (e.g., heart disease, hyperlipidemia, HbA1c), for “dimensions of the social environment” (e.g., social capital, area-level deprivation), and for the “contextual level” of the social environment (e.g., community, network). The latter was added to exclude studies focusing on individual-level social factors (e.g., individual SEP). Supplementary File 1 contains the full search strategy for all electronic databases including number of results. Articles in all languages were accepted during the search. Reference lists of the articles included were manually searched for other relevant publications.

The search was performed and duplicates were removed by a medical information specialist (LS). All de-duplicated titles and abstracts retrieved from the search were screened for eligibility by at least two out of three authors independently (TCA, JDM, JWJB), according to the criteria for inclusion and exclusion, using the semi-automation tool Covidence. A pilot test in a random sample of 100 results ensured consistency among screeners. Afterwards, TCA and JDM independently assessed all full texts for inclusion. Differences in judgement were resolved through a consensus procedure. Studies were included if they met the inclusion criteria as stated below.

Original studies that examined associations between dimensions of the social environment and risk factors of CMD in adults were reviewed. The scope of this review was limited to exposures that assessed properties of the social context, i.e., social factors assessed at the environmental level (e.g., area-level income), but not those that assessed properties of individuals, i.e., individual reported happiness derived from their social network. We did however include social factors that were assessed at the individual-level but reflected a property of the social context in which an individual is inserted (e.g., one’s social network size).

Studies were included for this review on risk factors of CMD or the twin review on cardiometabolic outcomes if they: (i) studied an adult population or followed children and adolescents beyond 18 years of age; (ii) studied risk factors of CMD, or incidence or prevalence of CMD outcomes; (iii) covered any measure of the social environmental that potentially influences CMD; (iv) were observational or intervention studies; and (v) were written in English. We excluded studies if they: (i) were limited to children and adolescents; (ii) studied obesity as outcome - given the recent evidence available for this outcome (Daniels et al., 2021; Glonti et al., 2016; Powell et al., 2015); (iii) studied risk factors of CMD with little or no influence of lifestyle behaviors (e.g., congenital heart disease, rheumatic heart disease, and type 1 diabetes) as outcomes; (iv) focused on treatment, medication, or management of disease outcomes; (v) were conducted in samples of patient populations or pregnant women; (vi) were health economic evaluations, simulation studies, or publications that did not report original scientific research; or (viii) studied mortality outcomes alone, or did not differentiate between morbidity and mortality outcomes.

### Data extraction

Three authors (TCA, JDM and FH) performed data extraction from eligible studies according to a standardized protocol and a predefined list of variables including study and sample characteristics. Social environment factors were categorized into one of eight dimensions according to a conceptual framework (Supplementary File 2): Social Cohesion and Social Capital; Sense of Place/Belonging; Crime and Safety; Disorder and Incivilities; Discrimination and Segregation; Economic and Social Disadvantage; Social Relationships and Norms; and Civic Participation/Engagement. Outcomes were categorized into one of four categories namely “glucose metabolism-related risk factors” (e.g., HOMA, HbA1c), “metabolic and inflammatory-related risk factors” (e.g., lipid levels, CRP, cortisol), “cardiovascular health-related risk factors” (e.g., atherosclerosis, systolic blood pressure, intima-media thickness) and “CVD risk scores” (e.g., Framingham Risk Score, metabolic syndrome, allostatic load). In case of missing data on effect measures, study investigators were contacted. When available, data on sex-specific effect metrics were extracted. If relevant papers contained both separate (e.g., HbA1c, lipid levels, systolic blood pressure, etc.) and CVD risk scores (e.g., “overall risk score”) in their results section, only the effect measures on combined outcomes were reported. Generally, we report on effect sizes from fully adjusted statistical models except when the fully adjusted model was corrected for lifestyle behaviors (e.g., diet, physical activity, alcohol or smoking). In this case, the associations from the model without lifestyle factors were reported because we hypothesized lifestyle behaviors to be intermediary rather than confounding variables.

### Quality assessment

Three authors (TCA, JDM and FH) assessed the quality of all studies included. Disagreements were resolved by consensus. The Newcastle-Ottawa Scale (NOS) was used to assess cohort studies (Wells et al.) (Supplementary File 3). An adapted version of the NOS was used for the quality assessment of cross-sectional studies (Herzog et al., 2013) (Supplementary File 4). Cohort studies were able to gain a total of 9 points based on 8 items, whereas cross-sectional studies could gain a total of 10 points based on 7 items. The assessment was divided into three domains namely selection, comparability and outcome. In order to get comparable quality ratings of all studies, we calculated the percentage of the maximum number of points a paper could gain. Ratings reflect the methodological quality of the associations between social environmental factors and risk factors of CMD, even if this was not the primary research question of the study. Low quality studies were defined as those who received less than 50% of all possible points. Prior to the quality assessment of included studies, the process of quality assessment was piloted in a random subsample of 10 studies.

### Data synthesis

Data synthesis was conducted for combined exposure categories (eight social environment dimensions) and combined outcome categories (four risk factor of CMD categories). In accordance with the Cochrane Handbook for Systematic Reviews of Interventions (Higgins et al., 2022), extracted data were synthesized where possible with two approaches namely meta-analysis and vote counting for studies that had a medium or high quality score. For both methods, associations from low quality studies and associations including an exposure measure from the dimension “Discrimination and Segregation” (e.g., proportion of migrants in the neighborhood) were excluded. “Discrimination and Segregation” was excluded because in general exposure measures used by authors were poorly defined, highly heterogeneous in operationalization, and therefore not comparable across different studies.

Criteria for associations to be included in meta-analyses were: i) three or more associations available per combination of social environment dimension and CMD risk factor category; ii) effect estimates reported as ratios (e.g., odds ratios, relative risk, and hazard ratios); iii) having variance measures; and iv) exposures being operationalized as dichotomous or categorical variables. In case of categorical exposures, we compared the two extreme categories (e.g., highest vs. lowest deprivation). We did not meta-analyze associations on continuous outcomes or continuous or incremental exposures due to methodological challenges in converting continuous data into categorical data, standardizing scales/scores, and in pooling together different effect estimates. We performed random-effects meta-analysis, which accounted for the multilevel structure of the data as many studies reported on more than one association of interest. All models where based on a t-distribution as recommended by the guidelines of R package used (Viechtbauer, 2010). The reference category across all exposures was harmonized and defined as the group with the most favorable social environment. Subgroup analyzes were performed for sex-specific effect measures when sufficient data was available. We aimed to perform sensitivity analyzes according to the studies’ country income level (World Bank, 2021) but were unable to given the absence of sufficient studies from low- and middle-income countries. We also aimed to run sensitivity analyzes with odds ratio effect estimates only as opposed to all ratios combined, but there were not sufficient associations. Forest plots were generated for each meta-analysis performed and heterogeneity was assessed with I^2^ statistics accounting for the dependency among associations originating from the same study. Results are expressed as odds ratio and 95% confidence interval (OR, 95% CI). Analyzes were performed in R version 4.2.1 (Team, 2020), using the functions rma. mv and forest of the Metafor package (Viechtbauer, 2010).

In addition to the meta-analysis, we used vote counting based on the direction of effects as an alternative method to synthesize the available evidence (McKenzie et al.). This method categorizes and compares the number of associations showing that unfavorable social environments are associated with higher risk of cardiometabolic risk factors and the number of associations showing that unfavorable social environments are associated with lower risk of cardiometabolic risk factors. Criteria for associations to be included in vote counting were: i) being overall, rather than sex-specific associations; and ii) we could establish a direction of effect such that “unfavorable social environment” was associated with either a higher, equal or lower risk of cardiometabolic risk factors. In accordance with Cochrane’s recommendations (McKenzie et al.), statistical significance nor effect size were considered in the categorization. Results of vote counting are presented in a direction-of-effect plot.

## Results

**Fig. 1:**
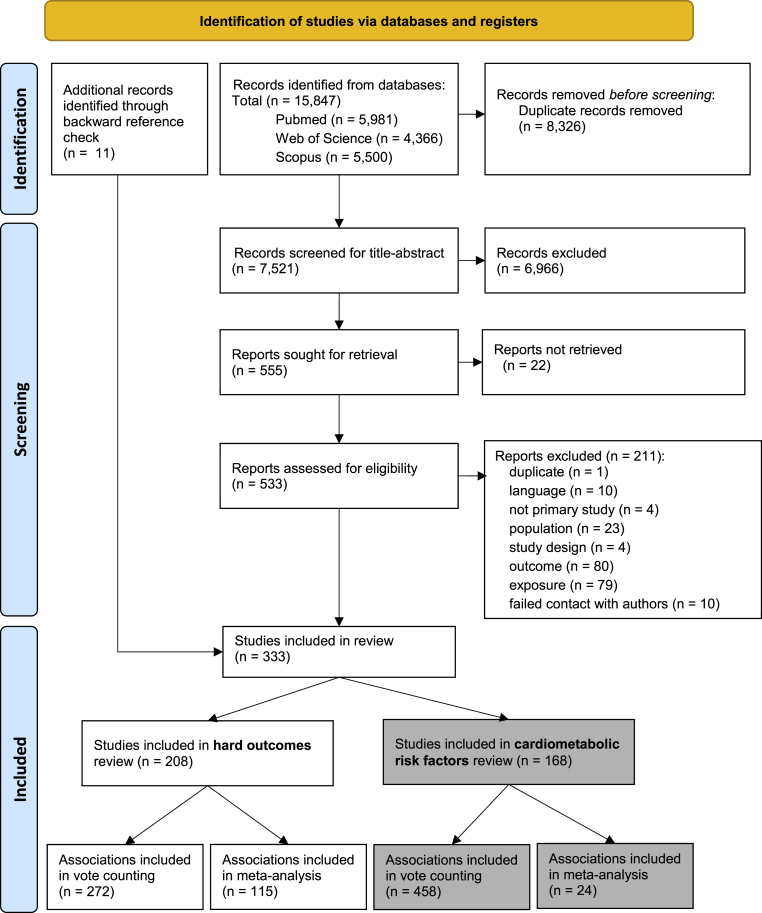
Flow diagram of study inclusion.

After screening 7521 titles and abstracts, 555 articles proceeded to full-text screening (Supplementary File 5). Of the 333 included articles, 208 were included in a review on hard outcomes reported elsewhere (Abreu et al. submitted elsewhere) and 168 are included in this review (some articles included both hard outcomes and risk factors of CMD) (see Fig. 1).

The large majority of studies were conducted in high income countries (95%), were cross-sectional in nature (74%) and were published in the last 10 years (64%) (Table 1). No qualitative nor experimental studies were included. The 168 included articles together described 1050 associations that were relevant for this review, of which 576 (49%) were overall associations, 317 (27%) were female-specific and 271 (23%) were male-specific (Supplementary Table 1).

**Table 1:** Characteristics of included studies, review on social environmental determinants of cardiometabolic risk factors.

Reference	First author, year	Study design	Population description	Type of environment	Sample Size	Average age (years)	Women (% of sample)	Country
Adams et al. (2009)	Adams, 2009	cross-sectional	general adult population	Collector’s districts (census collection units)	4060	na	na	Australia
Adjaye-Gbewonyo et al. (2018)	Adjaye-Gbewonyo, 2018	longitudinal	general adult population	district councils/district municipalities	9356	39.3 (SD 17.3)	63.00%	South Africa
Agyemang et al. (2007)	Agyemang, 2007	cross-sectional	general adult population	neighborhoods	1322	na	na	Netherlands
Aliarzadeh et al. (2014)	Aliarzadeh, 2014	cross-sectional	adult population enrolled in primary care providers	areas of residence	4870	62.5 (SD 11.2)	62.00%	Canada
Altevers et al. (2016)	Altevers, 2016	cross-sectional	general adult population	not applicable/non-geographic measure	8952	na	48.00%	Germany
Andell et al. (2020)	Andell, 2020	longitudinal	general adult population and echocardiography screening cohort with oversampling of patients with diabetes and glucose intolerance	administrative geographical units	Nationwide study population: 6,641,905; Malmo preventive project echocardiography screening cohort: 1586	Nationwide study population: na; Malmö cohort: 67.0 (SD 6.0)	Nationwide study population: 51%; Malmö cohort: na	Sweden
Andersen et al. (2008)	Andersen, 2008	cross-sectional	general female adult population	electoral wards	4286	na	100.00%	United Kingdom
Auchincloss et al. (2007)	Auchincloss, 2007	cross-sectional	general adult population	census blocks	4821	61.6 (SD 10.2)	53.00%	United States of America
Bagheri et al. (2015)	Bagheri, 2015	cross-sectional	general adult population	statistical areas of residence	4740	na	58.00%	South Australia
Bagheri, 2019	Bagheri, 2019	cross-sectional	general adult population	statistical areas of residence	55,693	na	59.00%	South Australia
Bajaj et al. (2016)	Bajaj, 2016	cross-sectional	Pittsburgh Healthy Heart Project: community-dwelling adult population and Adult Health and Behavior Project: general adult population	not applicable/non-geographic measure	Pittsburgh Healthy Heart Project: 306; Adult Health and Behavior Project: 419	PHHP: 60.7 (SD 4.8); AHAB-II: 42.9 (SD 7.3)	PHHP: 49%; AHAB-II: 53%	United States of America
Baldock et al. (2012)	Baldock, 2012	cross-sectional	general adult population	statistical areas of residence	1324	54.3 (SD 14.3)	54.00%	Australia
Baldock et al. (2018)	Baldock, 2018	cross-sectional	general adult population	suburbs	1172	male: 62.0 (SD 10.0); female: 62.0 (SD 10.0)	54.00%	South Australia
Barber et al. (2016a)	Barber, 2016	cross-sectional	African American adult population	census tracts	4408	54.5 (no SD)	64.00%	United States of America
Barber et al. (2016b)	Barber, 2016	cross-sectional	African American adult population	census tracts	4410	54.5 (no SD)	64.00%	United States of America
Barber et al. (2016c)	Barber, 2016	cross-sectional	African American adult population	census tracts	4096	na	na	United States of America
Bhopal et al. (2002)	Bhopal, 2002	cross-sectional	adult population	census district	2193	na	51.00%	United Kingdom
Bird et al. (2010)	Bird, 2010	cross-sectional	general adult population with oversampling of Black American and Mexican American population	census tracts	13,184	45.0 (no SD)	52.00%	United States of America
Bland et al. (1991)	Bland, 1991	cross-sectional	general adult population	not applicable/non-geographic measure	1409	male: 44.7 (SD 25.6); female: 43.3 (SD 13.6)	54.00%	United States of America
Bland et al. (2000)	Bland, 2000	cross-sectional	adult male factory workers	not applicable/non-geographic measure	693	44.4 (no SD)	0.00%	Italy
Boylan et al. (2017)	Boylan, 2017	longitudinal	general adult population and siblings of individuals from the RDD sample and a national RDD sample of twin pairs	census tracts	1012	58.1 (SD 11.6)	55.00%	United States of America
Breckenkamp et al. (2007)	Breckenkamp, 2007	cross-sectional	general adult population	regions	11,020	male: 45.6 (no SD); female: 46.5 (no SD)	53.00%	Germany
Browning et al. (2012)	Browning, 2012	cross-sectional	general adult population	census tracts	1410	48.5 (SD 8.8)	54.00%	United States of America
Bu, Steptoe, & Fancourt, 2021	Bu, 2021	cross-sectional	general adult population	not applicable/non-geographic measure	5947	na	na	United Kingdom
Carels et al. (1998)	Carels, 1998	cross-sectional	employed adult population	not applicable/non-geographic measure	126	na	43.00%	United States of America
Carson et al. (2007)	Carson, 2007	longitudinal	general adult population	neighborhoods	11,261	White male: 54.2 (no SD); White female: 53.7 (no SD); Black male: 52.8 (no SD); Black female: 52.6 (no SD)	57.00%	United States of America
Caspi et al. (2006)	Caspi, 2006	longitudinal	children followed up to adulthood	not applicable/non-geographic measure	841	na	47.00%	New Zealand
Cathorall et al. (2015)	Cathorall, 2015	cross-sectional	general adult population	census blocks	14,510	47.4 (SD 14.5)	65.00%	United States of America
Chaix et al. (2008)	Chaix, 2008	cross-sectional	general male adult population	areas of residence	7838	54.9 (SD 2.9)	0.00%	France
Chaix et al. (2010)	Chaix, 2010	cross-sectional	general adult population	neighborhoods	5941	na	na	France
Chaparro et al. (2018)	Chaparro, 2018	cross-sectional	general population	census area statistic ward	11,825	50.7 (SE 0.1)	56.00%	United Kingdom
Chichlowska et al. (2008)	Chichlowska, 2008	cross-sectional	general adult population	census tracts	12,709	Black female: 53.0 (SD 5.7); White female: 54.0 (SD 5.7); Black male: 54.0 (SD 6.0); White male: 55.0 (SD 5.7)	55.00%	United States of America
Clark et al. (2012)	Clark, 2012	cross-sectional	general female adult population	states	26,029	53.0 (IQR 49.0–59.0)	100.00%	United States of America
Clark et al. (2013)	Clark, 2013	cross-sectional	African American adult population	census tracts	3909	53.0 (IQR 44.0–63.0)	63.00%	United States of America
Claudel et al. (2018)	Claudel, 2018	longitudinal	general adult population with oversampling of African American population	census tracts	1174	40.1 (SD 9.6)	58.00%	United States of America
Climie et al. (2019)	Climie, 2019	cross-sectional	general adult population	communes	7803	na	38.00%	France
Cohn et al. (2017)	Cohn, 2017	cross-sectional	adult White Hispanic and non-Hispanic population	census tracts	3317	57.6 (no SD)	46.00%	United States of America
Coulon et al. (2016a)	Coulon, 2016	cross-sectional	African American adult population	census blocks	409	53.0 (SD 16.0)	62.00%	United States of America
Coulon et al. (2016b)	Coulon, 2016	cross-sectional	African American adult population	census blocks	208	55.6 (SD 15.2)	65.00%	United States of America
Cozier et al. (2016)	Cozier, 2016	cross-sectional	African American adult female population	neighborhoods	418	na	100.00%	United States of America
Creaven et al. (2013)	Creaven, 2013	cross-sectional	female student population	not applicable/non-geographic measure	144	19.2 (SD 1.4)	100.00%	Ireland
Cross et al. (2019)	Cross, 2019	cross-sectional	general adult population	statistical areas of residence	29,064	65.2 (SD 14.0)	47.00%	Australia
Cubbin et al. (2005)	Cubbin, 2005	cross-sectional	general adult population	neighborhoods	8197	na	55.00%	United States of America
De Moraes et al. (2019)	De Moraes, 2019	cross-sectional	general adult population	areas of residence	6792	62.2 (no SD)	53.00%	United States of America
Deans et al. (2009)	Deans, 2009	cross-sectional	general adult population	neighborhoods	666	na	50.00%	United Kingdom
Diez Roux, Jacobs, and Kiefe (2002)	Diez Roux, 2002	cross-sectional	general adult population	census blocks	3093	White male: 35.5 (SD 3.4); Black male: 34.2 (SD 3.8); White female: 35.7 (SD 3.4); Black female: 34.5 (SD 3.9)	55.00%	United States of America
Diez Roux et al. (2002b)	Diez Roux, 2002	longitudinal	general adult population	neighborhoods	8555	White male: 54.2 (no SD); white female: 53.3 (no SD); black male: 52.6 (no SD); black female: 52.0 (no SD)	56.00%	United States of America
Diez-Roux et al. (1997)	Diez-Roux, 1997	cross-sectional	general adult population	census blocks	12,601	na	55.00%	United States of America
Djekic et al. (2018)	Djekic, 2018	cross-sectional	general adult population	districts	1067	57.7 (SD 4.4)	51.00%	Sweden
Do et al. (2011)	Do, 2011	cross-sectional	general adult population	neighborhoods	892	66.0 (no IQR)	53.00%	United States of America
Dragano et al. (2009)	Dragano, 2009	cross-sectional	general adult population	neighborhoods	4301	59.4 (SD 7.8)	53.00%	Germany
Dubowitz et al. (2012)	Dubowitz, 2012	cross-sectional	female population	census tracts	60,775	68.2 (SD 7.3)	100.00%	United States of America
Duncan et al. (2016)	Duncan, 2016	cross-sectional	low-income adult population	census blocks	116	na	56.00%	United States of America
Dwane et al. (2020)	Dwane, 2020	cross-sectional	general population	health districts	25,532	na	58.00%	South Africa
Eichinger et al. (2015)	Eichinger, 2015	cross-sectional	general adult population	not applicable/non-geographic measure	904	na	42.00%	Austria
Ellaway et al. (2007)	Ellaway, 2007	cross-sectional	na	not applicable/non-geographic measure	2334	cohort 1: 30.0 (no SD); cohort 2: 50.0 (no SD); cohort 3: 68.0 (no SD)	55.00%	United Kingdom
Engström et al. (2001)	Engstrom, 2001	cross-sectional	general adult population	areas of residence	28,466	na	60.00%	Sweden
Ferguson et al. (2020)	Ferguson, 2020	cross-sectional	adult population	communities	2556	17.9 (SD 2.0)	57.00%	Jamaica
Finch et al. (2010)	Finch, 2010	cross-sectional	general adult population	census tracts	13,827	na	na	United States of America
Foraker et al. (2019)	Foraker, 2019	cross-sectional	African American adult population	census tracts	3667	55.1 (IQR 45.0–64.4)	64.00%	United States of America
Ford et al. (2006)	Ford, 2006	cross-sectional	general adult population	not applicable/non-geographic measure	14,818	male: 44.0 (SE 0.4); female: 45.7 (SE 0.5)	53.00%	United States of America
Ford et al. (2019)	Ford, 2019	cross-sectional	non-Hispanic Black adult female population	not applicable/non-geographic measure	1829	28.4 (SE 0.2)	100.00%	United States of America
Fuller et al. (2018)	Fuller, 2018	cross-sectional	African American adult population	not applicable/non-geographic measure	138	41.0 (no SD)	67.00%	United States of America
Gallo et al. (2012)	Gallo, 2012	cross-sectional	Mexican American adult female population	census tracts	284	49.7 (SD 6.5)	100.00%	United States of America
Garcia et al. (2015)	Garcia, 2015	cross-sectional	Latino adult population	census tracts	1777	70.7 (SD 7.1)	59.00%	United States of America
Garcia et al. (2016)	Garcia, 2016	longitudinal, followed for up to 10 years	Latino adult population	tracts	1777	diabetes group: 70.3 (SD 6.9); pre-diabetes group: 69.8 (SD 6.9); no diabetes group: 71.2 (SD 7.3)	na	United States of America
Gary-Webb et al. (2020)	Gary-Webb, 2020	longitudinal	African American adult population	neighborhoods	622	58.0 (no SD)	79.00%	United States of America
Gebreab et al. (2015)	Gebreab, 2015	cross-sectional	general adult population	states	281,198	na	50.00%	United States of America
Grimaud et al. (2013)	Grimaud, 2013	cross-sectional	adult population	neighborhoods	5474	73.3 (SD 4.9)	63.00%	France
Halonen et al. (2015)	Halonen, 2015	longitudinal	public sector workers	neighborhoods	37,699	49.9 (SD 10.4)	79.00%	Finland
Hamad et al. (2020)	Hamad, 2020	longitudinal	adult immigrants	neighborhoods (or parishes)	49,305	30.5 (IQR 24.9–39.8)	43.00%	Denmark
Hanson et al. (1988)	Hanson, 1988	cross-sectional	general male adult population	not applicable/non-geographic measure	485	na	0.00%	Sweden
Helminen et al. (1995a)	Helminen, 1995	cross-sectional	general male adult population	not applicable/non-geographic measure	212	55.1 (95%CI 54.7–55.5)	0.00%	Finland
Helminen et al. (1995b)	Helminen, 1995	cross-sectional	general male adult population	not applicable/non-geographic measure	108	na	0.00%	Finland
Helminen et al. (1997)	Helminen, 1997	cross-sectional	general male adult population	not applicable/non-geographic measure	212	55.1 (95%CI 54.7–55.5)	0.00%	Finland
Henning et al. (2014)	Henning, 2014	cross-sectional	general population with partly obese population	not applicable/non-geographic measure	667	51.0 (SD 14.5)	68.00%	Germany
Hickson et al. (2011)	Hickson, 2011	cross-sectional	African American adult population	census tracts	322	59.2 (SD 10.7)	69.00%	United States of America
Hilding et al. (2015)	Hilding, 2015	longitudinal	general adult population enriched with participants with family history for diabetes	not applicable/non-geographic measure	4963	na	59.00%	Sweden
Höfelmann et al. (2012)	Hofelmann, 2012	cross-sectional	general adult population	census tracts	1720	38.1 (95% CI 37.5–38.6)	56.00%	Brazil
Holmes et al. (2012)	Holmes, 2012	cross-sectional	legal and unauthorized Brazilian adult migrants	census blocks	151	33.5 (SD 9.6)	na	United States of America
Horsten et al. (1999)	Horsten, 1999	cross-sectional	general female adult population	not applicable/non-geographic measure	300	56.0 (SD 7.0)	100.00%	Sweden
Hosseini, 2020	Hosseini, 2020	cross-sectional	general adult population	not applicable/non-geographic measure	28,238	62.8 (SD 10.2)	51.00%	Canada
Hughes (2007)	Hughes, 2009	cross-sectional	female adult population	not applicable/non-geographic measure	211	25.3 (SD 8.8)	100.00%	Ireland
Islam et al. (2020)	Islam, 2020	cross-sectional	Black adult population	census tracts	394	52.8 (SD 10.3)	61.00%	United States of America
Jimenez et al. (2019)	Jimenez, 2019	longitudinal	children followed up to adulthood	census tracts	671	44.2 (SD 2.9)	59.00%	United States of America
Kakinami et al. (2017)	Kakinami, 2017	longitudinal	children followed up into adulthood	neighborhoods	3820	boys 9.6 (SD 2.6) and girls 9.4 (SD 2.6) at baseline	50.00%	Canada
Keita et al. (2014)	Keita, 2014	cross-sectional	general adult population with oversampling of African American population	census blocks	19,079	na	na	United States of America
Kelli et al. (2017)	Kelli, 2017	cross-sectional	general adult population	zip codes	1421	49.4 (SD 10.2)	62.00%	United States of America
Kent de Grey et al. (2019)	Kent de Grey, 2019	cross-sectional	general adult population and undergraduate students	not applicable/non-geographic measure	139	22.0 (SD 5.8)	68.00%	United States of America
Kershaw et al. (2017)	Kershaw, 2017	longitudinal	non-Hispanic Black adult population	census tracts	2280	na	57.00%	United States of America
Kim et al. (2010)	Kim, 2010	longitudinal	general adult population	neighborhoods	2974	45.3 (no SD)	57.00%	United States of America
Kim et al. (2020)	Kim, 2020	cross-sectional	community based and hospital-based adult population	not applicable/non-geographic measure	10,103	Community based population: male controls: 50.6 (SD 9.6); male cases: 51.2 (SD 8.7); Female controls: 51.0 (SD 8.5); Female cases: 55.2 (SD 6.3). Hospital based population: male controls: 52.2 (SD 8.4); male cases: 52.8 (SD 8.2); female controls: 52.9 (SD 8.2); female cases: 54.5 (SD 7.8)	61.00%	Korea
King et al. (2011)	King, 2011		general adult population	neighborhoods	549	43.0 (no SD)	58.00%	United States of America
Kivimäki et al. (2018)	Kivimaki, 2018	longitudinal	children followed up into adulthood	neighborhoods	3002	10.9 (SD 4.4) at baseline	52.00%	Finland
Lawlor et al. (2005)	Lawlor, 2005	cross-sectional	general female adult population	electoral wards	4286	68.9 (no SD)	100.00%	United Kingdom
Lee et al. (2020)	Lee, 2020	cross-sectional	general adult population with oversampling of Black/African Americans, Hispanics, men, and older adults	not applicable/non-geographic measure	2731	67.7 (SD 7.6)	52.00%	United States of America
Lei et al. (2018)	Lei, 2018	longitudinal	African American adult population	census tracts	408	10.6 (SD 0.6) at wave 1	63.00%	United States of America
Lemelin et al. (2009)	Lemelin, 2009	longitudinal	general adult population	census tracts	4423	62.0 (no SD)	52.00%	United States of America
Lewis et al. (2010)	Lewis, 2010	cross-sectional	general adult population	not applicable/non-geographic measure	2454	73.7 (SD 2.9)	53.00%	United States of America
Li et al. (2017)	Li, 2017	cross-sectional	Latino adult population	census tracts	1563	41.8 (SE 15.0)	69.00%	United States of America
Li et al. (2019)	Li, 2019	cross-sectional	general adult population	census tracts	10,122	41.2 (SE 0.2)	50.00%	United States of America
Linden et al. (1993)	Linden, 1993	cross-sectional	student population	not applicable/non-geographic measure	129	19.7 (no SD)	57.00%	United States of America
Lippert et al. (2017)	Lippert, 2017	longitudinal	adolescents followed up into adulthood	neighborhoods	11,767	15.4 (SE 0.0)	51.00%	United States of America
Loose et al. (2017)	Loose, 2017	cross-sectional	North African adult population	not applicable/non-geographic measure	82	31.4 (SD 11.1)	46.00%	France
Loucks et al. (2005)	Loucks, 2005	cross-sectional	general adult population	not applicable/non-geographic measure	800	male: 74.1 (SD 2.7); female: 74.4 (SD 2.7)	53.00%	United States of America
Loucks et al. (2006a)	Loucks, 2006	cross-sectional	general adult population	not applicable/non-geographic measure	805	male: 74.1 (SD 2.7); female: 74.4 (SD 2.7)	53.00%	United States of America
Loucks et al. (2006b)	Loucks, 2006	cross-sectional	general adult population with oversampling of White adult population	not applicable/non-geographic measure	3231	male 62.0 (SD 10.0); female 62.0 (SD 10.0)	54.00%	United States of America
Maki (2020)	Maki, 2020	cross-sectional	general adult population	not applicable/non-geographic measure	926	55.2 (SD 11.5)	37.00%	United States of America
Marley et al. (2015)	Marley, 2015	longitudinal	adolescents followed up into adulthood	neighborhoods	11,110	na	na	United States of America
Martin et al. (2019)	Martin, 2019	longitudinal	adolescents followed up into adulthood	census tracts	9500	15.4 (SE 0.1) in 1994	52.00%	United States of America
Matricciani et al. (2013)	Matricciani, 2013	cross-sectional	general adult population	area of residence (state suburb)	1352	48.5 (SD 14.6)	normotensive: 60%	Australia
Mayne et al. (2018)	Mayne, 2018	longitudinal	general adult population	neighborhood (street level or zip code)	528	60.4 (SD 9.6) at baseline	55% at baseline	United States of America
Mayne et al. (2019)	Mayne, 2019	longitudinal	general adult population	census tracts	5015	White: 60.5 (SD 7.2); Black 59.3 (SD 7.3)	47.00%	United States of America
McKenzie et al. (2020)	McKenzie, 2020	cross-sectional	adult population	communities	1025	47.0 (SD 17.5)	65.00%	Jamaica
Mellman et al. (2015)	Mellman, 2015	cross-sectional	African American adult population	census tracts	136	23.1 (SD 4.7)	54.00%	United States of America
Merkin et al. (2009)	Merkin, 2009	cross-sectional	general adult population	neighborhoods	13,199	41.0 (no IQR)	52.00%	United States of America
Merkin et al. (2020)	Merkin, 2020	longitudinal	general adult population	census tracts	5750	na	na	United States of America
Merlo et al. (2001)	Merlo, 2001	cross-sectional	general female adult population	areas of residence	15,569	na	100.00%	Sweden
Metcalf et al. (2008)	Metcalf, 2008	cross-sectional	general adult population	areas of residence	4020	na	52.00%	New Zealand
Meza et al. (2020)	Meza, 2020	cross-sectional	low-income adult population	not applicable/non-geographic measure	259	44.4 (SD 12.5)	86.00%	United States of America
Mobley et al. (2006)	Mobley, 2006	cross-sectional	uninsured low-income female adult population	zip codes	2692	50.0 (no SD)	100.00%	United States of America
Murakami et al. (2010)	Murakami, 2010	cross-sectional	adult female dietetic students	municipalities	1081	na	100.00%	Japan
Murray et al. (2010)	Murray, 2010	longitudinal	general female adult population	census tracts	2561	62.4 (no SD) for male and female combined	100.00%	United States of America
Naimi et al. (2009)	Naimi, 2009	cross-sectional	general adult population	census tracts	342	male: 35.8 (SD 8.9); female: 33.9 (SD 8.5)	51.00%	Canada
Nazmi et al. (2010)	Nazmi, 2010	longitudinal (& cross-sectional)	general adult population	census tracts	Cross-sectional analysis: 5370; longitudinal subsample: 946	62.2 (SD 10.2)	na	United States of America
Neergheen et al. (2019)	Neergheen, 2019	cross-sectional	African American and White adult population	not applicable/non-geographic measure	379	na	66.00%	United States of America
Ngo et al. (2013)	Ngo, 2013	longitudinal	general adult population	state suburbs	1877	47.0 (SD 15.5) at wave 1	56% at wave 1	Australia
Ngo et al. (2014)	Ngo, 2014	longitudinal	general adult population	state suburbs	2619	50.0 (no SD)	52.00%	Australia
Nikulina et al. (2014)	Nikulina, 2014	longitudinal	children followed up into adulthood	census tracts	539	41.0 (no SD)	51.00%	United States of America
Nordstrom et al. (2004)	Nordstrom, 2004	cross-sectional	general adult population	census blocks	3545	72.4 (SD 5.4)	62.00%	United States of America
Pedersen et al. (2016)	Pedersen, 2016	longitudinal	general adult population	not applicable/non-geographic measure	3621	male: 49.0 (no SD); female: 52.0 (no SD)	61.00%	Denmark
Petersen et al. (2008)	Petersen, 2008	cross-sectional	general adult population	census tracts	851	44.9 (SD 6.5)	50.00%	United States of America
Piferi et al. (2006)	Piferi, 2006	cross-sectional	undergraduate student population	not applicable/non-geographic measure	96	19.2 (SD 1.4)	58.00%	United States of America
Pollack et al. (2012)	Pollack, 2012	cross-sectional	general adult population	census tracts	6484	47.0 (SE 0.2)	52.00%	United States of America
Pollard et al. (2003)	Pollard, 2003	cross-sectional	South Asian and European adult population	not applicable/non-geographic measure	1509	na	na	United Kingdom
Pollitt et al. (2007)	Pollitt, 2007	longitudinal	general adult population	county and census tracts	11,842	White 53.9 (SD 5.6); African American 52.7 (SD 5.6)	na	United States of America
Pollitt et al. (2008)	Pollitt, 2008	longitudinal	general adult population	county and census tracts	11,842	White female: 53.7 (SD 5.6); White male: 54.3 (SD 5.6); African American female: 52.6 (SD 5.6); African American male: 52.8 (SD 5.7)	57.00%	United States of America
Ribeiro et al. (2019)	Ribeiro, 2019	cross-sectional	general adult population	neighborhoods	16,364	CoLaus: 57.8 (SD 10.5); EPIPorto: 52.9 (SD 15.5); Whitehall: 50.3 (SD 6.1)	43.00%	Switzerland, Portugal and United Kingdom
Riva et al. (2016)	Riva, 2016	cross-sectional	general adult population	communities	3108	44.4 (SD 14.8)	56.00%	Greenland
Robinette et al. (2016)	Robinette, 2016	cross-sectional	adult population consisting of random sample, twins and siblings	census tracts	995	55.2 (SD 11.8)	54.00%	United States of America
Robinette et al. (2020)	Robinette, 2020	cross-sectional	non-Hispanic White adult population	not applicable/non-geographic measure	6615	69.7 (SE 0.1)	54.00%	United States of America
Rosvall et al. (2007)	Rosvall, 2007	cross-sectional	general adult population	geographical areas	4033	na	na	Sweden
Samuel et al. (2015)	Samuel, 2015	cross-sectional	general adult population	census tracts	1326	White: 43.8 (SD 16.1); African American: 38.3 (SD 13.2)	White: 57%; African American: 53%	United States of America
Schulz et al. (2013)	Schulz, 2013	cross-sectional	general adult population with oversampling of African American and Latino population	census blocks	919	46.3 (SE 0.8)	52.00%	United States of America
Seeman et al. (2014)	Seeman, 2014	cross-sectional	general adult population	not applicable/non-geographic measure	782	40.0 (no SD)	58.00%	United States of America
Smith et al. (1998)	Smith, 1998	cross-sectional	general adult population	postcode sectors	14,952	na	53.00%	United Kingdom
Sörman et al. (2016)	Sorman, 2016	longitudinal	general adult population	not applicable/non-geographic measure	1097	62.4 (SD 11.1) at baseline	55.00%	Sweden
Sprung et al. (2019)	Sprung, 2019	cross-sectional	adult population	neighborhoods	1718	male: 47.8 (no SD); female: 47.1 (no SD)	54.00%	United States of America
Steppuhn et al. (2019)	Steppuhn, 2019	cross-sectional	general adult population	municipalities	6768	47.4 (SD 16.6)	50.00%	Germany
Strogatz et al. (1997)	Strogatz, 1997	cross-sectional	Black adult population	not applicable/non-geographic measure	1750	na	63.00%	United States of America
Theorell et al. (1982)	Theorell, 1982	cross-sectional	general male adult population	areas of residence	74,000	all participants were age 18	0.00%	Sweden
Toms et al. (2020)	Toms, 2020	cross-sectional	general adult population	statistical areas of residence	256,525	na	na	Australia
Troxel et al. (2010)	Troxel, 2010	cross-sectional	adult population with high/moderate or low Framingham risk scores	not applicable/non-geographic measure	224	White: 60.5 (SD 7.2); Black 59.3 (SD 7.3)	White: 40%; Black: 62%	United States of America
Tung et al. (2019)	Tung, 2019	longitudinal	adult patients of a academic medical center	census tracts	17,783	58.0 (IQR 41.0–71.0)	68.00%	United States of America
Uchino et al. (2013)	Uchino, 2013	cross-sectional	adult couples	not applicable/non-geographic measure	188	29.6 (no SD)	na	United States of America
Unger et al. (2014)	Unger, 2014	cross-sectional	general adult population	census tracts	5649	61.7 (SD 10.1)	52.00%	United States of America
Wagner et al. (2016)	Wagner, 2016	cross-sectional	general adult population	census tracts	1705	70.7 (SD 8.0)	64.00%	Brazil
Whittaker et al. (2012)	Whittaker, 2012	cross-sectional	general female adult population	not applicable/non-geographic measure	493	57.6 (SD 11.4)	100.00%	United States of America
Willets et al. (2019)	Willets, 2019	cross-sectional	general adult population	neighborhoods	9923	51.5 (SD 8.9)	56.00%	Brazil
Williams et al. (2012)	Williams, 2012	longitudinal	general adult population	census tracts	4572	na	na	Australia
Wing et al. (2016)	Wing, 2016	longitudinal	general adult population	neighborhoods	5950	57.9 (SD 9.1)	63.00%	United States of America
Yang et al. (2013)	Yang, 2013	longitudinal	general adult population	not applicable/non-geographic measure	4323	53.7 (SD 10.0)	55.00%	United States of America
Yang et al. (2014)	Yang, 2014	longitudinal	general adult population	not applicable/non-geographic measure	647	55.4 (SD 11.3)	50.00%	United States of America
Yang et al. (2015)	Yang, 2015	longitudinal	general adult population	not applicable/non-geographic measure	1264	67.3 (SD 7.8)	53.00%	United States of America
Yang et al. (2016)	Yang, 2016	longitudinal	general adult population	not applicable/non-geographic measure	Adolescence and Young Adulthood: 7889; Midadulthood: 863; and Late Adulthood: 1571 and 4323	Adolescence and young Adulthood: 28.2 (SD 1.9); Midadulthood: 44.0 (SD 9.9); Late Adulthood: 67.3 (SD 10.5) and 67.3 (SD 7.1)	Adolescence and Young Adulthood: 48%; Midadulthood: 55%; and Late Adulthood: 55% and 53%	United States of America
Yao et al. (2019)	Yao, 2019	longitudinal	general adult population	communities	16,547	46.0 (SD 14.4)	na	China
Zanelatto et al. (2019)	Zanelatto, 2019	cross-sectional	general adult population	census tracts	1720	38.0 (SD 11.6)	55.00%	Brazil
Zöller et al. (2012)	Zoller, 2013	longitudinal	general adult population	statistical areas of residence	4,266,289	na	50.00%	Sweden

The average percentage of quality assessment points scored was 57% for cross-sectional and 79% for longitudinal studies. Many cross-sectional studies scored low on the “selection” domains “sample size” and “non-respondents” (see Fig. 2a). Cohort studies generally scored low on the “selection” domains “representativeness” and “correction for presence of the outcome at baseline” (see Fig. 2b). Of all studies included, 20 (12%) were considered low quality, which were subsequently excluded from vote counting and meta-analyses.

**Fig. 2a:**
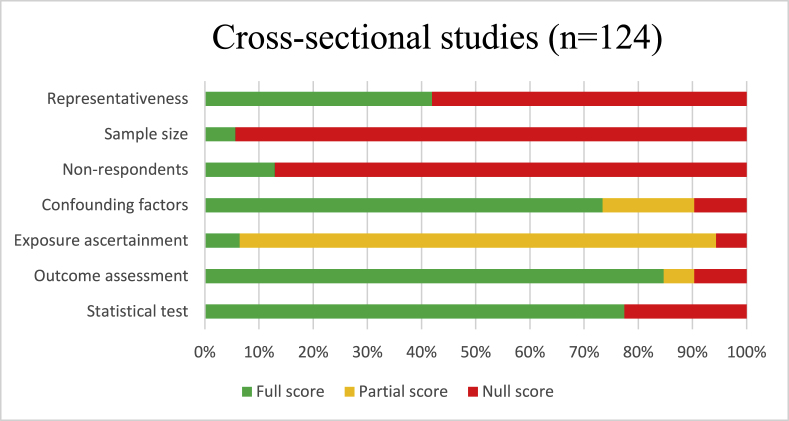
Quality of cross-sectional studies included based on the New-Ottawa Castle tool Quality Assessment Scale adapted for cross-sectional studies, review on social environmental determinants of cardiometabolic risk factors.

**Fig. 2b:**
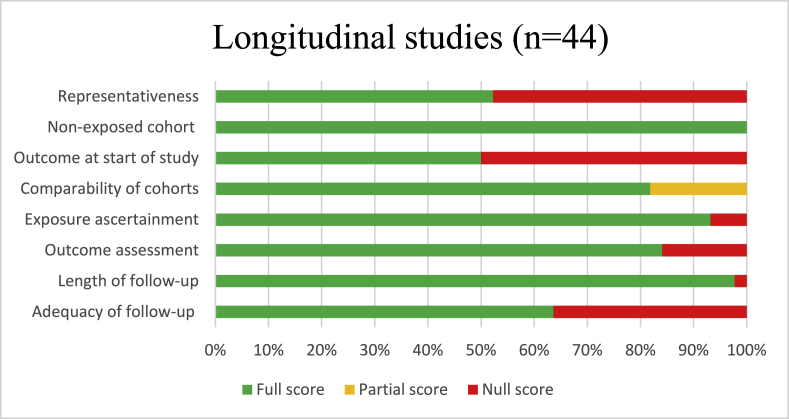
Quality of cohort studies included based on the New-Ottawa Castle tool Quality Assessment Scale, review on social environmental determinants of cardiometabolic risk factors.

**Fig. 3:**
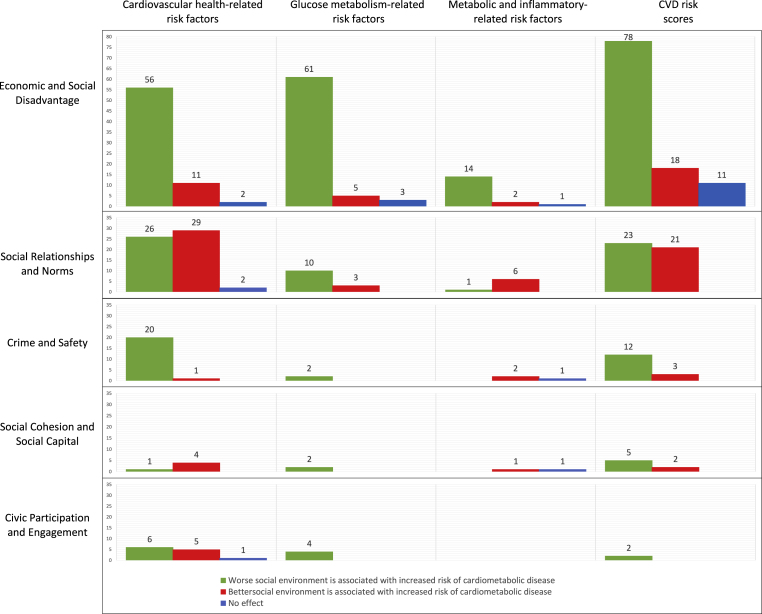
Overview of vote counts based on direction of effect for associations between dimensions of the social environment and cardiometabolic risk factors.

Of the 1050 unique associations, 167 were from low quality studies, 377 were sex-specific associations and for 43 the direction of effect could not be determined. Of the 458 associations included in the vote counting, 323 (71%) pointed towards unfavorable social environments being associated with higher risk of cardiometabolic risk factors (see Fig. 3). Of the four cardiometabolic risk factors categories considered, most associations used “CVD risk scores” such as Cumulative Biological Risk or a cardiovascular health risk score as outcome (n = 175, 38%), followed by “cardiovascular health-related risk factors” such as systolic and diastolic blood pressure (n = 164, 36%), “glucose metabolism-related risk factors” such as HbA1c or HOMA (n = 90, 20%), with least associations for “metabolic and inflammatory risk factors” such as CRP or IL-6 (n = 29, 6%). Two-hundred-sixty-two of the 458 associations (57%) were on the social environment dimension “Social and Economic Disadvantage”, which consistently showed that being exposed to more social and economic disadvantage is associated with less favorable cardiometabolic risk factors profiles. Similar consistent associations were found for the domain “Crime and Safety”: 34 out of 41 associations (83%) suggested that higher levels of crime and less safety was associated with higher risk of cardiometabolic risk factors. Associations in the dimension “Social Relationships and Norms” were considerably less consistent, with only 60 out of 121 associations pointing towards the direction of disadvantageous social relationships and norms being associated with less favorable cardiometabolic risk factors profiles. There was little evidence for associations of “Social Cohesion and Social Capital” (16 associations) and “Civic Participation and Engagement” (18 associations). There were no eligible associations from the dimensions “Sense of Place/Belonging” and “Disorder and Incivilities”.

**Fig. 4:**
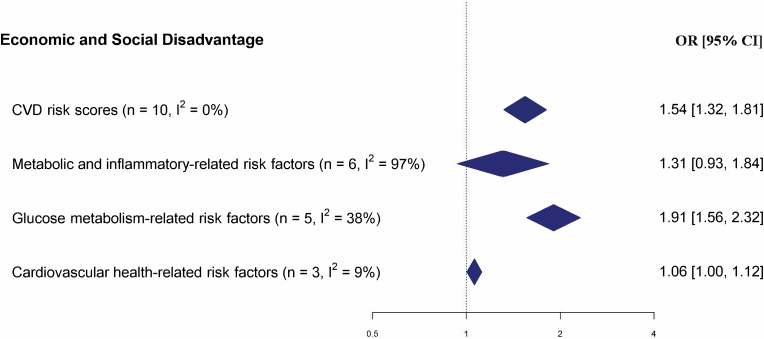
Summary of forest plots (random-effects model) for the meta-analyses of social environment dimensions and cardiometabolic risk factors.

Meta-analysis was possible for only 24 associations across four exposure-outcome combinations that had a dichotomous outcome and categorized exposure measure. As such, only one out of eight social environmental domains was represented in the overall meta-analyses (see Fig. 4, Supplementary Fig. 1 and Table 2). In all four meta-analyses “Economic and Social Disadvantage” was associated with increased cardiometabolic risk, but one was not statistically significant. Individuals that experienced more economic and social disadvantage had higher “CVD risk scores” (OR = 1.54, 95%CI: 1.35; 1.84), higher “glucose metabolism-related risk factors” (OR = 1.91, 95%CI: 1.56; 2.32) and somewhat higher “cardiovascular health-related risk factors” (OR = 1.06, 95%CI: 1.00; 1.12). Only one of four meta-analyses had high heterogeneity, namely for the outcome “metabolic and inflammatory-related risk factors” with six associations included (I^2^ = 97%).

**Table 2:** Summary pooled effects and between-study variance estimates with 95% confidence intervals from meta-analyses covering the social environmental determinants of cardiometabolic risk factors.

Exposure	Outcome	n	Odds ratio (95% CI)		Total I^2^
*Economic and Social Disadvantage*	*CVD risk scores*	10	1.54 (1.32–1.81)	***	0%
*Economic and Social Disadvantage*	*Metabolic and inflammatory-related risk factors*	6	1.31 (0.93–1.84)		97%
*Economic and Social Disadvantage*	*Glucose metabolism-related risk factors*	5	1.91 (1.56–2.32)	***	38%
*Economic and Social Disadvantage*	*Cardiovascular health-related risk factors*	3	1.06 (1.00–1.12)	*	0%

‘***’ = p ≤ 0.001; ‘**’ = p ≤ 0.01; ‘*’ = p ≤ 0.05 References of included studies.

It was possible to perform four sex-specific meta-analyses for the same combination of exposure-outcome for both men and women (Fig. 5a, Fig. 5b, Fig. 6a, Fig. 6bb and Supplementary Table 2). Two of those, namely “Economic and Social Disadvantage” with “cardiovascular health-related risk factors” and with “CVD risk scores”, were also performed with overall estimates and therefore could be compared (Fig. 4, Supplementary Fig. 1 and Table 2). The association between “Economic and Social Disadvantage” and “cardiovascular health-related risk factors” was similar for the sex-specific meta-analysis as for the overall meta-analysis. However, whereas exposure to more economic and social disadvantage was overall associated with higher “CVD risk scores” (OR = 1.54, 95%CI: 1.35; 1.84, Table 2), this was not the case for men (OR = 0.59, 95%CI: 0.05; 6.58) and women (OR = 0.98, 95%CI: 0.34; 2.78) separately. It is important to note that the extremely wide confidence intervals and high heterogeneity for the OR for “Economic and Social Disadvantage” and “CVD risk scores” among men suggests there is serious imprecision in this pooled effect estimate.

**Fig. 5a:**
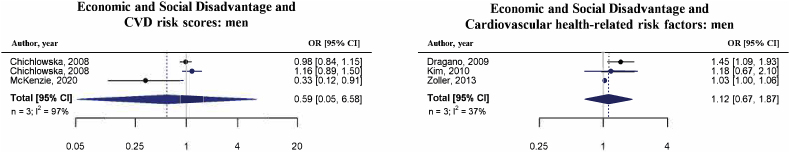
Summary of forest plots (random-effects model) for the meta-analyses of economic and social disadvantage and cardiometabolic risk factors among men.

**Fig. 5b:**
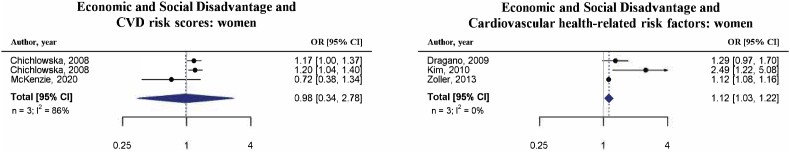
Summary of forest plots (random-effects model) for the meta-analyses of economic and social disadvantage and cardiometabolic risk factors among women.

**Fig. 6a:**
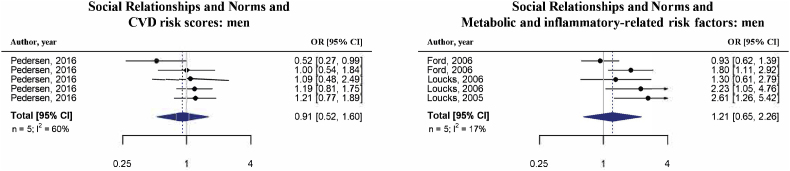
Summary of forest plots (random-effects model) for the meta-analyses of social relationships and norms and cardiometabolic risk factors among men.

**Fig. 6b:**
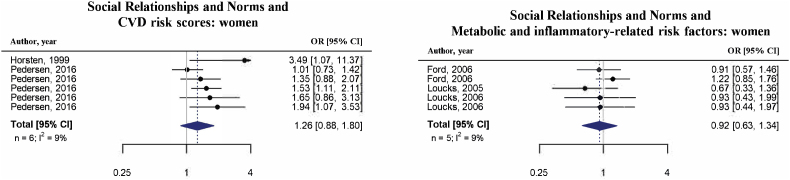
Summary of forest plots (random-effects model) for the meta-analyses of social relationships and norms and cardiometabolic risk factors among women.

Two of the sex-specific meta-analyses covering “Social Relationships and Norms” could not be compared with overall estimates. None of these sex-specific meta-analyses were statistically significant and direction of associations differed both between outcomes and men and women (see Fig. 6a, Fig. 6ba and b and Supplementary Table 2). For example, more favorable social relationships and norms were associated with lower “CVD risk scores” for men (OR = 0.91, 95%CI: 0.51; 1.60) and with higher “CVD risk scores” for women (OR = 1.26, 95%CI: 0.88; 1.80).

## Discussion

This systematic review and meta-analysis including a total of 168 studies reporting on 1050 associations indicates that unfavorable social environments are associated with higher cardiometabolic risk factors. Most associations focused on aspects of “Social and Economic Disadvantage” such as area-level deprivation, for which the evidence consistently pointed towards an adverse effect on cardiometabolic risk factors. Sex-specific associations showed inconsistent results, with both similarities and differences between associations for men and women, as well as similarities and differences in comparison with overall effect estimates. For other aspects of the social environment such as “Civic Participation and Engagement” limited evidence was found. Through this work we provide the most comprehensive overview to date of the literature on the social environmental determinants of cardiometabolic risk factors.

Our twin review on hard CMD outcomes (Abreu et al. submitted elsewhere) similarly showed that being exposed to a worse social environment is consistently associated with increased risk of CMD, with most evidence available for the dimension “Economic and Social Disadvantage”. The dominance of area-level disadvantage has also been observed in other reviews that considered a range of social environmental dimensions (Kepper et al., 2019; Suglia et al., 2016). The second-most studied social environmental dimension in this review was “Social Relationships and Norms”, with much less evidence for other social environmental dimensions.

It is challenging to compare the direction and strength of associations across the four categories of CMD risk factors we used in our data synthesis. Meta-analyses suggest that adverse social and economic circumstances are more strongly associated with “glucose metabolism-related risk factors” than with “CVD risk scores” or “cardiovascular health-related risk factors”, but this was based on a small number of associations only. Vote counting also demonstrate that the large majority (88%) of studies using “glucose metabolism-related risk factors” as outcome found associations in the expected direction, which was a stronger indication for the direction of the evidence than for “cardiovascular health-related risk factors” (67%), “CVD risk scores” (68%) and “metabolic and inflammatory-related risk factors” (52%).

Our results are partly in line with findings from previous reviews and meta-analyses that studied specific dimensions of the social environment or specific cardiometabolic risk factors. The results from another systematic review (Lovasi et al., 2009) suggest that neighborhood safety might be an important factor in decreasing obesity in more disadvantaged populations. We were unable to meta-analyze the association between the social environment dimension “Crime and Safety” in relation to “cardiovascular health-related risk factors”. However, the vote counting suggests that increased neighborhood crime is negatively associated with “cardiovascular health-related risk factors” and “CVD risk scores” outcomes, which is in line with earlier studies (Lovasi et al., 2009).

A meta-analysis including 21 studies found that living in neighborhood of low socioeconomic status was associated with a 31% higher odds of overweight and 45% higher odds of obesity compared to living in a neighborhood of high socioeconomic status (Mohammed et al., 2019). This study did unfortunately not stratify for sex while we found differences between the overall and sex-specific analyses. Sex differences have also been observed for associations between neighborhood environments and health (Macintyre et al., 2005), whereby worse economic indicators, like unemployment, on neighborhood level were associated with significantly better self-rated health in women but not in men. The authors suggest that this may be due to either women spending more time in their local neighborhoods than men, or a greater vulnerability to adverse neighborhood features among women. These explanations might help understand the large difference in the overall association on “Social and Economic Disadvantage” and “CVD risk scores” and the sex specific associations. While we were unable to include studies pertaining to “Segregation and Discrimination” at a contextual (i.e., community) level in our vote count and meta-analyses, a review on individual-level racial discrimination found a small but positive association with hypertensive status but not with resting blood pressure or diastolic blood pressure (Dolezsar et al., 2014). It needs to be noted that for some aspects of the social environment it is difficult to distinguish between individual-level and contextual dimensions of the social environment, and this is especially true for aspects such as discrimination. At the same time, it is challenging to operationalize social environment measures as contextual variables generally, which has been discussed in detail elsewhere (Oberndorfer et al., 2022).

Our vote counting results are also in line with a recent meta-analysis by Uchino et al. (Uchino et al., 2022) who found that perceived social support as individual-level construct was not significantly associated with ambulatory blood pressure. In another meta-analysis of Uchino et al. (Uchino et al., 2018), better social support and social integration were associated with lower levels of inflammatory cytokines. We were unable to reproduce a similar finding in the vote counting and stratified analyzes and even observed elevated, albeit non-significant, inflammatory values for men. However, it should be noted that Uchino et al. (Uchino et al., 2018) included 47 associations in their meta-analyses whereas we only included three due to the decision to only include associations with dichotomized exposures and outcomes. It is noteworthy that our vote counting results also points towards an unfavorable association of “Social Relationships and Norms” with “metabolic and inflammatory-related risk factors”. This may be attributed to the fact that there may be a ceiling effect in the beneficial effects of the number of social relations, i.e., after a certain number of social relations is reached, one extra social relation does not result in reduced risk.

### Strengths & limitations

The findings of this review and meta-analysis should be seen in the light of its limitations. First, due to the heterogeneity in social environment factors and the complexity of harmonizing continuous data, the meta-analysis was only limited to dichotomous outcomes. This resulted in a small number of associations that could be included in the meta-analyses which limits the generalizability of our results. An approach to pool different measures would be the standardized mean difference (SMD), which converts the results of the associations in a standardized measure before they can be combined in a unitless measure of pooled results, of which the disadvantage is its interpretability.

To reduce the impact of this limitation in our results, we performed complementary vote counting, which enabled us to give a visual and inclusive summary of the data. However, vote counting provides no information on the magnitude of the associations, does not account for differences in relative sizes of studies and is less powerful than meta-analysis (Borenstein et al., 2009). Secondly, as also observed in other reviews (Kepper et al., 2019; Mohammed et al., 2019), the high heterogeneity across studies in terms of measurement of exposures and outcomes, and adjustment for covariates hampered the comparison of retrieved data. Therefore, we advise readers to reflect on the results of the meta-analysis and its limitations in combination with the vote counting findings. Third, much of the data was from cross-sectional studies, limiting causal inference. This is particularly relevant for studies assessing contextual exposures, where observational studies may suffer from amongst others selection bias (i.e., an individual’s choice to reside in a certain area is related to the study outcome). Finally, almost all studies were conducted in high-income countries. This limits the ability to generalize the results to low-income countries. We were also unable to stratify study results by age group. It is thinkable that older people spend more time in their neighborhood than younger, employed people and older people more often experience feelings of social isolation/loneliness than young and middle-aged adults (Luhmann & Hawkley, 2016).

The main strength of this review is its broad scope. We employed a thorough and broad search across three large databases, assisted by an information specialist. In this way, we were able to capture all available relevant evidence and provide a comprehensive overview of the existing literature on this topic. In addition, we performed a backward reference check to complement this thorough search. Moreover, following the call for more consistency in the social environment literature (Kepper et al., 2019), we combined all social environment factors that were categorized into the same dimension for the purposes of synthesis of findings.

### Implications for practice, policy and research

This review focusing on the general adult population suggests that unfavorable social environments are associated with higher cardiometabolic risk. Other reviews have demonstrated the importance of favorable social environments for the treatment and management of CMD. For instance, Schram et al. showed that smaller network size and less social support was associated with increased risk of diabetes complications (Schram et al., 2021), and Spencer-Bonilla et al. found promising tentative effects of social network interventions on glycemic control and quality of life in T2DM patients (Spencer-Bonilla, Ponce, Rodrigeuz-Gutierrez, et al., 2017). It is therefore no surprise that there is increasing attention for the Social Determinants of Health (World Health Organization) - including social environmental aspects - among healthcare providers. In response to this development, White-Williams et al. suggest a number of conceptual models and screening tools that healthcare providers could use to consider the role of social determinants of health in the treatment of patients with heart failure (White-Williams et al., 2020). One of the best practices considered is having a list of availability community resources available in the healthcare clinic (Davidson & McGinn, 2019).

Given the evidence for links between the social environment and other health outcomes such as frailty (Duppen et al., 2019), mental health (Bjorlyhaug, Karsson, Kim Hesook, & Kleppe, 2021; Breedvelt et al., 2022), and general health (Ehsan et al., 2019; Pérez et al., 2020), the importance of the social environment goes beyond its effects on cardiometabolic health. Other studies have shown the impact of social isolation and loneliness on CVD risk through chronic stress (Leigh-Hunt et al., 2017; Rentscher et al., 2022; Xia & Li, 2018). The adverse effects of social isolation and societal polarization on health should therefore not be underestimated and taken into account in deliberations around policies such as Covid-19 restrictions (Lippke et al., 2021).

Yet, how exactly social relations may protect against CMD remains to be explored as this review showed inconsistent results between the dimension “Social Relationships and Norms” and cardiometabolic risk factors. This requires a better conceptualization and measurement of the aspects of social relationships that may be beneficial or harmful to health. The inconsistent terminology and conceptualization of the dimensions of the social environment has also been highlighted by other authors (Barnett & Casper, 2001; Braveman & Gottlieb, 2014; Kepper et al., 2019). It may also be important to specifically consider sex effects of social relationships, given the findings in this review. Another aspect to consider in future research is the dilution of the effects of offline social relationships by online relationships. Indeed, studies into the effects of the online social environment on CMD risk are rare but should be the topic of future research given the increasing use of social media and other online platforms that allow for interpersonal interactions.

While we aimed to capture the totality of social environment exposures and their association with CMD risk factors, most studies only studied a single aspect of the social environment in a cross-sectional setting or with modest follow-up at most. This hampers the estimation of the total “life course” (Ben-Shlomo et al., 2014) or “exposome” (Beulens et al., 2022) influence of the social environment on CMD risk and future studies would benefit from the integration of multiple dimensions of the social environment over the life course, and their combined effects on CMD risk.

Finally, referring back to the conceptual framework we used as the basis for this systematic review (Abreu et al. submitted elsewhere), future studies should assess the extent to which dimensions of the social environment explain the impact of structural socioeconomic factors on health outcomes. Indeed, there is a very strong link between poverty and ill health (Marmot et al., 2020) and part of this association may be explained by the adverse social environments individuals become exposed to when living in poverty. For example, in a cross-European study, we demonstrated that neighborhood-level social capital explained large parts of the association between neighborhood income inequality and BMI (Mackenbach et al., 2016-a). Regardless of the extent to which the links between structural socioeconomic factors and CMD is explained by the dimensions of the social environment, it is likely that both of those aspects should be targeted through upstream policies to improve population level cardiometabolic health.

In conclusion, the findings from vote counting and meta-analyses suggest that exposure to adverse social environments is associated with unfavorable cardiometabolic risk factors profiles. The evidence for the dimension “Economic and Social Disadvantage” is most robust while other dimensions of the social environment such as “Civic Engagement and Participation” require more evidence from well-designed prospective studies.

## Funding

This work was supported by EXPOSOME-NL and EXPANSE. EXPOSOME-NL is funded through the Gravitation program of the Dutch Ministry of Education, Culture, and Science and the Netherlands Organization for Scientific Research (10.13039/501100003246NWO grant number 024.004.017). EXPANSE received funding from the European Union’s 10.13039/501100007601Horizon 2020 research and innovation program under grant agreement number 874627. The funders had no role in study design, data collection and analysis, decision to publish, or preparation of the manuscript.

## Declaration of competing interest

The authors declare that they have no known competing financial interests or personal relationships that could have appeared to influence the work reported in this paper.

## Data Availability

Data sharing not applicable (review article)

## Appendix A Supplementary data

Multimedia component 1 Multimedia component 1

Multimedia component 2 **Supplementary File 3.** Quality assessment of cohort studies, based on NOS. **Supplementary File 4.** Quality assessment of cross-sectional studies, based on NOS. Multimedia component 2

Multimedia component 6 Multimedia component 6

Multimedia component 7 **Supplementary File 2.** Conceptual framework on social environment and cardiometabolic health. Multimedia component 7

Multimedia component 8 **Supplementary File 1.** Full search strategy. Multimedia component 8
