# Supplementary material for: Associations between dimensions of the social environment and cardiometabolic risk factors: Systematic review and meta-analysis
Source: SSM Popul Health. 2023 Nov 25;25:101559. doi: 10.1016/j.ssmph.2023.101559 (PMC10749911; doi:10.1016/j.ssmph.2023.101559)
Supplement: Multimedia component 2 — Supplementary File 3. Quality assessment of cohort studies, based on NOS. Supplementary File 4. Quality assessment of cross-sectional studies, based on NOS. [file mmc2.docx]

| **Supplementary File 3.** Quality assessment of longitudinal / cohort studies studies, based on NOS | | | | | | | | | |  | |  | |  |
| --- | --- | --- | --- | --- | --- | --- | --- | --- | --- | --- | --- | --- | --- | --- |
|  |  |  |  | **Selection** |  |  |  | **Comparability** | **Outcome** | |  | |  | |
| **Reference** | **First Author** | **Total score (max. 9)** | **%** | Representativeness of the exposed cohort (max. 1 point) | Selection of the non-exposed cohort (max. 1 point) | Ascertainment of exposure (max. 1 point) | Demonstration that outcome of interest was not present at start of study (max. 1 point) | Comparability of cohorts on the basis of the design or analysis (max. 2 points) | Assessment of outcome (max. 1 point) | | Was follow-up long enough for outcomes to occur (max. 1 point) | | Adequacy of follow up of cohorts (max. 1 point) | |
| [1] | Adjaye-Gbewonyo, 2018 | 7 | 78% | 1 | 1 | 1 | 1 | 2 | 1 | | 0 | | 0 | |
| [2] | Andell, 2020 | 9 | 100% | 1 | 1 | 1 | 1 | 2 | 1 | | 1 | | 1 | |
| [3] | Boylan, 2017 | 6 | 67% | 0 | 1 | 1 | 0 | 2 | 1 | | 1 | | 0 | |
| [4] | Browning, 2012 | 8 | 89% | 1 | 1 | 1 | 0 | 2 | 1 | | 1 | | 1 | |
| [5] | Carson, 2007 | 7 | 78% | 0 | 1 | 1 | 0 | 2 | 1 | | 1 | | 1 | |
| [6] | Caspi, 2006 | 6 | 67% | 0 | 1 | 1 | 0 | 1 | 1 | | 1 | | 1 | |
| [7] | Claudel, 2018 | 8 | 89% | 0 | 1 | 1 | 1 | 2 | 1 | | 1 | | 1 | |
| [8] | Diez Roux, 2002 | 8 | 89% | 0 | 1 | 1 | 1 | 2 | 1 | | 1 | | 1 | |
| [9] | Garcia, 2016 | 6 | 67% | 0 | 1 | 1 | 1 | 1 | 1 | | 1 | | 0 | |
| [10] | Gary-Webb, 2020 | 6 | 67% | 0 | 1 | 1 | 0 | 2 | 1 | | 1 | | 0 | |
| [11] | Halonen, 2015 | 6 | 67% | 0 | 1 | 1 | 0 | 1 | 1 | | 1 | | 1 | |
| [12] | Hamad, 2020 | 7 | 78% | 0 | 1 | 1 | 1 | 1 | 1 | | 1 | | 1 | |
| [13] | Hilding, 2015 | 8 | 89% | 0 | 1 | 1 | 1 | 2 | 1 | | 1 | | 1 | |
| [14] | Jimenez, 2019 | 7 | 78% | 0 | 1 | 1 | 1 | 2 | 1 | | 1 | | 0 | |
| [15] | Kakinami, 2017 | 6 | 67% | 1 | 1 | 1 | 0 | 1 | 0 | | 1 | | 1 | |
| [16] | Kershaw, 2017 | 7 | 78% | 0 | 1 | 1 | 0 | 2 | 1 | | 1 | | 1 | |
| [17] | Kim, 2010 | 7 | 78% | 0 | 1 | 1 | 0 | 2 | 1 | | 1 | | 1 | |
| [18] | Kivimaki, 2018 | 8 | 89% | 1 | 1 | 1 | 0 | 2 | 1 | | 1 | | 1 | |
| [19] | Lei, 2018 | 7 | 78% | 0 | 1 | 1 | 0 | 2 | 1 | | 1 | | 1 | |
| [20] | Lemelin, 2009 | 8 | 89% | 1 | 1 | 1 | 0 | 2 | 1 | | 1 | | 1 | |
| [21] | Lippert, 2017 | 7 | 78% | 1 | 1 | 1 | 0 | 2 | 1 | | 1 | | 0 | |
| [22] | Marley, 2015 | 8 | 89% | 1 | 1 | 1 | 0 | 2 | 1 | | 1 | | 1 | |
| [23] | Martin, 2019 | 5 | 56% | 0 | 1 | 1 | 0 | 2 | 0 | | 1 | | 0 | |
| [24] | Mayne, 2018 | 7 | 78% | 0 | 1 | 1 | 1 | 2 | 1 | | 1 | | 0 | |
| [25] | Mayne, 2019 | 8 | 89% | 1 | 1 | 1 | 1 | 2 | 0 | | 1 | | 1 | |
| [26] | Merkin, 2020 | 8 | 89% | 1 | 1 | 1 | 1 | 2 | 0 | | 1 | | 1 | |
| [27] | Murray, 2010 | 8 | 89% | 1 | 1 | 1 | 0 | 2 | 1 | | 1 | | 1 | |
| [28] | Nazmi, 2010 | 8 | 89% | 1 | 1 | 1 | 1 | 2 | 1 | | 1 | | 0 | |
| [29] | Ngo, 2013 | 9 | 100% | 1 | 1 | 1 | 1 | 2 | 1 | | 1 | | 1 | |
| [30] | Ngo, 2014 | 8 | 89% | 1 | 1 | 1 | 1 | 2 | 0 | | 1 | | 1 | |
| [31] | Nikulina, 2014 | 7 | 78% | 0 | 1 | 1 | 0 | 2 | 1 | | 1 | | 1 | |
| [32] | Pedersen, 2016 | 8 | 89% | 1 | 1 | 1 | 1 | 2 | 1 | | 1 | | 0 | |
| [33] | Pollitt, 2007 | 6 | 67% | 0 | 1 | 1 | 0 | 1 | 1 | | 1 | | 1 | |
| [34] | Pollitt, 2008 | 6 | 67% | 0 | 1 | 1 | 0 | 1 | 1 | | 1 | | 1 | |
| [35] | Sorman, 2016 | 7 | 78% | 1 | 1 | 1 | 0 | 2 | 1 | | 1 | | 0 | |
| [36] | Tung, 2019 | 8 | 89% | 0 | 1 | 1 | 1 | 2 | 1 | | 1 | | 1 | |
| [37] | Williams, 2012 | 9 | 100% | 1 | 1 | 1 | 1 | 2 | 1 | | 1 | | 1 | |
| [38] | Wing, 2016 | 7 | 78% | 1 | 1 | 1 | 1 | 1 | 1 | | 1 | | 0 | |
| [39] | Yang, 2013 | 6 | 67% | 1 | 1 | 1 | 0 | 2 | 0 | | 1 | | 0 | |
| [40] | Yang, 2014 | 6 | 67% | 0 | 1 | 0 | 1 | 2 | 1 | | 1 | | 0 | |
| [41] | Yang, 2015 | 9 | 100% | 1 | 1 | 1 | 1 | 2 | 1 | | 1 | | 1 | |
| [42] | Yang, 2016 | 5 | 56% | 1 | 1 | 0 | 0 | 2 | 0 | | 1 | | 0 | |
| [43] | Yao, 2019 | 7 | 78% | 1 | 1 | 0 | 1 | 2 | 1 | | 1 | | 0 | |
| [44] | Zoller, 2013 | 9 | 100% | 1 | 1 | 1 | 1 | 2 | 1 | | 1 | | 1 | |

| **Supplementary File 4.** Quality assessment of cross-sectional studies, based on NOS | | | | | | |  | |  |  | |  | |  | |  |
| --- | --- | --- | --- | --- | --- | --- | --- | --- | --- | --- | --- | --- | --- | --- | --- | --- |
|  |  |  |  | **Selection** |  |  | |  | | | **Comparability** | | **Outcome** | |  | |
| **Reference** | **Author, year** | **Total score (max. 10)** | **%** | Representativeness of the sample (max. 1 point) | Sample size (max. 1 point) | Non-respondents (max. 1 point) | | Ascertainment of the exposure (max. 2 points) | | | The subjects in different outcome groups are comparable, based on the study design or analysis. Confounding factors are controlled. (max. 2 point) | | Assessment of the outcome (max. 2 points) | | Statistical test (max. 1 point) | |
| [45] | Adams, 2009 | 7 | 70% | 1 | 0 | 0 | | 1 | | | 2 | | 2 | | 1 | |
| [46] | Agyemang, 2007 | 7 | 70% | 1 | 0 | 0 | | 1 | | | 2 | | 2 | | 1 | |
| [47] | Aliarzadeh, 2014 | 7 | 70% | 1 | 0 | 0 | | 2 | | | 1 | | 2 | | 1 | |
| [48] | Altevers, 2016 | 5 | 50% | 1 | 0 | 0 | | 1 | | | 0 | | 2 | | 1 | |
| [49] | Andersen, 2008 | 6 | 60% | 1 | 0 | 0 | | 1 | | | 2 | | 2 | | 0 | |
| [50] | Auchincloss, 2007 | 6 | 60% | 1 | 0 | 0 | | 1 | | | 2 | | 2 | | 0 | |
| [51] | Bagheri, 2015 | 3 | 30% | 0 | 0 | 0 | | 1 | | | 0 | | 2 | | 0 | |
| [52] | Bagheri, 2019 | 3 | 30% | 1 | 0 | 0 | | 0 | | | 0 | | 2 | | 0 | |
| [53] | Bajaj, 2016 | 6 | 60% | 0 | 0 | 0 | | 1 | | | 2 | | 2 | | 1 | |
| [54] | Baldock, 2012 | 7 | 70% | 1 | 0 | 0 | | 1 | | | 2 | | 2 | | 1 | |
| [55] | Baldock, 2018 | 7 | 70% | 1 | 0 | 0 | | 1 | | | 2 | | 2 | | 1 | |
| [56] | Barber, 2016 | 6 | 60% | 0 | 0 | 0 | | 1 | | | 2 | | 2 | | 1 | |
| [57] | Barber, 2016 | 3 | 30% | 0 | 0 | 0 | | 1 | | | 0 | | 2 | | 0 | |
| [58] | Barber, 2016 | 6 | 60% | 0 | 0 | 0 | | 1 | | | 2 | | 2 | | 1 | |
| [59] | Bhopal, 2002 | 1 | 10% | 0 | 0 | 0 | | 1 | | | 0 | | 0 | | 0 | |
| [60] | Bird, 2010 | 7 | 70% | 1 | 0 | 0 | | 1 | | | 2 | | 2 | | 1 | |
| [61] | Bland, 1991 | 6 | 60% | 0 | 0 | 0 | | 1 | | | 2 | | 2 | | 1 | |
| [62] | Bland, 2000 | 5 | 50% | 0 | 0 | 0 | | 1 | | | 2 | | 2 | | 0 | |
| [63] | Breckenkamp, 2007 | 7 | 70% | 1 | 0 | 0 | | 1 | | | 2 | | 2 | | 1 | |
| [64] | Bu, 2021 | 7 | 70% | 1 | 0 | 0 | | 1 | | | 2 | | 2 | | 1 | |
| [65] | Carels, 1998 | 4 | 40% | 0 | 0 | 0 | | 1 | | | 0 | | 2 | | 1 | |
| [66] | Cathorall, 2015 | 5 | 50% | 0 | 0 | 0 | | 1 | | | 1 | | 2 | | 1 | |
| [67] | Chaix, 2008 | 6 | 60% | 0 | 0 | 0 | | 1 | | | 2 | | 2 | | 1 | |
| [68] | Chaix, 2010 | 6 | 60% | 0 | 0 | 0 | | 1 | | | 2 | | 2 | | 1 | |
| [69] | Chaparro, 2018 | 6 | 60% | 0 | 0 | 0 | | 1 | | | 2 | | 2 | | 1 | |
| [70] | Chichlowska, 2008 | 6 | 60% | 0 | 0 | 0 | | 1 | | | 2 | | 2 | | 1 | |
| [71] | Clark, 2012 | 6 | 60% | 0 | 0 | 0 | | 1 | | | 2 | | 2 | | 1 | |
| [72] | Clark, 2013 | 6 | 60% | 0 | 0 | 0 | | 1 | | | 2 | | 2 | | 1 | |
| [73] | Climie, 2019 | 6 | 60% | 0 | 0 | 0 | | 1 | | | 2 | | 2 | | 1 | |
| [74] | Cohn, 2017 | 5 | 50% | 0 | 0 | 0 | | 1 | | | 1 | | 2 | | 1 | |
| [75] | Coulon, 2016 | 5 | 50% | 0 | 0 | 0 | | 1 | | | 1 | | 2 | | 1 | |
| [76] | Coulon, 2016 | 6 | 60% | 0 | 0 | 0 | | 1 | | | 2 | | 2 | | 1 | |
| [77] | Cozier, 2016 | 6 | 60% | 0 | 0 | 0 | | 1 | | | 2 | | 2 | | 1 | |
| [78] | Creaven, 2013 | 5 | 50% | 0 | 0 | 0 | | 1 | | | 1 | | 2 | | 1 | |
| [79] | Cross, 2019 | 6 | 60% | 1 | 0 | 0 | | 1 | | | 1 | | 2 | | 1 | |
| [80] | Cubbin, 2005 | 4 | 40% | 0 | 0 | 0 | | 1 | | | 2 | | 0 | | 1 | |
| [81] | De Moraes, 2019 | 6 | 60% | 0 | 0 | 0 | | 1 | | | 2 | | 2 | | 1 | |
| [82] | Deans, 2009 | 6 | 60% | 0 | 1 | 0 | | 1 | | | 2 | | 2 | | 0 | |
| [83] | Diez Roux, 2002 | 4 | 40% | 0 | 0 | 0 | | 1 | | | 1 | | 2 | | 0 | |
| [84] | Diez-Roux, 1997 | 6 | 60% | 0 | 0 | 0 | | 1 | | | 2 | | 2 | | 1 | |
| [85] | Djekic, 2018 | 4 | 40% | 1 | 0 | 0 | | 0 | | | 1 | | 2 | | 0 | |
| [86] | Do, 2011 | 6 | 60% | 1 | 0 | 0 | | 1 | | | 2 | | 2 | | 0 | |
| [87] | Dragano, 2009 | 7 | 70% | 1 | 0 | 0 | | 1 | | | 2 | | 2 | | 1 | |
| [88] | Dubowitz, 2012 | 6 | 60% | 0 | 0 | 0 | | 1 | | | 2 | | 2 | | 1 | |
| [89] | Duncan, 2016 | 6 | 60% | 0 | 0 | 0 | | 1 | | | 2 | | 2 | | 1 | |
| [90] | Dwane, 2020 | 5 | 50% | 0 | 0 | 0 | | 1 | | | 2 | | 1 | | 1 | |
| [91] | Eichinger, 2015 | 5 | 50% | 0 | 0 | 0 | | 1 | | | 2 | | 1 | | 1 | |
| [92] | Ellaway, 2007 | 6 | 60% | 0 | 0 | 0 | | 1 | | | 2 | | 2 | | 1 | |
| [93] | Engstrom, 2001 | 4 | 40% | 0 | 0 | 0 | | 1 | | | 1 | | 1 | | 1 | |
| [94] | Ferguson, 2020 | 8 | 80% | 1 | 1 | 1 | | 0 | | | 2 | | 2 | | 1 | |
| [95] | Finch, 2010 | 7 | 70% | 1 | 0 | 0 | | 1 | | | 2 | | 2 | | 1 | |
| [96] | Foraker, 2019 | 4 | 40% | 0 | 0 | 0 | | 1 | | | 2 | | 0 | | 1 | |
| [97] | Ford, 2006 | 8 | 80% | 1 | 0 | 0 | | 2 | | | 2 | | 2 | | 1 | |
| [98] | Ford, 2019 | 8 | 80% | 0 | 0 | 1 | | 2 | | | 2 | | 2 | | 1 | |
| [99] | Fuller, 2018 | 5 | 50% | 0 | 0 | 0 | | 1 | | | 1 | | 2 | | 1 | |
| [100] | Gallo, 2012 | 7 | 70% | 0 | 0 | 1 | | 1 | | | 2 | | 2 | | 1 | |
| [101] | Garcia, 2015 | 7 | 70% | 0 | 0 | 0 | | 2 | | | 2 | | 2 | | 1 | |
| [102] | Gebreab, 2015 | 6 | 60% | 1 | 0 | 0 | | 1 | | | 2 | | 1 | | 1 | |
| [103] | Grimaud, 2013 | 5 | 50% | 0 | 0 | 0 | | 1 | | | 2 | | 2 | | 0 | |
| [104] | Hanson, 1988 | 7 | 70% | 1 | 0 | 1 | | 0 | | | 2 | | 2 | | 1 | |
| [105] | Helminen, 1995 | 3 | 30% | 1 | 0 | 1 | | 1 | | | 0 | | 0 | | 0 | |
| [106] | Helminen, 1995 | 8 | 80% | 1 | 0 | 0 | | 2 | | | 2 | | 2 | | 1 | |
| [107] | Helminen, 1997 | 7 | 70% | 1 | 0 | 0 | | 1 | | | 2 | | 2 | | 1 | |
| [108] | Henning, 2014 | 4 | 40% | 0 | 0 | 0 | | 1 | | | 0 | | 2 | | 1 | |
| [109] | Hickson, 2011 | 5 | 50% | 0 | 0 | 0 | | 1 | | | 1 | | 2 | | 1 | |
| [110] | Hofelmann, 2012 | 9 | 90% | 1 | 1 | 1 | | 1 | | | 2 | | 2 | | 1 | |
| [111] | Holmes, 2012 | 6 | 60% | 0 | 0 | 0 | | 1 | | | 2 | | 2 | | 1 | |
| [112] | Horsten, 1999 | 7 | 70% | 1 | 0 | 0 | | 1 | | | 2 | | 2 | | 1 | |
| [113] | Hosseini, 2020 | 7 | 70% | 1 | 0 | 0 | | 1 | | | 2 | | 2 | | 1 | |
| [114] | Hughes, 2009 | 6 | 60% | 0 | 0 | 0 | | 2 | | | 1 | | 2 | | 1 | |
| [115] | Islam, 2020 | 4 | 40% | 0 | 0 | 0 | | 1 | | | 2 | | 0 | | 1 | |
| [116] | Keita, 2014 | 5 | 50% | 0 | 0 | 0 | | 1 | | | 2 | | 2 | | 0 | |
| [117] | Kelli, 2017 | 5 | 50% | 0 | 0 | 0 | | 1 | | | 2 | | 2 | | 0 | |
| [118] | Kent de Grey, 2019 | 7 | 70% | 0 | 1 | 0 | | 2 | | | 1 | | 2 | | 1 | |
| [119] | Kim, 2020 | 5 | 50% | 0 | 0 | 0 | | 1 | | | 2 | | 1 | | 1 | |
| [120] | King, 2011 | 7 | 70% | 1 | 0 | 1 | | 1 | | | 2 | | 1 | | 1 | |
| [121] | Lawlor, 2005 | 2 | 20% | 1 | 0 | 0 | | 1 | | | 0 | | 0 | | 0 | |
| [122] | Lee, 2020 | 8 | 80% | 1 | 0 | 1 | | 1 | | | 2 | | 2 | | 1 | |
| [123] | Lewis, 2010 | 6 | 60% | 0 | 0 | 0 | | 1 | | | 2 | | 2 | | 1 | |
| [124] | Li, 2017 | 5 | 50% | 0 | 0 | 0 | | 1 | | | 2 | | 1 | | 1 | |
| [125] | Li, 2019 | 5 | 50% | 1 | 0 | 0 | | 1 | | | 2 | | 0 | | 1 | |
| [126] | Linden, 1993 | 5 | 50% | 0 | 0 | 0 | | 1 | | | 1 | | 2 | | 1 | |
| [127] | Loose, 2017 | 6 | 60% | 0 | 0 | 1 | | 1 | | | 1 | | 2 | | 1 | |
| [128] | Loucks, 2005 | 6 | 60% | 0 | 0 | 0 | | 1 | | | 2 | | 2 | | 1 | |
| [129] | Loucks, 2006 | 6 | 60% | 0 | 0 | 0 | | 1 | | | 2 | | 2 | | 1 | |
| [130] | Loucks, 2006 | 6 | 60% | 0 | 0 | 0 | | 1 | | | 2 | | 2 | | 1 | |
| [131] | Maki, 2020 | 6 | 60% | 1 | 0 | 0 | | 1 | | | 2 | | 2 | | 0 | |
| [132] | Matricciani, 2013 | 7 | 70% | 1 | 0 | 0 | | 1 | | | 2 | | 2 | | 1 | |
| [133] | McKenzie, 2020 | 6 | 60% | 0 | 1 | 0 | | 1 | | | 2 | | 2 | | 0 | |
| [134] | Mellman, 2015 | 4 | 40% | 0 | 0 | 0 | | 1 | | | 1 | | 2 | | 0 | |
| [135] | Merkin, 2009 | 5 | 50% | 1 | 0 | 0 | | 1 | | | 2 | | 0 | | 1 | |
| [136] | Merlo, 2001 | 7 | 70% | 1 | 0 | 0 | | 1 | | | 2 | | 2 | | 1 | |
| [137] | Metcalf, 2008 | 4 | 40% | 1 | 0 | 0 | | 0 | | | 1 | | 2 | | 0 | |
| [138] | Meza, 2020 | 6 | 60% | 0 | 0 | 0 | | 1 | | | 2 | | 2 | | 1 | |
| [139] | Mobley, 2006 | 6 | 60% | 0 | 0 | 0 | | 1 | | | 2 | | 2 | | 1 | |
| [140] | Murakami, 2010 | 2 | 20% | 0 | 0 | 0 | | 1 | | | 1 | | 0 | | 0 | |
| [141] | Naimi, 2009 | 7 | 70% | 1 | 0 | 0 | | 1 | | | 2 | | 2 | | 1 | |
| [142] | Neergheen, 2019 | 8 | 80% | 1 | 0 | 0 | | 2 | | | 2 | | 2 | | 1 | |
| [143] | Nordstrom, 2004 | 5 | 50% | 1 | 0 | 0 | | 1 | | | 2 | | 0 | | 1 | |
| [144] | Petersen, 2008 | 7 | 70% | 1 | 0 | 0 | | 1 | | | 2 | | 2 | | 1 | |
| [145] | Piferi, 2006 | 4 | 40% | 0 | 0 | 0 | | 1 | | | 0 | | 2 | | 1 | |
| [146] | Pollack, 2012 | 6 | 60% | 1 | 0 | 1 | | 1 | | | 2 | | 0 | | 1 | |
| [147] | Pollard, 2003 | 5 | 50% | 0 | 0 | 0 | | 1 | | | 1 | | 2 | | 1 | |
| [148] | Ribeiro, 2019 | 5 | 50% | 0 | 0 | 0 | | 1 | | | 2 | | 2 | | 0 | |
| [149] | Riva, 2016 | 7 | 70% | 1 | 0 | 0 | | 1 | | | 2 | | 2 | | 1 | |
| [150] | Robinette, 2016 | 5 | 50% | 0 | 0 | 0 | | 1 | | | 2 | | 2 | | 0 | |
| [151] | Robinette, 2020 | 6 | 60% | 0 | 0 | 0 | | 1 | | | 2 | | 2 | | 1 | |
| [152] | Rosvall, 2007 | 6 | 60% | 1 | 0 | 0 | | 1 | | | 2 | | 2 | | 0 | |
| [153] | Samuel, 2015 | 6 | 60% | 0 | 0 | 0 | | 1 | | | 2 | | 2 | | 1 | |
| [154] | Schulz, 2013 | 7 | 70% | 1 | 0 | 0 | | 1 | | | 2 | | 2 | | 1 | |
| [155] | Seeman, 2014 | 8 | 80% | 1 | 0 | 1 | | 1 | | | 2 | | 2 | | 1 | |
| [156] | Smith, 1998 | 6 | 60% | 1 | 0 | 0 | | 1 | | | 2 | | 2 | | 0 | |
| [157] | Sprung, 2019 | 6 | 60% | 1 | 0 | 0 | | 1 | | | 2 | | 2 | | 0 | |
| [158] | Steppuhn, 2019 | 7 | 70% | 1 | 0 | 0 | | 1 | | | 2 | | 2 | | 1 | |
| [159] | Strogatz, 1997 | 6 | 60% | 0 | 0 | 1 | | 1 | | | 1 | | 2 | | 1 | |
| [160] | Theorell, 1982 | 3 | 30% | 0 | 0 | 1 | | 0 | | | 0 | | 2 | | 0 | |
| [161] | Toms, 2020 | 6 | 60% | 0 | 0 | 1 | | 1 | | | 1 | | 2 | | 1 | |
| [162] | Troxel, 2010 | 7 | 70% | 1 | 0 | 0 | | 1 | | | 2 | | 2 | | 1 | |
| [163] | Uchino, 2013 | 6 | 60% | 0 | 0 | 0 | | 1 | | | 2 | | 2 | | 1 | |
| [164] | Unger, 2014 | 6 | 60% | 1 | 0 | 0 | | 1 | | | 2 | | 2 | | 0 | |
| [165] | Wagner, 2016 | 9 | 90% | 1 | 1 | 1 | | 1 | | | 2 | | 2 | | 1 | |
| [166] | Whittaker, 2012 | 1 | 10% | 0 | 0 | 0 | | 0 | | | 0 | | 0 | | 1 | |
| [167] | Willets, 2019 | 6 | 60% | 0 | 0 | 0 | | 1 | | | 2 | | 2 | | 1 | |
| [168] | Zanelatto, 2019 | 9 | 90% | 1 | 1 | 1 | | 1 | | | 2 | | 2 | | 1 | |

1. Adjaye-Gbewonyo, K., et al., *Income inequality and cardiovascular disease risk factors in a highly unequal country: a fixed-effects analysis from South Africa.* Int J Equity Health, 2018. **17**(1): p. 31.

2. Andell, P., et al., *Neighborhood socioeconomic status and aortic stenosis: A Swedish study based on nationwide registries and an echocardiographic screening cohort.* Int J Cardiol, 2020. **318**: p. 153-159.

3. Boylan, J.M. and S.A. Robert, *Neighborhood SES is particularly important to the cardiovascular health of low SES individuals.* Soc Sci Med, 2017. **188**: p. 60-68.

4. Browning, C.R., K.A. Cagney, and J. Iveniuk, *Neighborhood stressors and cardiovascular health: crime and C-reactive protein in Dallas, USA.* Soc Sci Med, 2012. **75**(7): p. 1271-9.

5. Carson, A.P., et al., *Cumulative socioeconomic status across the life course and subclinical atherosclerosis.* Ann Epidemiol, 2007. **17**(4): p. 296-303.

6. Caspi, A., et al., *Socially isolated children 20 years later: risk of cardiovascular disease.* Arch Pediatr Adolesc Med, 2006. **160**(8): p. 805-11.

7. Claudel, S.E., et al., *Association between neighborhood-level socioeconomic deprivation and incident hypertension: A longitudinal analysis of data from the Dallas heart study.* Am Heart J, 2018. **204**: p. 109-118.

8. Diez Roux, A.V., et al., *Socioeconomic disadvantage and change in blood pressure associated with aging.* Circulation, 2002. **106**(6): p. 703-10.

9. Garcia, L., et al., *Influence of neighbourhood socioeconomic position on the transition to type II diabetes in older Mexican Americans: the Sacramento Area Longitudinal Study on Aging.* BMJ Open, 2016. **6**(8): p. e010905.

10. Gary-Webb, T.L., et al., *Changes in perceptions of neighborhood environment and Cardiometabolic outcomes in two predominantly African American neighborhoods.* BMC Public Health, 2020. **20**(1): p. 52.

11. Halonen, J.I., et al., *Childhood Psychosocial Adversity and Adult Neighborhood Disadvantage as Predictors of Cardiovascular Disease: A Cohort Study.* Circulation, 2015. **132**(5): p. 371-9.

12. Hamad, R., et al., *Association of Neighborhood Disadvantage With Cardiovascular Risk Factors and Events Among Refugees in Denmark.* JAMA Netw Open, 2020. **3**(8): p. e2014196.

13. Hilding, A., C. Shen, and C.G. Östenson, *Social network and development of prediabetes and type 2 diabetes in middle-aged Swedish women and men.* Diabetes Res Clin Pract, 2015. **107**(1): p. 166-77.

14. Jimenez, M.P., et al., *Longitudinal associations of neighborhood socioeconomic status with cardiovascular risk factors: A 46-year follow-up study.* Soc Sci Med, 2019. **241**: p. 112574.

15. Kakinami, L., et al., *Neighbourhood disadvantage and behavioural problems during childhood and the risk of cardiovascular disease risk factors and events from a prospective cohort.* Prev Med Rep, 2017. **8**: p. 294-300.

16. Kershaw, K.N., et al., *Association of Changes in Neighborhood-Level Racial Residential Segregation With Changes in Blood Pressure Among Black Adults: The CARDIA Study.* JAMA Intern Med, 2017. **177**(7): p. 996-1002.

17. Kim, D., et al., *Do neighborhood socioeconomic deprivation and low social cohesion predict coronary calcification?: the CARDIA study.* Am J Epidemiol, 2010. **172**(3): p. 288-98.

18. Kivimäki, M., et al., *Neighbourhood socioeconomic disadvantage, risk factors, and diabetes from childhood to middle age in the Young Finns Study: a cohort study.* Lancet Public Health, 2018. **3**(8): p. e365-e373.

19. Lei, M.K., S.R.H. Beach, and R.L. Simons, *Biological embedding of neighborhood disadvantage and collective efficacy: Influences on chronic illness via accelerated cardiometabolic age.* Dev Psychopathol, 2018. **30**(5): p. 1797-1815.

20. Lemelin, E.T., et al., *Life-course socioeconomic positions and subclinical atherosclerosis in the multi-ethnic study of atherosclerosis.* Soc Sci Med, 2009. **68**(3): p. 444-51.

21. Lippert, A.M., et al., *Associations of Continuity and Change in Early Neighborhood Poverty With Adult Cardiometabolic Biomarkers in the United States: Results From the National Longitudinal Study of Adolescent to Adult Health, 1995-2008.* Am J Epidemiol, 2017. **185**(9): p. 765-776.

22. Marley, T.L. and M.W. Metzger, *A longitudinal study of structural risk factors for obesity and diabetes among American Indian young adults, 1994-2008.* Prev Chronic Dis, 2015. **12**: p. E69.

23. Martin, C.L., et al., *Neighborhood disadvantage across the transition from adolescence to adulthood and risk of metabolic syndrome.* Health Place, 2019. **57**: p. 131-138.

24. Mayne, S.L., et al., *Longitudinal Associations of Neighborhood Crime and Perceived Safety With Blood Pressure: The Multi-Ethnic Study of Atherosclerosis (MESA).* Am J Hypertens, 2018. **31**(9): p. 1024-1032.

25. Mayne, S.L., et al., *Neighbourhood racial/ethnic residential segregation and cardiometabolic risk: the multiethnic study of atherosclerosis.* J Epidemiol Community Health, 2019. **73**(1): p. 26-33.

26. Merkin, S.S., et al., *Race/ethnicity, neighborhood socioeconomic status and cardio-metabolic risk.* SSM Popul Health, 2020. **11**: p. 100634.

27. Murray, E.T., et al., *Trajectories of neighborhood poverty and associations with subclinical atherosclerosis and associated risk factors: the multi-ethnic study of atherosclerosis.* Am J Epidemiol, 2010. **171**(10): p. 1099-108.

28. Nazmi, A., et al., *Cross-sectional and longitudinal associations of neighborhood characteristics with inflammatory markers: findings from the multi-ethnic study of atherosclerosis.* Health Place, 2010. **16**(6): p. 1104-12.

29. Ngo, A.D., et al., *Area-level socioeconomic characteristics and incidence of metabolic syndrome: a prospective cohort study.* BMC Public Health, 2013. **13**: p. 681.

30. Ngo, A.D., et al., *Area-level socioeconomic characteristics, prevalence and trajectories of cardiometabolic risk.* Int J Environ Res Public Health, 2014. **11**(1): p. 830-48.

31. Nikulina, V. and C.S. Widom, *Do race, neglect, and childhood poverty predict physical health in adulthood? A multilevel prospective analysis.* Child Abuse Negl, 2014. **38**(3): p. 414-24.

32. Pedersen, J.M., et al., *Psychosocial risk factors for the metabolic syndrome: A prospective cohort study.* Int J Cardiol, 2016. **215**: p. 41-6.

33. Pollitt, R.A., et al., *Early-life and adult socioeconomic status and inflammatory risk markers in adulthood.* Eur J Epidemiol, 2007. **22**(1): p. 55-66.

34. Pollitt, R.A., et al., *Cumulative life course and adult socioeconomic status and markers of inflammation in adulthood.* J Epidemiol Community Health, 2008. **62**(6): p. 484-91.

35. Sörman, D.E., P. Hansson, and M. Rönnlund, *Blood pressure levels and longitudinal changes in relation to social network factors.* Psihologijske Teme, 2016. **25**(1): p. 59-73.

36. Tung, E.L., et al., *Association of Rising Violent Crime With Blood Pressure and Cardiovascular Risk: Longitudinal Evidence From Chicago, 2014-2016.* Am J Hypertens, 2019. **32**(12): p. 1192-1198.

37. Williams, E.D., et al., *Area-level socioeconomic status and incidence of abnormal glucose metabolism: the Australian Diabetes, Obesity and Lifestyle (AusDiab) study.* Diabetes Care, 2012. **35**(7): p. 1455-61.

38. Wing, J.J., et al., *Change in Neighborhood Characteristics and Change in Coronary Artery Calcium: A Longitudinal Investigation in the MESA (Multi-Ethnic Study of Atherosclerosis) Cohort.* Circulation, 2016. **134**(7): p. 504-13.

39. Yang, Y.C., T. Li, and Y. Ji, *Impact of social integration on metabolic functions: evidence from a nationally representative longitudinal study of US older adults.* BMC Public Health, 2013. **13**: p. 1210.

40. Yang, Y.C., K. Schorpp, and K.M. Harris, *Social support, social strain and inflammation: Evidence from a national longitudinal study of U.S. adults.* Social Science & Medicine, 2014. **107**: p. 124-135.

41. Yang, Y.C., C. Boen, and K. Mullan Harris, *Social relationships and hypertension in late life: evidence from a nationally representative longitudinal study of older adults.* J Aging Health, 2015. **27**(3): p. 403-31.

42. Yang, Y.C., et al., *Social relationships and physiological determinants of longevity across the human life span.* Proc Natl Acad Sci U S A, 2016. **113**(3): p. 578-83.

43. Yao, Y., G. Wan, and D. Meng, *Income distribution and health: can polarization explain health outcomes better than inequality?* Eur J Health Econ, 2019. **20**(4): p. 543-557.

44. Zöller, B., et al., *Neighborhood deprivation and hospitalization for venous thromboembolism in Sweden.* J Thromb Thrombolysis, 2012. **34**(3): p. 374-82.

45. Adams, R.J., et al., *Effects of area deprivation on health risks and outcomes: a multilevel, cross-sectional, Australian population study.* Int J Public Health, 2009. **54**(3): p. 183-92.

46. Agyemang, C., et al., *Ethnic differences in the effect of environmental stressors on blood pressure and hypertension in the Netherlands.* BMC Public Health, 2007. **7**: p. 118.

47. Aliarzadeh, B., et al., *Association between socio-economic status and hemoglobin A1c levels in a Canadian primary care adult population without diabetes.* BMC Fam Pract, 2014. **15**: p. 7.

48. Altevers, J., et al., *Poor structural social support is associated with an increased risk of Type 2 diabetes mellitus: findings from the MONICA/KORA Augsburg cohort study.* Diabet Med, 2016. **33**(1): p. 47-54.

49. Andersen, A.F., et al., *Life-course socio-economic position, area deprivation and Type 2 diabetes: findings from the British Women's Heart and Health Study.* Diabet Med, 2008. **25**(12): p. 1462-8.

50. Auchincloss, A.H., et al., *Association of insulin resistance with distance to wealthy areas: the multi-ethnic study of atherosclerosis.* Am J Epidemiol, 2007. **165**(4): p. 389-97.

51. Bagheri, N., et al., *Community cardiovascular disease risk from cross-sectional general practice clinical data: a spatial analysis.* Prev Chronic Dis, 2015. **12**: p. E26.

52. Bagheri, N., et al., *Identifying hotspots of type 2 diabetes risk using general practice data and geospatial analysis: an approach to inform policy and practice.* Aust J Prim Health, 2019.

53. Bajaj, A., et al., *Daily social interactions, close relationships, and systemic inflammation in two samples: Healthy middle-aged and older adults.* Brain Behav Immun, 2016. **58**: p. 152-164.

54. Baldock, K., et al., *Associations between resident perceptions of the local residential environment and metabolic syndrome.* J Environ Public Health, 2012. **2012**: p. 589409.

55. Baldock, K.L., et al., *Gender-specific associations between perceived and objective neighbourhood crime and metabolic syndrome.* PLoS One, 2018. **13**(7): p. e0201336.

56. Barber, S., et al., *Double-jeopardy: The joint impact of neighborhood disadvantage and low social cohesion on cumulative risk of disease among African American men and women in the Jackson Heart Study.* Soc Sci Med, 2016. **153**: p. 107-15.

57. Barber, S., et al., *Neighborhood Disadvantage, Poor Social Conditions, and Cardiovascular Disease Incidence Among African American Adults in the Jackson Heart Study.* Am J Public Health, 2016. **106**(12): p. 2219-2226.

58. Barber, S., et al., *Neighborhood Disadvantage and Cumulative Biological Risk Among a Socioeconomically Diverse Sample of African American Adults: An Examination in the Jackson Heart Study.* Journal of Racial and Ethnic Health Disparities, 2016. **3**(3): p. 444-456.

59. Bhopal, R., et al., *Ethnic and socio-economic inequalities in coronary heart disease, diabetes and risk factors in Europeans and South Asians.* J Public Health Med, 2002. **24**(2): p. 95-105.

60. Bird, C.E., et al., *Neighbourhood socioeconomic status and biological 'wear and tear' in a nationally representative sample of US adults.* Journal of Epidemiology and Community Health, 2010. **64**(10): p. 860-865.

61. Bland, S.H., et al., *Social network and blood pressure: a population study.* Psychosom Med, 1991. **53**(6): p. 598-607.

62. Bland, S.H., et al., *Long term relations between earthquake experiences and coronary heart disease risk factors.* Am J Epidemiol, 2000. **151**(11): p. 1086-90.

63. Breckenkamp, J., A. Mielck, and O. Razum, *Health inequalities in Germany: do regional-level variables explain differentials in cardiovascular risk?* BMC Public Health, 2007. **7**: p. 132.

64. Bu, F., A. Steptoe, and D. Fancourt, *Relationship between loneliness, social isolation and modifiable risk factors for cardiovascular disease: a latent class analysis.* J Epidemiol Community Health, 2021.

65. Carels, R.A., J.A. Blumenthal, and A. Sherwood, *Effect of satisfaction with social support on blood pressure in normotensive and borderline hypertensive men and women.* Int J Behav Med, 1998. **5**(1): p. 76-85.

66. Cathorall, M.L., et al., *Neighborhood Disadvantage and Variations in Blood Pressure.* Am. J. Health Educ., 2015. **46**(5): p. 266-273.

67. Chaix, B., et al., *Residential environment and blood pressure in the PRIME Study: is the association mediated by body mass index and waist circumference?* J Hypertens, 2008. **26**(6): p. 1078-84.

68. Chaix, B., et al., *Individual/neighborhood social factors and blood pressure in the RECORD Cohort Study: which risk factors explain the associations?* Hypertension, 2010. **55**(3): p. 769-75.

69. Chaparro, M.P., et al., *Neighborhood deprivation and biomarkers of health in Britain: the mediating role of the physical environment.* BMC Public Health, 2018. **18**(1): p. 801.

70. Chichlowska, K.L., et al., *Individual and neighborhood socioeconomic status characteristics and prevalence of metabolic syndrome: the Atherosclerosis Risk in Communities (ARIC) Study.* Psychosom Med, 2008. **70**(9): p. 986-92.

71. Clark, C.R., et al., *Cardiovascular inflammation in healthy women: multilevel associations with state-level prosperity, productivity and income inequality.* BMC Public Health, 2012. **12**: p. 211.

72. Clark, C.R., et al., *Neighborhood disadvantage, neighborhood safety and cardiometabolic risk factors in African Americans: biosocial associations in the Jackson Heart study.* PLoS One, 2013. **8**(5): p. e63254.

73. Climie, R.E., et al., *Individual and Neighborhood Deprivation and Carotid Stiffness.* Hypertension, 2019. **73**(6): p. 1185-1194.

74. Cohn, T., et al., *Impact of Individual and Neighborhood Factors on Cardiovascular Risk in White Hispanic and Non-Hispanic Women and Men.* Res Nurs Health, 2017. **40**(2): p. 120-131.

75. Coulon, S.M., et al., *Multilevel Associations of Neighborhood Poverty, Crime, and Satisfaction With Blood Pressure in African-American Adults.* Am J Hypertens, 2016. **29**(1): p. 90-5.

76. Coulon, S.M., et al., *The Association of Neighborhood Gene-Environment Susceptibility with Cortisol and Blood Pressure in African-American Adults.* Ann Behav Med, 2016. **50**(1): p. 98-107.

77. Cozier, Y.C., et al., *Neighborhood Socioeconomic Status in Relation to Serum Biomarkers in the Black Women's Health Study.* J Urban Health, 2016. **93**(2): p. 279-91.

78. Creaven, A.M., S. Howard, and B.M. Hughes, *Social support and trait personality are independently associated with resting cardiovascular function in women.* Br J Health Psychol, 2013. **18**(3): p. 556-73.

79. Cross, R., et al., *Cross-sectional study of area-level disadvantage and glycaemic-related risk in community health service users in the Southern.IML Research (SIMLR) cohort.* Aust Health Rev, 2019. **43**(1): p. 85-91.

80. Cubbin, C. and M.A. Winkleby, *Protective and harmful effects of neighborhood-level deprivation on individual-level health knowledge, behavior changes, and risk of coronary heart disease.* Am J Epidemiol, 2005. **162**(6): p. 559-68.

81. De Moraes, A.C.F., et al., *Sex and ethnicity modify the associations between individual and contextual socioeconomic indicators and ideal cardiovascular health: MESA study.* J Public Health (Oxf), 2019. **41**(3): p. e237-e244.

82. Deans, K.A., et al., *Differences in atherosclerosis according to area level socioeconomic deprivation: cross sectional, population based study.* Bmj, 2009. **339**: p. b4170.

83. Diez Roux, A.V., D.R. Jacobs, and C.I. Kiefe, *Neighborhood characteristics and components of the insulin resistance syndrome in young adults: the coronary artery risk development in young adults (CARDIA) study.* Diabetes Care, 2002. **25**(11): p. 1976-82.

84. Diez-Roux, A.V., et al., *Neighborhood environments and coronary heart disease: a multilevel analysis.* Am J Epidemiol, 1997. **146**(1): p. 48-63.

85. Djekic, D., et al., *Impact of socioeconomic status on coronary artery calcification.* Eur J Prev Cardiol, 2018. **25**(16): p. 1756-1764.

86. Do, D.P., et al., *Circadian rhythm of cortisol and neighborhood characteristics in a population-based sample: the Multi-Ethnic Study of Atherosclerosis.* Health Place, 2011. **17**(2): p. 625-32.

87. Dragano, N., et al., *Subclinical coronary atherosclerosis and neighbourhood deprivation in an urban region.* Eur J Epidemiol, 2009. **24**(1): p. 25-35.

88. Dubowitz, T., et al., *The Women's Health Initiative: The food environment, neighborhood socioeconomic status, BMI, and blood pressure.* Obesity (Silver Spring), 2012. **20**(4): p. 862-71.

89. Duncan, D.T., et al., *Perceived spatial stigma, body mass index and blood pressure: a global positioning system study among low-income housing residents in New York City.* Geospat Health, 2016. **11**(2): p. 399.

90. Dwane, N., N. Wabiri, and S. Manda, *Small-area variation  of cardiovascular diseases and select risk factors and their association to household and area poverty in South Africa: Capturing emerging trends in South Africa to better target local level interventions.* PLoS One, 2020. **15**(4): p. e0230564.

91. Eichinger, M., et al., *How are physical activity behaviors and cardiovascular risk factors associated with characteristics of the built and social residential environment?* PLoS One, 2015. **10**(6): p. e0126010.

92. Ellaway, A. and S. Macintyre, *Is social participation associated with cardiovascular disease risk factors?* Soc Sci Med, 2007. **64**(7): p. 1384-91.

93. Engström, G., et al., *Geographic distribution of stroke incidence within an urban population: Relations to socioeconomic circumstances and prevalence of cardiovascular risk factors.* Stroke, 2001. **32**(5): p. 1098-1103.

94. Ferguson, T.S., et al., *Neighbourhood socioeconomic characteristics and blood pressure among Jamaican youth: a pooled analysis of data from observational studies.* PeerJ, 2020. **8**: p. e10058.

95. Finch, B.K., et al., *Neighborhood effects on health: Concentrated advantage and disadvantage.* Health & Place, 2010. **16**(5): p. 1058-1060.

96. Foraker, R.E., et al., *Distribution of Cardiovascular Health by Individual- and Neighborhood-Level Socioeconomic Status: Findings From the Jackson Heart Study.* Glob Heart, 2019. **14**(3): p. 241-250.

97. Ford, E.S., E.B. Loucks, and L.F. Berkman, *Social integration and concentrations of C-reactive protein among US adults.* Ann Epidemiol, 2006. **16**(2): p. 78-84.

98. Ford, J., et al., *Social Integration and Quality of Social Relationships as Protective Factors for Inflammation in a Nationally Representative Sample of Black Women.* J Urban Health, 2019. **96**(Suppl 1): p. 35-43.

99. Fuller, K.C., et al., *ACE gene haplotypes and social networks: Using a biocultural framework to investigate blood pressure variation in African Americans.* Plos One, 2018. **13**(9).

100. Gallo, L.C., et al., *Individual and neighborhood socioeconomic status and inflammation in Mexican American women: what is the role of obesity?* Psychosom Med, 2012. **74**(5): p. 535-42.

101. Garcia, L., et al., *The Impact of Neighborhood Socioeconomic Position on Prevalence of Diabetes and Prediabetes in Older Latinos: The Sacramento Area Latino Study on Aging.* Hisp Health Care Int, 2015. **13**(2): p. 77-85.

102. Gebreab, S.Y., et al., *Geographic variations in cardiovascular health in the United States: contributions of state- and individual-level factors.* J Am Heart Assoc, 2015. **4**(6): p. e001673.

103. Grimaud, O., et al., *Gender differences in the association between socioeconomic status and subclinical atherosclerosis.* PLoS One, 2013. **8**(11): p. e80195.

104. Hanson, B.S., et al., *Social anchorage and blood pressure in elderly men--a population study.* J Hypertens, 1988. **6**(6): p. 503-10.

105. Helminen, A., et al., *Validity assessment of a social support index.* Scand J Soc Med, 1995. **23**(1): p. 66-74.

106. Helminen, A., et al., *Carotid atherosclerosis in middle-aged men. Relation to conjugal circumstances and social support.* Scand J Soc Med, 1995. **23**(3): p. 167-72.

107. Helminen, A., et al., *Social network in relation to plasma fibrinogen.* J Biosoc Sci, 1997. **29**(2): p. 129-39.

108. Henning, C.H., et al., *Identification of direct and indirect social network effects in the pathophysiology of insulin resistance in obese human subjects.* PLoS One, 2014. **9**(4): p. e93860.

109. Hickson, D.A., et al., *Socioeconomic position is positively associated with blood pressure dipping among African-American adults: the Jackson Heart Study.* Am J Hypertens, 2011. **24**(9): p. 1015-21.

110. Höfelmann, D.A., et al., *Is income area level associated with blood pressure in adults regardless of individual-level characteristics? A multilevel approach.* Health Place, 2012. **18**(5): p. 971-7.

111. Holmes, L.M. and E.A. Marcelli, *Neighborhoods and systemic inflammation: High CRP among legal and unauthorized Brazilian migrants.* Health Place, 2012. **18**(3): p. 683-693.

112. Horsten, M., et al., *Social relations and the metabolic syndrome in middle-aged Swedish women.* J Cardiovasc Risk, 1999. **6**(6): p. 391-7.

113. Hosseini, Z., et al., *Social connections and hypertension in women and men: a population-based cross-sectional study of the Canadian Longitudinal Study on Aging.* J Hypertens, 2020.

114. Hughes, B.M., *Social support in ordinary life and laboratory measures of cardiovascular reactivity: gender differences in habituation-sensitization.* Ann Behav Med, 2007. **34**(2): p. 166-76.

115. Islam, S.J., et al., *Neighborhood Characteristics and Ideal Cardiovascular Health Among Black Adults: Results From the Morehouse-Emory Cardiovascular (MECA) Center for Health Equity.* Ann Epidemiol, 2020.

116. Keita, A.D., et al., *Associations of neighborhood area level deprivation with the metabolic syndrome and inflammation among middle- and older- age adults.* BMC Public Health, 2014. **14**: p. 1319.

117. Kelli, H.M., et al., *Association Between Living in Food Deserts and Cardiovascular Risk.* Circ Cardiovasc Qual Outcomes, 2017. **10**(9).

118. Kent de Grey, R.G., et al., *Enemies and friends in high-tech places: the development and validation of the Online Social Experiences Measure.* Digit Health, 2019. **5**: p. 2055207619878351.

119. Kim, K., et al., *Associations between social network properties and metabolic syndrome and the mediating effect of physical activity: findings from the Cardiovascular and Metabolic Diseases Etiology Research Center (CMERC) Cohort.* BMJ Open Diabetes Res Care, 2020. **8**(1).

120. King, K.E., J.D. Morenoff, and J.S. House, *Neighborhood context and social disparities in cumulative biological risk factors.* Psychosom Med, 2011. **73**(7): p. 572-9.

121. Lawlor, D.A., et al., *Life-course socioeconomic position, area deprivation, and coronary heart disease: findings from the British Women's Heart and Health Study.* Am J Public Health, 2005. **95**(1): p. 91-7.

122. Lee, J.E. and K.E. Cichy, *Complex Role of Touch in Social Relationships for Older Adults' Cardiovascular Disease Risk.* Res Aging, 2020. **42**(7-8): p. 208-216.

123. Lewis, T.T., et al., *Race, psychosocial factors, and aortic pulse wave velocity: the Health, Aging, and Body Composition Study.* J Gerontol A Biol Sci Med Sci, 2010. **65**(10): p. 1079-85.

124. Li, K., M. Wen, and K.A. Henry, *Ethnic density, immigrant enclaves, and Latino health risks: A propensity score matching approach.* Soc Sci Med, 2017. **189**: p. 44-52.

125. Li, K., M. Wen, and J.X. Fan, *Neighborhood Racial Diversity and Metabolic Syndrome: 2003-2008 National Health and Nutrition Examination Survey.* J Immigr Minor Health, 2019. **21**(1): p. 151-160.

126. Linden, W., et al., *Sex differences in social support, self-deception, hostility, and ambulatory cardiovascular activity.* Health Psychol, 1993. **12**(5): p. 376-80.

127. Loose, F., et al., *Blood pressure and psychological distress among North Africans in France: The role of perceived personal/group discrimination and gender.* Am J Hum Biol, 2017. **29**(5).

128. Loucks, E.B., et al., *Social integration is associated with fibrinogen concentration in elderly men.* Psychosom Med, 2005. **67**(3): p. 353-8.

129. Loucks, E.B., et al., *Relation of social integration to inflammatory marker concentrations in men and women 70 to 79 years.* Am J Cardiol, 2006. **97**(7): p. 1010-6.

130. Loucks, E.B., et al., *Social networks and inflammatory markers in the Framingham Heart Study.* J Biosoc Sci, 2006. **38**(6): p. 835-42.

131. Maki, K.G., *Social support, strain, and glycemic control: A path analysis.* Pers. Relat., 2020. **27**(3): p. 592-612.

132. Matricciani, L.A., et al., *Investigating individual- and area-level socioeconomic gradients of pulse pressure among normotensive and hypertensive participants.* Int J Environ Res Public Health, 2013. **10**(2): p. 571-89.

133. McKenzie, J.A., et al., *Ideal cardiovascular health in urban Jamaica: prevalence estimates and relationship to community property value, household assets and educational attainment: a cross-sectional study.* BMJ Open, 2020. **10**(12): p. e040664.

134. Mellman, T.A., et al., *Blood Pressure Dipping and Urban Stressors in Young Adult African Americans.* Ann Behav Med, 2015. **49**(4): p. 622-7.

135. Merkin, S.S., et al., *Neighborhoods and cumulative biological risk profiles by race/ethnicity in a national sample of U.S. adults: NHANES III.* Ann Epidemiol, 2009. **19**(3): p. 194-201.

136. Merlo, J., et al., *Diastolic blood pressure and area of residence: multilevel versus ecological analysis of social inequity.* J Epidemiol Community Health, 2001. **55**(11): p. 791-8.

137. Metcalf, P.A., et al., *Comparison of different markers of socioeconomic status with cardiovascular disease and diabetes risk factors in the Diabetes, Heart and Health Survey.* N Z Med J, 2008. **121**(1269): p. 45-56.

138. Meza, B.P.L., et al., *Social network factors and cardiovascular health among baltimore public housing residents.* Prev Med Rep, 2020. **20**: p. 101192.

139. Mobley, L.R., et al., *Environment, obesity, and cardiovascular disease risk in low-income women.* Am J Prev Med, 2006. **30**(4): p. 327-332.

140. Murakami, K., et al., *Neighborhood socioeconomic status in relation to dietary intake and insulin resistance syndrome in female Japanese dietetic students.* Nutrition, 2010. **26**(5): p. 508-14.

141. Naimi, A.I., et al., *Associations between area-level unemployment, body mass index, and risk factors for cardiovascular disease in an urban area.* Int J Environ Res Public Health, 2009. **6**(12): p. 3082-96.

142. Neergheen, V.L., et al., *Neighborhood social cohesion is associated with lower levels of interleukin-6 in African American women.* Brain Behav Immun, 2019. **76**: p. 28-36.

143. Nordstrom, C.K., et al., *The association of personal and neighborhood socioeconomic indicators with subclinical cardiovascular disease in an elderly cohort. The cardiovascular health study.* Soc Sci Med, 2004. **59**(10): p. 2139-47.

144. Petersen, K.L., et al., *Community socioeconomic status is associated with circulating interleukin-6 and C-reactive protein.* Psychosomatic Medicine, 2008. **70**(6): p. 646-652.

145. Piferi, R.L. and K.A. Lawler, *Social support and ambulatory blood pressure: an examination of both receiving and giving.* Int J Psychophysiol, 2006. **62**(2): p. 328-36.

146. Pollack, C.E., et al., *Neighborhood socioeconomic status and coronary heart disease risk prediction in a nationally representative sample.* Public Health, 2012. **126**(10): p. 827-35.

147. Pollard, T.M., et al., *Social networks and coronary heart disease risk factors in South Asians and Europeans in the UK.* Ethn Health, 2003. **8**(3): p. 263-75.

148. Ribeiro, A.I., et al., *Neighbourhood socioeconomic deprivation and allostatic load: a multi-cohort study.* Sci Rep, 2019. **9**(1): p. 8790.

149. Riva, M., C.V. Larsen, and P. Bjerregaard, *Association between individual-level and community-level socio-economic status and blood pressure among Inuit in Greenland.* Int J Circumpolar Health, 2016. **75**: p. 32757.

150. Robinette, J.W., et al., *Neighborhood features and physiological risk: An examination of allostatic load.* Health & Place, 2016. **41**: p. 110-118.

151. Robinette, J.W., J.D. Boardman, and E. Crimmins, *Perceived neighborhood social cohesion and cardiometabolic risk: a gene × environment study.* Biodemography Soc Biol, 2020. **65**(1): p. 1-15.

152. Rosvall, M., et al., *Area social characteristics and carotid atherosclerosis.* Eur. J. Public Health, 2007. **17**(4): p. 333-339.

153. Samuel, L.J., et al., *Community Characteristics are Associated with Blood Pressure Levels in a Racially Integrated Community.* J Urban Health, 2015. **92**(3): p. 403-14.

154. Schulz, A.J., et al., *Do observed or perceived characteristics of the neighborhood environment mediate associations between neighborhood poverty and cumulative biological risk?* Health & Place, 2013. **24**: p. 147-156.

155. Seeman, T.E., et al., *Social relationships and their biological correlates: Coronary Artery Risk Development in Young Adults (CARDIA) study.* Psychoneuroendocrinology, 2014. **43**: p. 126-38.

156. Smith, G.D., et al., *Individual social class, area-based deprivation, cardiovascular disease risk factors, and mortality: the Renfrew and Paisley Study.* J Epidemiol Community Health, 1998. **52**(6): p. 399-405.

157. Sprung, M.R., et al., *Neighborhood crime is differentially associated with cardiovascular risk factors as a function of race and sex.* J Public Health Res, 2019. **8**(3): p. 1643.

158. Steppuhn, H., et al., *Individual and area-level determinants associated with C-reactive protein as a marker of cardiometabolic risk among adults: Results from the German National Health Interview and Examination Survey 2008-2011.* PLoS One, 2019. **14**(2): p. e0211774.

159. Strogatz, D.S., et al., *Social support, stress, and blood pressure in black adults.* Epidemiology, 1997. **8**(5): p. 482-7.

160. Theorell, T., et al., *Blood pressure variations across areas in the greater Stockholm region: analysis of 74,000 18-year-old men.* Soc Sci Med, 1982. **16**(4): p. 469-73.

161. Toms, R., et al., *Geographic variation in cardiometabolic risk factor prevalence explained by area-level disadvantage in the Illawarra-Shoalhaven region of the NSW, Australia.* Sci Rep, 2020. **10**(1): p. 12770.

162. Troxel, W.M., et al., *Social integration, social contacts, and blood pressure dipping in African-Americans and whites.* J Hypertens, 2010. **28**(2): p. 265-71.

163. Uchino, B.N., et al., *The quality of spouses' social networks contributes to each other's cardiovascular risk.* PLoS One, 2013. **8**(8): p. e71881.

164. Unger, E., et al., *Association of neighborhood characteristics with cardiovascular health in the multi-ethnic study of atherosclerosis.* Circ Cardiovasc Qual Outcomes, 2014. **7**(4): p. 524-31.

165. Wagner, K.J., et al., *Effects of neighborhood socioeconomic status on blood pressure in older adults.* Rev Saude Publica, 2016. **50**: p. 78.

166. Whittaker, K.S., et al., *Combining psychosocial data to improve prediction of cardiovascular disease risk factors and events: The National Heart, Lung, and Blood Institute--sponsored Women's Ischemia Syndrome Evaluation study.* Psychosom Med, 2012. **74**(3): p. 263-70.

167. Willets, C., et al., *Association Between Perceived Neighborhood Characteristics and Carotid Artery Intima-Media Thickness: Cross-Sectional Results From the ELSA-Brasil Study.* Glob Heart, 2019. **14**(4): p. 379-385.

168. Zanelatto, C., et al., *Perception of neighborhood disorder and blood pressure in adults: a multilevel population-based study.* Cad Saude Publica, 2019. **35**(2): p. e00016418.
