# Supplementary material for: Associations between dimensions of the social environment and cardiometabolic risk factors: Systematic review and meta-analysis
Source: SSM Popul Health. 2023 Nov 25;25:101559. doi: 10.1016/j.ssmph.2023.101559 (PMC10749911; doi:10.1016/j.ssmph.2023.101559)
Supplement: Multimedia component 3 [file mmc3.docx]

| **Supplementary Table 1.** Effect estimates of included studies | | | | | | |  | | | | | | |
| --- | --- | --- | --- | --- | --- | --- | --- | --- | --- | --- | --- | --- | --- |
| **Reference** | **First author, year** | **Social Environment dimension** | **Outcome category** | **Sex-specific** | **Effect estimate** | **Association n** | | **Association level** | **Categories** | **Effects estimate** | **Lower bound 95%CI** | **Upper bound 95%CI** | **SE** |
| [1] | Adams, 2009 | Economic and Social Disadvantage | CVD risk scores | male | OR (95%CI) | 1 | | 1 | I (lowest disadvantage, reference) | 1.00 | - | - |  |
|  |  |  |  | male |  | 1 | | 2 | II | 1.00 | - | - |  |
|  |  |  |  | male |  | 1 | | 3 | III | 1.10 | - | - |  |
|  |  |  |  | male |  | 1 | | 4 | IV | 1.00 | - | - |  |
|  |  |  |  | male |  | 1 | | 5 | V | 1.50 | - | - |  |
|  |  | Economic and Social Disadvantage | CVD risk scores | female | OR (95%CI) | 2 | | 1 | I (lowest disadvantage, reference) | 1.00 | - | - |  |
|  |  |  |  | female |  | 2 | | 2 | II | 0.90 | - | - |  |
|  |  |  |  | female |  | 2 | | 3 | III | 0.90 | - | - |  |
|  |  |  |  | female |  | 2 | | 4 | IV | 0.70 | - | - |  |
|  |  |  |  | female |  | 2 | | 5 | V | 0.40 | - | - |  |
| [2] | Adjaye-Gbewonyo, 2018 | Economic and Social Disadvantage | cardiovascular health-related risk factors | overall | Beta (95%CI) | 1 | | 1 |  | -1.14 | -4.19 | 1.90 |  |
|  |  | Economic and Social Disadvantage | cardiovascular health-related risk factors | overall | Beta (95%CI) | 2 | | 1 |  | 1.31 | -1.17 | 3.79 |  |
| [3] | Agyemang, 2007 | Crime and Safety | cardiovascular health-related risk factors | overall | Beta (95%CI) | 1 | | 1 | Dutch | 1.03 | -2.88 | 4.93 |  |
|  |  | Crime and Safety | cardiovascular health-related risk factors | overall |  | 2 | | 1 | Turk | 1.11 | -2.81 | 5.02 |  |
|  |  | Crime and Safety | cardiovascular health-related risk factors | overall |  | 3 | | 1 | Moroccan | 3.31 | -1.59 | 8.21 |  |
|  |  | Civic Participation and Engagement | cardiovascular health-related risk factors | overall | Beta (95%CI) | 4 | | 1 | Dutch | -1.72 | -5.66 | 2.23 |  |
|  |  | Civic Participation and Engagement | cardiovascular health-related risk factors | overall |  | 5 | | 1 | Turk | -2.40 | -6.31 | 1.52 |  |
|  |  | Civic Participation and Engagement | cardiovascular health-related risk factors | overall |  | 6 | | 1 | Moroccan | -5.27 | -9.67 | -0.87 |  |
|  |  | Crime and Safety | cardiovascular health-related risk factors | overall | Beta (95%CI) | 7 | | 1 | Dutch | 0.80 | -1.74 | 3.33 |  |
|  |  | Crime and Safety | cardiovascular health-related risk factors | overall |  | 8 | | 1 | Turk | 2.96 | 0.71 | 5.20 |  |
|  |  | Crime and Safety | cardiovascular health-related risk factors | overall |  | 9 | | 1 | Moroccan | 0.54 | -2.90 | 3.98 |  |
|  |  | Civic Participation and Engagement | cardiovascular health-related risk factors | overall | Beta (95%CI) | 10 | | 1 | Dutch | -0.57 | -3.18 | 2.04 |  |
|  |  | Civic Participation and Engagement | cardiovascular health-related risk factors | overall |  | 11 | | 1 | Turk | -0.77 | -3.61 | 2.06 |  |
|  |  | Civic Participation and Engagement | cardiovascular health-related risk factors | overall |  | 12 | | 1 | Moroc. | -2.76 | -5.89 | 0.38 |  |
| [4] | Aliarzadeh, 2014 | Economic and Social Disadvantage | glucose metabolism-related risk factors | overall | Beta | 1 | | 1 | Most deprived  (reference) | - | - | - |  |
|  |  |  |  | overall |  | 1 | | 2 | Deprived | 0.00 | -0.03 | 0.04 |  |
|  |  |  |  | overall |  | 1 | | 3 | Neutral | -0.02 | -0.05 | 0.01 |  |
|  |  |  |  | overall |  | 1 | | 4 | Less deprived | -0.02 | -0.06 | 0.01 |  |
|  |  |  |  | overall |  | 1 | | 5 | Least deprived | -0.02 | -0.05 | 0.01 |  |
|  |  | Economic and Social Disadvantage | glucose metabolism-related risk factors | overall | Beta | 2 | | 1 | Most deprived (reference) | - | - | - |  |
|  |  |  |  | overall |  | 2 | | 2 | Deprived | -0.05 | -0.09 | -0.01 |  |
|  |  |  |  | overall |  | 2 | | 3 | Neutral | -0.03 | -0.07 | 0.01 |  |
|  |  |  |  | overall |  | 2 | | 4 | Less deprived | -0.04 | -0.08 | 0.00 |  |
|  |  |  |  | overall |  | 2 | | 5 | Least deprived | -0.04 | -0.07 | 0.00 |  |
|  |  | Economic and Social Disadvantage | glucose metabolism-related risk factors | overall | Beta | 3 | | 1 | Most deprived (reference) | - | - | - |  |
|  |  |  |  | overall |  | 3 | | 2 | Deprived | 0.02 | -0.01 | 0.05 |  |
|  |  |  |  | overall |  | 3 | | 3 | Neutral | 0.01 | -0.02 | 0.04 |  |
|  |  |  |  | overall |  | 3 | | 4 | Less deprived | 0.01 | -0.02 | 0.04 |  |
|  |  |  |  | overall |  | 3 | | 5 | Least deprived | 0.02 | -0.01 | 0.05 |  |
|  |  | Economic and Social Disadvantage | glucose metabolism-related risk factors | overall | Beta | 4 | | 1 | Most deprived  (reference) | - | - | - |  |
|  |  |  |  | overall |  | 4 | | 2 | Deprived | -0.02 | -0.05 | 0.02 |  |
|  |  |  |  | overall |  | 4 | | 3 | Neutral | -0.01 | -0.05 | 0.02 |  |
|  |  |  |  | overall |  | 4 | | 4 | Less deprived | 0.00 | -0.04 | 0.03 |  |
|  |  |  |  | overall |  | 4 | | 5 | Least deprived | -0.01 | -0.04 | 0.02 |  |
| [5] | Altevers, 2016 | Social Relationships and Norms | metabolic and inflammatory-related risk factors | male | Prevalence | 1 | | 1 | poor structural social support | 48% | - | - |  |
|  |  |  |  | male |  | 1 | | 2 | good structural social support | 45% | - | - |  |
|  |  | Social Relationships and Norms | metabolic and inflammatory-related risk factors | female | Prevalence | 2 | | 1 | poor structural social support | 19% | - | - |  |
|  |  |  |  | female |  | 2 | | 2 | good structural social support | 14% | - | - |  |
| [6] | Andell, 2020 | Economic and Social Disadvantage | cardiovascular health-related risk factors | overall | OR (95%CI) | 1 | | 1 | High SES (reference) | 1.00 | - | - | - |
|  |  |  |  | overall |  | 1 | | 2 | Moderate SES | 1.07 | 1.05 | 1.09 |  |
|  |  |  |  | overall |  | 1 | | 3 | Low SES | 1.06 | 1.03 | 1.08 |  |
|  |  | Economic and Social Disadvantage | cardiovascular health-related risk factors | overall | OR (95%CI) | 2 | | 1 | High SES (reference) | 1.00 | - | - |  |
|  |  |  |  | overall |  | 2 | | 2 | Moderate SES | 1.55 | 0.60 | 4.05 |  |
|  |  |  |  | overall |  | 2 | | 3 | Low SES | 2.73 | 1.05 | 7.12 |  |
| [7] | Andersen, 2008 | Economic and Social Disadvantage | glucose metabolism-related risk factors | female | Beta (95%CI) | 1 | | 1 |  | 1.61 | -0.27 | 3.54 |  |
|  |  |  |  | female | Beta (95%CI) | 2 | | 1 |  | 0.62 | 0.10 | 1.15 |  |
|  |  | Economic and Social Disadvantage | cardiovascular health-related risk factors | female | Beta (95%CI) | 3 | | 1 | 1 (most affluent) | 146.70 | 144.80 | 148.70 |  |
|  |  |  |  | female |  | 3 | | 2 | 2 | 149.20 | 147.40 | 151.00 |  |
|  |  |  |  | female |  | 3 | | 3 | 3 | 149.60 | 147.90 | 151.40 |  |
|  |  |  |  | female |  | 3 | | 4 | 4 | 149.70 | 148.30 | 151.10 |  |
|  |  |  |  | female |  | 3 | | 5 | 5 (most deprived) | 150.00 | 148.80 | 151.90 |  |
|  |  | Economic and Social Disadvantage | metabolic and inflammatory-related risk factors | female | Mean (95%CI) | 4 | | 1 | 1 (most affluent) | 1.72 | 1.69 | 1.76 |  |
|  |  |  |  | female |  | 4 | | 2 | 2 | 1.75 | 1.71 | 1.79 |  |
|  |  |  |  | female |  | 4 | | 3 | 3 | 1.65 | 1.62 | 1.69 |  |
|  |  |  |  | female |  | 4 | | 4 | 4 | 1.62 | 1.59 | 1.64 |  |
|  |  |  |  | female |  | 4 | | 5 | 5 (most deprived) | 1.60 | 1.57 | 1.63 |  |
|  |  | Economic and Social Disadvantage | metabolic and inflammatory-related risk factors | female | Beta (95%CI) | 5 | | 1 | 1 (most affluent) | 1.70 | 1.63 | 1.77 |  |
|  |  |  |  | female |  | 5 | | 2 | 2 | 1.73 | 1.66 | 1.79 |  |
|  |  |  |  | female |  | 5 | | 3 | 3 | 1.86 | 1.79 | 1.94 |  |
|  |  |  |  | female |  | 5 | | 4 | 4 | 1.94 | 1.87 | 2.01 |  |
|  |  |  |  | female |  | 5 | | 5 | 5 (most deprived) | 1.99 | 1.88 | 2.10 |  |
| [8] | Auchincloss, 2007 | Economic and Social Disadvantage | glucose metabolism-related risk factors | overall | Beta (95%CI) | 1 | | 1 |  | 0.97 | 0.92 | 1.03 |  |
|  |  | Economic and Social Disadvantage | glucose metabolism-related risk factors | overall | Beta (95%CI) | 2 | | 1 |  | 1.13 | 1.07 | 1.19 |  |
| [9] | Bagheri, 2015 | Economic and Social Disadvantage | CVD risk scores | overall | Beta | 1 | | 1 | Most disadvantaged areas | 7.90 | - | - |  |
|  |  |  |  | overall |  | 1 | | 2 | Moderately disadvantaged areas | 6.60 | - | - |  |
|  |  |  |  | overall |  | 1 | | 3 | Least disadvantaged areas | 6.20 | - | - |  |
|  |  | Economic and Social Disadvantage | CVD risk scores | overall | Beta | 2 | | 1 | Most disadvantaged areas | 16.40 | - | - |  |
|  |  |  |  | overall |  | 2 | | 2 | Moderately disadvantaged areas | 14.20 | - | - |  |
|  |  |  |  | overall |  | 2 | | 3 | Least disadvantaged areas | 13.40 | - | - |  |
| [10] | Bagheri, 2019 | Economic and Social Disadvantage | CVD risk scores | overall | Mean (95%CI) | 1 | | 1 | Low | 8.60 | 8.40 | 8.80 |  |
|  |  |  |  | overall |  | 1 | | 2 | Moderate | 8.60 | 8.40 | 8.70 |  |
|  |  |  |  | overall |  | 1 | | 3 | High | 8.30 | 8.20 | 8.50 |  |
| [11] | Bajaj, 2016 | Social Relationships and Norms | metabolic and inflammatory-related risk factors | overall | Beta | 1 | | 1 |  | na | na | na |  |
|  |  | Social Relationships and Norms | metabolic and inflammatory-related risk factors | overall | Beta | 2 | | 1 |  | na | na | na |  |
|  |  | Social Relationships and Norms | metabolic and inflammatory-related risk factors | overall | Beta | 3 | | 1 |  | na | na | na |  |
|  |  | Social Relationships and Norms | metabolic and inflammatory-related risk factors | overall | Beta | 4 | | 1 |  | na | na | na |  |
|  |  | Social Relationships and Norms | metabolic and inflammatory-related risk factors | overall | Beta | 5 | | 1 |  | −0.01 | - | - |  |
|  |  | Social Relationships and Norms | metabolic and inflammatory-related risk factors | overall | Beta | 6 | | 1 |  | −0.01 | - | - |  |
|  |  | Social Relationships and Norms | metabolic and inflammatory-related risk factors | overall | Beta | 7 | | 1 |  | 0.04 | - | - |  |
|  |  | Social Relationships and Norms | metabolic and inflammatory-related risk factors | overall | Beta | 8 | | 1 |  | −0.11 | - | - |  |
|  |  | Social Relationships and Norms | metabolic and inflammatory-related risk factors | overall | Beta | 9 | | 1 |  | 0.03 | - | - |  |
|  |  | Social Relationships and Norms | metabolic and inflammatory-related risk factors | overall | Beta | 10 | | 1 |  | −0.11 | - | - |  |
|  |  | Social Relationships and Norms | metabolic and inflammatory-related risk factors | overall | Beta | 11 | | 1 |  | 0.09 | - | - |  |
|  |  | Social Relationships and Norms | metabolic and inflammatory-related risk factors | overall | Beta | 12 | | 1 |  | 0.01 | - | - |  |
|  |  | Social Relationships and Norms | metabolic and inflammatory-related risk factors | overall | Beta | 13 | | 1 |  | 0.09 | - | - |  |
|  |  | Social Relationships and Norms | metabolic and inflammatory-related risk factors | overall | Beta | 14 | | 1 |  | 0.01 | - | - |  |
|  |  | Social Relationships and Norms | metabolic and inflammatory-related risk factors | overall | Beta | 15 | | 1 |  | 0.02 | - | - |  |
|  |  | Social Relationships and Norms | metabolic and inflammatory-related risk factors | overall | Beta | 16 | | 1 |  | 0.00 | - | - |  |
|  |  | Social Relationships and Norms | metabolic and inflammatory-related risk factors | overall | Beta | 17 | | 1 |  | 0.05 | - | - |  |
|  |  | Social Relationships and Norms | metabolic and inflammatory-related risk factors | overall | Beta | 18 | | 1 |  | −0.01 | - | - |  |
|  |  | Social Relationships and Norms | metabolic and inflammatory-related risk factors | overall | Beta | 19 | | 1 |  | 0.03 | - | - |  |
|  |  | Social Relationships and Norms | metabolic and inflammatory-related risk factors | overall | Beta | 20 | | 1 |  | −0.10 | - | - |  |
|  |  | Social Relationships and Norms | metabolic and inflammatory-related risk factors | overall | Beta | 21 | | 1 |  | −0.05 | - | - |  |
|  |  | Social Relationships and Norms | metabolic and inflammatory-related risk factors | overall | Beta | 22 | | 1 |  | −0.001 | - | - |  |
|  |  | Social Relationships and Norms | metabolic and inflammatory-related risk factors | overall | Beta | 23 | | 1 |  | −0.05 | - | - |  |
|  |  | Social Relationships and Norms | metabolic and inflammatory-related risk factors | overall | Beta | 24 | | 1 |  | −0.03 | - | - |  |
|  |  | Social Relationships and Norms | metabolic and inflammatory-related risk factors | overall | Beta | 25 | | 1 |  | 0.04 | - | - |  |
|  |  | Social Relationships and Norms | metabolic and inflammatory-related risk factors | overall | Beta | 26 | | 1 |  | 0.06 | - | - |  |
|  |  | Social Relationships and Norms | metabolic and inflammatory-related risk factors | overall | Beta | 27 | | 1 |  | 0.04 | - | - |  |
|  |  | Social Relationships and Norms | metabolic and inflammatory-related risk factors | overall | Beta | 28 | | 1 |  | 0.03 | - | - |  |
|  |  | Social Relationships and Norms | metabolic and inflammatory-related risk factors | overall | Beta | 29 | | 1 |  | 0.04 | - | - |  |
|  |  | Social Relationships and Norms | metabolic and inflammatory-related risk factors | overall | Beta | 30 | | 1 |  | −0.14 | - | - |  |
|  |  | Social Relationships and Norms | metabolic and inflammatory-related risk factors | overall | Beta | 31 | | 1 |  | 0.07 | - | - |  |
|  |  | Social Relationships and Norms | metabolic and inflammatory-related risk factors | overall | Beta | 32 | | 1 |  | −0.01 | - | - |  |
|  |  | Social Relationships and Norms | metabolic and inflammatory-related risk factors | overall | Beta | 33 | | 1 |  | −0.03 | - | - |  |
|  |  | Social Relationships and Norms | metabolic and inflammatory-related risk factors | overall | Beta | 34 | | 1 |  | −0.09 | - | - |  |
|  |  | Social Relationships and Norms | metabolic and inflammatory-related risk factors | overall | Beta | 35 | | 1 |  | −0.11 | - | - |  |
|  |  | Social Relationships and Norms | metabolic and inflammatory-related risk factors | overall | Beta | 36 | | 1 |  | −0.03 | - | - |  |
|  |  | Social Relationships and Norms | metabolic and inflammatory-related risk factors | overall | Beta | 37 | | 1 |  | 0.02 | - | - |  |
|  |  | Social Relationships and Norms | metabolic and inflammatory-related risk factors | overall | Beta | 38 | | 1 |  | 0.08 | - | - |  |
|  |  | Social Relationships and Norms | metabolic and inflammatory-related risk factors | overall | Beta | 39 | | 1 |  | −0.05 | - | - |  |
|  |  | Social Relationships and Norms | metabolic and inflammatory-related risk factors | overall | Beta | 40 | | 1 |  | 0.04 | - | - |  |
| [12] | Baldock, 2018 | Crime and Safety | CVD risk scores | overall | OR (95%CI) | 1 | | 1 |  | 1.15 | 1.01 | 1.31 |  |
| [13] | Baldock, 2012 | Crime and Safety | CVD risk scores | male | OR (95%CI) | 1 | | 1 |  | 1.06 | 0.89 | 1.27 |  |
|  |  | Crime and Safety | CVD risk scores | male | OR (95%CI) | 2 | | 1 |  | 1.18 | 1.06 | 1.30 |  |
|  |  | Crime and Safety | CVD risk scores | male | OR (95%CI) | 3 | | 1 |  | 1.21 | 1.12 | 1.32 |  |
|  |  | Crime and Safety | CVD risk scores | male | OR (95%CI) | 4 | | 1 |  | 1.14 | 1.00 | 1.29 |  |
|  |  | Crime and Safety | CVD risk scores | female | OR (95%CI) | 5 | | 1 |  | 1.34 | 1.14 | 1.59 |  |
|  |  | Crime and Safety | CVD risk scores | female | OR (95%CI) | 6 | | 1 |  | 1.06 | 0.87 | 1.29 |  |
|  |  | Crime and Safety | CVD risk scores | female | OR (95%CI) | 7 | | 1 |  | 1.05 | 0.82 | 1.34 |  |
|  |  | Crime and Safety | CVD risk scores | female | OR (95%CI) | 8 | | 1 |  | 1.01 | 0.83 | 1.23 |  |
| [14] | Barber, 2016 | Economic and Social Disadvantage | CVD risk scores | overall | Beta (SE) | 1 | | 1 | Neighborhood disadvantage | 0.22 | - | - | 0.07 |
| [15] | Barber, 2016 | Economic and Social Disadvantage | CVD risk scores | male | Beta (SE) | 1 | | 1 |  | −0.004 | - | - | 0.12 |
|  |  | Economic and Social Disadvantage | CVD risk scores | male | Beta (SE) | 2 | | 1 |  | 0.46 | - | - | 0.23 |
|  |  | Economic and Social Disadvantage | CVD risk scores | female | Beta (SE) | 3 | | 1 |  | 0.16 | - | - | 0.11 |
|  |  | Economic and Social Disadvantage | CVD risk scores | female | Beta (SE) | 4 | | 1 |  | 0.16 | - | - | 0.19 |
|  |  | Economic and Social Disadvantage | CVD risk scores | male | Beta (SE) | 5 | | 1 |  | 0.36 | - | - | 0.12 |
| [16] | Barber, 2016 | Economic and Social Disadvantage | metabolic and inflammatory-related risk factors | overall | Mean (SD) | 1 | | 1 | High | 53.00 | - | - | 15.00 |
|  |  |  |  | overall |  | 1 | | 2 | Medium | 52.20 | - | - | 14.80 |
|  |  |  |  | overall |  | 1 | | 3 | Low | 51.10 | - | - | 13.60 |
|  |  | Economic and Social Disadvantage | metabolic and inflammatory-related risk factors | overall | Mean (SD) | 2 | | 1 | High | 126.40 | - | - | 36.00 |
|  |  |  |  | overall |  | 2 | | 2 | Medium | 126.90 | - | - | 35.80 |
|  |  |  |  | overall |  | 2 | | 3 | Low | 127.70 | - | - | 37.30 |
|  |  | Economic and Social Disadvantage | metabolic and inflammatory-related risk factors | overall | Mean (SD) | 3 | | 1 | High | 101.70 | - | - | 53.60 |
|  |  |  |  | overall |  | 3 | | 2 | Medium | 103.50 | - | - | 57.50 |
|  |  |  |  | overall |  | 3 | | 3 | Low | 97.50 | - | - | 51.60 |
| [17] | Bhopal, 2002 | Economic and Social Disadvantage | glucose metabolism-related risk factors | male | Percentage excess of OR | 1 | | 1 |  | 135.00 | - | - |  |
|  |  | Economic and Social Disadvantage | glucose metabolism-related risk factors | male | Percentage excess of OR | 2 | | 1 |  | 41.00 | - | - |  |
|  |  | Economic and Social Disadvantage | glucose metabolism-related risk factors | male | Percentage excess of OR | 3 | | 1 |  | 66.00 | - | - |  |
|  |  | Economic and Social Disadvantage | glucose metabolism-related risk factors | male | Percentage excess of OR | 4 | | 1 |  | 48.00 | - | - |  |
|  |  | Economic and Social Disadvantage | glucose metabolism-related risk factors | male | Percentage excess of OR | 5 | | 1 |  | -11.00 | - | - |  |
|  |  | Economic and Social Disadvantage | cardiovascular health-related risk factors | male | Mean difference | 6 | | 1 |  | 0.20 | - | - |  |
|  |  | Economic and Social Disadvantage | cardiovascular health-related risk factors | male | Mean difference | 7 | | 1 |  | –0.70 | - | - |  |
|  |  | Economic and Social Disadvantage | cardiovascular health-related risk factors | male | Mean difference | 8 | | 1 |  | 0.90 | - | - |  |
|  |  | Economic and Social Disadvantage | cardiovascular health-related risk factors | male | Mean difference | 9 | | 1 |  | 0.60 | - | - |  |
|  |  | Economic and Social Disadvantage | cardiovascular health-related risk factors | male | Mean difference | 10 | | 1 |  | –1.90 | - | - |  |
|  |  | Economic and Social Disadvantage | cardiovascular health-related risk factors | male | Mean difference | 11 | | 1 |  | –1.00 | - | - |  |
|  |  | Economic and Social Disadvantage | cardiovascular health-related risk factors | male | Mean difference | 12 | | 1 |  | –1.10 | - | - |  |
|  |  | Economic and Social Disadvantage | cardiovascular health-related risk factors | male | Mean difference | 13 | | 1 |  | 2.80 | - | - |  |
|  |  | Economic and Social Disadvantage | cardiovascular health-related risk factors | male | Mean difference | 14 | | 1 |  | –2.40 | - | - |  |
|  |  | Economic and Social Disadvantage | cardiovascular health-related risk factors | male | Mean difference | 15 | | 1 |  | –2.00 | - | - |  |
|  |  | Economic and Social Disadvantage | glucose metabolism-related risk factors | male | Mean difference | 16 | | 1 |  | 0.00 | - | - |  |
|  |  | Economic and Social Disadvantage | glucose metabolism-related risk factors | male | Mean difference | 17 | | 1 |  | 0.40 | - | - |  |
|  |  | Economic and Social Disadvantage | glucose metabolism-related risk factors | male | Mean difference | 18 | | 1 |  | 1.20 | - | - |  |
|  |  | Economic and Social Disadvantage | glucose metabolism-related risk factors | male | Mean difference | 19 | | 1 |  | 0.00 | - | - |  |
|  |  | Economic and Social Disadvantage | glucose metabolism-related risk factors | male | Mean difference | 20 | | 1 |  | 0.20 | - | - |  |
|  |  | Economic and Social Disadvantage | glucose metabolism-related risk factors | male | Mean difference | 21 | | 1 |  | 0.40 | - | - |  |
|  |  | Economic and Social Disadvantage | glucose metabolism-related risk factors | male | Mean difference | 22 | | 1 |  | 0.80 | - | - |  |
|  |  | Economic and Social Disadvantage | glucose metabolism-related risk factors | male | Mean difference | 23 | | 1 |  | 3.00 | - | - |  |
|  |  | Economic and Social Disadvantage | glucose metabolism-related risk factors | male | Mean difference | 24 | | 1 |  | –0.10 | - | - |  |
|  |  | Economic and Social Disadvantage | glucose metabolism-related risk factors | male | Mean difference | 25 | | 1 |  | –0.70 | - | - |  |
|  |  | Economic and Social Disadvantage | metabolic and inflammatory-related risk factors | male | Mean difference | 26 | | 1 |  | –0.17 | - | - |  |
|  |  | Economic and Social Disadvantage | metabolic and inflammatory-related risk factors | male | Mean difference | 27 | | 1 |  | 0.03 | - | - |  |
|  |  | Economic and Social Disadvantage | metabolic and inflammatory-related risk factors | male | Mean difference | 28 | | 1 |  | 0.52 | - | - |  |
|  |  | Economic and Social Disadvantage | metabolic and inflammatory-related risk factors | male | Mean difference | 29 | | 1 |  | –0.22 | - | - |  |
|  |  | Economic and Social Disadvantage | metabolic and inflammatory-related risk factors | male | Mean difference | 30 | | 1 |  | 0.05 | - | - |  |
|  |  | Economic and Social Disadvantage | glucose metabolism-related risk factors | female | Percentage excess of OR | 31 | | 1 |  | 10.00 | - | - |  |
|  |  | Economic and Social Disadvantage | glucose metabolism-related risk factors | female | Percentage excess of OR | 32 | | 1 |  | 4.00 | - | - |  |
|  |  | Economic and Social Disadvantage | glucose metabolism-related risk factors | female | Percentage excess of OR | 33 | | 1 |  | 3.00 | - | - |  |
|  |  | Economic and Social Disadvantage | glucose metabolism-related risk factors | female | Percentage excess of OR | 34 | | 1 |  | 91.00 | - | - |  |
|  |  | Economic and Social Disadvantage | glucose metabolism-related risk factors | female | Percentage excess of OR | 35 | | 1 |  | -81.00 | - | - |  |
|  |  | Economic and Social Disadvantage | cardiovascular health-related risk factors | female | Mean difference | 36 | | 1 |  | 0.10 | - | - |  |
|  |  | Economic and Social Disadvantage | cardiovascular health-related risk factors | female | Mean difference | 37 | | 1 |  | 2.60 | - | - |  |
|  |  | Economic and Social Disadvantage | cardiovascular health-related risk factors | female | Mean difference | 38 | | 1 |  | 5.40 | - | - |  |
|  |  | Economic and Social Disadvantage | cardiovascular health-related risk factors | female | Mean difference | 39 | | 1 |  | 1.60 | - | - |  |
|  |  | Economic and Social Disadvantage | cardiovascular health-related risk factors | female | Mean difference | 40 | | 1 |  | –1.30 | - | - |  |
|  |  | Economic and Social Disadvantage | cardiovascular health-related risk factors | female | Mean difference | 41 | | 1 |  | –1.00 | - | - |  |
|  |  | Economic and Social Disadvantage | cardiovascular health-related risk factors | female | Mean difference | 42 | | 1 |  | 1.00 | - | - |  |
|  |  | Economic and Social Disadvantage | cardiovascular health-related risk factors | female | Mean difference | 43 | | 1 |  | 0.00 | - | - |  |
|  |  | Economic and Social Disadvantage | cardiovascular health-related risk factors | female | Mean difference | 44 | | 1 |  | 1.60 | - | - |  |
|  |  | Economic and Social Disadvantage | cardiovascular health-related risk factors | female | Mean difference | 45 | | 1 |  | 0.80 | - | - |  |
|  |  | Economic and Social Disadvantage | glucose metabolism-related risk factors | female | Mean difference | 46 | | 1 |  | 0.10 | - | - |  |
|  |  | Economic and Social Disadvantage | glucose metabolism-related risk factors | female | Mean difference | 47 | | 1 |  | 0.10 | - | - |  |
|  |  | Economic and Social Disadvantage | glucose metabolism-related risk factors | female | Mean difference | 48 | | 1 |  | 0.30 | - | - |  |
|  |  | Economic and Social Disadvantage | glucose metabolism-related risk factors | female | Mean difference | 49 | | 1 |  | 0.00 | - | - |  |
|  |  | Economic and Social Disadvantage | glucose metabolism-related risk factors | female | Mean difference | 50 | | 1 |  | –0.50 | - | - |  |
|  |  | Economic and Social Disadvantage | glucose metabolism-related risk factors | female | Mean difference | 51 | | 1 |  | 0.30 | - | - |  |
|  |  | Economic and Social Disadvantage | glucose metabolism-related risk factors | female | Mean difference | 52 | | 1 |  | 0.60 | - | - |  |
|  |  | Economic and Social Disadvantage | glucose metabolism-related risk factors | female | Mean difference | 53 | | 1 |  | –0.10 | - | - |  |
|  |  | Economic and Social Disadvantage | glucose metabolism-related risk factors | female | Mean difference | 54 | | 1 |  | 1.60 | - | - |  |
|  |  | Economic and Social Disadvantage | glucose metabolism-related risk factors | female | Mean difference | 55 | | 1 |  | 0.50 | - | - |  |
|  |  | Economic and Social Disadvantage | metabolic and inflammatory-related risk factors | female | Mean difference | 56 | | 1 |  | 0.08 | - | - |  |
|  |  | Economic and Social Disadvantage | metabolic and inflammatory-related risk factors | female | Mean difference | 57 | | 1 |  | –0.02 | - | - |  |
|  |  | Economic and Social Disadvantage | metabolic and inflammatory-related risk factors | female | Mean difference | 58 | | 1 |  | 0.27 | - | - |  |
|  |  | Economic and Social Disadvantage | metabolic and inflammatory-related risk factors | female | Mean difference | 59 | | 1 |  | –0.41 | - | - |  |
|  |  | Economic and Social Disadvantage | metabolic and inflammatory-related risk factors | female | Mean difference | 60 | | 1 |  | 0.33 | - | - |  |
| [18] | Bird, 2010 | Economic and Social Disadvantage | CVD risk scores | overall | Beta (95%CI) | 1 | | 1 |  | -0.05 | -0.08 | -0.01 |  |
|  |  | Economic and Social Disadvantage | CVD risk scores | overall | Beta (95%CI) | 2 | | 1 |  | 0.00 | -0.02 | 0.01 |  |
|  |  | Economic and Social Disadvantage | CVD risk scores | overall | Beta (95%CI) | 3 | | 1 |  | -0.03 | -0.05 | -0.01 |  |
|  |  | Economic and Social Disadvantage | CVD risk scores | overall | Beta (95%CI) | 4 | | 1 |  | -0.02 | -0.03 | 0.00 |  |
| [19] | Bland, 2000 | Social Relationships and Norms | cardiovascular health-related risk factors | male | Beta | 1 | | 1 |  | 125.00 | - | - |  |
|  |  | Social Relationships and Norms | cardiovascular health-related risk factors | male | Beta | 2 | | 1 |  | 127.10 | - | - |  |
|  |  | Social Relationships and Norms | cardiovascular health-related risk factors | male | Beta | 3 | | 1 |  | 85.60 | - | - |  |
|  |  | Social Relationships and Norms | cardiovascular health-related risk factors | male | Beta | 4 | | 1 |  | 86.34 | - | - |  |
|  |  | Social Relationships and Norms | metabolic and inflammatory-related risk factors | male | Beta | 5 | | 1 |  | 221.10 | - | - |  |
|  |  | Social Relationships and Norms | metabolic and inflammatory-related risk factors | male | Beta | 6 | | 1 |  | 215.80 | - | - |  |
| [20] | Bland, 1991 | Social Relationships and Norms | cardiovascular health-related risk factors | male | Mean | 1 | | 1 | Low (0-5) | 145.10 | - | - |  |
|  |  |  |  | male |  | 1 | | 2 | Medium (6) | 141.20 | - | - |  |
|  |  |  |  | male |  | 1 | | 3 | High (7-10) | 138.40 | - | - |  |
|  |  | Social Relationships and Norms | cardiovascular health-related risk factors | male | Beta | 2 | | 1 |  | -1.90 | - | - |  |
|  |  | Social Relationships and Norms | cardiovascular health-related risk factors | male | Mean | 3 | | 1 | Low (0-5) | 90.20 | - | - |  |
|  |  |  |  | male |  | 3 | | 2 | Medium (6) | 86.70 | - | - |  |
|  |  |  |  | male |  | 3 | | 3 | High (7-10) | 86.30 | - | - |  |
|  |  | Social Relationships and Norms | cardiovascular health-related risk factors | male | Beta | 4 | | 1 |  | -0.80 | - | - |  |
|  |  | Social Relationships and Norms | cardiovascular health-related risk factors | female | Mean | 5 | | 1 | Low (0-5) | 138.50 | - | - |  |
|  |  |  |  | female |  | 5 | | 2 | Medium (6) | 131.50 | - | - |  |
|  |  |  |  | female |  | 5 | | 3 | High (7-10) | 132.50 | - | - |  |
|  |  | Social Relationships and Norms | cardiovascular health-related risk factors | female | Beta | 6 | | 1 |  | -1.60 | - | - |  |
|  |  | Social Relationships and Norms | cardiovascular health-related risk factors | female | Mean | 7 | | 1 | Low (0-5) | 83.60 | - | - |  |
|  |  |  |  | female |  | 7 | | 2 | Medium (6) | 80.30 | - | - |  |
|  |  |  |  | female |  | 7 | | 3 | High (7-10) | 82.10 | - | - |  |
|  |  | Social Relationships and Norms | cardiovascular health-related risk factors | female | Beta | 8 | | 1 |  | -0.60 | - | - |  |
|  |  | Civic Participation and Engagement | cardiovascular health-related risk factors | male | Mean | 9 | | 1 | None | 143.90 | - | - |  |
|  |  |  |  | male |  | 9 | | 2 | Medium (1 or more clubs, 12 or less meetings/year) | 142.90 | - | - |  |
|  |  |  |  | male |  | 9 | | 3 | High (1 or more clubs, more than 12 meetings/year) | 138.10 | - | - |  |
|  |  | Civic Participation and Engagement | cardiovascular health-related risk factors | male | Beta | 10 | | 1 |  | -2.90 | - | - |  |
|  |  | Civic Participation and Engagement | cardiovascular health-related risk factors | male | Mean | 11 | | 1 | no | 139.90 | - | - |  |
|  |  |  |  | male |  | 11 | | 2 | yes | 140.20 | - | - |  |
|  |  | Civic Participation and Engagement | cardiovascular health-related risk factors | male | Ratio for differences in means | 12 | | 1 |  | 0.00 | - | - |  |
|  |  | Civic Participation and Engagement | cardiovascular health-related risk factors | male | Mean | 13 | | 1 | None | 88.50 | - | - |  |
|  |  |  |  | male |  | 13 | | 2 | Medium (1 or more clubs, 12 or less meetings/year) | 87.90 | - | - |  |
|  |  |  |  | male |  | 13 | | 3 | High (1 or more clubs, more than 12 meetings/year) | 86.30 | - | - |  |
|  |  | Civic Participation and Engagement | cardiovascular health-related risk factors | male | Beta | 14 | | 1 |  | -1.10 | - | - |  |
|  |  | Civic Participation and Engagement | cardiovascular health-related risk factors | male | Mean | 15 | | 1 | no | 88.20 | - | - |  |
|  |  |  |  | male |  | 15 | | 2 | yes | 87.60 | - | - |  |
|  |  | Civic Participation and Engagement | cardiovascular health-related risk factors | male | Ratio for differences in means | 16 | | 1 |  | 0.70 | - | - |  |
|  |  | Civic Participation and Engagement | cardiovascular health-related risk factors | female | Mean | 17 | | 1 | None | 135.90 | - | - |  |
|  |  |  |  | female |  | 17 | | 2 | Medium (1 or more clubs, 12 or less meetings/year) | 133.50 | - | - |  |
|  |  |  |  | female |  | 17 | | 3 | High (1 or more clubs, more than 12 meetings/year) | 130.90 | - | - |  |
|  |  | Civic Participation and Engagement | cardiovascular health-related risk factors | female | Beta | 18 | | 1 |  | -1.60 | - | - |  |
|  |  | Civic Participation and Engagement | cardiovascular health-related risk factors | female | Mean | 19 | | 1 | No | 134.80 | - | - |  |
|  |  |  |  | female |  | 19 | | 2 | yes | 134.60 | - | - |  |
|  |  | Civic Participation and Engagement | cardiovascular health-related risk factors | female | Ratio for differences in means | 20 | | 1 |  | 0.00 | - | - |  |
|  |  | Civic Participation and Engagement | cardiovascular health-related risk factors | female | Mean | 21 | | 1 | None | 82.90 | - | - |  |
|  |  |  |  | female |  | 21 | | 2 | Medium (1 or more clubs, 12 or less meetings/year) | 81.80 | - | - |  |
|  |  |  |  | female |  | 21 | | 3 | High (1 or more clubs, more than 12 meetings/year) | 81.30 | - | - |  |
|  |  | Civic Participation and Engagement | cardiovascular health-related risk factors | female | Beta | 22 | | 1 |  | -0.80 | - | - |  |
|  |  | Civic Participation and Engagement | cardiovascular health-related risk factors | female | Mean | 23 | | 1 | No | 82.70 | - | - |  |
|  |  |  |  | female |  | 23 | | 2 | Yes | 82.10 | - | - |  |
|  |  | Civic Participation and Engagement | cardiovascular health-related risk factors | female | Ratio for differences in means | 24 | | 1 |  | 0.30 | - | - |  |
| [21] | Boylan, 2017 | Economic and Social Disadvantage | CVD risk scores | overall | Beta (SE) | 1 | | 1 |  | 0.06 | - | - | 0.07 |
| [22] | Breckenkamp, 2007 | Economic and Social Disadvantage | cardiovascular health-related risk factors | male | Beta (SE) | 1 | | 1 |  | -0.33 | - | - | 0.20 |
|  |  | Economic and Social Disadvantage | cardiovascular health-related risk factors | male | Beta (SE) | 2 | | 1 |  | -0.43 | - | - | 0.10 |
|  |  | Economic and Social Disadvantage | metabolic and inflammatory-related risk factors | male | Beta (SE) | 3 | | 1 |  | -0.58 | - | - | 0.21 |
|  |  | Economic and Social Disadvantage | cardiovascular health-related risk factors | female | Beta | 4 | | 1 |  | na | na | na |  |
|  |  | Economic and Social Disadvantage | cardiovascular health-related risk factors | female | Beta (SE) | 5 | | 1 |  | -0.29 | - | - | 0.14 |
|  |  | Economic and Social Disadvantage | metabolic and inflammatory-related risk factors | female | Beta (SE) | 6 | | 1 |  | -0.39 | - | - | 0.14 |
| [23] | Browning, 2012 | Crime and Safety | metabolic and inflammatory-related risk factors | overall | coefficient for piecewise linear slope (splines) | 1 | | 1 |  | 0.04 | - | - | 0.02 |
|  |  | Crime and Safety | metabolic and inflammatory-related risk factors | overall | coefficient for piecewise linear slope (splines) | 2 | | 1 |  | 0.04 | - | - | 0.01 |
| [24] | Bu, 2021 | Civic Participation and Engagement | CVD risk scores | overall | RR (95%CI) | 1 | | 1 | low risk (reference) | 1.00 | - | - |  |
|  |  |  |  | overall |  | 1 | | 2 | high risk | 2.40 | 1.96 | 2.94 |  |
|  |  |  |  | overall |  | 1 | | 3 | clinical risk | 0.84 | 0.72 | 0.98 |  |
|  |  |  |  | overall |  | 1 | | 4 | lifestyle risk | 1.36 | 1.10 | 1.67 |  |
|  |  | Civic Participation and Engagement | CVD risk scores | overall | RR (95%CI) | 2 | | 1 | low risk (reference) | 1.00 | - | - |  |
|  |  |  |  | overall |  | 2 | | 2 | high risk | 1.15 | 0.77 | 1.72 |  |
|  |  |  |  | overall |  | 2 | | 3 | clinical risk | 1.19 | 0.87 | 1.65 |  |
|  |  |  |  | overall |  | 2 | | 4 | lifestyle risk | 1.86 | 1.18 | 2.91 |  |
|  |  | Civic Participation and Engagement | CVD risk scores | overall | RR (95%CI) | 3 | | 1 | low risk (reference) | 1.00 | - | - |  |
|  |  |  |  | overall |  | 3 | | 2 | high risk | 0.78 | 0.66 | 0.92 |  |
|  |  |  |  | overall |  | 3 | | 3 | clinical risk | 0.95 | 0.84 | 1.07 |  |
|  |  |  |  | overall |  | 3 | | 4 | lifestyle risk | 1.13 | 0.94 | 1.35 |  |
|  |  | Civic Participation and Engagement | CVD risk scores | overall | RR (95%CI) | 4 | | 1 | low risk (reference) | 1.00 | - | - |  |
|  |  |  |  | overall |  | 4 | | 2 | high risk | 3.03 | 2.40 | 3.83 |  |
|  |  |  |  | overall |  | 4 | | 3 | clinical risk | 1.22 | 1.04 | 1.43 |  |
|  |  |  |  | overall |  | 4 | | 4 | lifestyle risk | 3.70 | 2.91 | 4.70 |  |
| [25] | Carels, 1998 | Social Relationships and Norms | cardiovascular health-related risk factors | overall | Mean | 1 | | 1 |  | 5.73 | - | - |  |
|  |  | Social Relationships and Norms | cardiovascular health-related risk factors | overall | Mean | 2 | | 1 |  | 0.84 | - | - |  |
|  |  | Social Relationships and Norms | cardiovascular health-related risk factors | overall | Mean | 3 | | 1 |  | 0.22 | - | - |  |
|  |  | Social Relationships and Norms | cardiovascular health-related risk factors | overall | Mean (SE) | 4 | | 1 | normotensive | 5.43 | - | - | 1.17 |
|  |  | Social Relationships and Norms | cardiovascular health-related risk factors | overall |  | 5 | | 1 | borderline hypertensive | 5.01 | - | - | 0.79 |
|  |  | Social Relationships and Norms | cardiovascular health-related risk factors | overall | Mean (SE) | 6 | | 1 | borderline hypertension | 1.99 | - | - | 1.37 |
|  |  | Social Relationships and Norms | cardiovascular health-related risk factors | overall |  | 7 | | 1 | normotensive | 1.75 | - | - | 1.35 |
|  |  | Social Relationships and Norms | cardiovascular health-related risk factors | overall | Mean (SE) | 8 | | 1 | borderline hypertensive | 3.39 | - | - | 1.89 |
|  |  | Social Relationships and Norms | cardiovascular health-related risk factors | overall |  | 9 | | 1 | normotensive | 3.23 | - | - | 1.89 |
|  |  | Social Relationships and Norms | cardiovascular health-related risk factors | overall | Mean | 10 | | 1 |  | 7.08 | - | - |  |
|  |  | Social Relationships and Norms | cardiovascular health-related risk factors | overall | Mean | 11 | | 1 |  | 9.60 | - | - |  |
| [26] | Carson, 2007 | Economic and Social Disadvantage | cardiovascular health-related risk factors | male | Beta (95%CI) | 1 | | 1 | lowest tertile | 4.50 | -15.50 | 24.40 |  |
|  |  |  |  | male |  | 1 | | 2 | middle tertile | -2.70 | -17.60 | 12.10 |  |
|  |  |  |  | male |  | 1 | | 3 | highest tertile (reference) | - | - | - |  |
|  |  | Economic and Social Disadvantage | cardiovascular health-related risk factors | female | Beta (95%CI) | 2 | | 1 | lowest tertile | 4.00 | -6.50 | 14.60 |  |
|  |  |  |  | female |  | 2 | | 2 | middle tertile | 0.20 | -10.20 | 10.60 |  |
|  |  |  |  | female |  | 2 | | 3 | highest tertile (reference) | - | - | - |  |
|  |  | Economic and Social Disadvantage | cardiovascular health-related risk factors | male | Beta (95%CI) | 3 | | 1 | lowest tertile | 7.00 | -20.90 | 34.90 |  |
|  |  |  |  | male |  | 3 | | 2 | middle tertile | -3.20 | -28.90 | 22.50 |  |
|  |  |  |  | male |  | 3 | | 3 | highest tertile (reference) | - | - | - |  |
|  |  | Economic and Social Disadvantage | cardiovascular health-related risk factors | female | Beta (95%CI) | 4 | | 1 | lowest tertile | 1.30 | -15.60 | 18.10 |  |
|  |  |  |  | female |  | 4 | | 2 | middle tertile | 6.10 | -12.80 | 25.00 |  |
|  |  |  |  | female |  | 4 | | 3 | highest tertile (reference) | - | - | - |  |
|  |  | Economic and Social Disadvantage | cardiovascular health-related risk factors | male | OR (95%CI) | 5 | | 1 | lowest tertile | 1.14 | 0.54 | 2.40 |  |
|  |  |  |  | male |  | 5 | | 2 | middle tertile | 0.93 | 0.40 | 2.17 |  |
|  |  |  |  | male |  | 5 | | 3 | highest tertile (reference) | 1.00 | - | - |  |
|  |  | Economic and Social Disadvantage | cardiovascular health-related risk factors | female | OR (95%CI) | 6 | | 1 | lowest tertile | 1.19 | 0.81 | 1.76 |  |
|  |  |  |  | female |  | 6 | | 2 | middle tertile | 0.94 | 0.63 | 1.40 |  |
|  |  |  |  | female |  | 6 | | 3 | highest tertile (reference) | 1.00 | - | - |  |
|  |  | Economic and Social Disadvantage | cardiovascular health-related risk factors | male | OR (95%CI) | 7 | | 1 | lowest tertile | 2.91 | 0.80 | 10.65 |  |
|  |  |  |  | male |  | 7 | | 2 | middle tertile | 1.60 | 0.32 | 7.87 |  |
|  |  |  |  | male |  | 7 | | 3 | highest tertile (reference) | 1.00 | - | - |  |
|  |  | Economic and Social Disadvantage | cardiovascular health-related risk factors | female | OR (95%CI) | 8 | | 1 | lowest tertile | 1.00 | 0.50 | 2.00 |  |
|  |  |  |  | female |  | 8 | | 2 | middle tertile | 0.81 | 0.37 | 1.80 |  |
|  |  |  |  | female |  | 8 | | 3 | highest tertile (reference) | 1.00 | - | - |  |
| [27] | Caspi, 2006 | Social Relationships and Norms | CVD risk scores | overall | RR (95%CI) | 1 | | 1 |  | 2.58 | 1.46 | 4.56 |  |
| [28] | Cathorall, 2015 | Economic and Social Disadvantage | cardiovascular health-related risk factors | overall | Beta (SE) | 1 | | 1 |  | 0.12 | - | - | 0.05 |
|  |  | Economic and Social Disadvantage | cardiovascular health-related risk factors | overall | Beta (SE) | 2 | | 1 |  | -0.03 | - | - | 0.03 |
| [29] | Chaparro, 2018 | Economic and Social Disadvantage | cardiovascular health-related risk factors | overall | Beta (95%CI) | 1 | | 1 |  | 0.18 | 0.10 | 0.27 |  |
|  |  | Economic and Social Disadvantage | metabolic and inflammatory-related risk factors | overall | Beta (95%CI) | 2 | | 1 |  | 0.06 | 0.04 | 0.07 |  |
| [30] | Chaix, 2010 | Economic and Social Disadvantage | cardiovascular health-related risk factors | overall | Beta | 1 | | 1 | high (reference) | - | - | - |  |
|  |  |  |  | overall |  | 1 | | 2 | mid-high | 0.45 | -0.71 | 1.60 |  |
|  |  |  |  | overall |  | 1 | | 3 | mid-low | 1.29 | 0.12 | 2.47 |  |
|  |  |  |  | overall |  | 1 | | 4 | low | 2.39 | 1.16 | 3.62 |  |
| [31] | Chaix, 2008 | Economic and Social Disadvantage | cardiovascular health-related risk factors | male | Beta | 1 | | 1 | high (reference) | - | - | - |  |
|  |  |  |  | male |  | 1 | | 2 | mid-high | 0.20 | -0.90 | 1.30 |  |
|  |  |  |  | male |  | 1 | | 3 | mid-low | 0.10 | -1.00 | 1.20 |  |
|  |  |  |  | male |  | 1 | | 4 | low | 1.50 | 0.30 | 2.70 |  |
| [32] | Chichlowska, 2008 | Economic and Social Disadvantage | CVD risk scores | female | PR (95%CI) | 1 | | 1 | high (reference) | 1.00 | - | - |  |
|  |  |  |  | female |  | 1 | | 2 | medium | 1.25 | 1.03 | 1.53 |  |
|  |  |  |  | female |  | 1 | | 3 | low | 1.20 | 1.04 | 1.40 |  |
|  |  | Economic and Social Disadvantage | CVD risk scores | female | PR (95%CI) | 2 | | 1 | high (reference) | 1.00 | - | - |  |
|  |  |  |  | female |  | 2 | | 2 | medium | 1.14 | 1.00 | 1.30 |  |
|  |  |  |  | female |  | 2 | | 3 | low | 1.17 | 1.00 | 1.37 |  |
|  |  | Economic and Social Disadvantage | CVD risk scores | male | PR (95%CI) | 3 | | 1 | high (reference) | 1.00 | - | - |  |
|  |  |  |  | male |  | 3 | | 2 | medium | 1.28 | 1.01 | 1.62 |  |
|  |  |  |  | male |  | 3 | | 3 | low | 1.16 | 0.89 | 1.50 |  |
|  |  | Economic and Social Disadvantage | CVD risk scores | male | PR (95%CI) | 4 | | 1 | high (reference) | 1.00 | - | - |  |
|  |  |  |  | male |  | 4 | | 2 | medium | 0.99 | 0.88 | 1.12 |  |
|  |  |  |  | male |  | 4 | | 3 | low | 0.98 | 0.84 | 1.15 |  |
| [33] | Clark, 2013 | Economic and Social Disadvantage | CVD risk scores | female | PR (95%CI) | 1 | | 1 | Living in most advantaged neighborhoods (reference) | 1.00 | - | - |  |
|  |  |  |  | female |  | 1 | | 2 | Living in most disadvantaged neighborhoods | 1.13 | 1.01 | 1.27 |  |
|  |  | Crime and Safety | CVD risk scores | female | PR (95%CI) | 2 | | 1 | does not perceives neighborhood as unsafe reference) | 1.00 | - | - |  |
|  |  |  |  | female |  | 2 | | 2 | Perceives neighborhood as unsafe | 1.00 | 0.88 | 1.14 |  |
|  |  | Economic and Social Disadvantage | CVD risk scores | male | PR (95%CI) | 3 | | 1 | Living in most advantaged neighborhoods (reference) | 1.00 | - | - |  |
|  |  |  |  | male |  | 3 | | 2 | Living in most disadvantaged neighborhoods | 0.96 | 0.79 | 1.18 |  |
|  |  | Crime and Safety | CVD risk scores | male | PR (95%CI) | 4 | | 1 | Does not perceives neighborhood as unsafe reference) | 1.00 | - | - |  |
|  |  |  |  | male |  | 4 | | 2 | Perceives neighborhood as unsafe | 1.16 | 0.98 | 1.38 |  |
| [34] | Clark, 2012 | Economic and Social Disadvantage | metabolic and inflammatory-related risk factors | female | Beta (95%CI) | 1 | | 1 |  | -0.05 | -0.07 | -0.03 |  |
|  |  | Economic and Social Disadvantage | metabolic and inflammatory-related risk factors | female | Beta (95%CI) | 2 | | 1 |  | -0.02 | -0.03 | 0.00 |  |
|  |  | Economic and Social Disadvantage | metabolic and inflammatory-related risk factors | female | Beta (95%CI) | 3 | | 1 |  | -0.01 | -0.02 | 0.01 |  |
|  |  | Economic and Social Disadvantage | metabolic and inflammatory-related risk factors | female | Beta (95%CI) | 4 | | 1 |  | 0.04 | 0.01 | 0.06 |  |
|  |  | Economic and Social Disadvantage | metabolic and inflammatory-related risk factors | female | Beta (95%CI) | 5 | | 1 |  | 0.02 | 0.00 | 0.03 |  |
|  |  | Economic and Social Disadvantage | metabolic and inflammatory-related risk factors | female | Beta (95%CI) | 6 | | 1 |  | 0.03 | 0.02 | 0.05 |  |
|  |  | Economic and Social Disadvantage | metabolic and inflammatory-related risk factors | female | Beta (95%CI) | 7 | | 1 |  | 0.05 | 0.03 | 0.07 |  |
|  |  | Economic and Social Disadvantage | metabolic and inflammatory-related risk factors | female | Beta (95%CI) | 8 | | 1 |  | 0.02 | 0.00 | 0.03 |  |
|  |  | Economic and Social Disadvantage | metabolic and inflammatory-related risk factors | female | Beta (95%CI) | 9 | | 1 |  | 0.01 | 0.00 | 0.03 |  |
| [35] | Claudel, 2018 | Economic and Social Disadvantage | cardiovascular health-related risk factors | overall | Beta | 1 | | 1 | Low neighborhood disadvantage (reference) | - | - | - |  |
|  |  |  |  | overall |  | 1 | | 2 | Medium neighborhood disadvantage | 4.81 | - | - | 1.39 |
|  |  |  |  | overall |  | 1 | | 3 | High neighborhood disadvantage | 7.64 | - | - | 1.55 |
|  |  | Economic and Social Disadvantage | cardiovascular health-related risk factors | overall | Beta | 2 | | 1 | Low neighborhood disadvantage (reference) | - | - | - | - |
|  |  |  |  | overall |  | 2 | | 2 | Medium neighborhood disadvantage | 2.61 | - | - | 0.71 |
|  |  |  |  | overall |  | 2 | | 3 | High neighborhood disadvantage | 4.64 | - | - | 0.78 |
| [36] | Climie, 2019 | Economic and Social Disadvantage | cardiovascular health-related risk factors | male | Beta | 1 | | 1 | tertile 1 (reference) | - | - | - |  |
|  |  |  |  | male |  | 1 | | 2 | tertile 2 | 0.05 | −0.05 | 0.15 |  |
|  |  |  |  | male |  | 1 | | 3 | tertile 3 | 0.10 | 0.01 | 0.19 |  |
|  |  | Economic and Social Disadvantage | cardiovascular health-related risk factors | female | Beta | 2 | | 1 | tertile 1 (reference) | - | - | - |  |
|  |  |  |  | female |  | 2 | | 2 | tertile 2 | −0.04 | −0.15 | 0.07 |  |
|  |  |  |  | female |  | 2 | | 3 | tertile 3 | 0.01 | −0.10 | 0.13 |  |
| [37] | Cohn, 2017 | Discrimination and Segregation | CVD risk scores | overall | Beta (SE) | 1 | | 1 |  | 0.02 | 0.00 | 0.05 | 0.01 |
| [38] | Coulon, 2016 | Crime and Safety | cardiovascular health-related risk factors | overall | Beta (SE) | 1 | | 1 |  | 2.40 | - | - | 1.56 |
|  |  | Economic and Social Disadvantage | cardiovascular health-related risk factors | overall | Beta (SE) | 2 | | 1 |  | 13.10 | - | - | 6.34 |
|  |  | Crime and Safety | cardiovascular health-related risk factors | overall | Beta (SE) | 3 | | 1 |  | 0.24 | - | - | 1.00 |
|  |  | Economic and Social Disadvantage | cardiovascular health-related risk factors | overall | Beta (SE) | 4 | | 1 |  | 11.48 | - | - | 4.08 |
| [39] | Coulon, 2016 | Economic and Social Disadvantage | metabolic and inflammatory-related risk factors | overall | Beta (SE) | 1 | | 1 |  | −0.05 | - | - | 0.05 |
|  |  | Economic and Social Disadvantage | cardiovascular health-related risk factors | overall | Beta (SE) | 2 | | 1 |  | -0.79 | - | - | 0.36 |
|  |  | Economic and Social Disadvantage | cardiovascular health-related risk factors | overall | Beta (SE) | 3 | | 1 |  | −0.30 | - | - | 0.19 |
| [40] | Cozier, 2016 | Economic and Social Disadvantage | metabolic and inflammatory-related risk factors | female | Mean (95%CI) | 1 | | 1 | quantile 1 | 2.60 | 2.07 | 3.28 |  |
|  |  |  |  | female |  | 1 | | 2 | quantile 2 | 2.07 | 1.73 | 2.47 |  |
|  |  |  |  | female |  | 1 | | 3 | quantile 3 | 2.43 | 2.02 | 2.93 |  |
|  |  |  |  | female |  | 1 | | 4 | quantile 4 | 1.82 | 1.41 | 2.35 |  |
|  |  |  |  | female |  | 1 | | 5 | quantile 5 | 1.57 | 1.11 | 2.22 |  |
|  |  | Economic and Social Disadvantage | metabolic and inflammatory-related risk factors | female | Mean difference | 2 | | 1 |  | 1.03 | - | - |  |
|  |  | Economic and Social Disadvantage | metabolic and inflammatory-related risk factors | female | Mean (95%CI) | 3 | | 1 | quantile 1 | 6.07 | 5.94 | 6.21 |  |
|  |  |  |  | female |  | 3 | | 2 | quantile 2 | 5.98 | 5.87 | 6.09 |  |
|  |  |  |  | female |  | 3 | | 3 | quantile 3 | 5.96 | 5.86 | 6.07 |  |
|  |  |  |  | female |  | 3 | | 4 | quantile 4 | 6.04 | 5.88 | 6.20 |  |
|  |  |  |  | female |  | 3 | | 5 | quantile 5 | 5.78 | 5.61 | 5.94 |  |
|  |  | Economic and Social Disadvantage | metabolic and inflammatory-related risk factors | female | Mean difference | 4 | | 1 |  | 0.29 | - | - |  |
|  |  | Economic and Social Disadvantage | metabolic and inflammatory-related risk factors | female | Mean (95%CI) | 5 | | 1 | quantile 1 | 59.80 | 56.80 | 62.90 |  |
|  |  |  |  | female |  | 5 | | 2 | quantile 2 | 61.70 | 59.10 | 64.40 |  |
|  |  |  |  | female |  | 5 | | 3 | quantile 3 | 61.90 | 59.00 | 64.90 |  |
|  |  |  |  | female |  | 5 | | 4 | quantile 4 | 60.30 | 57.50 | 63.20 |  |
|  |  |  |  | female |  | 5 | | 5 | quantile 5 | 65.40 | 60.70 | 70.60 |  |
|  |  | Economic and Social Disadvantage | metabolic and inflammatory-related risk factors | female | Mean difference | 6 | | 1 |  | 5.60 | - | - |  |
| [41] | Creaven, 2013 | Social Relationships and Norms | cardiovascular health-related risk factors | female | Beta (SE) | 1 | | 1 |  | 0.02 | - | - | 0.09 |
|  |  | Social Relationships and Norms | cardiovascular health-related risk factors | female | Beta (SE) | 2 | | 1 |  | -0.19 | - | - | 0.19 |
|  |  | Social Relationships and Norms | cardiovascular health-related risk factors | female | Beta (SE) | 3 | | 1 |  | 0.00 | - | - | 0.07 |
|  |  | Social Relationships and Norms | cardiovascular health-related risk factors | female | Beta (SE) | 4 | | 1 |  | -0.09 | - | - | 0.15 |
|  |  | Social Relationships and Norms | cardiovascular health-related risk factors | female | Beta (SE) | 5 | | 1 |  | -0.22 | - | - | 0.09 |
|  |  | Social Relationships and Norms | cardiovascular health-related risk factors | female | Beta (SE) | 6 | | 1 |  | 0.06 | - | - | 0.19 |
| [42] | Cross, 2019 | Economic and Social Disadvantage | glucose metabolism-related risk factors | overall | OR (95%CI) | 1 | | 1 | quantile 1 | 1.74 | 1.58 | 1.93 |  |
|  |  |  |  | overall |  | 1 | | 2 | quantile 2 | 1.48 | 1.33 | 1.65 |  |
|  |  |  |  | overall |  | 1 | | 3 | quantile 3 | 1.34 | 1.20 | 1.49 |  |
|  |  |  |  | overall |  | 1 | | 4 | quantile 4 | 1.22 | 1.09 | 1.37 |  |
|  |  |  |  | overall |  | 1 | | 5 | quantile 5 (reference) | 1.00 | - | - |  |
|  |  | Economic and Social Disadvantage | glucose metabolism-related risk factors | overall | OR (95%CI) | 2 | | 1 | quantile 1 | 1.85 | 1.65 | 2.08 |  |
|  |  |  |  | overall |  | 2 | | 2 | quantile 2 | 1.53 | 1.35 | 1.73 |  |
|  |  |  |  | overall |  | 2 | | 3 | quantile 3 | 1.40 | 1.23 | 1.59 |  |
|  |  |  |  | overall |  | 2 | | 4 | quantile 4 | 1.25 | 1.10 | 1.43 |  |
|  |  |  |  | overall |  | 2 | | 5 | quantile 5 (reference) | 1.00 | - | - |  |
| [43] | Cubbin, 2005 | Economic and Social Disadvantage | CVD risk scores | overall | Beta (95%CI) | 1 | | 1 | high | 0.19 | -0.82 | 0.45 |  |
|  |  |  |  | overall |  | 1 | | 2 | moderate (reference) | - | - | - |  |
|  |  |  |  | overall |  | 1 | | 3 | low | 0.35 | -0.78 | 0.09 |  |
| [44] | De Moraes, 2019 | Economic and Social Disadvantage | CVD risk scores | male | PR (95%CI) | 1 | | 1 | low | 1.17 | 0.99 | 1.38 |  |
|  |  |  |  | male |  | 1 | | 2 | medium | 1.16 | 0.99 | 1.36 |  |
|  |  |  |  | male |  | 1 | | 3 | high (reference) | 1.00 | - | - |  |
|  |  | Economic and Social Disadvantage | CVD risk scores | female | PR (95%CI) | 2 | | 1 | low | 1.61 | 1.37 | 1.89 |  |
|  |  |  |  | female |  | 2 | | 2 | medium | 1.44 | 1.22 | 1.69 |  |
|  |  |  |  | female |  | 2 | | 3 | high (reference) | 1.00 | - | - |  |
| [45] | Deans, 2009 | Economic and Social Disadvantage | cardiovascular health-related risk factors | overall | Mean difference (95%CI) | 1 | | 1 |  | −0.07 | −0.12 | −0.02 |  |
| [46] | Diez Roux, 2002 | Economic and Social Disadvantage | cardiovascular health-related risk factors | overall | Beta (SE) | 1 | | 1 | lowest | 5.60 | - | - | 0.20 |
|  |  |  |  | overall |  | 1 | | 2 | middle | 5.50 | - | - | 0.20 |
|  |  |  |  | overall |  | 1 | | 3 | highest | 5.30 | - | - | 0.20 |
|  |  | Economic and Social Disadvantage | cardiovascular health-related risk factors | overall | Mean change (SE) | 2 | | 1 | lowest | 5.50 | - | - | 0.50 |
|  |  |  |  | overall |  | 2 | | 2 | middle | 6.30 | - | - | 0.50 |
|  |  |  |  | overall |  | 2 | | 3 | highest | 6.40 | - | - | 0.50 |
|  |  | Economic and Social Disadvantage | cardiovascular health-related risk factors | overall | Mean change (SE) | 3 | | 1 | lowest | 0.50 | - | - | 0.20 |
|  |  |  |  | overall |  | 3 | | 2 | middle | 0.80 | - | - | 0.20 |
|  |  |  |  | overall |  | 3 | | 3 | highest | 1.10 | - | - | 0.20 |
|  |  | Economic and Social Disadvantage | cardiovascular health-related risk factors | overall | Mean change (SE) | 4 | | 1 | lowest | -0.80 | - | - | 0.10 |
|  |  |  |  | overall |  | 4 | | 2 | middle | -0.40 | - | - | 0.10 |
|  |  |  |  | overall |  | 4 | | 3 | highest | 0.10 | - | - | 0.10 |
| [47] | Diez Roux, 2002 | Economic and Social Disadvantage | CVD risk scores | male | Mean difference (SE) | 1 | | 1 | Q1 | 0.14 | - | - | 0.10 |
|  |  |  |  | male |  | 1 | | 2 | Q2 | -0.06 | - | - | 0.09 |
|  |  |  |  | male |  | 1 | | 3 | Q3 | 0.01 | - | - | 0.09 |
|  |  |  |  | male |  | 1 | | 4 | Q4 (reference) | - | - | - |  |
|  |  | Economic and Social Disadvantage | CVD risk scores | male | Mean difference (SE) | 2 | | 1 |  | -0.02 | - | - | 0.01 |
|  |  | Economic and Social Disadvantage | CVD risk scores | female | Mean difference (SE) | 3 | | 1 | Q1 | 0.44 | - | - | 0.10 |
|  |  |  |  | female |  | 3 | | 2 | Q2 | 0.25 | - | - | 0.09 |
|  |  |  |  | female |  | 3 | | 3 | Q3 | 0.16 | - | - | 0.08 |
|  |  |  |  | female |  | 3 | | 4 | Q4 (reference) | - | - | - |  |
|  |  | Economic and Social Disadvantage | CVD risk scores | female | Mean difference (SE) | 4 | | 1 |  | -0.04 | - | - | 0.01 |
|  |  | Economic and Social Disadvantage | CVD risk scores | male | Mean difference (SE) | 5 | | 1 | Q1 | 0.45 | - | - | 0.22 |
|  |  |  |  | male |  | 5 | | 2 | Q2 | 0.41 | - | - | 0.21 |
|  |  |  |  | male |  | 5 | | 3 | Q3 | 0.00 | - | - | 0.23 |
|  |  |  |  | male |  | 5 | | 4 | Q4 (reference) | - | - | - |  |
|  |  | Economic and Social Disadvantage | CVD risk scores | male | Mean difference (SE) | 6 | | 1 |  | 0.05 | - | - | 0.02 |
|  |  | Economic and Social Disadvantage | CVD risk scores | male | Mean difference (SE) | 7 | | 1 | Q1 | 0.36 | - | - | 0.33 |
|  |  |  |  | male |  | 7 | | 2 | Q2 | 0.28 | - | - | 0.29 |
|  |  |  |  | male |  | 7 | | 3 | Q3 | 0.18 | - | - | 0.25 |
|  |  |  |  | male |  | 7 | | 4 | Q4 (reference) | - | - | - |  |
|  |  | Economic and Social Disadvantage | CVD risk scores | male | Mean difference (SE) | 8 | | 1 |  | -0.05 | - | - | 0.02 |
|  |  | Economic and Social Disadvantage | CVD risk scores | female | Mean difference (SE) | 9 | | 1 | Q1 | 0.09 | - | - | 0.18 |
|  |  |  |  | female |  | 9 | | 2 | Q2 | 0.07 | - | - | 0.19 |
|  |  |  |  | female |  | 9 | | 3 | Q3 | 0.26 | - | - | 0.19 |
|  |  |  |  | female |  | 9 | | 4 | Q4 (reference) | - | - | - |  |
|  |  | Economic and Social Disadvantage | CVD risk scores | female | Mean difference (SE) | 10 | | 1 |  | 0.00 | - | - | 0.01 |
|  |  | Economic and Social Disadvantage | CVD risk scores | female | Mean difference (SE) | 11 | | 1 | Q1 | 0.41 | - | - | 0.26 |
|  |  |  |  | female |  | 11 | | 2 | Q2 | 0.38 | - | - | 0.24 |
|  |  |  |  | female |  | 11 | | 3 | Q3 | 0.14 | - | - | 0.22 |
|  |  |  |  | female |  | 11 | | 4 | Q4 (reference) | - | - | - |  |
|  |  | Economic and Social Disadvantage | CVD risk scores | female | Mean difference (SE) | 12 | | 1 |  | -0.04 | - | - | 0.02 |
|  |  | Economic and Social Disadvantage | CVD risk scores | male | Beta | 13 | | 1 | Q1 | 0.44 | - | - |  |
|  |  |  |  | male |  | 13 | | 2 | Q2 | 0.21 | - | - |  |
|  |  |  |  | male |  | 13 | | 3 | Q3 | 0.27 | - | - |  |
|  |  |  |  | male |  | 13 | | 4 | Q4 | 0.20 | - | - |  |
|  |  | Economic and Social Disadvantage | CVD risk scores | female | Beta | 14 | | 1 | Q1 | -0.18 | - | - |  |
|  |  |  |  | female |  | 14 | | 2 | Q2 | -0.44 | - | - |  |
|  |  |  |  | female |  | 14 | | 3 | Q3 | -0.56 | - | - |  |
|  |  |  |  | female |  | 14 | | 4 | Q4 | -0.80 | - | - |  |
|  |  | Economic and Social Disadvantage | CVD risk scores | male | Beta | 15 | | 1 | Q1 | 0.18 | - | - |  |
|  |  |  |  | male |  | 15 | | 2 | Q2 | 0.20 | - | - |  |
|  |  |  |  | male |  | 15 | | 3 | Q3 | 0.47 | - | - |  |
|  |  |  |  | male |  | 15 | | 4 | Q4 | 0.33 | - | - |  |
|  |  | Economic and Social Disadvantage | CVD risk scores | female | Beta | 16 | | 1 | Q1 | 0.11 | - | - |  |
|  |  |  |  | female |  | 16 | | 2 | Q2 | 0.07 | - | - |  |
|  |  |  |  | female |  | 16 | | 3 | Q3 | 0.08 | - | - |  |
|  |  |  |  | female |  | 16 | | 4 | Q4 | -0.15 | - | - |  |
| [48] | Diez-Roux, 1997 | Economic and Social Disadvantage | cardiovascular health-related risk factors | overall | Mean difference (95%CI) | 1 | | 1 | quantile 1 | 1.60 | -0.20 | 34.00 |  |
|  |  | Economic and Social Disadvantage | cardiovascular health-related risk factors | overall | Mean difference (95%CI) | 2 | | 1 | quantile 1 | 1.00 | -0.20 | 2.30 |  |
|  |  | Economic and Social Disadvantage | cardiovascular health-related risk factors | overall | Mean difference (95%CI) | 3 | | 1 | quantile 1 | 0.90 | -0.30 | 2.00 |  |
|  |  | Economic and Social Disadvantage | cardiovascular health-related risk factors | overall | Mean difference (95%CI) | 4 | | 1 | quantile 1 | 3.21 | 1.50 | 4.00 |  |
|  |  | Economic and Social Disadvantage | cardiovascular health-related risk factors | overall | Mean difference (95%CI) | 5 | | 1 | quantile 1 | 1.40 | -0.80 | 3.60 |  |
|  |  | Economic and Social Disadvantage | cardiovascular health-related risk factors | overall | Mean difference (95%CI) | 6 | | 1 | quantile 1 | 1.70 | -0.40 | 3.70 |  |
|  |  | Economic and Social Disadvantage | cardiovascular health-related risk factors | overall | Mean difference (95%CI) | 7 | | 1 | quantile 1 | 0.10 | -1.90 | 2.10 |  |
|  |  | Economic and Social Disadvantage | cardiovascular health-related risk factors | overall | Mean difference (95%CI) | 8 | | 1 | quantile 1 | 3.10 | 0.50 | 5.70 |  |
|  |  | Economic and Social Disadvantage | cardiovascular health-related risk factors | overall | Mean difference (95%CI) | 9 | | 1 | quantile 1 | 1.40 | -0.50 | 3.20 |  |
|  |  | Economic and Social Disadvantage | cardiovascular health-related risk factors | overall | Mean difference (95%CI) | 10 | | 1 | quantile 1 | 0.30 | -0.90 | 1.60 |  |
|  |  | Economic and Social Disadvantage | cardiovascular health-related risk factors | overall | Mean difference (95%CI) | 11 | | 1 | quantile 1 | 0.80 | -0.40 | 2.00 |  |
|  |  | Economic and Social Disadvantage | cardiovascular health-related risk factors | overall | Mean difference (95%CI) | 12 | | 1 | quantile 1 | 1.80 | 0.70 | 3.20 |  |
|  |  | Economic and Social Disadvantage | cardiovascular health-related risk factors | overall | Mean difference (95%CI) | 13 | | 1 | quantile 1 | 2.60 | -0.10 | 5.20 |  |
|  |  | Economic and Social Disadvantage | cardiovascular health-related risk factors | overall | Mean difference (95%CI) | 14 | | 1 | quantile 1 | 0.70 | -1.50 | 3.00 |  |
|  |  | Economic and Social Disadvantage | cardiovascular health-related risk factors | overall | Mean difference (95%CI) | 15 | | 1 | quantile 1 | 0.60 | -1.60 | 2.80 |  |
|  |  | Economic and Social Disadvantage | cardiovascular health-related risk factors | overall | Mean difference (95%CI) | 16 | | 1 | quantile 1 | 2.90 | 0.10 | 5.70 |  |
|  |  | Economic and Social Disadvantage | metabolic and inflammatory-related risk factors | overall | Mean difference (95%CI) | 17 | | 1 | quantile 1 | 3.50 | -0.90 | 7.90 |  |
|  |  | Economic and Social Disadvantage | metabolic and inflammatory-related risk factors | overall | Mean difference (95%CI) | 18 | | 1 | quantile 1 | 1.80 | -1.30 | 4.80 |  |
|  |  | Economic and Social Disadvantage | metabolic and inflammatory-related risk factors | overall | Mean difference (95%CI) | 19 | | 1 | quantile 1 | 4.40 | 1.60 | 7.20 |  |
|  |  | Economic and Social Disadvantage | metabolic and inflammatory-related risk factors | overall | Mean difference (95%CI) | 20 | | 1 | quantile 1 | 4.60 | 0.70 | 8.60 |  |
|  |  | Economic and Social Disadvantage | metabolic and inflammatory-related risk factors | overall | Mean difference (95%CI) | 21 | | 1 | quantile 1 | -1.30 | -6.70 | 4.10 |  |
|  |  | Economic and Social Disadvantage | metabolic and inflammatory-related risk factors | overall | Mean difference (95%CI) | 22 | | 1 | quantile 1 | -2.50 | -7.50 | 2.50 |  |
|  |  | Economic and Social Disadvantage | metabolic and inflammatory-related risk factors | overall | Mean difference (95%CI) | 23 | | 1 | quantile 1 | -1.00 | -5.90 | 3.90 |  |
|  |  | Economic and Social Disadvantage | metabolic and inflammatory-related risk factors | overall | Mean difference (95%CI) | 24 | | 1 | quantile 1 | 1.60 | -5.00 | 8.30 |  |
|  |  | Economic and Social Disadvantage | metabolic and inflammatory-related risk factors | overall | Mean difference (95%CI) | 25 | | 1 | quantile 1 | 4.20 | 0.00 | 8.30 |  |
|  |  | Economic and Social Disadvantage | metabolic and inflammatory-related risk factors | overall | Mean difference (95%CI) | 26 | | 1 | quantile 1 | 2.20 | -0.80 | 5.10 |  |
|  |  | Economic and Social Disadvantage | metabolic and inflammatory-related risk factors | overall | Mean difference (95%CI) | 27 | | 1 | quantile 1 | 2.40 | -0.60 | 5.40 |  |
|  |  | Economic and Social Disadvantage | metabolic and inflammatory-related risk factors | overall | Mean difference (95%CI) | 28 | | 1 | quantile 1 | 5.20 | 1.80 | 8.60 |  |
|  |  | Economic and Social Disadvantage | metabolic and inflammatory-related risk factors | overall | Mean difference (95%CI) | 29 | | 1 | quantile 1 | 1.40 | -5.00 | 7.80 |  |
|  |  | Economic and Social Disadvantage | metabolic and inflammatory-related risk factors | overall | Mean difference (95%CI) | 30 | | 1 | quantile 1 | 3.60 | -1.90 | 9.10 |  |
|  |  | Economic and Social Disadvantage | metabolic and inflammatory-related risk factors | overall | Mean difference (95%CI) | 31 | | 1 | quantile 1 | 4.70 | -0.70 | 10.00 |  |
|  |  | Economic and Social Disadvantage | metabolic and inflammatory-related risk factors | overall | Mean difference (95%CI) | 32 | | 1 | quantile 1 | 2.90 | -3.90 | 9.70 |  |
| [49] | Djekic, 2018 | Economic and Social Disadvantage | glucose metabolism-related risk factors | overall | Mean (SD) | 1 | | 1 | high | 35.40 | - | - | 4.80 |
|  |  |  |  | overall |  | 1 | | 2 | low | 37.50 | - | - | 9.20 |
|  |  | Economic and Social Disadvantage | cardiovascular health-related risk factors | overall | Mean (SD) | 2 | | 1 | high | 125.40 | - | - | 17.30 |
|  |  |  |  | overall |  | 2 | | 2 | low | 128.90 | - | - | 17.70 |
|  |  | Economic and Social Disadvantage | cardiovascular health-related risk factors | overall | Mean (SD) | 3 | | 1 | high | 74.80 | - | - | 9.60 |
|  |  |  |  | overall |  | 3 | | 2 | low | 77.10 | - | - | 9.00 |
|  |  | Economic and Social Disadvantage | metabolic and inflammatory-related risk factors | overall | Mean (SD) | 4 | | 1 | high | 23.30 | - | - |  |
|  |  |  |  | overall |  | 4 | | 2 | low | 28.90 | - | - |  |
|  |  | Economic and Social Disadvantage | metabolic and inflammatory-related risk factors | overall | Mean (SD) | 5 | | 1 | high | 1.13 | - | - | 0.56 |
|  |  |  |  | overall |  | 5 | | 2 | low | 1.55 | - | - | 0.98 |
|  |  | Economic and Social Disadvantage | metabolic and inflammatory-related risk factors | overall | Mean (SD) | 6 | | 1 | high | 3.80 | - | - | 0.94 |
|  |  |  |  | overall |  | 6 | | 2 | low | 3.75 | - | - | 0.98 |
|  |  | Economic and Social Disadvantage | metabolic and inflammatory-related risk factors | overall | Mean (SD) | 7 | | 1 | high | 1.80 | - | - | 0.52 |
|  |  |  |  | overall |  | 7 | | 2 | low | 1.58 | - | - | 0.50 |
|  |  | Economic and Social Disadvantage | metabolic and inflammatory-related risk factors | overall | Mean (SD) | 8 | | 1 | high | 5.85 | - | - | 1.03 |
|  |  |  |  | overall |  | 8 | | 2 | low | 5.70 | - | - | 1.08 |
|  |  | Economic and Social Disadvantage | cardiovascular health-related risk factors | overall | OR (95%CI) | 9 | | 1 | high (reference) | 1.00 | - | - | - |
|  |  |  |  | overall |  | 9 | | 2 | low | 1.62 | 1.24 | 2.12 |  |
|  |  | Economic and Social Disadvantage | cardiovascular health-related risk factors | overall | OR (95%CI) | 10 | | 1 | high (reference) | 1.00 | - | - | - |
|  |  |  |  | overall |  | 10 | | 2 | low | 1.69 | 1.13 | 2.53 |  |
|  |  | Economic and Social Disadvantage | cardiovascular health-related risk factors | overall | OR (95%CI) | 11 | | 1 | high (reference) | 1.00 | - | - | - |
|  |  |  |  | overall |  | 11 | | 2 | low | 1.56 | 1.09 | 2.24 |  |
| [50] | Do, 2011 | Economic and Social Disadvantage | metabolic and inflammatory-related risk factors | overall | Beta | 1 | | 1 | tertile 1: low (reference) | - | - | - |  |
|  |  |  |  | overall |  | 1 | | 2 | tertile 2: medium | 8.65 | −0.62 | 18.78 |  |
|  |  |  |  | overall |  | 1 | | 3 | tertile 3: high | −3.04 | −11.91 | 6.72 |  |
|  |  | Social Cohesion and Social Capital | metabolic and inflammatory-related risk factors | overall | Beta (95%CI) | 2 | | 1 | tertile 1: low | −9.14 | −18.05 | 0.73 |  |
|  |  |  |  | overall |  | 2 | | 2 | tertile 2: medium | −10.30 | −18.27 | −1.57 |  |
|  |  |  |  | overall |  | 2 | | 3 | tertile 3: high (reference) | - | - | - |  |
|  |  | Crime and Safety | metabolic and inflammatory-related risk factors | overall | Beta | 3 | | 1 | tertile 1: low (reference) | - | - | - |  |
|  |  |  |  | overall |  | 3 | | 2 | tertile 2: medium | −14.50 | −21.82 | −6.52 |  |
|  |  |  |  | overall |  | 3 | | 3 | tertile 3: high | −14.25 | −22.96 | −4.55 |  |
|  |  | Crime and Safety | metabolic and inflammatory-related risk factors | overall | Beta | 4 | | 1 | tertile 1: low (reference) | - | - | - |  |
|  |  |  |  | overall |  | 4 | | 2 | tertile 2: medium | −8.27 | −16.90 | 1.25 |  |
|  |  |  |  | overall |  | 4 | | 3 | tertile 3: high | −10.46 | −19.20 | −0.77 |  |
|  |  | Economic and Social Disadvantage | metabolic and inflammatory-related risk factors | overall | Beta | 5 | | 1 | tertile 1: low (reference) | - | - | - |  |
|  |  |  |  | overall |  | 5 | | 2 | tertile 2: medium | −3.71 | −18.93 | 14.36 |  |
|  |  |  |  | overall |  | 5 | | 3 | tertile 3: high | 3.57 | −12.92 | 23.17 |  |
|  |  | Social Cohesion and Social Capital | metabolic and inflammatory-related risk factors | overall | Beta (95%CI) | 6 | | 1 | tertile 1: low | 7.21 | −11.00 | 29.14 |  |
|  |  |  |  | overall |  | 6 | | 2 | tertile 2: medium | 2.76 | −12.96 | 21.31 |  |
|  |  |  |  | overall |  | 6 | | 3 | tertile 3: high (reference) | - | - | - |  |
|  |  | Crime and Safety | metabolic and inflammatory-related risk factors | overall | Beta | 7 | | 1 | tertile 1: low (reference) | - | - | - |  |
|  |  |  |  | overall |  | 7 | | 2 | tertile 2: medium | 8.77 | −8.02 | 28.62 |  |
|  |  |  |  | overall |  | 7 | | 3 | tertile 3: high | 14.55 | −6.01 | 39.58 |  |
|  |  | Crime and Safety | metabolic and inflammatory-related risk factors | overall | Beta | 8 | | 1 | tertile 1: low (reference) | - | - | - |  |
|  |  |  |  | overall |  | 8 | | 2 | tertile 2: medium | 3.88 | −12.35 | 23.02 |  |
|  |  |  |  | overall |  | 8 | | 3 | tertile 3: high | 14.29 | −4.59 | 37.27 |  |
|  |  | Economic and Social Disadvantage | metabolic and inflammatory-related risk factors | overall | Beta | 9 | | 1 | tertile 1: low (reference) | - | - | - |  |
|  |  |  |  | overall |  | 9 | | 2 | tertile 2: medium | 2.41 | −4.32 | 9.62 |  |
|  |  |  |  | overall |  | 9 | | 3 | tertile 3: high | 7.44 | 0.15 | 15.26 |  |
|  |  | Social Cohesion and Social Capital | metabolic and inflammatory-related risk factors | overall | Beta (95%CI) | 10 | | 1 | tertile 1: low | 3.59 | −4.06 | 11.84 |  |
|  |  |  |  | overall |  | 10 | | 2 | tertile 2: medium | 5.22 | −1.58 | 12.48 |  |
|  |  |  |  | overall |  | 10 | | 3 | tertile 3: high (reference) | - | - | - |  |
|  |  | Crime and Safety | metabolic and inflammatory-related risk factors | overall | Beta | 11 | | 1 | tertile 1: low (reference) | - | - | - |  |
|  |  |  |  | overall |  | 11 | | 2 | tertile 2: medium | 9.16 | 2.09 | 16.72 |  |
|  |  |  |  | overall |  | 11 | | 3 | tertile 3: high | 8.03 | −0.17 | 16.89 |  |
|  |  | Crime and Safety | metabolic and inflammatory-related risk factors | overall | Beta | 12 | | 1 | tertile 1: low (reference) | - | - | - |  |
|  |  |  |  | overall |  | 12 | | 2 | tertile 2: medium | 5.62 | −1.42 | 13.17 |  |
|  |  |  |  | overall |  | 12 | | 3 | tertile 3: high | 3.99 | −3.92 | 12.57 |  |
|  |  | Economic and Social Disadvantage | metabolic and inflammatory-related risk factors | overall | Beta | 13 | | 1 | tertile 1: low (reference) | - | - | - |  |
|  |  |  |  | overall |  | 13 | | 2 | tertile 2: medium | 0.03 | −0.84 | 0.90 |  |
|  |  |  |  | overall |  | 13 | | 3 | tertile 3: high | 0.49 | −0.47 | 1.46 |  |
|  |  | Social Cohesion and Social Capital | metabolic and inflammatory-related risk factors | overall | Beta (95%CI) | 14 | | 1 | tertile 1: low | 0.98 | −0.04 | 2.01 |  |
|  |  |  |  | overall |  | 14 | | 2 | tertile 2: medium | 0.59 | −0.40 | 1.60 |  |
|  |  |  |  | overall |  | 14 | | 3 | tertile 3: high (reference) | - | - | - |  |
|  |  | Crime and Safety | metabolic and inflammatory-related risk factors | overall | Beta | 15 | | 1 | tertile 1: low (reference) | - | - | - |  |
|  |  |  |  | overall |  | 15 | | 2 | tertile 2: medium | −0.08 | −1.02 | 0.86 |  |
|  |  |  |  | overall |  | 15 | | 3 | tertile 3: high | 0.79 | −0.29 | 1.88 |  |
|  |  | Crime and Safety | metabolic and inflammatory-related risk factors | overall | Beta | 16 | | 1 | tertile 1: low (reference) | - | - | - |  |
|  |  |  |  | overall |  | 16 | | 2 | tertile 2: medium | 0.02 | −1.00 | 1.05 |  |
|  |  |  |  | overall |  | 16 | | 3 | tertile 3: high | 0.71 | −0.31 | 1.74 |  |
| [51] | Dragano, 2009 | Economic and Social Disadvantage | cardiovascular health-related risk factors | male | OR (95%CI) | 1 | | 1 | lowest (reference) | 1.00 | - | - |  |
|  |  |  |  | male |  | 1 | | 2 | mid-low | 1.16 | 0.92 | 1.46 |  |
|  |  |  |  | male |  | 1 | | 3 | mid-high | 1.25 | 0.98 | 1.60 |  |
|  |  |  |  | male |  | 1 | | 4 | highest | 1.45 | 1.09 | 1.93 |  |
|  |  | Economic and Social Disadvantage | cardiovascular health-related risk factors | female | OR (95%CI) | 2 | | 1 | lowest (reference) | 1.00 | - | - |  |
|  |  |  |  | female |  | 2 | | 2 | mid-low | 1.33 | 1.07 | 1.66 |  |
|  |  |  |  | female |  | 2 | | 3 | mid-high | 1.40 | 1.11 | 1.77 |  |
|  |  |  |  | female |  | 2 | | 4 | highest | 1.29 | 0.97 | 1.70 |  |
| [52] | Dubowitz, 2012 | Economic and Social Disadvantage | cardiovascular health-related risk factors | female | Beta (95%CI) | 1 | | 1 |  | −0.05 | −0.07 | −0.03 |  |
|  |  | Economic and Social Disadvantage | cardiovascular health-related risk factors | female | Beta (95%CI) | 2 | | 1 |  | −0.02 | −0.03 | −0.01 |  |
| [53] | Duncan, 2016 | Discrimination and Segregation | cardiovascular health-related risk factors | overall | Beta | 1 | | 1 | good (reference) | - | - | - |  |
|  |  |  |  | overall |  | 1 | | 2 | moderate | 7.30 | -0.92 | 15.40 |  |
|  |  |  |  | overall |  | 1 | | 3 | bad | 13.20 | 3.20 | 23.10 |  |
|  |  |  |  | overall | Beta | 2 | | 1 | yes (reference) | - | - | - |  |
|  |  |  |  | overall |  | 2 | | 2 | no | -6.40 | -16.70 | 3.90 |  |
|  |  |  |  | overall | Beta | 3 | | 1 | no (reference) | - | - | - |  |
|  |  |  |  | overall |  | 3 | | 2 | yes | -4.96 | -14.50 | 4.60 |  |
|  |  |  |  | overall | Beta | 4 | | 1 | no (reference) | - | - | - |  |
|  |  |  |  | overall |  | 4 | | 2 | yes | -6.04 | -13.90 | 1.90 |  |
|  |  |  |  | overall | Beta | 5 | | 1 | good (reference) | - | - | - |  |
|  |  |  |  | overall |  | 5 | | 2 | moderate | 0.68 | -4.30 | 5.60 |  |
|  |  |  |  | overall |  | 5 | | 3 | bad | 8.50 | 2.80 | 14.30 |  |
|  |  |  |  | overall | Beta | 6 | | 1 | yes (reference) | - | - | - |  |
|  |  |  |  | overall |  | 6 | | 2 | no | -0.64 | -8.40 | 7.10 |  |
|  |  |  |  | overall | Beta | 7 | | 1 | no (reference) | - | - | - |  |
|  |  |  |  | overall |  | 7 | | 2 | yes | -1.98 | -8.80 | 4.90 |  |
|  |  |  |  | overall | Beta | 8 | | 1 | no (reference) | - | - | - |  |
|  |  |  |  | overall |  | 8 | | 2 | yes | -5.50 | -10.20 | -0.66 |  |
| [54] | Dwane, 2020 | Economic and Social Disadvantage | metabolic and inflammatory-related risk factors | overall | Posterior median OR (95%CI) | 1 | | 1 | I (least deprived) | 1.00 | 0.60 | -1.60 |  |
|  |  |  |  | overall |  | 1 | | 2 | II | 1.20 | 0.70 | 1.90 |  |
|  |  |  |  | overall |  | 1 | | 3 | III | 0.90 | 0.60 | 1.40 |  |
|  |  |  |  | overall |  | 1 | | 4 | IV | 0.90 | 0.60 | 1.30 |  |
|  |  |  |  | overall |  | 1 | | 5 | V (most deprived - reference) | 1.00 | - | - |  |
| [55] | Eichinger, 2015 | Social Relationships and Norms | glucose metabolism-related risk factors | male | Beta | 1 | | 1 |  | 0.01 | - | - |  |
|  |  | Social Relationships and Norms | glucose metabolism-related risk factors | overall | Beta | 2 | | 1 |  | 0.01 | - | - |  |
| [56] | Ellaway, 2007 | Civic Participation and Engagement | cardiovascular health-related risk factors | male | Beta (SE) | 1 | | 1 | yes | 141.50 | - | - | 1.82 |
|  |  |  |  | male |  | 1 | | 2 | no | 140.70 | - | - | 0.77 |
|  |  | Civic Participation and Engagement | cardiovascular health-related risk factors | male | Beta (SE) | 2 | | 1 | yes | 84.97 | - | - | 1.05 |
|  |  |  |  | male |  | 2 | | 2 | no | 83.80 | - | - | 0.44 |
|  |  | Civic Participation and Engagement | cardiovascular health-related risk factors | female | Beta (SE) | 3 | | 1 | yes | 131.70 | - | - | 2.03 |
|  |  |  |  | female |  | 3 | | 2 | no | 131.60 | - | - | 0.62 |
|  |  | Civic Participation and Engagement | cardiovascular health-related risk factors | female | Beta (SE) | 4 | | 1 | yes | 79.96 | - | - | 1.15 |
|  |  |  |  | female |  | 4 | | 2 | no | 79.65 | - | - | 0.35 |
|  |  | Civic Participation and Engagement | cardiovascular health-related risk factors | male | Beta (SE) | 5 | | 1 | yes | 141.40 | - | - | 1.56 |
|  |  |  |  | male |  | 5 | | 2 | no | 140.60 | - | - | 0.77 |
|  |  | Civic Participation and Engagement | cardiovascular health-related risk factors | male | Beta (SE) | 6 | | 1 | yes | 84.63 | - | - | 0.90 |
|  |  |  |  | male |  | 6 | | 2 | no | 83.81 | - | - | 0.45 |
|  |  | Civic Participation and Engagement | cardiovascular health-related risk factors | female | Beta (SE) | 7 | | 1 | yes | 132.20 | - | - | 1.25 |
|  |  |  |  | female |  | 7 | | 2 | no | 131.50 | - | - | 0.65 |
|  |  | Civic Participation and Engagement | cardiovascular health-related risk factors | female | Beta (SE) | 8 | | 1 | yes | 80.51 | - | - | 0.71 |
|  |  |  |  | female |  | 8 | | 2 | no | 79.49 | - | - | 0.37 |
|  |  | Civic Participation and Engagement | cardiovascular health-related risk factors | male | Beta (SE) | 9 | | 1 | yes | 139.10 | - | - | 1.31 |
|  |  |  |  | male |  | 9 | | 2 | no | 141.10 | - | - | 0.79 |
|  |  | Civic Participation and Engagement | cardiovascular health-related risk factors | male | Beta (SE) | 10 | | 1 | yes | 82.89 | - | - | 0.76 |
|  |  |  |  | male |  | 10 | | 2 | no | 84.18 | - | - | 0.46 |
|  |  | Civic Participation and Engagement | cardiovascular health-related risk factors | female | Beta (SE) | 11 | | 1 | yes | 131.00 | - | - | 0.92 |
|  |  |  |  | female |  | 11 | | 2 | no | 132.00 | - | - | 0.72 |
|  |  | Civic Participation and Engagement | cardiovascular health-related risk factors | female | Beta (SE) | 12 | | 1 | yes | 79.21 | - | - | 0.52 |
|  |  |  |  | female |  | 12 | | 2 | no | 79.94 | - | - | 0.41 |
|  |  | Civic Participation and Engagement | cardiovascular health-related risk factors | male | Beta (SE) | 13 | | 1 | yes | 139.90 | - | - | 1.73 |
|  |  |  |  | male |  | 13 | | 2 | no | 140.80 | - | - | 0.77 |
|  |  | Civic Participation and Engagement | cardiovascular health-related risk factors | male | Beta (SE) | 14 | | 1 | yes | 84.55 | - | - | 1.00 |
|  |  |  |  | male |  | 14 | | 2 | no | 83.84 | - | - | 0.44 |
|  |  | Civic Participation and Engagement | cardiovascular health-related risk factors | female | Beta (SE) | 15 | | 1 | yes | 130.70 | - | - | 1.24 |
|  |  |  |  | female |  | 15 | | 2 | no | 131.80 | - | - | 0.65 |
|  |  | Civic Participation and Engagement | cardiovascular health-related risk factors | female | Beta (SE) | 16 | | 1 | yes | 79.59 | - | - | 0.70 |
|  |  |  |  | female |  | 16 | | 2 | no | 79.69 | - | - | 0.37 |
|  |  | Civic Participation and Engagement | cardiovascular health-related risk factors | male | Beta (SE) | 17 | | 1 | yes | 142.70 | - | - | 1.52 |
|  |  |  |  | male |  | 17 | | 2 | no | 140.40 | - | - | 0.78 |
|  |  | Civic Participation and Engagement | cardiovascular health-related risk factors | male | Beta (SE) | 18 | | 1 | yes | 84.14 | - | - | 0.88 |
|  |  |  |  | male |  | 18 | | 2 | no | 83.88 | - | - | 0.45 |
|  |  | Civic Participation and Engagement | cardiovascular health-related risk factors | female | Beta (SE) | 19 | | 1 | yes | 130.70 | - | - | 1.22 |
|  |  |  |  | female |  | 19 | | 2 | no | 131.90 | - | - | 0.66 |
|  |  | Civic Participation and Engagement | cardiovascular health-related risk factors | female | Beta (SE) | 20 | | 1 | yes | 79.14 | - | - | 0.69 |
|  |  |  |  | female |  | 20 | | 2 | no | 79.80 | - | - | 0.37 |
|  |  | Civic Participation and Engagement | cardiovascular health-related risk factors | male | Beta (SE) | 21 | | 1 | yes | 141.20 | - | - | 1.07 |
|  |  |  |  | male |  | 21 | | 2 | no | 140.50 | - | - | 0.87 |
|  |  | Civic Participation and Engagement | cardiovascular health-related risk factors | male | Beta (SE) | 22 | | 1 | yes | 84.26 | - | - | 0.62 |
|  |  |  |  | male |  | 22 | | 2 | no | 83.72 | - | - | 0.50 |
|  |  | Civic Participation and Engagement | cardiovascular health-related risk factors | female | Beta (SE) | 23 | | 1 | yes | 130.70 | - | - | 0.95 |
|  |  |  |  | female |  | 23 | | 2 | no | 132.10 | - | - | 0.71 |
|  |  | Civic Participation and Engagement | cardiovascular health-related risk factors | female | Beta (SE) | 24 | | 1 | yes | 79.75 | - | - | 0.54 |
|  |  |  |  | female |  | 24 | | 2 | no | 79.63 | - | - | 0.40 |
|  |  | Civic Participation and Engagement | cardiovascular health-related risk factors | male | Beta (SE) | 25 | | 1 | yes | 141.10 | - | - | 0.86 |
|  |  |  |  | male |  | 25 | | 2 | no | 140.10 | - | - | 1.11 |
|  |  | Civic Participation and Engagement | cardiovascular health-related risk factors | male | Beta (SE) | 26 | | 1 | yes | 84.04 | - | - | 0.49 |
|  |  |  |  | male |  | 26 | | 2 | no | 83.70 | - | - | 0.64 |
|  |  | Civic Participation and Engagement | cardiovascular health-related risk factors | female | Beta (SE) | 27 | | 1 | yes | 131.20 | - | - | 0.68 |
|  |  |  |  | female |  | 27 | | 2 | no | 132.80 | - | - | 1.07 |
|  |  | Civic Participation and Engagement | cardiovascular health-related risk factors | female | Beta (SE) | 28 | | 1 | yes | 79.59 | - | - | 0.38 |
|  |  |  |  | female |  | 28 | | 2 | no | 79.91 | - | - | 0.60 |
|  |  | Civic Participation and Engagement | cardiovascular health-related risk factors | male | Beta (SE) | 29 | | 1 | yes | 64.87 | - | - | 1.10 |
|  |  |  |  | male |  | 29 | | 2 | no | 65.22 | - | - | 0.46 |
|  |  | Civic Participation and Engagement | cardiovascular health-related risk factors | female | Beta (SE) | 30 | | 1 | yes | 67.36 | - | - | 1.15 |
|  |  |  |  | female |  | 30 | | 2 | no | 68.29 | - | - | 0.35 |
|  |  | Civic Participation and Engagement | cardiovascular health-related risk factors | male | Beta (SE) | 31 | | 1 | yes | 64.44 | - | - | 0.94 |
|  |  |  |  | male |  | 31 | | 2 | no | 65.30 | - | - | 0.47 |
|  |  | Civic Participation and Engagement | cardiovascular health-related risk factors | female | Beta (SE) | 32 | | 1 | yes | 67.96 | - | - | 0.71 |
|  |  |  |  | female |  | 32 | | 2 | no | 68.30 | - | - | 0.37 |
|  |  | Civic Participation and Engagement | cardiovascular health-related risk factors | male | Beta (SE) | 33 | | 1 | yes | 63.19 | - | - | 0.79 |
|  |  |  |  | male |  | 33 | | 2 | no | 65.68 | - | - | 0.48 |
|  |  | Civic Participation and Engagement | cardiovascular health-related risk factors | female | Beta (SE) | 34 | | 1 | yes | 67.10 | - | - | 0.52 |
|  |  |  |  | female |  | 34 | | 2 | no | 68.89 | - | - | 0.41 |
|  |  | Civic Participation and Engagement | cardiovascular health-related risk factors | male | Beta (SE) | 35 | | 1 | yes | 65.36 | - | - | 1.04 |
|  |  |  |  | male |  | 35 | | 2 | no | 65.16 | - | - | 0.46 |
|  |  | Civic Participation and Engagement | cardiovascular health-related risk factors | female | Beta (SE) | 36 | | 1 | yes | 66.84 | - | - | 0.70 |
|  |  |  |  | female |  | 36 | | 2 | no | 68.55 | - | - | 0.37 |
|  |  | Civic Participation and Engagement | cardiovascular health-related risk factors | male | Beta (SE) | 37 | | 1 | yes | 64.65 | - | - | 0.92 |
|  |  |  |  | male |  | 37 | | 2 | no | 65.27 | - | - | 0.47 |
|  |  | Civic Participation and Engagement | cardiovascular health-related risk factors | female | Beta (SE) | 38 | | 1 | yes | 66.46 | - | - | 0.69 |
|  |  |  |  | female |  | 38 | | 2 | no | 68.67 | - | - | 0.37 |
|  |  | Civic Participation and Engagement | cardiovascular health-related risk factors | male | Beta (SE) | 39 | | 1 | yes | 63.20 | - | - | 0.64 |
|  |  |  |  | male |  | 39 | | 2 | no | 66.35 | - | - | 0.52 |
|  |  | Civic Participation and Engagement | cardiovascular health-related risk factors | female | Beta (SE) | 40 | | 1 | yes | 65.95 | - | - | 0.53 |
|  |  |  |  | female |  | 40 | | 2 | no | 69.38 | - | - | 0.40 |
|  |  | Civic Participation and Engagement | cardiovascular health-related risk factors | male | Beta (SE) | 41 | | 1 | yes | 64.27 | - | - | 0.51 |
|  |  |  |  | male |  | 41 | | 2 | no | 66.95 | - | - | 0.66 |
|  |  | Civic Participation and Engagement | cardiovascular health-related risk factors | female | Beta (SE) | 42 | | 1 | yes | 67.38 | - | - | 0.38 |
|  |  |  |  | female |  | 42 | | 2 | no | 70.66 | - | - | 0.60 |
| [57] | Engström, 2001 | Economic and Social Disadvantage | CVD risk scores | female | correlation | 1 | | 1 |  | -0.72 | - | - |  |
|  |  | Economic and Social Disadvantage | CVD risk scores | female | correlation | 2 | | 1 |  | -0.73 | - | - |  |
| [58] | Ferguson, 2020 | Economic and Social Disadvantage | cardiovascular health-related risk factors | male | Beta (95%CI) | 1 | | 1 |  | -1.31 | -1.80 | -0.81 |  |
|  |  | Economic and Social Disadvantage | cardiovascular health-related risk factors | male | Beta (95%CI) | 2 | | 1 | Spline 1 (z-score <-1) | -0.74 | -4.77 | 3.28 |  |
|  |  |  |  | male |  | 2 | | 2 | Spline 2 (z-score -1 to 0) | 1.45 | -1.87 | 4.77 |  |
|  |  |  |  | male |  | 2 | | 3 | Spline 3 (z-score >0 to 1) | -0.65 | -3.28 | 1.97 |  |
|  |  |  |  | male |  | 2 | | 4 | Spline 4 (z-score >1) | 0.76 | -0.06 | 1.57 |  |
|  |  | Economic and Social Disadvantage | cardiovascular health-related risk factors | female | Beta (95%CI) | 3 | | 1 |  | -0.46 | -1.58 | 0.66 |  |
|  |  | Economic and Social Disadvantage | cardiovascular health-related risk factors | female | Beta (95%CI) | 4 | | 1 | Spline 1 (z-score <-1) | -0.52 | -2.83 | 1.80 |  |
|  |  |  |  | female |  | 4 | | 2 | Spline 2 (z-score -1 to 0) | 4.02 | 1.71 | 6.33 |  |
|  |  |  |  | female |  | 4 | | 3 | Spline 3 (z-score >0 to 1) | -2.52 | -4.82 | -0.22 |  |
|  |  |  |  | female |  | 4 | | 4 | Spline 4 (z-score >1) | 0.36 | -1.16 | 1.88 |  |
|  |  | Economic and Social Disadvantage | cardiovascular health-related risk factors | male | Beta (95%CI) | 5 | | 1 |  | -0.09 | -0.76 | 0.58 |  |
|  |  | Economic and Social Disadvantage | cardiovascular health-related risk factors | male | Beta (95%CI) | 6 | | 1 | Spline 1 (z-score <-1) | -4.28 | -6.10 | -2.47 |  |
|  |  |  |  | male |  | 6 | | 2 | Spline 2 (z-score -1 to 0) | 2.80 | 0.86 | 4.75 |  |
|  |  |  |  | male |  | 6 | | 3 | Spline 3 (z-score >0 to 1) | -2.58 | -4.71 | -0.43 |  |
|  |  |  |  | male |  | 6 | | 4 | Spline 4 (z-score >1) | 0.76 | 0.02 | 1.50 |  |
|  |  | Economic and Social Disadvantage | cardiovascular health-related risk factors | female | Beta (95%CI) | 7 | | 1 |  | 0.50 | -0.68 | 1.68 |  |
|  |  | Economic and Social Disadvantage | cardiovascular health-related risk factors | female | Beta (95%CI) | 8 | | 1 | Spline 1 (z-score <-1) | 1.11 | 0.22 | 2.00 |  |
|  |  |  |  | female |  | 8 | | 2 | Spline 2 (z-score -1 to 0) | 0.19 | -1.68 | 2.07 |  |
|  |  |  |  | female |  | 8 | | 3 | Spline 3 (z-score >0 to 1) | -1.47 | -3.79 | 0.85 |  |
|  |  |  |  | female |  | 8 | | 4 | Spline 4 (z-score >1) | 0.39 | -0.69 | 1.47 |  |
| [59] | Finch, 2010 | Economic and Social Disadvantage | CVD risk scores | overall | Beta | 1 | | 1 | ICE | -0.01 | - | - |  |
| [60] | Foraker, 2019 | Economic and Social Disadvantage | CVD risk scores | overall | Beta (95%CI) | 1 | | 1 |  | 0.19 | 0.09 | 0.28 |  |
|  |  | Economic and Social Disadvantage | CVD risk scores | overall | Beta | 2 | | 1 | cat 1: <$25,480 (reference) | - | - | - |  |
|  |  |  |  | overall |  | 2 | | 2 | cat 2 | 0.17 | 0.01 | 0.32 |  |
|  |  |  |  | overall |  | 2 | | 3 | cat 3: >$35,375 | 0.37 | 0.19 | 0.56 |  |
|  |  | Economic and Social Disadvantage | CVD risk scores | overall | Beta (95%CI) | 3 | | 1 |  | 0.06 | 0.03 | 0.09 |  |
| [61] | Ford, 2006 | Social Relationships and Norms | metabolic and inflammatory-related risk factors | male | Mean (SE) | 1 | | 1 | 0-1 (few ties) | 5.19 | - | - | 0.04 |
|  |  |  |  | male |  | 1 | | 2 | 2 | 5.29 | - | - | 0.04 |
|  |  |  |  | male |  | 1 | | 3 | 3 | 5.29 | - | - | 0.03 |
|  |  |  |  | male |  | 1 | | 4 | 4 (most ties) | 5.29 | - | - | 0.05 |
|  |  | Social Relationships and Norms | metabolic and inflammatory-related risk factors | female | Mean (SE) | 2 | | 1 | 0-1 (few ties) | 5.39 | - | - | 0.04 |
|  |  |  |  | female |  | 2 | | 2 | 2 | 5.38 | - | - | 0.03 |
|  |  |  |  | female |  | 2 | | 3 | 3 | 5.36 | - | - | 0.03 |
|  |  |  |  | female |  | 2 | | 4 | 4 (most ties) | 5.32 | - | - | 0.03 |
|  |  | Social Relationships and Norms | metabolic and inflammatory-related risk factors | male | OR (95%CI) | 3 | | 1 | 0-1 (few ties) | 0.93 | 0.62 | 1.39 |  |
|  |  |  |  | male |  | 3 | | 2 | 2 | 0.96 | 0.67 | 1.38 |  |
|  |  |  |  | male |  | 3 | | 3 | 3 | 0.86 | 0.53 | 1.40 |  |
|  |  |  |  | male |  | 3 | | 4 | 4 (most ties) (reference) | 1.00 | - | - |  |
|  |  | Social Relationships and Norms | metabolic and inflammatory-related risk factors | male | OR (95%CI) | 4 | | 1 | 0-1 (few ties) | 1.80 | 1.11 | 2.92 |  |
|  |  |  |  | male |  | 4 | | 2 | 2 | 1.54 | 1.04 | 2.28 |  |
|  |  |  |  | male |  | 4 | | 3 | 3 | 1.29 | 0.83 | 2.03 |  |
|  |  |  |  | male |  | 4 | | 4 | 4 (most ties) (reference) | 1.00 | - | - |  |
|  |  | Social Relationships and Norms | metabolic and inflammatory-related risk factors | female | OR (95%CI) | 5 | | 1 | 0-1 (few ties) | 1.22 | 0.85 | 1.76 |  |
|  |  |  |  | female |  | 5 | | 2 | 2 | 0.88 | 0.65 | 1.21 |  |
|  |  |  |  | female |  | 5 | | 3 | 3 | 1.05 | 0.75 | 1.47 |  |
|  |  |  |  | female |  | 5 | | 4 | 4 (most ties) (reference) | 1.00 | - | - |  |
|  |  | Social Relationships and Norms | metabolic and inflammatory-related risk factors | female | OR (95%CI) | 6 | | 1 | 0-1 (few ties) | 0.91 | 0.57 | 1.46 |  |
|  |  |  |  | female |  | 6 | | 2 | 2 | 1.00 | 0.66 | 1.50 |  |
|  |  |  |  | female |  | 6 | | 3 | 3 | 1.06 | 0.75 | 1.49 |  |
|  |  |  |  | female |  | 6 | | 4 | 4 (most ties) (reference) | 1.00 | - | - |  |
|  |  | Social Relationships and Norms | metabolic and inflammatory-related risk factors | male | OR (95%CI) | 7 | | 1 |  | 1.10 | 0.85 | 1.42 |  |
|  |  | Social Relationships and Norms | metabolic and inflammatory-related risk factors | male | OR (95%CI) | 8 | | 1 |  | 0.92 | 0.71 | 1.19 |  |
|  |  | Social Relationships and Norms | metabolic and inflammatory-related risk factors | male | OR (95%CI) | 9 | | 1 |  | 1.05 | 0.81 | 1.36 |  |
|  |  | Social Relationships and Norms | metabolic and inflammatory-related risk factors | male | OR (95%CI) | 10 | | 1 |  | 0.88 | 0.64 | 1.20 |  |
|  |  | Social Relationships and Norms | metabolic and inflammatory-related risk factors | male | OR (95%CI) | 11 | | 1 |  | 0.82 | 0.65 | 1.03 |  |
|  |  | Social Relationships and Norms | metabolic and inflammatory-related risk factors | male | OR (95%CI) | 12 | | 1 |  | 0.67 | 0.52 | 0.87 |  |
|  |  | Social Relationships and Norms | metabolic and inflammatory-related risk factors | female | OR (95%CI) | 13 | | 1 |  | 1.14 | 0.82 | 1.58 |  |
|  |  | Social Relationships and Norms | metabolic and inflammatory-related risk factors | female | OR (95%CI) | 14 | | 1 |  | 0.84 | 0.67 | 1.05 |  |
|  |  | Social Relationships and Norms | metabolic and inflammatory-related risk factors | female | OR (95%CI) | 15 | | 1 |  | 0.92 | 0.76 | 1.12 |  |
|  |  | Social Relationships and Norms | metabolic and inflammatory-related risk factors | female | OR (95%CI) | 16 | | 1 |  | 1.21 | 0.82 | 1.79 |  |
|  |  | Social Relationships and Norms | metabolic and inflammatory-related risk factors | female | OR (95%CI) | 17 | | 1 |  | 1.01 | 0.81 | 1.26 |  |
|  |  | Social Relationships and Norms | metabolic and inflammatory-related risk factors | female | OR (95%CI) | 18 | | 1 |  | 1.04 | 0.79 | 1.37 |  |
| [62] | Ford, 2019 | Social Relationships and Norms | metabolic and inflammatory-related risk factors | female | OR (95%CI) | 1 | | 1 | < 1 mg/L (low risk, reference) | 1.00 | - | - |  |
|  |  |  |  | female |  | 1 | | 2 | 1–3 mg/L (moderate risk) | 0.84 | 0.68 | 1.03 |  |
|  |  |  |  | female |  | 1 | | 3 | > 3–10 mg/L (high risk) | 1.01 | 0.78 | 1.30 |  |
|  |  |  |  | female |  | 1 | | 4 | > 10mg/L (very high risk) | 1.00 | 0.75 | 1.33 |  |
| [63] | Fuller, 2018 | Social Relationships and Norms | cardiovascular health-related risk factors | overall | Beta (SE) | 1 | | 1 |  | 2.43 | - | - | 1.27 |
|  |  | Social Relationships and Norms | cardiovascular health-related risk factors | overall | Beta (SE) | 2 | | 1 |  | 8.79 | - | - | 3.84 |
|  |  | Social Relationships and Norms | cardiovascular health-related risk factors | overall | Beta (SE) | 3 | | 1 |  | 10.80 | - | - | 5.12 |
|  |  | Social Relationships and Norms | cardiovascular health-related risk factors | overall | Beta (SE) | 4 | | 1 |  | 15.98 | - | - | 5.32 |
| [64] | Gallo, 2012 | Economic and Social Disadvantage | metabolic and inflammatory-related risk factors | overall | Beta (95%CI) | 1 | | 1 |  | −18.05 | −37.40 | 1.30 |  |
|  |  | Economic and Social Disadvantage | metabolic and inflammatory-related risk factors | overall | Beta (95%CI) | 2 | | 1 |  | 0.40 | −11.72 | 12.52 |  |
|  |  | Economic and Social Disadvantage | metabolic and inflammatory-related risk factors | overall | Beta (95%CI) | 3 | | 1 |  | −2.08 | −7.89 | 3.73 |  |
| [65] | Garcia, 2016 | Economic and Social Disadvantage | glucose metabolism-related risk factors | overall | HR (95%CI) | 1 | | 1 |  | 1.05 | 0.88 | 1.26 |  |
|  |  | Economic and Social Disadvantage | glucose metabolism-related risk factors | overall | HR (95%CI) | 2 | | 1 |  | 1.22 | 0.99 | 1.50 |  |
| [66] | Garcia, 2015 | Economic and Social Disadvantage | glucose metabolism-related risk factors | overall | OR (95%CI) | 1 | | 1 |  | 0.90 | 0.71 | 1.14 |  |
| [67] | Gary-Webb, 2020 | Crime and Safety | cardiovascular health-related risk factors | overall | Beta (SE) | 1 | | 1 |  | −3.79 | - | - | 1.80 |
|  |  | Crime and Safety | cardiovascular health-related risk factors | overall | Beta (SE) | 2 | | 1 |  | −1.36 | - | - | 0.96 |
|  |  | Crime and Safety | glucose metabolism-related risk factors | overall | Beta (SE) | 3 | | 1 |  | 0.23 | - | - | 0.12 |
|  |  | Crime and Safety | metabolic and inflammatory-related risk factors | overall | Beta (SE) | 4 | | 1 |  | −0.75 | - | - | 1.54 |
|  |  | Crime and Safety | metabolic and inflammatory-related risk factors | overall | Beta (SE) | 5 | | 1 |  | −1.15 | - | - | 2.00 |
|  |  | Crime and Safety | metabolic and inflammatory-related risk factors | overall | Beta (SE) | 6 | | 1 |  | −0.83 | - | - | 1.06 |
|  |  | Crime and Safety | metabolic and inflammatory-related risk factors | overall | Beta (SE) | 7 | | 1 |  | −0.25 | - | - | 0.13 |
|  |  | Crime and Safety | metabolic and inflammatory-related risk factors | overall | Beta (SE) | 8 | | 1 |  | −0.46 | - | - | 1.69 |
|  |  | Crime and Safety | glucose metabolism-related risk factors | overall | Beta | 9 | | 1 |  | 0.27 | - | - |  |
| [68] | Gebreab, 2015 | Economic and Social Disadvantage | CVD risk scores | overall | OR (95%CI) | 1 | | 1 |  | 0.89 | 0.84 | 0.94 |  |
|  |  | Economic and Social Disadvantage | CVD risk scores | overall | OR (95%CI) | 2 | | 1 |  | 1.04 | 0.99 | 1.09 |  |
| [69] | Grimaud, 2013 | Economic and Social Disadvantage | cardiovascular health-related risk factors | male | Beta | 1 | | 1 | tertile 1 (reference) | 0.00 | - | - |  |
|  |  |  |  | male |  | 1 | | 2 | tertile 2 | -5.80 | -19.80 | 8.10 |  |
|  |  |  |  | male |  | 1 | | 3 | tertile 3 | -5.90 | -21.20 | 9.50 |  |
|  |  | Economic and Social Disadvantage | cardiovascular health-related risk factors | female | Beta | 2 | | 1 | tertile 1 (reference) | 0.00 | - | - |  |
|  |  |  |  | female |  | 2 | | 2 | tertile 2 | -5.70 | -15.00 | 3.90 |  |
|  |  |  |  | female |  | 2 | | 3 | tertile 3 | -12.40 | -22.80 | -2.00 |  |
| [70] | Höfelmann, 2012 | Economic and Social Disadvantage | cardiovascular health-related risk factors | overall | Beta | 1 | | 1 | lower tertiles (reference) | - | - | - |  |
|  |  |  |  | overall |  | 1 | | 2 | intermediary | -2.90 | -3.22 | -2.58 |  |
|  |  |  |  | overall |  | 1 | | 3 | higher | -6.62 | -6.96 | -6.29 |  |
| [71] | Halonen, 2015 | Economic and Social Disadvantage | metabolic and inflammatory-related risk factors | overall | RR (95%CI) | 1 | | 1 | low childhood adversity-low adult disadvantage (reference) | 1.00 | - | - |  |
|  |  |  |  | overall |  | 1 | | 2 | low childhood adversity-high adult disadvantage | 1.06 | 1.01 | 1.11 |  |
|  |  |  |  | overall |  | 1 | | 3 | high childhood adversity-low adult disadvantage | 1.06 | 1.00 | 1.14 |  |
|  |  |  |  | overall |  | 1 | | 4 | high childhood adversity-high adult disadvantage | 1.10 | 1.02 | 1.18 |  |
| [72] | Hamad, 2020 | Economic and Social Disadvantage | metabolic and inflammatory-related risk factors | overall | Beta (95%CI) | 1 | | 1 |  | 0.44 | 0.06 | 0.83 |  |
| [73] | Hanson, 1988 | Social Relationships and Norms | cardiovascular health-related risk factors | overall | Beta (SE) | 1 | | 1 |  | -1.59 | - | - | 0.72 |
|  |  | Social Relationships and Norms | cardiovascular health-related risk factors | overall | Beta (SE) | 2 | | 1 |  | -1.37 | - | - | 0.36 |
| [74] | Helminen, 1995 | Social Relationships and Norms | cardiovascular health-related risk factors | overall | Mean (95%CI) | 1 | | 1 |  | 136.00 | 131.00 | 142.00 |  |
|  |  |  |  | male | Mean (95%CI) | 1 | | 1 |  | 130.00 | 127.00 | 134.00 |  |
| [75] | Helminen, 1995 | Social Relationships and Norms | cardiovascular health-related risk factors | male | Mean (95%CI) | 1 | | 1 | weak | 1.01 | 0.97 | 1.05 |  |
|  |  |  |  | male |  | 1 | | 2 | strong | 1.04 | 1.01 | 1.08 |  |
|  |  | Social Relationships and Norms | cardiovascular health-related risk factors | male | Mean (95%CI) | 2 | | 1 | weak | 1.02 | 0.97 | 1.06 |  |
|  |  |  |  | male |  | 2 | | 2 | strong | 1.04 | 1.01 | 1.07 |  |
|  |  | Social Relationships and Norms | cardiovascular health-related risk factors | male | Mean (95%CI) | 3 | | 1 | weak | 1.02 | 0.99 | 1.06 |  |
|  |  |  |  | male |  | 3 | | 2 | strong | 1.04 | 1.01 | 1.07 |  |
|  |  | Social Relationships and Norms | cardiovascular health-related risk factors | male | Mean (95%CI) | 4 | | 1 | weak | 1.05 | 1.00 | 1.10 |  |
|  |  |  |  | male |  | 4 | | 2 | strong | 1.03 | 1.00 | 1.06 |  |
|  |  | Social Relationships and Norms | cardiovascular health-related risk factors | male | Mean (95%CI) | 5 | | 1 | weak | 1.03 | 0.98 | 1.08 |  |
|  |  |  |  | male |  | 5 | | 2 | strong | 1.01 | 1.01 | 1.07 |  |
|  |  | Social Relationships and Norms | cardiovascular health-related risk factors | male | Mean (95%CI) | 6 | | 1 | weak | 1.00 | 0.91 | 1.05 |  |
|  |  |  |  | male |  | 6 | | 2 | strong | 1.04 | 1.01 | 1.08 |  |
|  |  | Social Relationships and Norms | cardiovascular health-related risk factors | male | Mean (95%CI) | 7 | | 1 | weak | 1.02 | 0.98 | 1.07 |  |
|  |  |  |  | male |  | 7 | | 2 | strong | 1.04 | 1.01 | 1.07 |  |
|  |  | Social Relationships and Norms | cardiovascular health-related risk factors | male | Mean (95%CI) | 8 | | 1 | weak | 1.03 | 0.98 | 1.08 |  |
|  |  |  |  | male |  | 8 | | 2 | strong | 1.04 | 1.00 | 1.07 |  |
|  |  | Social Relationships and Norms | cardiovascular health-related risk factors | male | Mean (95%CI) | 9 | | 1 | weak | 0.97 | 0.95 | 1.00 |  |
|  |  |  |  | male |  | 9 | | 2 | strong | 1.07 | 1.03 | 1.10 |  |
|  |  | Social Relationships and Norms | cardiovascular health-related risk factors | male | Mean (95%CI) | 10 | | 1 | weak | 1.07 | 1.02 | 1.12 |  |
|  |  |  |  | male |  | 10 | | 2 | strong | 1.02 | 0.99 | 1.05 |  |
|  |  | Social Relationships and Norms | cardiovascular health-related risk factors | male | Mean (95%CI) | 11 | | 1 | weak | 1.67 | 1.54 | 1.80 |  |
|  |  |  |  | male |  | 11 | | 2 | strong | 1.74 | 1.65 | 1.83 |  |
|  |  | Social Relationships and Norms | cardiovascular health-related risk factors | male | Mean (95%CI) | 12 | | 1 | weak | 1.54 | 1.42 | 1.60 |  |
|  |  |  |  | male |  | 12 | | 2 | strong | 1.75 | 1.67 | 1.83 |  |
|  |  | Social Relationships and Norms | cardiovascular health-related risk factors | male | Mean (95%CI) | 13 | | 1 | weak | 1.70 | 1.58 | 1.81 |  |
|  |  |  |  | male |  | 13 | | 2 | strong | 1.75 | 1.65 | 1.84 |  |
|  |  | Social Relationships and Norms | cardiovascular health-related risk factors | male | Mean (95%CI) | 14 | | 1 | weak | 1.79 | 1.67 | 1.91 |  |
|  |  |  |  | male |  | 14 | | 2 | strong | 1.71 | 1.61 | 1.80 |  |
|  |  | Social Relationships and Norms | cardiovascular health-related risk factors | male | Mean (95%CI) | 15 | | 1 | weak | 1.69 | 1.55 | 1.82 |  |
|  |  |  |  | male |  | 15 | | 2 | strong | 1.75 | 1.66 | 1.84 |  |
|  |  | Social Relationships and Norms | cardiovascular health-related risk factors | male | Mean (95%CI) | 16 | | 1 | weak | 1.78 | 1.64 | 1.93 |  |
|  |  |  |  | male |  | 16 | | 2 | strong | 1.71 | 1.63 | 1.80 |  |
|  |  | Social Relationships and Norms | cardiovascular health-related risk factors | male | Mean (95%CI) | 17 | | 1 | weak | 1.72 | 1.60 | 1.85 |  |
|  |  |  |  | male |  | 17 | | 2 | strong | 1.73 | 1.64 | 1.82 |  |
|  |  | Social Relationships and Norms | cardiovascular health-related risk factors | male | Mean (95%CI) | 18 | | 1 | weak | 1.74 | 1.61 | 1.86 |  |
|  |  |  |  | male |  | 18 | | 2 | strong | 1.74 | 1.65 | 1.84 |  |
|  |  | Social Relationships and Norms | cardiovascular health-related risk factors | male | Mean (95%CI) | 19 | | 1 | weak | 1.75 | 1.63 | 1.87 |  |
|  |  |  |  | male |  | 19 | | 2 | strong | 1.72 | 1.62 | 1.81 |  |
|  |  | Social Relationships and Norms | cardiovascular health-related risk factors | male | Mean (95%CI) | 20 | | 1 | weak | 1.82 | 1.68 | 1.96 |  |
|  |  |  |  | male |  | 20 | | 2 | strong | 1.69 | 1.66 | 1.77 |  |
| [76] | Helminen, 1997 | Social Relationships and Norms | metabolic and inflammatory-related risk factors | male | Mean (95%CI) | 1 | | 1 | weak | 3.36 | 3.23 | 3.49 | 0.49 |
|  |  |  |  | male |  | 1 | | 2 | strong | 3.26 | 3.16 | 3.35 | 0.57 |
|  |  | Social Relationships and Norms | metabolic and inflammatory-related risk factors | male | Mean (95%CI) | 2 | | 1 | weak | 3.29 | 3.19 | 3.39 | 0.49 |
|  |  |  |  | male |  | 2 | | 2 | strong | 3.29 | 3.15 | 3.39 | 0.58 |
|  |  | Social Relationships and Norms | metabolic and inflammatory-related risk factors | male | Mean (95%CI) | 3 | | 1 | weak | 3.26 | 3.06 | 3.47 | 0.68 |
|  |  |  |  | male |  | 3 | | 2 | strong | 3.29 | 3.21 | 3.38 | 0.53 |
|  |  | Social Relationships and Norms | metabolic and inflammatory-related risk factors | male | Mean (95%CI) | 4 | | 1 | weak | 3.34 | 3.24 | 3.44 | 0.56 |
|  |  |  |  | male |  | 4 | | 2 | strong | 3.21 | 3.09 | 3.44 | 0.57 |
|  |  | Social Relationships and Norms | metabolic and inflammatory-related risk factors | male | Mean (95%CI) | 5 | | 1 | weak | 3.68 | 3.35 | 3.99 | 0.55 |
|  |  |  |  | male |  | 5 | | 2 | strong | 3.26 | 3.18 | 3.34 | 0.55 |
|  |  | Social Relationships and Norms | metabolic and inflammatory-related risk factors | male | Mean (95%CI) | 6 | | 1 | weak | 3.12 | 2.98 | 3.26 | 0.43 |
|  |  |  |  | male |  | 6 | | 2 | strong | 3.33 | 3.23 | 3.42 | 0.58 |
|  |  | Social Relationships and Norms | metabolic and inflammatory-related risk factors | male | Mean (95%CI) | 7 | | 1 | weak | 3.19 | 3.06 | 3.31 | 0.47 |
|  |  |  |  | male |  | 7 | | 2 | strong | 3.32 | 3.22 | 3.31 | 0.59 |
|  |  | Social Relationships and Norms | metabolic and inflammatory-related risk factors | male | Mean (95%CI) | 8 | | 1 | weak | 3.16 | 3.03 | 3.28 | 0.40 |
|  |  |  |  | male |  | 8 | | 2 | strong | 3.33 | 3.23 | 3.42 | 0.59 |
|  |  | Social Relationships and Norms | metabolic and inflammatory-related risk factors | male | Mean (95%CI) | 9 | | 1 | weak | 3.27 | 3.13 | 3.41 | 0.55 |
|  |  |  |  | male |  | 9 | | 2 | strong | 3.30 | 3.21 | 3.37 | 0.56 |
|  |  | Social Relationships and Norms | metabolic and inflammatory-related risk factors | male | Mean (SE) | 10 | | 1 | weak | 3.60 | - | - | 0.91 |
|  |  |  |  | male |  | 10 | | 2 | strong | 3.70 | - | - | 0.88 |
|  |  | Social Relationships and Norms | metabolic and inflammatory-related risk factors | male | Mean (SE) | 11 | | 1 | weak | 1.20 | - | - | 0.28 |
|  |  |  |  | male |  | 11 | | 2 | strong | 1.20 | - | - | 0.27 |
|  |  | Social Relationships and Norms | metabolic and inflammatory-related risk factors | male | Mean (SE) | 12 | | 1 | weak | 0.70 | - | - | 0.21 |
|  |  |  |  | male |  | 12 | | 2 | strong | 0.70 | - | - | 0.20 |
|  |  | Social Relationships and Norms | metabolic and inflammatory-related risk factors | male | Mean (SE) | 13 | | 1 | weak | 3.80 | - | - | 1.13 |
|  |  |  |  | male |  | 13 | | 2 | strong | 3.70 | - | - | 0.87 |
|  |  | Social Relationships and Norms | metabolic and inflammatory-related risk factors | male | Mean (SE) | 14 | | 1 | weak | 1.10 | - | - | 0.20 |
|  |  |  |  | male |  | 14 | | 2 | strong | 1.20 | - | - | 0.27 |
|  |  | Social Relationships and Norms | metabolic and inflammatory-related risk factors | male | Mean (SE) | 15 | | 1 | weak | 0.10 | - | - | 0.18 |
|  |  |  |  | male |  | 15 | | 2 | strong | 0.60 | - | - | 0.20 |
| [77] | Henning, 2014 | Social Relationships and Norms | glucose metabolism-related risk factors | overall | Beta | 1 | | 1 |  | 0.04 | - | - |  |
|  |  | Social Relationships and Norms | glucose metabolism-related risk factors | overall | Beta | 2 | | 1 |  | -0.11 | - | - |  |
|  |  | Social Relationships and Norms | glucose metabolism-related risk factors | overall | Beta | 3 | | 1 |  | 0.09 | - | - |  |
|  |  | Social Relationships and Norms | glucose metabolism-related risk factors | overall | Beta | 4 | | 1 |  | 0.05 | - | - |  |
|  |  | Social Relationships and Norms | glucose metabolism-related risk factors | overall | Beta | 5 | | 1 |  | -0.09 | - | - |  |
| [78] | Hickson, 2011 | Economic and Social Disadvantage | cardiovascular health-related risk factors | overall | Mean difference (95%CI) | 1 | | 1 |  | 0.18 | −0.70 | 1.06 |  |
|  |  | Economic and Social Disadvantage | cardiovascular health-related risk factors | overall | Mean difference (95%CI) | 2 | | 1 |  | 0.05 | −0.62 | 0.72 |  |
|  |  | Economic and Social Disadvantage | cardiovascular health-related risk factors | overall | OR (95%CI) | 3 | | 1 |  | 0.98 | 0.93 | 1.03 |  |
| [79] | Hilding, 2015 | Social Relationships and Norms | glucose metabolism-related risk factors | female | OR (95%CI) | 1 | | 1 | low (reference) | 1.00 | - | - |  |
|  |  |  |  | female |  | 1 | | 2 | middle | 0.82 | 0.54 | 1.25 |  |
|  |  |  |  | female |  | 1 | | 3 | high | 1.02 | 0.68 | 1.54 |  |
|  |  | Civic Participation and Engagement | glucose metabolism-related risk factors | female | OR (95%CI) | 2 | | 1 | no (reference) | 1.00 | - | - |  |
|  |  |  |  | female |  | 2 | | 2 | yes | 0.78 | 0.54 | 1.12 |  |
|  |  | Social Relationships and Norms | glucose metabolism-related risk factors | male | OR (95%CI) | 3 | | 1 | low (reference) | 1.00 | - | - |  |
|  |  |  |  | male |  | 3 | | 2 | middle | 1.08 | 0.76 | 1.55 |  |
|  |  |  |  | male |  | 3 | | 3 | high | 1.33 | 0.93 | 1.92 |  |
|  |  | Civic Participation and Engagement | glucose metabolism-related risk factors | male | OR (95%CI) | 4 | | 1 | no (reference) | 1.00 | - | - |  |
|  |  |  |  | male |  | 4 | | 2 | yes | 0.59 | 0.43 | 0.82 |  |
| [80] | Holmes, 2012 | Economic and Social Disadvantage | metabolic and inflammatory-related risk factors | overall | Beta (SE) | 1 | | 1 |  | −0.67 | - | - | 1.72 |
|  |  | Economic and Social Disadvantage | metabolic and inflammatory-related risk factors | overall | Beta (SE) | 2 | | 1 |  | 3.10 | - | - | 1.56 |
|  |  | Social Relationships and Norms | metabolic and inflammatory-related risk factors | overall | Beta (SE) | 3 | | 1 |  | −0.13 | - | - | 0.21 |
| [81] | Horsten, 1999 | Social Relationships and Norms | CVD risk scores | female | RR (95%CI) | 12 | | 1 | low social support | 3.49 | 1.07 | 11.37 |  |
|  |  |  |  | female |  | 12 | | 2 | intermediate social support | 2.19 | 0.67 | 7.20 |  |
|  |  |  |  | female |  | 12 | | 3 | high social support (reference) | 1.00 | - | - |  |
|  |  | Social Relationships and Norms | CVD risk scores | female | RR (95%CI) | 13 | | 1 |  | 1.40 | 1.10 | 1.70 |  |
| [82] | Hosseini, 2020 | Social Relationships and Norms | cardiovascular health-related risk factors | female | Beta | 1 | | 1 | Q4,largest, reference | - | - | - |  |
|  |  |  |  | female |  | 1 | | 2 | Q3 | 0.43 | -0.32 | 1.18 |  |
|  |  |  |  | female |  | 1 | | 3 | Q2 | 0.26 | -0.50 | 1.01 |  |
|  |  |  |  | female |  | 1 | | 4 | Q1,smallest | 1.57 | 0.76 | 2.38 |  |
|  |  | Social Relationships and Norms | cardiovascular health-related risk factors | male | Beta | 2 | | 1 | Q4,largest, reference | - | - | - |  |
|  |  |  |  | male |  | 2 | | 2 | Q3 | -0.33 | -1.04 | 0.37 |  |
|  |  |  |  | male |  | 2 | | 3 | Q2 | -0.66 | -1.39 | 0.08 |  |
|  |  |  |  | male |  | 2 | | 4 | Q1,smallest | -1.11 | -1.89 | -0.34 |  |
|  |  | Civic Participation and Engagement | cardiovascular health-related risk factors | female | Beta | 3 | | 1 | a lot, reference | - | - | - |  |
|  |  |  |  | female |  | 3 | | 2 | some | 0.10 | -0.49 | 0.68 |  |
|  |  |  |  | female |  | 3 | | 3 | a few | 1.31 | 0.48 | 2.13 |  |
|  |  |  |  | female |  | 3 | | 4 | none | 2.03 | -0.63 | 4.68 |  |
|  |  | Civic Participation and Engagement | cardiovascular health-related risk factors | male | Beta | 4 | | 1 | a lot, reference | - | - | - |  |
|  |  |  |  | male |  | 4 | | 2 | some | 0.27 | -0.32 | 0.86 |  |
|  |  |  |  | male |  | 4 | | 3 | a few | 0.19 | -0.59 | 0.97 |  |
|  |  |  |  | male |  | 4 | | 4 | none | -0.25 | -2.47 | 1.98 |  |
|  |  | Social Relationships and Norms | cardiovascular health-related risk factors | female | Beta | 5 | | 1 | Q4,largest, reference | - | - | - |  |
|  |  |  |  | female |  | 5 | | 2 | Q3 | 0.29 | -0.16 | 0.73 |  |
|  |  |  |  | female |  | 5 | | 3 | Q2 | 0.35 | -0.10 | 0.79 |  |
|  |  |  |  | female |  | 5 | | 4 | Q1,smallest | 1.25 | 0.78 | 1.72 |  |
|  |  | Social Relationships and Norms | cardiovascular health-related risk factors | male | Beta | 6 | | 1 | Q4,largest, reference | - | - | - |  |
|  |  |  |  | male |  | 6 | | 2 | Q3 | -0.11 | -0.54 | 0.33 |  |
|  |  |  |  | male |  | 6 | | 3 | Q2 | -0.28 | -0.73 | 0.17 |  |
|  |  |  |  | male |  | 6 | | 4 | Q1,smallest | -0.36 | -0.83 | 0.11 |  |
|  |  | Civic Participation and Engagement | cardiovascular health-related risk factors | female | Beta | 7 | | 1 | a lot, reference | - | - | - |  |
|  |  |  |  | female |  | 7 | | 2 | some | -0.19 | -0.53 | 0.15 |  |
|  |  |  |  | female |  | 7 | | 3 | a few | 0.39 | -0.09 | 0.87 |  |
|  |  |  |  | female |  | 7 | | 4 | none | 0.81 | -0.70 | 2.32 |  |
|  |  | Civic Participation and Engagement | cardiovascular health-related risk factors | male | Beta | 8 | | 1 | a lot, reference | - | - | - |  |
|  |  |  |  | male |  | 8 | | 2 | some | 0.00 | -0.36 | 0.35 |  |
|  |  |  |  | male |  | 8 | | 3 | a few | -0.20 | -0.67 | 0.27 |  |
|  |  |  |  | male |  | 8 | | 4 | none | 0.05 | -1.20 | 1.30 |  |
| [83] | Hughes, 2007 | Social Relationships and Norms | cardiovascular health-related risk factors | female | Beta (SE) | 1 | | 1 |  | -0.03 | - | - | 0.52 |
|  |  | Social Relationships and Norms | cardiovascular health-related risk factors | female | Beta (SE) | 2 | | 1 |  | -0.08 | - | - | 0.37 |
|  |  | Social Relationships and Norms | cardiovascular health-related risk factors | female | Beta (SE) | 3 | | 1 |  | -0.06 | - | - | 0.41 |
|  |  | Social Relationships and Norms | cardiovascular health-related risk factors | female | Beta (SE) | 4 | | 1 |  | -3.04 | - | - | 1.36 |
|  |  | Social Relationships and Norms | cardiovascular health-related risk factors | female | Beta (SE) | 5 | | 1 |  | -0.44 | - | - | 0.97 |
|  |  | Social Relationships and Norms | cardiovascular health-related risk factors | female | Beta (SE) | 6 | | 1 |  | 2.33 | - | - | 1.10 |
| [84] | Islam, 2020 | composite of multiple dimensions - not classified | CVD risk scores | overall | OR (95%CI) | 1 | | 1 |  | 1.11 | 0.86 | 1.43 |  |
|  |  | composite of multiple dimensions - not classified | CVD risk scores | overall | OR (95%CI) | 2 | | 1 |  | 1.62 | 1.11 | 2.36 |  |
|  |  | Social Cohesion and Social Capital | CVD risk scores | overall | OR (95%CI) | 9 | | 1 |  | 2.02 | 1.36 | 3.01 |  |
|  |  | Social Cohesion and Social Capital | CVD risk scores | overall | OR (95%CI) | 10 | | 1 |  | 1.71 | 1.20 | 2.45 |  |
|  |  | composite of multiple dimensions - not classified | CVD risk scores | female | OR (95%CI) | 11 | | 1 |  | 1.30 | 0.94 | 1.82 |  |
|  |  | Crime and Safety | CVD risk scores | female | OR (95%CI) | 12 | | 1 |  | 1.39 | 1.00 | 1.92 |  |
|  |  | Social Cohesion and Social Capital | CVD risk scores | female | OR (95%CI) | 13 | | 1 |  | 1.23 | 0.88 | 1.75 |  |
|  |  | Civic Participation and Engagement | CVD risk scores | female | OR (95%CI) | 14 | | 1 |  | 1.04 | 0.77 | 1.42 |  |
|  |  | Crime and Safety | CVD risk scores | female | OR (95%CI) | 15 | | 1 |  | 0.85 | 0.60 | 1.18 |  |
|  |  | composite of multiple dimensions - not classified | CVD risk scores | female | OR (95%CI) | 16 | | 1 |  | 1.57 | 0.98 | 2.52 |  |
|  |  | Crime and Safety | CVD risk scores | female | OR (95%CI) | 17 | | 1 |  | 1.36 | 0.88 | 2.11 |  |
|  |  | Social Cohesion and Social Capital | CVD risk scores | female | OR (95%CI) | 18 | | 1 |  | 2.61 | 1.48 | 4.61 |  |
|  |  | Civic Participation and Engagement | CVD risk scores | female | OR (95%CI) | 19 | | 1 |  | 1.82 | 1.15 | 2.86 |  |
|  |  | Crime and Safety | CVD risk scores | female | OR (95%CI) | 20 | | 1 |  | 0.91 | 0.59 | 1.42 |  |
|  |  | composite of multiple dimensions - not classified | CVD risk scores | male | OR (95%CI) | 21 | | 1 |  | 0.88 | 0.59 | 1.32 |  |
|  |  | Crime and Safety | CVD risk scores | male | OR (95%CI) | 22 | | 1 |  | 0.93 | 0.63 | 1.39 |  |
|  |  | Social Cohesion and Social Capital | CVD risk scores | male | OR (95%CI) | 23 | | 1 |  | 0.81 | 0.56 | 1.19 |  |
|  |  | Civic Participation and Engagement | CVD risk scores | male | OR (95%CI) | 24 | | 1 |  | 0.90 | 0.59 | 1.36 |  |
|  |  | Crime and Safety | CVD risk scores | male | OR (95%CI) | 25 | | 1 |  | 1.10 | 0.73 | 1.64 |  |
|  |  | composite of multiple dimensions - not classified | CVD risk scores | male | OR (95%CI) | 26 | | 1 |  | 1.64 | 0.88 | 3.05 |  |
|  |  | Crime and Safety | CVD risk scores | male | OR (95%CI) | 27 | | 1 |  | 1.52 | 0.85 | 2.69 |  |
|  |  | Social Cohesion and Social Capital | CVD risk scores | male | OR (95%CI) | 28 | | 1 |  | 1.40 | 0.82 | 2.40 |  |
|  |  | Civic Participation and Engagement | CVD risk scores | male | OR (95%CI) | 29 | | 1 |  | 1.53 | 0.84 | 2.78 |  |
|  |  | Crime and Safety | CVD risk scores | male | OR (95%CI) | 30 | | 1 |  | 0.64 | 0.35 | 1.17 |  |
| [85] | Jimenez, 2019 | Economic and Social Disadvantage | cardiovascular health-related risk factors | overall | Beta | 1 | | 1 | stayed in low neighborhood socioeconomic status (reference) | - | - | - |  |
|  |  |  |  | overall |  | 1 | | 2 | fluctuated | −3.37 | −6.40 | −0.35 |  |
|  |  |  |  | overall |  | 1 | | 3 | stayed in high neighborhood socioeconomic status | −2.51 | −6.12 | 1.10 |  |
|  |  | Economic and Social Disadvantage | cardiovascular health-related risk factors | overall | Beta | 2 | | 1 | stayed in low neighborhood socioeconomic status (reference) | - | - | - |  |
|  |  |  |  | overall |  | 2 | | 2 | fluctuated | −1.35 | −3.46 | 0.76 |  |
|  |  |  |  | overall |  | 2 | | 3 | stayed in high neighborhood socioeconomic status | −1.27 | −3.79 | 1.25 |  |
| [86] | Kakinami, 2017 | Economic and Social Disadvantage | CVD risk scores | male | HR (95%CI) | 1 | | 1 |  | 2.40 | 1.49 | 3.85 |  |
|  |  | Economic and Social Disadvantage | CVD risk scores | female | HR (95%CI) | 2 | | 1 |  | 1.77 | 1.31 | 2.39 |  |
| [87] | Keita, 2014 | Economic and Social Disadvantage | CVD risk scores | overall | OR (95%CI) | 4 | | 1 | Q1 | 1.19 | 0.91 | 1.57 |  |
|  |  |  |  | overall |  | 4 | | 2 | Q2 | 1.19 | 0.90 | 1.57 |  |
|  |  |  |  | overall |  | 4 | | 3 | Q3 | 1.18 | 0.89 | 1.57 |  |
|  |  |  |  | overall |  | 4 | | 4 | Q4 | 0.96 | 0.71 | 1.29 |  |
|  |  |  |  | overall |  | 4 | | 5 | Q5 (reference) | 1.00 | - | - |  |
|  |  | Economic and Social Disadvantage | metabolic and inflammatory-related risk factors | overall | OR (95%CI) | 5 | | 1 | Q1 | 1.36 | 1.09 | 1.70 |  |
|  |  |  |  | overall |  | 5 | | 2 | Q2 | 1.23 | 0.98 | 1.54 |  |
|  |  |  |  | overall |  | 5 | | 3 | Q3 | 1.21 | 0.97 | 1.52 |  |
|  |  |  |  | overall |  | 5 | | 4 | Q4 | 1.17 | 0.93 | 1.48 |  |
|  |  |  |  | overall |  | 5 | | 5 | Q5 (reference) | 1.00 | - | - |  |
|  |  | Economic and Social Disadvantage | CVD risk scores | overall | OR (95%CI) | 6 | | 1 | Q1 | 1.52 | 1.20 | 1.92 |  |
|  |  |  |  | overall |  | 6 | | 2 | Q2 | 1.39 | 1.10 | 1.77 |  |
|  |  |  |  | overall |  | 6 | | 3 | Q3 | 1.36 | 1.07 | 1.74 |  |
|  |  |  |  | overall |  | 6 | | 4 | Q4 | 1.13 | 0.88 | 1.45 |  |
|  |  |  |  | overall |  | 6 | | 5 | Q5 (reference) | 1.00 | - | - |  |
|  |  | Economic and Social Disadvantage | CVD risk scores | overall | OR (95%CI) | 10 | | 1 | Q1 | 1.78 | 1.50 | 2.12 |  |
|  |  |  |  | overall |  | 10 | | 2 | Q2 | 1.62 | 1.39 | 1.88 |  |
|  |  |  |  | overall |  | 10 | | 3 | Q3 | 1.56 | 1.36 | 1.79 |  |
|  |  |  |  | overall |  | 10 | | 4 | Q4 | 1.38 | 1.21 | 1.57 |  |
|  |  |  |  | overall |  | 10 | | 5 | Q5 (reference) | 1.00 | - | - |  |
|  |  | Economic and Social Disadvantage | metabolic and inflammatory-related risk factors | overall | OR (95%CI) | 11 | | 1 | Q1 | 1.25 | 1.07 | 1.45 |  |
|  |  |  |  | overall |  | 11 | | 2 | Q2 | 1.37 | 1.21 | 1.56 |  |
|  |  |  |  | overall |  | 11 | | 3 | Q3 | 1.19 | 1.06 | 1.35 |  |
|  |  |  |  | overall |  | 11 | | 4 | Q4 | 1.17 | 1.05 | 1.31 |  |
|  |  |  |  | overall |  | 11 | | 5 | Q5 (reference) | 1.00 | - | - |  |
|  |  | Economic and Social Disadvantage | CVD risk scores | overall | OR (95%CI) | 12 | | 1 | Q1 | 1.65 | 1.41 | 1.93 |  |
|  |  |  |  | overall |  | 12 | | 2 | Q2 | 1.67 | 1.46 | 1.91 |  |
|  |  |  |  | overall |  | 12 | | 3 | Q3 | 1.55 | 1.37 | 1.75 |  |
|  |  |  |  | overall |  | 12 | | 4 | Q4 | 1.37 | 1.22 | 1.54 |  |
|  |  |  |  | overall |  | 12 | | 5 | Q5 (reference) | 1.00 | - | - |  |
| [88] | Kelli, 2017 | Economic and Social Disadvantage | metabolic and inflammatory-related risk factors | overall | Beta (95%CI) | 1 | | 1 |  | 0.10 | −0.04 | 0.24 |  |
|  |  | Economic and Social Disadvantage | cardiovascular health-related risk factors | female | Beta (95%CI) | 2 | | 1 |  | 0.30 | −0.78 | 1.38 |  |
|  |  | Economic and Social Disadvantage | cardiovascular health-related risk factors | female | Beta (95%CI) | 3 | | 1 |  | 0.14 | −0.07 | 0.34 |  |
|  |  | Economic and Social Disadvantage | metabolic and inflammatory-related risk factors | female | Beta (95%CI) | 4 | | 1 |  | −0.09 | −0.17 | −0.01 |  |
|  |  | Economic and Social Disadvantage | metabolic and inflammatory-related risk factors | female | Beta (95%CI) | 5 | | 1 |  | −0.42 | −2.74 | 1.90 |  |
|  |  | Economic and Social Disadvantage | cardiovascular health-related risk factors | female | Beta (95%CI) | 6 | | 1 |  | 0.14 | 0.02 | 0.25 |  |
|  |  | Economic and Social Disadvantage | CVD risk scores | female | Prevalence | 8 | | 1 | low income area | 30.10 | - | - |  |
|  |  |  |  | female |  | 8 | | 2 | high income area | 27.40 | - | - |  |
| [89] | Kent de Grey, 2019 | Social Relationships and Norms | cardiovascular health-related risk factors | overall | Beta (SE) | 1 | | 1 |  | -0.01 | -0.10 | 0.07 | 0.04 |
|  |  | Social Relationships and Norms | cardiovascular health-related risk factors | overall | Beta (SE) | 2 | | 1 |  | 0.08 | -0.02 | 0.19 | 0.05 |
|  |  | Social Relationships and Norms | cardiovascular health-related risk factors | overall | Beta (SE) | 3 | | 1 |  | 0.01 | -0.04 | -0.05 | 0.02 |
|  |  | Social Relationships and Norms | cardiovascular health-related risk factors | overall | Beta (SE) | 4 | | 1 |  | 0.06 | 0.00 | 0.12 | 0.03 |
|  |  | Social Relationships and Norms | cardiovascular health-related risk factors | overall | Beta (SE) | 5 | | 1 |  | 0.02 | -0.03 | 0.07 | 0.03 |
|  |  | Social Relationships and Norms | cardiovascular health-related risk factors | overall | Beta (SE) | 6 | | 1 |  | 0.07 | 0.01 | 0.13 | 0.03 |
| [90] | Kershaw, 2017 | Discrimination and Segregation | cardiovascular health-related risk factors | overall | Beta (95%CI) | 1 | | 1 |  | 0.14 | 0.03 | 0.26 |  |
|  |  | Discrimination and Segregation | cardiovascular health-related risk factors | overall | Beta (95%CI) | 2 | | 1 |  | 0.03 | −0.07 | 0.12 |  |
|  |  | Discrimination and Segregation | cardiovascular health-related risk factors | overall | Beta | 3 | | 1 | High segregation | - | - | - |  |
|  |  |  |  | overall |  | 3 | | 2 | Medium segregation | −1.22 | −2.14 | −0.30 |  |
|  |  |  |  | overall |  | 3 | | 3 | Low segregation | −1.38 | −2.40 | −0.37 |  |
|  |  | Discrimination and Segregation | cardiovascular health-related risk factors | overall | Beta | 4 | | 1 | High segregation | - | - | - |  |
|  |  |  |  | overall |  | 4 | | 2 | Medium segregation | 0.33 | −0.39 | 1.06 |  |
|  |  |  |  | overall |  | 4 | | 3 | Low segregation | 0.54 | −0.27 | 1.34 |  |
| [91] | Kim, 2010 | Economic and Social Disadvantage | cardiovascular health-related risk factors | female | OR (95%CI) | 1 | | 1 | 1 least socioeconomically deprived (reference) | 1.00 | - | - | - |
|  |  |  |  | female |  | 1 | | 2 | 2 | 1.81 | 1.06 | 3.11 |  |
|  |  |  |  | female |  | 1 | | 3 | 3 | 1.44 | 0.79 | 2.63 |  |
|  |  |  |  | female |  | 1 | | 4 | 4 | 2.49 | 1.22 | 5.08 |  |
|  |  | Social Cohesion and Social Capital | cardiovascular health-related risk factors | female | OR (95%CI) | 2 | | 1 | 1 highest perceived level of neighborhood cohesion (reference) | 1.00 | - | - |  |
|  |  |  |  | female |  | 2 | | 2 | 2 | 1.24 | 0.73 | 2.11 |  |
|  |  |  |  | female |  | 2 | | 3 | 3 | 1.47 | 0.89 | 2.43 |  |
|  |  |  |  | female |  | 2 | | 4 | 4 | 1.87 | 1.10 | 3.16 |  |
|  |  | Economic and Social Disadvantage | cardiovascular health-related risk factors | male | OR (95%CI) | 3 | | 1 | 1 least socioeconomically deprived (reference) | 1.00 | - | - |  |
|  |  |  |  | male |  | 3 | | 2 | 2 | 0.75 | 0.51 | 1.10 |  |
|  |  |  |  | male |  | 3 | | 3 | 3 | 1.00 | 0.65 | 1.54 |  |
|  |  |  |  | male |  | 3 | | 4 | 4 | 1.18 | 0.67 | 2.10 |  |
|  |  | Social Cohesion and Social Capital | cardiovascular health-related risk factors | male | OR (95%CI) | 4 | | 1 | 1 highest perceived level of neighborhood cohesion (reference) | 1.00 | - | - |  |
|  |  |  |  | male |  | 4 | | 2 | 2 | 1.54 | 1.03 | 2.30 |  |
|  |  |  |  | male |  | 4 | | 3 | 3 | 1.46 | 0.98 | 2.16 |  |
|  |  |  |  | male |  | 4 | | 4 | 4 | 1.45 | 0.94 | 2.22 |  |
| [92] | Kim, 2020 | Social Relationships and Norms | CVD risk scores | male | OR (95%CI) | 1 | | 1 | Large (≥4) | 1.00 | - | - |  |
|  |  |  |  | male |  | 1 | | 2 | Small (≤3) | 1.25 | 1.05 | 1.48 |  |
|  |  | Social Relationships and Norms | CVD risk scores | male | OR (95%CI) | 2 | | 1 |  | 1.07 | 1.02 | 1.12 |  |
|  |  | Social Relationships and Norms | CVD risk scores | male | OR (95%CI) | 3 | | 1 | High (≥3.2) | 1.00 | - | - |  |
|  |  |  |  | male |  | 3 | | 2 | Low (<3.2) | 1.10 | 0.93 | 1.30 |  |
|  |  | Social Relationships and Norms | CVD risk scores | male | OR (95%CI) | 4 | | 1 |  | 1.00 | 0.88 | 1.15 |  |
|  |  | Social Relationships and Norms | CVD risk scores | female | OR (95%CI) | 5 | | 1 | Large (≥4) | 1.00 | - | - |  |
|  |  |  |  | female |  | 5 | | 2 | Small (≤3) | 1.32 | 1.14 | 1.53 |  |
|  |  | Social Relationships and Norms | CVD risk scores | female | OR (95%CI) | 6 | | 1 |  | 1.10 | 1.05 | 1.15 |  |
|  |  | Social Relationships and Norms | CVD risk scores | female | OR (95%CI) | 7 | | 1 | High (≥3.2) | 1.00 | - | - |  |
|  |  |  |  | female |  | 7 | | 2 | Low (<3.2) | 0.90 | 0.78 | 1.04 |  |
|  |  | Social Relationships and Norms | CVD risk scores | female | OR (95%CI) | 8 | | 1 |  | 0.93 | 0.83 | 1.05 |  |
|  |  | Social Relationships and Norms | CVD risk scores | male | OR (95%CI) | 9 | | 1 | Large (≥4) | 1.00 | - | - |  |
|  |  |  |  | male |  | 9 | | 2 | Small (≤3) | 0.98 | 0.71 | 1.36 |  |
|  |  | Social Relationships and Norms | CVD risk scores | male | OR (95%CI) | 10 | | 1 |  | 0.85 | 0.87 | 1.03 |  |
|  |  | Social Relationships and Norms | CVD risk scores | male | OR (95%CI) | 11 | | 1 | High (≥3.2) | 1.00 | - | - |  |
|  |  |  |  | male |  | 11 | | 2 | Low (<3.2) | 1.02 | 0.80 | 1.30 |  |
|  |  | Social Relationships and Norms | CVD risk scores | male | OR (95%CI) | 12 | | 1 |  | 1.06 | 0.88 | 1.27 |  |
|  |  | Social Relationships and Norms | CVD risk scores | female | OR (95%CI) | 13 | | 1 | Large (≥4) | 1.00 | - | - |  |
|  |  |  |  | female |  | 13 | | 2 | Small (≤3) | 1.40 | 0.97 | 2.00 |  |
|  |  | Social Relationships and Norms | CVD risk scores | female | OR (95%CI) | 14 | | 1 |  | 1.08 | 0.97 | 1.19 |  |
|  |  | Social Relationships and Norms | CVD risk scores | female | OR (95%CI) | 15 | | 1 | High (≥3.2) | 1.00 | - | - |  |
|  |  |  |  | female |  | 15 | | 2 | Low (<3.2) | 1.16 | 0.86 | 1.56 |  |
|  |  | Social Relationships and Norms | CVD risk scores | female | OR (95%CI) | 16 | | 1 |  | 1.12 | 0.91 | 1.39 |  |
| [93] | King, 2011 | Economic and Social Disadvantage | CVD risk scores | overall | IRR (SE) | 1 | | 1 |  | 1.05 | - | - | 0.09 |
|  |  | Economic and Social Disadvantage | CVD risk scores | overall | IRR (SE) | 2 | | 1 |  | 0.82 | - | - | 0.09 |
| [94] | Kivimäki, 2018 | Economic and Social Disadvantage | cardiovascular health-related risk factors | overall | OR (95%CI) | 1 | | 1 | low (<= -0.5SD) | 1.00 | - | - |  |
|  |  |  |  | overall |  | 1 | | 2 | low intermediate (-0.5 to 0 SD) | 0.73 | 0.40 | 1.33 |  |
|  |  |  |  | overall |  | 1 | | 3 | high intermediate (>0 to 0.5 SD) | 0.68 | 0.35 | 1.32 |  |
|  |  |  |  | overall |  | 1 | | 4 | high (>= 0.5SD) | 0.91 | 0.43 | 1.95 |  |
|  |  | Economic and Social Disadvantage | cardiovascular health-related risk factors | overall | Mean difference | 2 | | 1 | low (<= -0.5SD) | 0.00 | - | - |  |
|  |  |  |  | overall |  | 2 | | 2 | low intermediate (-0.5 to 0 SD) | 0.20 | –0.59 | 0.98 |  |
|  |  |  |  | overall |  | 2 | | 3 | high intermediate (>0 to 0.5 SD) | 0.40 | –0.49 | 1.28 |  |
|  |  |  |  | overall |  | 2 | | 4 | high (>= 0.5SD) | 0.14 | –0.99 | 1.27 |  |
| [95] | Lawlor, 2005 | Economic and Social Disadvantage | cardiovascular health-related risk factors | female | Mean (95%CI) | 1 | | 1 | 1 (Most Affluent) | 146.60 | 144.90 | 148.20 |  |
|  |  |  |  | female |  | 1 | | 2 | 2 | 145.10 | 143.30 | 146.90 |  |
|  |  |  |  | female |  | 1 | | 3 | 3 | 147.70 | 145.90 | 149.50 |  |
|  |  |  |  | female |  | 1 | | 4 | 4 | 147.50 | 145.70 | 149.30 |  |
|  |  |  |  | female |  | 1 | | 5 | 5 (Most Deprived) | 148.90 | 147.10 | 150.60 |  |
|  |  | Economic and Social Disadvantage | metabolic and inflammatory-related risk factors | female | Mean (95%CI) | 2 | | 1 | 1 (Most Affluent) | 1.72 | 1.69 | 1.75 |  |
|  |  |  |  | female |  | 2 | | 2 | 2 | 1.75 | 1.71 | 1.78 |  |
|  |  |  |  | female |  | 2 | | 3 | 3 | 1.63 | 1.60 | 1.66 |  |
|  |  |  |  | female |  | 2 | | 4 | 4 | 1.60 | 1.57 | 1.63 |  |
|  |  |  |  | female |  | 2 | | 5 | 5 (Most Deprived) | 1.57 | 1.53 | 1.60 |  |
|  |  | Economic and Social Disadvantage | metabolic and inflammatory-related risk factors | female | Mean (95%CI) | 3 | | 1 | 1 (Most Affluent) | 1.61 | 1.56 | 1.66 |  |
|  |  |  |  | female |  | 3 | | 2 | 2 | 1.59 | 1.54 | 1.64 |  |
|  |  |  |  | female |  | 3 | | 3 | 3 | 1.67 | 1.61 | 1.72 |  |
|  |  |  |  | female |  | 3 | | 4 | 4 | 1.72 | 1.66 | 1.77 |  |
|  |  |  |  | female |  | 3 | | 5 | 5 (Most Deprived) | 1.79 | 1.73 | 1.84 |  |
|  |  | Economic and Social Disadvantage | glucose metabolism-related risk factors | female | Mean (95%CI) | 4 | | 1 | 1 (Most Affluent) | 1.50 | 1.43 | 1.57 |  |
|  |  |  |  | female |  | 4 | | 2 | 2 | 1.65 | 1.57 | 1.73 |  |
|  |  |  |  | female |  | 4 | | 3 | 3 | 1.67 | 1.59 | 1.75 |  |
|  |  |  |  | female |  | 4 | | 4 | 4 | 1.73 | 1.65 | 1.81 |  |
|  |  |  |  | female |  | 4 | | 5 | 5 (Most Deprived) | 1.80 | 1.72 | 1.89 |  |
| [96] | Lee, 2020 | Social Relationships and Norms | cardiovascular health-related risk factors | overall | Beta (SE) | 1 | | 1 |  | -1.11 | - | - | 0.49 |
|  |  | Social Relationships and Norms | cardiovascular health-related risk factors | overall | Beta (SE) | 2 | | 1 |  | -0.66 | - | - | 0.38 |
|  |  | Social Relationships and Norms | cardiovascular health-related risk factors | overall | Beta (SE) | 3 | | 1 |  | -0.03 | - | - | 0.63 |
|  |  | Social Relationships and Norms | cardiovascular health-related risk factors | overall | Beta (SE) | 4 | | 1 |  | -0.33 | - | - | 0.50 |
| [97] | Lei, 2018 | Economic and Social Disadvantage | CVD risk scores | overall | Beta (SE) | 1 | | 1 |  | 0.72 | - | - | 0.23 |
|  |  | Discrimination and Segregation | CVD risk scores | overall | Beta (SE) | 2 | | 1 |  | 0.54 | - | - | 0.94 |
|  |  | Economic and Social Disadvantage | CVD risk scores | overall | Beta (SE) | 3 | | 1 |  | 0.71 | - | - | 0.22 |
|  |  | Discrimination and Segregation | CVD risk scores | overall | Beta (SE) | 4 | | 1 |  | 0.57 | - | - | 0.92 |
| [98] | Lemelin, 2009 | Economic and Social Disadvantage | cardiovascular health-related risk factors | male | Mean difference | 1 | | 1 | neighborhood socioeconomic position -low (reference) | - | - | - |  |
|  |  |  |  | male |  | 1 | | 2 | neighborhood socioeconomic position-med | 21.10 | 1.50 | 40.80 |  |
|  |  |  |  | male |  | 1 | | 3 | neighborhood socioeconomic position-high | 2.00 | −19.20 | 23.20 |  |
|  |  | Economic and Social Disadvantage | cardiovascular health-related risk factors | female | Mean difference | 2 | | 1 | neighborhood socioeconomic position -low (reference) | - | - | - |  |
|  |  |  |  | female |  | 2 | | 2 | neighborhood socioeconomic position-med | −7.10 | −23.80 | 9.60 |  |
|  |  |  |  | female |  | 2 | | 3 | neighborhood socioeconomic position-high | −13.70 | −32.10 | 4.70 |  |
| [99] | Lewis, 2010 | Social Relationships and Norms | cardiovascular health-related risk factors | overall | Beta (SE) | 1 | | 1 |  | 0.01 | - | - | 0.02 |
| [100] | Li, 2019 | Discrimination and Segregation | CVD risk scores | overall | OR (95%CI) | 1 | | 1 |  | 0.71 | 0.52 | 0.96 |  |
|  |  | Discrimination and Segregation | CVD risk scores | female | OR (95%CI) | 2 | | 1 |  | 0.68 | 0.45 | 1.02 |  |
|  |  | Discrimination and Segregation | CVD risk scores | male | OR (95%CI) | 3 | | 1 |  | 0.80 | 0.53 | 1.21 |  |
| [101] | Li, 2017 | Discrimination and Segregation | metabolic and inflammatory-related risk factors | overall | OR (95%CI) | 1 | | 1 |  | 1.48 | 1.02 | 2.14 |  |
|  |  | Discrimination and Segregation | metabolic and inflammatory-related risk factors | overall | OR (95%CI) | 2 | | 1 | immigrant concentration (low; reference) | 1.00 | - | - |  |
|  |  |  |  | overall |  | 2 | | 2 | immigrant concentration (medium) | 0.79 | 0.55 | 1.14 |  |
|  |  |  |  | overall |  | 2 | | 3 | immigrant concentration (high) | 0.70 | 0.49 | 1.00 |  |
| [102] | Linden, 1993 | Social Relationships and Norms | cardiovascular health-related risk factors | female | Beta | 1 | | 1 |  | -0.22 | - | - |  |
|  |  | Social Relationships and Norms | cardiovascular health-related risk factors | female | Beta | 2 | | 1 |  | -0.07 | - | - |  |
| [103] | Lippert, 2017 | Economic and Social Disadvantage | cardiovascular health-related risk factors | overall | Beta (95%CI) | 1 | | 1 | Consistently lived in poor neighborhoods vs. Never lived in poor neighborhood | 1.77 | 0.53 | 3.02 |  |
|  |  | Economic and Social Disadvantage | cardiovascular health-related risk factors | overall | Beta (95%CI) | 2 | | 1 | Consistently lived in poor neighborhoods vs. Never lived in poor neighborhood | 1.73 | 0.82 | 2.64 |  |
|  |  | Economic and Social Disadvantage | cardiovascular health-related risk factors | overall | Beta (95%CI) | 3 | | 1 | Consistently lived in poor neighborhoods vs. Never lived in poor neighborhood | 0.10 | −0.08 | 0.28 |  |
|  |  | Economic and Social Disadvantage | metabolic and inflammatory-related risk factors | overall | Beta (95%CI) | 4 | | 1 | Consistently lived in poor neighborhoods vs. Never lived in poor neighborhood | −0.09 | −0.36 | 0.19 |  |
|  |  | Economic and Social Disadvantage | metabolic and inflammatory-related risk factors | overall | Beta (95%CI) | 5 | | 1 | Consistently lived in poor neighborhoods vs. Never lived in poor neighborhood | −0.07 | −0.35 | 0.22 |  |
|  |  | Economic and Social Disadvantage | metabolic and inflammatory-related risk factors | overall | Beta (95%CI) | 6 | | 1 | Consistently lived in poor neighborhoods vs. Never lived in poor neighborhood | 0.09 | −0.17 | 0.35 |  |
|  |  | Economic and Social Disadvantage | cardiovascular health-related risk factors | overall | Beta (95%CI) | 7 | | 1 | Entered neighborhood poverty vs. Never lived in poor neighborhood | 1.08 | 0.07 | 2.10 |  |
|  |  | Economic and Social Disadvantage | cardiovascular health-related risk factors | overall | Beta (95%CI) | 8 | | 1 | Entered neighborhood poverty vs. Never lived in poor neighborhood | 1.08 | 0.32 | 1.85 |  |
|  |  | Economic and Social Disadvantage | cardiovascular health-related risk factors | overall | Beta (95%CI) | 9 | | 1 | Entered neighborhood poverty vs. Never lived in poor neighborhood | 0.14 | −0.04 | 0.33 |  |
|  |  | Economic and Social Disadvantage | metabolic and inflammatory-related risk factors | overall | Beta (95%CI) | 10 | | 1 | Entered neighborhood poverty vs. Never lived in poor neighborhood | −0.13 | −0.37 | 0.12 |  |
|  |  | Economic and Social Disadvantage | metabolic and inflammatory-related risk factors | overall | Beta (95%CI) | 11 | | 1 | Entered neighborhood poverty vs. Never lived in poor neighborhood | −0.10 | −0.34 | 0.14 |  |
|  |  | Economic and Social Disadvantage | metabolic and inflammatory-related risk factors | overall | Beta (95%CI) | 12 | | 1 | Entered neighborhood poverty vs. Never lived in poor neighborhood | −0.08 | −0.32 | 0.15 |  |
|  |  | Economic and Social Disadvantage | cardiovascular health-related risk factors | overall | Beta (95%CI) | 13 | | 1 | Entered neighborhood poverty vs. Consistently lived in poor neighborhoods | −0.69 | −2.14 | 0.76 |  |
|  |  | Economic and Social Disadvantage | cardiovascular health-related risk factors | overall | Beta (95%CI) | 14 | | 1 | Entered neighborhood poverty vs. Consistently lived in poor neighborhoods | −0.65 | −1.71 | 0.41 |  |
|  |  | Economic and Social Disadvantage | cardiovascular health-related risk factors | overall | Beta (95%CI) | 15 | | 1 | Entered neighborhood poverty vs. Consistently lived in poor neighborhoods | 0.04 | −0.18 | 0.27 |  |
|  |  | Economic and Social Disadvantage | metabolic and inflammatory-related risk factors | overall | Beta (95%CI) | 16 | | 1 | Entered neighborhood poverty vs. Consistently lived in poor neighborhoods | −0.04 | −0.36 | 0.28 |  |
|  |  | Economic and Social Disadvantage | metabolic and inflammatory-related risk factors | overall | Beta (95%CI) | 17 | | 1 | Entered neighborhood poverty vs. Consistently lived in poor neighborhoods | −0.04 | −0.36 | 0.28 |  |
|  |  | Economic and Social Disadvantage | metabolic and inflammatory-related risk factors | overall | Beta (95%CI) | 18 | | 1 | Entered neighborhood poverty vs. Consistently lived in poor neighborhoods | −0.18 | −0.46 | 0.11 |  |
|  |  | Economic and Social Disadvantage | cardiovascular health-related risk factors | overall | Beta (95%CI) | 19 | | 1 | Exited neighborhood poverty vs. Never lived in poor neighborhood | −0.07 | −1.15 | 1.01 |  |
|  |  | Economic and Social Disadvantage | cardiovascular health-related risk factors | overall | Beta (95%CI) | 20 | | 1 | Exited neighborhood poverty vs. Never lived in poor neighborhood | 0.76 | −0.06 | 1.57 |  |
|  |  | Economic and Social Disadvantage | cardiovascular health-related risk factors | overall | Beta (95%CI) | 21 | | 1 | Exited neighborhood poverty vs. Never lived in poor neighborhood | 0.07 | −0.11 | 0.24 |  |
|  |  | Economic and Social Disadvantage | metabolic and inflammatory-related risk factors | overall | Beta (95%CI) | 22 | | 1 | Exited neighborhood poverty vs. Never lived in poor neighborhood | −0.19 | −0.44 | 0.06 |  |
|  |  | Economic and Social Disadvantage | metabolic and inflammatory-related risk factors | overall | Beta (95%CI) | 23 | | 1 | Exited neighborhood poverty vs. Never lived in poor neighborhood | −0.02 | −0.30 | 0.27 |  |
|  |  | Economic and Social Disadvantage | metabolic and inflammatory-related risk factors | overall | Beta (95%CI) | 24 | | 1 | Exited neighborhood poverty vs. Never lived in poor neighborhood | 0.09 | −0.16 | 0.34 |  |
|  |  | Economic and Social Disadvantage | cardiovascular health-related risk factors | overall | Beta (95%CI) | 25 | | 1 | Exited neighborhood poverty vs. Consistently lived in poor neighborhoods | −1.84 | −3.24 | −0.44 |  |
|  |  | Economic and Social Disadvantage | cardiovascular health-related risk factors | overall | Beta (95%CI) | 26 | | 1 | Exited neighborhood poverty vs. Consistently lived in poor neighborhoods | −0.98 | −2.02 | 0.06 |  |
|  |  | Economic and Social Disadvantage | cardiovascular health-related risk factors | overall | Beta (95%CI) | 27 | | 1 | Exited neighborhood poverty vs. Consistently lived in poor neighborhoods | −0.04 | −0.25 | 0.18 |  |
|  |  | Economic and Social Disadvantage | metabolic and inflammatory-related risk factors | overall | Beta (95%CI) | 28 | | 1 | Exited neighborhood poverty vs. Consistently lived in poor neighborhoods | −0.10 | −0.42 | 0.21 |  |
|  |  | Economic and Social Disadvantage | metabolic and inflammatory-related risk factors | overall | Beta (95%CI) | 29 | | 1 | Exited neighborhood poverty vs. Consistently lived in poor neighborhoods | 0.05 | −0.27 | 0.37 |  |
|  |  | Economic and Social Disadvantage | metabolic and inflammatory-related risk factors | overall | Beta (95%CI) | 30 | | 1 | Exited neighborhood poverty vs. Consistently lived in poor neighborhoods | −0.01 | −0.29 | 0.29 |  |
|  |  | Economic and Social Disadvantage | cardiovascular health-related risk factors | overall | Beta (95%CI) | 31 | | 1 | Exited neighborhood poverty vs. Entered neighborhood poverty | −1.15 | −2.48 | 0.18 |  |
|  |  | Economic and Social Disadvantage | cardiovascular health-related risk factors | overall | Beta (95%CI) | 32 | | 1 | Exited neighborhood poverty vs. Entered neighborhood poverty | −0.33 | −1.33 | 0.67 |  |
|  |  | Economic and Social Disadvantage | cardiovascular health-related risk factors | overall | Beta (95%CI) | 33 | | 1 | Exited neighborhood poverty vs. Entered neighborhood poverty | −0.08 | −0.31 | 0.15 |  |
|  |  | Economic and Social Disadvantage | metabolic and inflammatory-related risk factors | overall | Beta (95%CI) | 34 | | 1 | Exited neighborhood poverty vs. Entered neighborhood poverty | −0.06 | −0.36 | 0.24 |  |
|  |  | Economic and Social Disadvantage | metabolic and inflammatory-related risk factors | overall | Beta (95%CI) | 35 | | 1 | Exited neighborhood poverty vs. Entered neighborhood poverty | 0.09 | −0.22 | 0.40 |  |
|  |  | Economic and Social Disadvantage | metabolic and inflammatory-related risk factors | overall | Beta (95%CI) | 36 | | 1 | Exited neighborhood poverty vs. Entered neighborhood poverty | 0.18 | −0.10 | 0.46 |  |
| [104] | Loose, 2017 | Discrimination and Segregation | cardiovascular health-related risk factors | overall | Beta (SE) | 1 | | 1 |  | 3.43 | - | - | 1.60 |
|  |  | Discrimination and Segregation | cardiovascular health-related risk factors | overall | Beta (SE) | 2 | | 1 |  | 2.52 | - | - | 1.00 |
| [105] | Loucks, 2006 | Social Relationships and Norms | metabolic and inflammatory-related risk factors | male | OR (95%CI) | 1 | | 1 | 4 high (reference) | 1.00 | - | - |  |
|  |  |  |  | male |  | 1 | | 2 | 3 | 1.46 | 0.71 | 2.99 |  |
|  |  |  |  | male |  | 1 | | 3 | 2 | 1.57 | 0.75 | 3.29 |  |
|  |  |  |  | male |  | 1 | | 4 | 1 low | 2.23 | 1.05 | 4.76 |  |
|  |  | Social Relationships and Norms | metabolic and inflammatory-related risk factors | female | OR (95%CI) | 2 | | 1 | 4 high (reference) | 1.00 | - | - |  |
|  |  |  |  | female |  | 2 | | 2 | 3 | 1.21 | 0.62 | 2.37 |  |
|  |  |  |  | female |  | 2 | | 3 | 2 | 1.22 | 0.62 | 2.38 |  |
|  |  |  |  | female |  | 2 | | 4 | 1 low | 0.93 | 0.43 | 1.99 |  |
|  |  | Social Relationships and Norms | metabolic and inflammatory-related risk factors | male | OR (95%CI) | 3 | | 1 | 4 high (reference) | 1.00 | - | - |  |
|  |  |  |  | male |  | 3 | | 2 | 3 | 1.63 | 0.82 | 3.24 |  |
|  |  |  |  | male |  | 3 | | 3 | 2 | 1.06 | 0.52 | 2.17 |  |
|  |  |  |  | male |  | 3 | | 4 | 1 low | 1.30 | 0.61 | 2.79 |  |
|  |  | Social Relationships and Norms | metabolic and inflammatory-related risk factors | female | OR (95%CI) | 4 | | 1 | 4 high (reference) | 1.00 | - | - |  |
|  |  |  |  | female |  | 4 | | 2 | 3 | 1.37 | 0.71 | 2.63 |  |
|  |  |  |  | female |  | 4 | | 3 | 2 | 0.82 | 0.42 | 1.61 |  |
|  |  |  |  | female |  | 4 | | 4 | 1 low | 0.93 | 0.44 | 1.97 |  |
| [106] | Loucks, 2005 | Social Relationships and Norms | metabolic and inflammatory-related risk factors | male | OR (95%CI) | 1 | | 1 | 4 high; reference | 1.00 | - | - |  |
|  |  |  |  | male |  | 1 | | 2 | 3 | 1.73 | 0.86 | 3.47 |  |
|  |  |  |  | male |  | 1 | | 3 | 2 | 2.31 | 1.16 | 4.63 |  |
|  |  |  |  | male |  | 1 | | 4 | 1 low | 2.61 | 1.26 | 5.42 |  |
|  |  | Social Relationships and Norms | metabolic and inflammatory-related risk factors | female | OR (95%CI) | 2 | | 1 | 4 high; reference | 1.00 | - | - |  |
|  |  |  |  | female |  | 2 | | 2 | 3 | 1.10 | 0.60 | 2.07 |  |
|  |  |  |  | female |  | 2 | | 3 | 2 | 1.14 | 0.63 | 2.07 |  |
|  |  |  |  | female |  | 2 | | 4 | 1 low | 0.67 | 0.33 | 1.36 |  |
| [107] | Loucks, 2006 | Social Relationships and Norms | cardiovascular health-related risk factors | female | Mean | 1 | | 1 | 1 low | 124.00 | - | - |  |
|  |  |  |  | female |  | 1 | | 2 | 2 | 127.00 | - | - |  |
|  |  |  |  | female |  | 1 | | 3 | 3 | 126.00 | - | - |  |
|  |  |  |  | female |  | 1 | | 4 | 4 high | 126.00 | - | - |  |
|  |  | Social Relationships and Norms | cardiovascular health-related risk factors | female | Mean | 2 | | 1 | 1 low | 71.00 | - | - |  |
|  |  |  |  | female |  | 2 | | 2 | 2 | 73.00 | - | - |  |
|  |  |  |  | female |  | 2 | | 3 | 3 | 72.00 | - | - |  |
|  |  |  |  | female |  | 2 | | 4 | 4 high | 73.00 | - | - |  |
|  |  | Social Relationships and Norms | cardiovascular health-related risk factors | male | Mean | 3 | | 1 | 1 low | 128.00 | - | - |  |
|  |  |  |  | male |  | 3 | | 2 | 2 | 128.00 | - | - |  |
|  |  |  |  | male |  | 3 | | 3 | 3 | 127.00 | - | - |  |
|  |  |  |  | male |  | 3 | | 4 | 4 high | 128.00 | - | - |  |
|  |  | Social Relationships and Norms | cardiovascular health-related risk factors | male | Mean | 4 | | 1 | 1 low | 76.00 | - | - |  |
|  |  |  |  | male |  | 4 | | 2 | 2 | 76.00 | - | - |  |
|  |  |  |  | male |  | 4 | | 3 | 3 | 76.00 | - | - |  |
|  |  |  |  | male |  | 4 | | 4 | 4 high | 75.00 | - | - |  |
|  |  | Social Relationships and Norms | metabolic and inflammatory-related risk factors | male | Mean (SE) | 5 | | 1 | 1 low | 3.85 | - | - | 0.38 |
|  |  |  |  | male |  | 5 | | 2 | 2 | 3.97 | - | - | 0.27 |
|  |  |  |  | male |  | 5 | | 3 | 3 | 3.59 | - | - | 0.28 |
|  |  |  |  | male |  | 5 | | 4 | 4 high | 3.52 | - | - | 0.35 |
|  |  | Social Relationships and Norms | metabolic and inflammatory-related risk factors | female | Mean (SE) | 6 | | 1 | 1 low | 3.64 | - | - | 0.28 |
|  |  |  |  | female |  | 6 | | 2 | 2 | 3.33 | - | - | 0.21 |
|  |  |  |  | female |  | 6 | | 3 | 3 | 3.43 | - | - | 0.19 |
|  |  |  |  | female |  | 6 | | 4 | 4 high | 3.38 | - | - | 0.23 |
|  |  | Social Relationships and Norms | metabolic and inflammatory-related risk factors | male | Mean (SE) | 7 | | 1 | 1 low | 3.18 | - | - | 0.62 |
|  |  |  |  | male |  | 7 | | 2 | 2 | 3.41 | - | - | 0.43 |
|  |  |  |  | male |  | 7 | | 3 | 3 | 3.09 | - | - | 0.46 |
|  |  |  |  | male |  | 7 | | 4 | 4 high | 3.34 | - | - | 0.57 |
|  |  | Social Relationships and Norms | metabolic and inflammatory-related risk factors | female | Mean (SE) | 8 | | 1 | 1 low | 3.90 | - | - | 0.38 |
|  |  |  |  | female |  | 8 | | 2 | 2 | 3.86 | - | - | 0.28 |
|  |  |  |  | female |  | 8 | | 3 | 3 | 4.15 | - | - | 0.26 |
|  |  |  |  | female |  | 8 | | 4 | 4 high | 4.21 | - | - | 0.31 |
|  |  | Social Relationships and Norms | metabolic and inflammatory-related risk factors | male | Mean (SE) | 9 | | 1 | 1 low | 254.00 | - | - | 5.30 |
|  |  |  |  | male |  | 9 | | 2 | 2 | 256.00 | - | - | 3.70 |
|  |  |  |  | male |  | 9 | | 3 | 3 | 258.00 | - | - | 3.90 |
|  |  |  |  | male |  | 9 | | 4 | 4 high | 253.00 | - | - | 5.00 |
|  |  | Social Relationships and Norms | metabolic and inflammatory-related risk factors | female | Mean (SE) | 10 | | 1 | 1 low | 256.00 | - | - | 4.60 |
|  |  |  |  | female |  | 10 | | 2 | 2 | 250.00 | - | - | 3.40 |
|  |  |  |  | female |  | 10 | | 3 | 3 | 256.00 | - | - | 3.10 |
|  |  |  |  | female |  | 10 | | 4 | 4 high | 255.00 | - | - | 3.70 |
|  |  | Social Relationships and Norms | metabolic and inflammatory-related risk factors | male | Mean (SE) | 11 | | 1 | 1 low | 335.00 | - | - | 7.70 |
|  |  |  |  | male |  | 11 | | 2 | 2 | 321.00 | - | - | 5.40 |
|  |  |  |  | male |  | 11 | | 3 | 3 | 338.00 | - | - | 5.70 |
|  |  |  |  | male |  | 11 | | 4 | 4 high | 325.00 | - | - | 7.10 |
|  |  | Social Relationships and Norms | metabolic and inflammatory-related risk factors | female | Mean (SE) | 12 | | 1 | 1 low | 329.00 | - | - | 9.00 |
|  |  |  |  | female |  | 12 | | 2 | 2 | 320.00 | - | - | 6.50 |
|  |  |  |  | female |  | 12 | | 3 | 3 | 329.00 | - | - | 6.00 |
|  |  |  |  | female |  | 12 | | 4 | 4 high | 328.00 | - | - | 7.20 |
|  |  | Social Relationships and Norms | metabolic and inflammatory-related risk factors | female | Mean | 13 | | 1 | 1 low | 3.60 | - | - |  |
|  |  |  |  | female |  | 13 | | 2 | 2 | 3.70 | - | - |  |
|  |  |  |  | female |  | 13 | | 3 | 3 | 3.60 | - | - |  |
|  |  |  |  | female |  | 13 | | 4 | 4 high | 3.70 | - | - |  |
|  |  | Social Relationships and Norms | metabolic and inflammatory-related risk factors | male | Mean | 14 | | 1 | 1 low | 4.70 | - | - |  |
|  |  |  |  | male |  | 14 | | 2 | 2 | 4.50 | - | - |  |
|  |  |  |  | male |  | 14 | | 3 | 3 | 4.50 | - | - |  |
|  |  |  |  | male |  | 14 | | 4 | 4 high | 4.60 | - | - |  |
| [108] | Maki, 2020 | Social Relationships and Norms | glucose metabolism-related risk factors | overall | Beta (SE) | 1 | | 1 |  | −0.05 | - | - | 0.03 |
|  |  | Social Relationships and Norms | glucose metabolism-related risk factors | overall | Beta | 2 | | 1 |  | 0.03 | - | - |  |
|  |  | Social Relationships and Norms | glucose metabolism-related risk factors | overall | Beta | 3 | | 1 |  | 0.02 | - | - |  |
|  |  | Social Relationships and Norms | glucose metabolism-related risk factors | overall | Beta | 4 | | 1 |  | −0.02 | - | - |  |
|  |  | Social Relationships and Norms | glucose metabolism-related risk factors | overall | Beta | 5 | | 1 |  | −0.03 | - | - |  |
|  |  | Social Relationships and Norms | glucose metabolism-related risk factors | overall | Beta | 6 | | 1 |  | −0.03 | - | - |  |
| [109] | Marley, 2015 | Social Cohesion and Social Capital | glucose metabolism-related risk factors | overall | OR (95%CI) | 1 | | 1 |  | 1.07 | - | - |  |
|  |  | Economic and Social Disadvantage | glucose metabolism-related risk factors | overall | OR (95%CI) | 2 | | 1 |  | 1.06 | - | - |  |
| [110] | Martin, 2019 | Economic and Social Disadvantage | CVD risk scores | overall | Standardized estimate from structural equation modeling | 1 | | 1 |  | 0.06 | - | - |  |
|  |  | Economic and Social Disadvantage | CVD risk scores | overall | Standardized estimate from structural equation modeling | 2 | | 1 |  | 0.04 | - | - |  |
|  |  | Economic and Social Disadvantage | CVD risk scores | overall | Standardized estimate from structural equation modeling | 3 | | 1 |  | −0.004 | - | - |  |
| [111] | Matricciani, 2013 | Economic and Social Disadvantage | cardiovascular health-related risk factors | overall | Beta (95%CI) | 1 | | 1 |  | −0.10 | −0.17 | −0.04 |  |
|  |  | Economic and Social Disadvantage | cardiovascular health-related risk factors | overall | Beta (95%CI) | 2 | | 1 |  | −0.002 | −0.004 | 0.00 |  |
|  |  | Economic and Social Disadvantage | cardiovascular health-related risk factors | overall | Beta (95%CI) | 3 | | 1 |  | 0.14 | −0.16 | 0.43 |  |
| [112] | Mayne, 2019 | Discrimination and Segregation | CVD risk scores | overall | Beta | 1 | | 1 | Low segregation (reference) | - | - | - |  |
|  |  |  |  | overall |  | 1 | | 2 | Medium segregation | −0.06 | −0.15 | 0.03 |  |
|  |  |  |  | overall |  | 1 | | 3 | High segregation | −0.05 | −0.19 | 0.08 |  |
|  |  | Discrimination and Segregation | CVD risk scores | overall | Beta | 3 | | 1 | Low segregation (reference) | - | - | - |  |
|  |  |  |  | overall |  | 3 | | 2 | Medium segregation | −0.01 | −0.01 | 0.00 |  |
|  |  |  |  | overall |  | 3 | | 3 | High segregation | 0.00 | −0.02 | 0.01 |  |
|  |  | Discrimination and Segregation | CVD risk scores | overall | Beta | 4 | | 1 | Low segregation (reference) | - | - | - |  |
|  |  |  |  | overall |  | 4 | | 2 | Medium segregation | 0.18 | 0.03 | 0.33 |  |
|  |  |  |  | overall |  | 4 | | 3 | High segregation | 0.17 | 0.02 | 0.32 |  |
|  |  | Discrimination and Segregation | CVD risk scores | overall | Beta | 6 | | 1 | Low segregation (reference) | - | - | - |  |
|  |  |  |  | overall |  | 6 | | 2 | Medium segregation | 0.00 | −0.02 | 0.01 |  |
|  |  |  |  | overall |  | 6 | | 3 | High segregation | 0.00 | −0.01 | 0.01 |  |
|  |  | Discrimination and Segregation | CVD risk scores | overall | Beta | 7 | | 1 | Low segregation (reference) | - | - | - |  |
|  |  |  |  | overall |  | 7 | | 2 | Medium segregation | 0.04 | −0.13 | 0.20 |  |
|  |  |  |  | overall |  | 7 | | 3 | High segregation | 0.12 | −0.03 | 0.27 |  |
|  |  | Discrimination and Segregation | CVD risk scores | overall | Beta | 9 | | 1 | Low segregation (reference) | - | - | - |  |
|  |  |  |  | overall |  | 9 | | 2 | Medium segregation | 0.00 | −0.02 | 0.01 |  |
|  |  |  |  | overall |  | 9 | | 3 | High segregation | −0.01 | −0.02 | 0.01 |  |
| [113] | Mayne, 2018 | Crime and Safety | cardiovascular health-related risk factors | male | Beta (95%CI) | 1 | | 1 |  | −1.38 | −3.43 | 0.67 |  |
|  |  | Crime and Safety | cardiovascular health-related risk factors | female | Beta (95%CI) | 2 | | 1 |  | −0.30 | −2.37 | 1.77 |  |
|  |  | Crime and Safety | cardiovascular health-related risk factors | male | Beta (95%CI) | 3 | | 1 |  | 0.45 | −0.70 | 1.60 |  |
|  |  | Crime and Safety | cardiovascular health-related risk factors | female | Beta (95%CI) | 4 | | 1 |  | −0.12 | −1.08 | 0.84 |  |
|  |  | Crime and Safety | cardiovascular health-related risk factors | male | Beta (95%CI) | 5 | | 1 |  | 0.32 | −0.13 | 0.78 |  |
|  |  | Crime and Safety | cardiovascular health-related risk factors | female | Beta (95%CI) | 6 | | 1 |  | −0.83 | −1.36 | −0.30 |  |
|  |  | Crime and Safety | cardiovascular health-related risk factors | male | Beta (95%CI) | 7 | | 1 |  | 0.11 | −0.15 | 0.36 |  |
|  |  | Crime and Safety | cardiovascular health-related risk factors | female | Beta (95%CI) | 8 | | 1 |  | −0.25 | −0.50 | −0.01 |  |
| [114] | McKenzie, 2020 | Economic and Social Disadvantage | CVD risk scores | male | OR (95%CI) | 1 | | 1 | Upper tertile (>2–9 million JMD) | 1.00 | - | - |  |
|  |  |  |  | male |  | 1 | | 2 | Middle tertile (1–2 million JMD) | 0.46 | 0.20 | 1.04 |  |
|  |  |  |  | male |  | 1 | | 3 | Lower tertile (<1 million JMD) | 0.33 | 0.12 | 0.91 |  |
|  |  | Economic and Social Disadvantage | CVD risk scores | female | OR (95%CI) | 2 | | 1 | Upper tertile (>2–9 million JMD) | 1.00 | - | - |  |
|  |  |  |  | female |  | 2 | | 2 | Middle tertile (1–2 million JMD) | 0.70 | 0.37 | 1.32 |  |
|  |  |  |  | female |  | 2 | | 3 | Lower tertile (<1 million JMD) | 0.72 | 0.38 | 1.34 |  |
| [115] | Mellman, 2015 | Disorder and Incivilities | cardiovascular health-related risk factors | overall | Beta | 1 | | 1 |  | -0.16 | - | - |  |
|  |  | Economic and Social Disadvantage | cardiovascular health-related risk factors | overall | Beta | 2 | | 1 |  | -0.16 | - | - |  |
| [116] | Merkin, 2009 | Economic and Social Disadvantage | CVD risk scores | overall | Beta (95%CI) | 1 | | 1 | Q1 (low) | 0.11 | −0.05 | 0.28 |  |
|  |  |  |  | overall |  | 1 | | 2 | Q2 | −0.004 | −0.15 | 0.14 |  |
|  |  |  |  | overall |  | 1 | | 3 | Q3 | 0.08 | −0.04 | 0.20 |  |
|  |  |  |  | overall |  | 1 | | 4 | Q4 | 0.01 | −0.09 | 0.10 |  |
|  |  |  |  | overall |  | 1 | | 5 | Q5 (high) - reference | - | - | - |  |
|  |  | Economic and Social Disadvantage | CVD risk scores | overall | Beta (95%CI) | 2 | | 1 | Q1 (low) | 0.40 | 0.19 | 0.61 |  |
|  |  |  |  | overall |  | 2 | | 2 | Q2 | 0.26 | 0.04 | 0.47 |  |
|  |  |  |  | overall |  | 2 | | 3 | Q3 | 0.17 | −0.10 | 0.43 |  |
|  |  |  |  | overall |  | 2 | | 4 | Q4 | 0.36 | 0.06 | 0.65 |  |
|  |  |  |  | overall |  | 2 | | 5 | Q5 (high) - reference | - | - | - |  |
|  |  | Economic and Social Disadvantage | CVD risk scores | overall | Beta (95%CI) | 3 | | 1 | Q1 (low) | 0.30 | 0.05 | 0.55 |  |
|  |  |  |  | overall |  | 3 | | 2 | Q2 | 0.24 | −0.02 | 0.49 |  |
|  |  |  |  | overall |  | 3 | | 3 | Q3 | 0.29 | 0.04 | 0.54 |  |
|  |  |  |  | overall |  | 3 | | 4 | Q4 | 0.16 | −0.13 | 0.45 |  |
|  |  |  |  | overall |  | 3 | | 5 | Q5 (high) - reference | - | - | - |  |
|  |  | Economic and Social Disadvantage | CVD risk scores | overall | OR (95%CI) | 4 | | 1 | Q1 (low) | 1.30 | 0.90 | 1.80 |  |
|  |  |  |  | overall |  | 4 | | 2 | Q2 | 0.80 | 0.60 | 1.10 |  |
|  |  |  |  | overall |  | 4 | | 3 | Q3 | 1.10 | 0.80 | 1.50 |  |
|  |  |  |  | overall |  | 4 | | 4 | Q4 | 1.00 | 0.70 | 1.30 |  |
|  |  |  |  | overall |  | 4 | | 5 | Q5 (high) - reference | - | - | - |  |
|  |  | Economic and Social Disadvantage | CVD risk scores | overall | OR (95%CI) | 5 | | 1 | Q1 (low) | 2.20 | 1.10 | 4.10 |  |
|  |  |  |  | overall |  | 5 | | 2 | Q2 | 2.10 | 1.10 | 4.10 |  |
|  |  |  |  | overall |  | 5 | | 3 | Q3 | 1.70 | 0.80 | 3.40 |  |
|  |  |  |  | overall |  | 5 | | 4 | Q4 | 1.90 | 0.90 | 3.70 |  |
|  |  |  |  | overall |  | 5 | | 5 | Q5 (high) - reference | - | - | - |  |
|  |  | Economic and Social Disadvantage | CVD risk scores | overall | OR (95%CI) | 6 | | 1 | Q1 (low) | 1.70 | 0.80 | 3.30 |  |
|  |  |  |  | overall |  | 6 | | 2 | Q2 | 1.60 | 0.80 | 3.20 |  |
|  |  |  |  | overall |  | 6 | | 3 | Q3 | 1.80 | 0.90 | 3.60 |  |
|  |  |  |  | overall |  | 6 | | 4 | Q4 | 1.20 | 0.60 | 2.70 |  |
|  |  |  |  | overall |  | 6 | | 5 | Q5 (high) - reference | - | - | - |  |
| [117] | Merkin, 2020 | Economic and Social Disadvantage | CVD risk scores | overall | Mean difference (95%CI) | 1 | | 1 |  | -0.30 | -0.46 | -0.13 |  |
|  |  | Economic and Social Disadvantage | CVD risk scores | overall | Mean difference (95%CI) | 2 | | 1 |  | -0.29 | -0.57 | 0.00 |  |
|  |  | Economic and Social Disadvantage | CVD risk scores | overall | Mean difference (95%CI) | 3 | | 1 |  | -0.25 | -0.44 | -0.06 |  |
|  |  | Economic and Social Disadvantage | CVD risk scores | overall | Mean difference (95%CI) | 4 | | 1 |  | -0.26 | -0.44 | -0.09 |  |
|  |  | Economic and Social Disadvantage | CVD risk scores | overall | Mean difference (95%CI) | 5 | | 1 |  | 0.00 | -0.01 | 0.02 |  |
|  |  | Economic and Social Disadvantage | CVD risk scores | overall | Mean difference (95%CI) | 6 | | 1 |  | -0.01 | -0.04 | 0.01 |  |
|  |  | Economic and Social Disadvantage | CVD risk scores | overall | Mean difference (95%CI) | 7 | | 1 |  | 0.00 | -0.02 | 0.02 |  |
|  |  | Economic and Social Disadvantage | CVD risk scores | overall | Mean difference (95%CI) | 8 | | 1 |  | 0.01 | -0.01 | 0.02 |  |
|  |  | Economic and Social Disadvantage | CVD risk scores | overall | Mean difference | 9 | | 1 | Q1 (lowest) - reference | - | - | - |  |
|  |  |  |  | overall |  | 9 | | 2 | Q2 | -0.23 | -0.82 | 0.37 |  |
|  |  |  |  | overall |  | 9 | | 3 | Q3 | -0.18 | -0.57 | 0.21 |  |
|  |  |  |  | overall |  | 9 | | 4 | Q4 | -0.32 | -0.77 | 0.13 |  |
|  |  |  |  | overall |  | 9 | | 5 | Q5 (highest) | -0.86 | -1.41 | -0.31 |  |
|  |  | Economic and Social Disadvantage | CVD risk scores | overall | Mean difference | 10 | | 1 | Q1 (lowest) - reference | - | - | - |  |
|  |  |  |  | overall |  | 10 | | 2 | Q2 | 0.22 | -0.70 | 1.14 |  |
|  |  |  |  | overall |  | 10 | | 3 | Q3 | -0.18 | -1.08 | 0.72 |  |
|  |  |  |  | overall |  | 10 | | 4 | Q4 | -0.30 | -1.20 | 0.61 |  |
|  |  |  |  | overall |  | 10 | | 5 | Q5 (highest) | -0.60 | -1.66 | 0.45 |  |
|  |  | Economic and Social Disadvantage | CVD risk scores | overall | Mean difference | 11 | | 1 | Q1 (lowest) - reference | - | - | - |  |
|  |  |  |  | overall |  | 11 | | 2 | Q2 | 0.12 | -0.45 | 0.68 |  |
|  |  |  |  | overall |  | 11 | | 3 | Q3 | -0.01 | -0.55 | 0.53 |  |
|  |  |  |  | overall |  | 11 | | 4 | Q4 | -0.22 | -0.81 | 0.37 |  |
|  |  |  |  | overall |  | 11 | | 5 | Q5 (highest) | -0.71 | -1.48 | 0.06 |  |
|  |  | Economic and Social Disadvantage | CVD risk scores | overall | Mean difference | 12 | | 1 | Q1 (lowest) - reference | - | - | - |  |
|  |  |  |  | overall |  | 12 | | 2 | Q2 | -0.24 | -0.72 | 0.24 |  |
|  |  |  |  | overall |  | 12 | | 3 | Q3 | -0.19 | -0.70 | 0.31 |  |
|  |  |  |  | overall |  | 12 | | 4 | Q4 | -0.27 | 0.31 | 0.28 |  |
|  |  |  |  | overall |  | 12 | | 5 | Q5 (highest) | -1.15 | -1.87 | -0.44 |  |
|  |  | Economic and Social Disadvantage | CVD risk scores | overall | Mean difference | 13 | | 1 | Q1 (lowest) - reference | - | - | - |  |
|  |  |  |  | overall |  | 13 | | 2 | Q2 | 0.02 | -0.04 | 0.09 |  |
|  |  |  |  | overall |  | 13 | | 3 | Q3 | 0.01 | -0.03 | 0.06 |  |
|  |  |  |  | overall |  | 13 | | 4 | Q4 | 0.02 | -0.03 | 0.07 |  |
|  |  |  |  | overall |  | 13 | | 5 | Q5 (highest) | 0.03 | -0.03 | 0.08 |  |
|  |  | Economic and Social Disadvantage | CVD risk scores | overall | Mean difference | 14 | | 1 | Q1 (lowest) - reference | - | - | - |  |
|  |  |  |  | overall |  | 14 | | 2 | Q2 | 0.04 | -0.04 | 0.13 |  |
|  |  |  |  | overall |  | 14 | | 3 | Q3 | -0.04 | -0.12 | 0.03 |  |
|  |  |  |  | overall |  | 14 | | 4 | Q4 | -0.03 | -0.11 | 0.05 |  |
|  |  |  |  | overall |  | 14 | | 5 | Q5 (highest) | 0.00 | -0.09 | 0.09 |  |
|  |  | Economic and Social Disadvantage | CVD risk scores | overall | Mean difference | 15 | | 1 | Q1 (lowest) - reference | - | - | - |  |
|  |  |  |  | overall |  | 15 | | 2 | Q2 | -0.05 | -0.11 | 0.00 |  |
|  |  |  |  | overall |  | 15 | | 3 | Q3 | -0.01 | -0.06 | 0.04 |  |
|  |  |  |  | overall |  | 15 | | 4 | Q4 | -0.05 | -0.10 | 0.00 |  |
|  |  |  |  | overall |  | 15 | | 5 | Q5 (highest) | -0.01 | -0.08 | 0.06 |  |
|  |  | Economic and Social Disadvantage | CVD risk scores | overall | Mean difference | 16 | | 1 | Q1 (lowest) - reference | - | - | - |  |
|  |  |  |  | overall |  | 16 | | 2 | Q2 | -0.05 | -0.09 | 0.00 |  |
|  |  |  |  | overall |  | 16 | | 3 | Q3 | 0.00 | -0.05 | 0.05 |  |
|  |  |  |  | overall |  | 16 | | 4 | Q4 | 0.02 | -0.04 | 0.07 |  |
|  |  |  |  | overall |  | 16 | | 5 | Q5 (highest) | -0.01 | -0.09 | 0.07 |  |
| [118] | Merlo, 2001 | Economic and Social Disadvantage | cardiovascular health-related risk factors | female | Beta (SE) | 1 | | 1 |  | 2.97 | - | - | 1.25 |
| [119] | Metcalf, 2008 | Economic and Social Disadvantage | CVD risk scores | overall | Mean (SE) | 9 | | 1 | 1&2 | 7.00 | - | - | 0.20 |
|  |  |  |  | overall |  | 9 | | 2 | 3&4 | 7.60 | - | - | 0.21 |
|  |  |  |  | overall |  | 9 | | 3 | 5&6 | 7.70 | - | - | 0.19 |
|  |  |  |  | overall |  | 9 | | 4 | 7&8 | 8.30 | - | - | 0.21 |
|  |  |  |  | overall |  | 9 | | 5 | 9&10 | 8.20 | - | - | 0.23 |
|  |  | Economic and Social Disadvantage | metabolic and inflammatory-related risk factors | overall | Mean (SE) | 10 | | 1 | 1&2 | 3.60 | - | - | 1.09 |
|  |  |  |  | overall |  | 10 | | 2 | 3&4 | 4.50 | - | - | 1.09 |
|  |  |  |  | overall |  | 10 | | 3 | 5&6 | 4.70 | - | - | 1.08 |
|  |  |  |  | overall |  | 10 | | 4 | 7&8 | 5.30 | - | - | 1.12 |
|  |  |  |  | overall |  | 10 | | 5 | 9&10 | 4.90 | - | - | 1.12 |
| [120] | Meza, 2020 | Social Relationships and Norms | cardiovascular health-related risk factors | overall | Beta (95%CI) | 1 | | 1 |  | −2.20 | −5.88 | 1.60 |  |
|  |  | Social Relationships and Norms | cardiovascular health-related risk factors | overall | Beta (95%CI) | 2 | | 1 |  | 0.70 | −2.93 | 4.37 |  |
|  |  | Social Relationships and Norms | cardiovascular health-related risk factors | overall | Beta (95%CI) | 3 | | 1 |  | −1.20 | −4.80 | 2.49 |  |
|  |  | Social Relationships and Norms | cardiovascular health-related risk factors | overall | Beta (95%CI) | 4 | | 1 |  | 2.20 | −1.62 | 6.21 |  |
|  |  | Social Relationships and Norms | cardiovascular health-related risk factors | overall | Beta (95%CI) | 5 | | 1 |  | 1.20 | −2.50 | 5.15 |  |
|  |  | Social Relationships and Norms | cardiovascular health-related risk factors | overall | Beta (95%CI) | 6 | | 1 |  | −3.60 | −7.64 | 0.70 |  |
|  |  | Social Relationships and Norms | cardiovascular health-related risk factors | overall | Beta (95%CI) | 7 | | 1 |  | −0.40 | −4.41 | 3.79 |  |
|  |  | Social Relationships and Norms | cardiovascular health-related risk factors | overall | Beta (95%CI) | 8 | | 1 |  | −0.70 | −4.74 | 3.58 |  |
|  |  | Social Relationships and Norms | cardiovascular health-related risk factors | overall | Beta (95%CI) | 9 | | 1 |  | 1.40 | −2.94 | 5.89 |  |
|  |  | Social Relationships and Norms | cardiovascular health-related risk factors | overall | Beta (95%CI) | 10 | | 1 |  | 2.40 | −1.87 | 6.89 |  |
| [121] | Mobley, 2006 | Discrimination and Segregation | CVD risk scores | female | Beta (SE) | 1 | | 1 |  | 0.08 | - | - | 0.14 |
|  |  | Discrimination and Segregation | CVD risk scores | female | Beta (SE) | 2 | | 1 |  | -0.52 | - | - | 0.26 |
|  |  | Discrimination and Segregation | CVD risk scores | female | Beta (SE) | 3 | | 1 |  | -0.61 | - | - | 0.22 |
|  |  | Discrimination and Segregation | CVD risk scores | female | Beta (SE) | 4 | | 1 |  | -2.44 | - | - | 0.96 |
|  |  | Discrimination and Segregation | CVD risk scores | female | Beta (SE) | 5 | | 1 |  | 1.91 | - | - | 1.02 |
|  |  | Discrimination and Segregation | CVD risk scores | female | Beta (SE) | 6 | | 1 |  | 4.17 | - | - | 6.54 |
|  |  | Crime and Safety | CVD risk scores | female | Beta (SE) | 7 | | 1 |  | 0.00 | - | - | 0.00 |
|  |  | Economic and Social Disadvantage | CVD risk scores | female | Beta (SE) | 8 | | 1 |  | 0.01 | - | - | 0.07 |
|  |  | Economic and Social Disadvantage | CVD risk scores | female | Beta (SE) | 9 | | 1 |  | -0.07 | - | - | 0.03 |
| [122] | Murakami, 2010 | Economic and Social Disadvantage | cardiovascular health-related risk factors | female | Beta (95%CI) | 1 | | 1 | Q1 | 105.20 | 104.00 | 106.50 |  |
|  |  |  |  | female |  | 1 | | 2 | Q2 | 105.20 | 103.90 | 106.50 |  |
|  |  |  |  | female |  | 1 | | 3 | Q3 | 106.00 | 104.60 | 107.30 |  |
|  |  |  |  | female |  | 1 | | 4 | Q4 | 108.50 | 107.30 | 109.70 |  |
|  |  | Economic and Social Disadvantage | metabolic and inflammatory-related risk factors | female | Beta (95%CI) | 2 | | 1 | Q1 | 71.00 | 69.50 | 72.60 |  |
|  |  |  |  | female |  | 2 | | 2 | Q2 | 71.60 | 70.00 | 73.20 |  |
|  |  |  |  | female |  | 2 | | 3 | Q3 | 70.30 | 68.60 | 72.00 |  |
|  |  |  |  | female |  | 2 | | 4 | Q4 | 70.10 | 68.50 | 71.60 |  |
|  |  | Economic and Social Disadvantage | metabolic and inflammatory-related risk factors | female | Beta (95%CI) | 3 | | 1 | Q1 | 54.40 | 52.40 | 56.40 |  |
|  |  |  |  | female |  | 3 | | 2 | Q2 | 54.90 | 52.90 | 56.90 |  |
|  |  |  |  | female |  | 3 | | 3 | Q3 | 59.60 | 57.60 | 61.70 |  |
|  |  |  |  | female |  | 3 | | 4 | Q4 | 56.00 | 54.00 | 58.00 |  |
|  |  | Economic and Social Disadvantage | glucose metabolism-related risk factors | female | Beta (95%CI) | 4 | | 1 | Q1 | 84.20 | 83.40 | 84.90 |  |
|  |  |  |  | female |  | 4 | | 2 | Q2 | 84.00 | 83.20 | 84.80 |  |
|  |  |  |  | female |  | 4 | | 3 | Q3 | 83.90 | 83.10 | 84.80 |  |
|  |  |  |  | female |  | 4 | | 4 | Q4 | 84.00 | 83.20 | 84.80 |  |
|  |  | Economic and Social Disadvantage | glucose metabolism-related risk factors | female | Beta (95%CI) | 5 | | 1 | Q1 | 7.10 | 7.10 | 7.20 |  |
|  |  |  |  | female |  | 5 | | 2 | Q2 | 7.40 | 7.30 | 7.40 |  |
|  |  |  |  | female |  | 5 | | 3 | Q3 | 7.30 | 7.30 | 7.40 |  |
|  |  |  |  | female |  | 5 | | 4 | Q4 | 7.90 | 7.80 | 7.90 |  |
|  |  | Economic and Social Disadvantage | glucose metabolism-related risk factors | female | Beta (95%CI) | 6 | | 1 | Q1 | -0.11 | -0.23 | 0.01 |  |
|  |  |  |  | female |  | 6 | | 2 | Q2 | -0.08 | -0.20 | 0.04 |  |
|  |  |  |  | female |  | 6 | | 3 | Q3 | 0.08 | -0.05 | 0.21 |  |
|  |  |  |  | female |  | 6 | | 4 | Q4 | 0.11 | 0.00 | 0.23 |  |
| [123] | Murray, 2010 | Economic and Social Disadvantage | cardiovascular health-related risk factors | female | Mean difference (95%CI) | 1 | | 1 |  | 28.70 | 4.50 | 52.90 |  |
|  |  | Economic and Social Disadvantage | cardiovascular health-related risk factors | female | Mean difference (95%CI) | 2 | | 1 |  | -14.20 | -37.30 | 8.90 |  |
|  |  | Economic and Social Disadvantage | cardiovascular health-related risk factors | female | Mean difference (95%CI) | 3 | | 1 |  | 25.00 | 1.00 | 48.90 |  |
|  |  | Economic and Social Disadvantage | cardiovascular health-related risk factors | female | Mean difference (95%CI) | 4 | | 1 |  | -2.60 | -29.10 | 23.90 |  |
| [124] | Naimi, 2009 | Economic and Social Disadvantage | CVD risk scores | overall | OR (95%CI) | 1 | | 1 | ALU4 | 1.85 | 1.32 | 2.59 |  |
|  |  |  |  | overall |  | 1 | | 2 | ALU3 | 1.60 | 1.25 | 2.04 |  |
|  |  |  |  | overall |  | 1 | | 3 | ALU2 | 1.28 | 0.92 | 1.77 |  |
|  |  |  |  | overall |  | 1 | | 4 | ALU1 - reference | 1.00 | - | - |  |
|  |  | Economic and Social Disadvantage | CVD risk scores | overall | OR (95%CI) | 2 | | 1 | ALU4 | 1.64 | 1.13 | 2.39 |  |
|  |  |  |  | overall |  | 2 | | 2 | ALU3 | 1.42 | 1.03 | 1.96 |  |
|  |  |  |  | overall |  | 2 | | 3 | ALU2 | 1.19 | 0.71 | 2.01 |  |
|  |  |  |  | overall |  | 2 | | 4 | ALU1 - reference | 1.00 | - | - |  |
|  |  | Economic and Social Disadvantage | CVD risk scores | overall | OR (95%CI) | 3 | | 1 | ALU4 | 2.38 | 0.98 | 5.79 |  |
|  |  |  |  | overall |  | 3 | | 2 | ALU3 | 2.64 | 0.67 | 4.02 |  |
|  |  |  |  | overall |  | 3 | | 3 | ALU2 | 1.27 | 0.61 | 2.64 |  |
|  |  |  |  | overall |  | 3 | | 4 | ALU1 - reference | 0.99 | 0.46 | 2.09 |  |
|  |  | Economic and Social Disadvantage | CVD risk scores | male | PR (95%CI) | 5 | | 1 | ALU4 | 1.64 | 1.13 | 2.39 |  |
|  |  |  |  | male |  | 5 | | 2 | ALU3 | 1.42 | 1.03 | 1.96 |  |
|  |  |  |  | male |  | 5 | | 3 | ALU2 | 1.19 | 0.71 | 2.01 |  |
|  |  |  |  | male |  | 5 | | 4 | ALU1 - reference | - | - | - |  |
|  |  | Economic and Social Disadvantage | CVD risk scores | female | PR (95%CI) | 6 | | 1 | ALU4 | 2.38 | 0.98 | 5.79 |  |
|  |  |  |  | female |  | 6 | | 2 | ALU3 | 2.64 | 0.67 | 4.02 |  |
|  |  |  |  | female |  | 6 | | 3 | ALU2 | 1.27 | 0.61 | 2.64 |  |
|  |  |  |  | female |  | 6 | | 4 | ALU1 - reference | - | - | - |  |
| [125] | Nazmi, 2010 | Economic and Social Disadvantage | metabolic and inflammatory-related risk factors | overall | Beta (95%CI) | 1 | | 1 |  | 0.90 | 0.30 | 1.50 |  |
|  |  | Crime and Safety | metabolic and inflammatory-related risk factors | overall | Beta (95%CI) | 2 | | 1 |  | −0.90 | −1.50 | −0.40 |  |
|  |  | Social Cohesion and Social Capital | metabolic and inflammatory-related risk factors | overall | Beta (95%CI) | 3 | | 1 |  | −0.50 | −1.00 | 0.10 |  |
|  |  | Economic and Social Disadvantage | metabolic and inflammatory-related risk factors | overall | Beta (95%CI) | 4 | | 1 |  | 0.50 | −1.50 | 2.50 |  |
|  |  | Crime and Safety | metabolic and inflammatory-related risk factors | overall | Beta (95%CI) | 5 | | 1 |  | −2.20 | −4.00 | −0.30 |  |
|  |  | Social Cohesion and Social Capital | metabolic and inflammatory-related risk factors | overall | Beta (95%CI) | 6 | | 1 |  | −1.40 | −3.20 | 0.40 |  |
|  |  | Economic and Social Disadvantage | metabolic and inflammatory-related risk factors | overall | Beta (95%CI) | 7 | | 1 |  | −3.60 | −6.90 | −0.30 |  |
|  |  | Crime and Safety | metabolic and inflammatory-related risk factors | overall | Beta (95%CI) | 8 | | 1 |  | 1.10 | −2.20 | 4.50 |  |
|  |  | Social Cohesion and Social Capital | metabolic and inflammatory-related risk factors | overall | Beta (95%CI) | 9 | | 1 |  | 0.40 | −2.70 | 3.60 |  |
|  |  | Economic and Social Disadvantage | metabolic and inflammatory-related risk factors | overall | Mean difference (95%CI) | 10 | | 1 |  | 0.06 | 0.02 | 0.09 |  |
|  |  | Crime and Safety | metabolic and inflammatory-related risk factors | overall | Mean difference (95%CI) | 11 | | 1 |  | −0.04 | −0.07 | −0.01 |  |
|  |  | Social Cohesion and Social Capital | metabolic and inflammatory-related risk factors | overall | Mean difference (95%CI) | 12 | | 1 |  | −0.01 | −0.04 | 0.02 |  |
| [126] | Neergheen, 2019 | Social Cohesion and Social Capital | metabolic and inflammatory-related risk factors | female | Beta (SE) | 1 | | 1 |  | 0.02 | - | - | 0.05 |
|  |  | Social Cohesion and Social Capital | metabolic and inflammatory-related risk factors | female | Beta (SE) | 2 | | 1 |  | −0.15 | - | - | 0.05 |
|  |  | Social Cohesion and Social Capital | metabolic and inflammatory-related risk factors | male | Beta (SE) | 3 | | 1 |  | −0.03 | - | - | 0.08 |
|  |  | Social Cohesion and Social Capital | metabolic and inflammatory-related risk factors | male | Beta (SE) | 4 | | 1 |  | 0.01 | - | - | 0.07 |
|  |  | Social Cohesion and Social Capital | metabolic and inflammatory-related risk factors | overall | Beta | 5 | | 1 |  | -0.06 | - | - |  |
| [127] | Ngo, 2013 | Economic and Social Disadvantage | CVD risk scores | overall | PR (95%CI) | 1 | | 1 |  | 0.98 | 0.96 | 0.99 |  |
|  |  | Economic and Social Disadvantage | CVD risk scores | overall | PR (95%CI) | 2 | | 1 |  | 0.89 | 0.75 | 1.07 |  |
| [128] | Ngo, 2014 | Economic and Social Disadvantage | CVD risk scores | overall | OR (95%CI) | 1 | | 1 |  | 0.75 | 0.63 | 0.90 |  |
|  |  | Economic and Social Disadvantage | CVD risk scores | overall | OR (95%CI) | 2 | | 1 |  | 1.53 | 1.30 | 1.80 |  |
|  |  | Economic and Social Disadvantage | CVD risk scores | overall | OR (95%CI) | 3 | | 1 |  | 0.55 | 0.43 | 0.70 |  |
|  |  | Economic and Social Disadvantage | CVD risk scores | overall | OR (95%CI) | 4 | | 1 |  | 0.84 | 0.67 | 1.06 |  |
|  |  | Economic and Social Disadvantage | CVD risk scores | overall | PR (95%CI) | 5 | | 1 |  | 0.96 | 0.93 | 0.99 |  |
|  |  | Economic and Social Disadvantage | CVD risk scores | overall | PR (95%CI) | 6 | | 1 |  | 1.07 | 1.05 | 1.10 |  |
|  |  | Economic and Social Disadvantage | CVD risk scores | overall | PR (95%CI) | 7 | | 1 |  | 0.92 | 0.89 | 0.95 |  |
|  |  | Economic and Social Disadvantage | CVD risk scores | overall | PR (95%CI) | 8 | | 1 |  | 0.97 | 0.93 | 1.01 |  |
| [129] | Nikulina, 2014 | Economic and Social Disadvantage | metabolic and inflammatory-related risk factors | overall | Beta (95%CI) | 1 | | 1 |  | −0.07 | −0.40 | 0.21 |  |
| [130] | Nordstrom, 2004 | Economic and Social Disadvantage | CVD risk scores | overall | OR (95%CI) | 1 | | 1 | tertile 1 | 1.25 | 0.97 | 1.60 |  |
|  |  |  |  | overall |  | 1 | | 2 | tertile 2 | 1.12 | 0.90 | 1.39 |  |
|  |  |  |  | overall |  | 1 | | 3 | tertile 3 - reference | 1.00 | - | - |  |
| [131] | Ribeiro, 2019 | Economic and Social Disadvantage | CVD risk scores | overall | unclear | 1 | | 1 | Q1 - least deprived | 0.47 | - | - | 0.75 |
|  |  |  |  | overall |  | 1 | | 2 | Q2 | 0.50 | - | - | 0.77 |
|  |  |  |  | overall |  | 1 | | 3 | Q3 | 0.49 | - | - | 0.76 |
|  |  |  |  | overall |  | 1 | | 4 | Q4 | 0.48 | - | - | 0.75 |
|  |  |  |  | overall |  | 1 | | 5 | Q5 | 0.51 | - | - | 0.75 |
|  |  | Economic and Social Disadvantage | CVD risk scores | overall | unclear | 2 | | 1 | Q1 - least deprived | 1.95 | - | - | 1.54 |
|  |  |  |  | overall |  | 2 | | 2 | Q2 | 2.13 | - | - | 1.59 |
|  |  |  |  | overall |  | 2 | | 3 | Q3 | 2.19 | - | - | 1.63 |
|  |  |  |  | overall |  | 2 | | 4 | Q4 | 2.20 | - | - | 1.62 |
|  |  |  |  | overall |  | 2 | | 5 | Q5 | 2.29 | - | - | 1.65 |
|  |  | Economic and Social Disadvantage | CVD risk scores | overall | unclear | 3 | | 1 | Q1 - least deprived | 0.21 | - | - | 0.41 |
|  |  |  |  | overall |  | 3 | | 2 | Q2 | 0.24 | - | - | 0.43 |
|  |  |  |  | overall |  | 3 | | 3 | Q3 | 0.24 | - | - | 0.42 |
|  |  |  |  | overall |  | 3 | | 4 | Q4 | 0.26 | - | - | 0.44 |
|  |  |  |  | overall |  | 3 | | 5 | Q5 | 0.28 | - | - | 0.45 |
|  |  | Economic and Social Disadvantage | CVD risk scores | overall | unclear | 4 | | 1 | Q1 - least deprived | 0.40 | - | - | 0.68 |
|  |  |  |  | overall |  | 4 | | 2 | Q2 | 0.44 | - | - | 0.69 |
|  |  |  |  | overall |  | 4 | | 3 | Q3 | 0.43 | - | - | 0.70 |
|  |  |  |  | overall |  | 4 | | 4 | Q4 | 0.52 | - | - | 0.75 |
|  |  |  |  | overall |  | 4 | | 5 | Q5 | 0.52 | - | - | 0.76 |
|  |  | Economic and Social Disadvantage | CVD risk scores | overall | unclear | 5 | | 1 | Q1 - least deprived | 1.64 | - | - | 1.59 |
|  |  |  |  | overall |  | 5 | | 2 | Q2 | 1.65 | - | - | 1.58 |
|  |  |  |  | overall |  | 5 | | 3 | Q3 | 1.65 | - | - | 1.65 |
|  |  |  |  | overall |  | 5 | | 4 | Q4 | 1.82 | - | - | 1.67 |
|  |  |  |  | overall |  | 5 | | 5 | Q5 | 1.82 | - | - | 1.60 |
|  |  | Economic and Social Disadvantage | CVD risk scores | overall | unclear | 6 | | 1 | Q1 - least deprived | 0.23 | - | - | 0.42 |
|  |  |  |  | overall |  | 6 | | 2 | Q2 | 0.24 | - | - | 0.43 |
|  |  |  |  | overall |  | 6 | | 3 | Q3 | 0.23 | - | - | 0.42 |
|  |  |  |  | overall |  | 6 | | 4 | Q4 | 0.26 | - | - | 0.44 |
|  |  |  |  | overall |  | 6 | | 5 | Q5 | 0.30 | - | - | 0.46 |
|  |  | Economic and Social Disadvantage | CVD risk scores | overall | unclear | 7 | | 1 | Q1 - least deprived | 0.44 | - | - | 0.71 |
|  |  |  |  | overall |  | 7 | | 2 | Q2 | 0.48 | - | - | 0.75 |
|  |  |  |  | overall |  | 7 | | 3 | Q3 | 0.46 | - | - | 0.74 |
|  |  |  |  | overall |  | 7 | | 4 | Q4 | 0.49 | - | - | 0.75 |
|  |  |  |  | overall |  | 7 | | 5 | Q5 | 0.50 | - | - | 0.76 |
|  |  | Economic and Social Disadvantage | CVD risk scores | overall | unclear | 8 | | 1 | Q1 - least deprived | 1.64 | - | - | 1.67 |
|  |  |  |  | overall |  | 8 | | 2 | Q2 | 1.73 | - | - | 1.66 |
|  |  |  |  | overall |  | 8 | | 3 | Q3 | 1.75 | - | - | 1.67 |
|  |  |  |  | overall |  | 8 | | 4 | Q4 | 1.71 | - | - | 1.73 |
|  |  |  |  | overall |  | 8 | | 5 | Q5 | 1.79 | - | - | 1.72 |
|  |  | Economic and Social Disadvantage | CVD risk scores | overall | unclear | 9 | | 1 | Q1 - least deprived | 0.22 | - | - | 0.42 |
|  |  |  |  | overall |  | 9 | | 2 | Q2 | 0.23 | - | - | 0.42 |
|  |  |  |  | overall |  | 9 | | 3 | Q3 | 0.25 | - | - | 0.43 |
|  |  |  |  | overall |  | 9 | | 4 | Q4 | 0.27 | - | - | 0.44 |
|  |  |  |  | overall |  | 9 | | 5 | Q5 | 0.29 | - | - | 0.46 |
|  |  | Economic and Social Disadvantage | CVD risk scores | overall | RR (95%CI) | 10 | | 1 | Q1 - least deprived | 1.00 | - | - |  |
|  |  |  |  | overall |  | 10 | | 2 | Q2 | 1.06 | 1.03 | 1.09 |  |
|  |  |  |  | overall |  | 10 | | 3 | Q3 | na | na | na |  |
|  |  |  |  | overall |  | 10 | | 4 | Q4 | na | na | na |  |
|  |  |  |  | overall |  | 10 | | 5 | Q5 | 1.13 | 1.09 | 1.16 |  |
| [132] | Pedersen, 2016 | Social Relationships and Norms | CVD risk scores | female | OR (95%CI) | 1 | | 1 | yes - reference | 1.00 | - | - |  |
|  |  |  |  | female |  | 1 | | 2 | no | 1.01 | 0.73 | 1.42 |  |
|  |  | Social Relationships and Norms | CVD risk scores | female | OR (95%CI) | 2 | | 1 | yes - reference | 1.00 | - | - |  |
|  |  |  |  | female |  | 2 | | 2 | no | 1.65 | 0.86 | 3.13 |  |
|  |  | Social Relationships and Norms | CVD risk scores | female | OR (95%CI) | 3 | | 1 | yes - reference | 1.00 | - | - |  |
|  |  |  |  | female |  | 3 | | 2 | no | 1.35 | 0.88 | 2.07 |  |
|  |  | Social Relationships and Norms | CVD risk scores | female | OR (95%CI) | 4 | | 1 | yes - reference | 1.00 | - | - |  |
|  |  |  |  | female |  | 4 | | 2 | no | 1.94 | 1.07 | 3.53 |  |
|  |  | Social Relationships and Norms | CVD risk scores | female | OR (95%CI) | 5 | | 1 | yes - reference | 1.00 | - | - |  |
|  |  |  |  | female |  | 5 | | 2 | Somewhat/not at all | 1.53 | 1.11 | 2.11 |  |
|  |  | Social Relationships and Norms | CVD risk scores | male | OR (95%CI) | 6 | | 1 | yes - reference | 1.00 | - | - |  |
|  |  |  |  | male |  | 6 | | 2 | no | 1.21 | 0.77 | 1.89 |  |
|  |  | Social Relationships and Norms | CVD risk scores | male | OR (95%CI) | 7 | | 1 | yes - reference | 1.00 | - | - |  |
|  |  |  |  | male |  | 7 | | 2 | no | 1.00 | 0.54 | 1.84 |  |
|  |  | Social Relationships and Norms | CVD risk scores | male | OR (95%CI) | 8 | | 1 | yes - reference | 1.00 | - | - |  |
|  |  |  |  | male |  | 8 | | 2 | no | 0.52 | 0.27 | 0.99 |  |
|  |  | Social Relationships and Norms | CVD risk scores | male | OR (95%CI) | 9 | | 1 | yes - reference | 1.00 | - | - |  |
|  |  |  |  | male |  | 9 | | 2 | no | 1.09 | 0.48 | 2.49 |  |
|  |  | Social Relationships and Norms | CVD risk scores | male | OR (95%CI) | 10 | | 1 | yes - reference | 1.00 | - | - |  |
|  |  |  |  | male |  | 10 | | 2 | Somewhat/not at all | 1.19 | 0.81 | 1.75 |  |
| [133] | Petersen, 2008 | Economic and Social Disadvantage | metabolic and inflammatory-related risk factors | overall | Beta (SE) | 1 | | 1 | community socioeconomic status | -0.14 | - | - | 0.01 |
|  |  | Economic and Social Disadvantage | metabolic and inflammatory-related risk factors | overall | Beta (SE) | 2 | | 1 | community socioeconomic status | -0.02 | - | - | 0.01 |
| [134] | Piferi, 2006 | Social Relationships and Norms | cardiovascular health-related risk factors | overall | Beta (SE) | 1 | | 1 |  | −0.04 | - | - | 0.01 |
|  |  | Social Relationships and Norms | cardiovascular health-related risk factors | overall | Beta (SE) | 2 | | 1 |  | −0.01 | - | - | 0.05 |
|  |  | Social Relationships and Norms | cardiovascular health-related risk factors | overall | Beta (SE) | 3 | | 1 |  | −0.02 | - | - | 0.04 |
|  |  | Social Relationships and Norms | cardiovascular health-related risk factors | overall | Beta (SE) | 4 | | 1 |  | −0.01 | - | - | 0.03 |
| [135] | Pollack, 2012 | Economic and Social Disadvantage | CVD risk scores | overall | Beta (95%CI) | 1 | | 1 |  | −0.01 | −0.02 | −0.005 |  |
|  |  | Economic and Social Disadvantage | CVD risk scores | overall | Beta (95%CI) | 2 | | 1 |  | −0.03 | −0.03 | −0.02 |  |
| [136] | Pollard, 2003 | Social Relationships and Norms | cardiovascular health-related risk factors | overall | Mean | 1 | | 1 | fewest | 127.20 | - | - |  |
|  |  |  |  | overall |  | 1 | | 2 | intermediate | 125.40 | - | - |  |
|  |  |  |  | overall |  | 1 | | 3 | most | 127.90 | - | - |  |
|  |  | Social Relationships and Norms | cardiovascular health-related risk factors | overall | Mean | 2 | | 1 | fewest | 73.80 | - | - |  |
|  |  |  |  | overall |  | 2 | | 2 | intermediate | 72.70 | - | - |  |
|  |  |  |  | overall |  | 2 | | 3 | most | 75.30 | - | - |  |
|  |  | Social Relationships and Norms | metabolic and inflammatory-related risk factors | overall | Mean | 3 | | 1 | fewest | 4.17 | - | - |  |
|  |  |  |  | overall |  | 3 | | 2 | intermediate | 3.90 | - | - |  |
|  |  |  |  | overall |  | 3 | | 3 | most | 4.05 | - | - |  |
|  |  | Civic Participation and Engagement | cardiovascular health-related risk factors | overall | Mean | 4 | | 1 | Never or rarely | 126.60 | - | - |  |
|  |  |  |  | overall |  | 4 | | 2 | At least once a month | 127.70 | - | - |  |
|  |  | Civic Participation and Engagement | cardiovascular health-related risk factors | overall | Mean | 5 | | 1 | Never or rarely | 73.40 | - | - |  |
|  |  |  |  | overall |  | 5 | | 2 | At least once a month | 75.30 | - | - |  |
|  |  | Civic Participation and Engagement | metabolic and inflammatory-related risk factors | overall | Mean | 6 | | 1 | Never or rarely | 4.01 | - | - |  |
|  |  |  |  | overall |  | 6 | | 2 | At least once a month | 3.95 | - | - |  |
|  |  | Social Relationships and Norms | cardiovascular health-related risk factors | overall | Mean | 7 | | 1 | fewest | 117.00 | - | - |  |
|  |  |  |  | overall |  | 7 | | 2 | intermediate | 118.70 | - | - |  |
|  |  |  |  | overall |  | 7 | | 3 | most | 118.30 | - | - |  |
|  |  | Social Relationships and Norms | cardiovascular health-related risk factors | overall | Mean | 8 | | 1 | fewest | 68.20 | - | - |  |
|  |  |  |  | overall |  | 8 | | 2 | intermediate | 69.80 | - | - |  |
|  |  |  |  | overall |  | 8 | | 3 | most | 69.70 | - | - |  |
|  |  | Social Relationships and Norms | metabolic and inflammatory-related risk factors | overall | Mean | 9 | | 1 | fewest | 4.40 | - | - |  |
|  |  |  |  | overall |  | 9 | | 2 | intermediate | 4.51 | - | - |  |
|  |  |  |  | overall |  | 9 | | 3 | most | 4.58 | - | - |  |
|  |  | Civic Participation and Engagement | cardiovascular health-related risk factors | overall | Mean | 10 | | 1 | Never or rarely | 117.50 | - | - |  |
|  |  |  |  | overall |  | 10 | | 2 | At least once a month | 118.30 | - | - |  |
|  |  | Civic Participation and Engagement | cardiovascular health-related risk factors | overall | Mean | 11 | | 1 | Never or rarely | 69.50 | - | - |  |
|  |  |  |  | overall |  | 11 | | 2 | At least once a month | 69.20 | - | - |  |
|  |  | Civic Participation and Engagement | metabolic and inflammatory-related risk factors | overall | Mean | 12 | | 1 | Never or rarely | 4.53 | - | - |  |
|  |  |  |  | overall |  | 12 | | 2 | At least once a month | 4.48 | - | - |  |
| [137] | Pollitt, 2008 | Economic and Social Disadvantage | metabolic and inflammatory-related risk factors | overall | Geometric Mean (95% CI) | 1 | | 1 | 0 | 286.00 | 283.00 | 288.00 |  |
|  |  |  |  | overall |  | 1 | | 2 | 1 | 287.00 | 285.00 | 289.00 |  |
|  |  |  |  | overall |  | 1 | | 3 | 2 | 290.00 | 287.00 | 293.00 |  |
|  |  |  |  | overall |  | 1 | | 4 | 3 | 295.00 | 291.00 | 298.00 |  |
|  |  | Economic and Social Disadvantage | metabolic and inflammatory-related risk factors | overall | Geometric Mean (95% CI) | 2 | | 1 | 0 | 5.90 | 5.80 | 5.90 |  |
|  |  |  |  | overall |  | 2 | | 2 | 1 | 5.90 | 5.80 | 6.00 |  |
|  |  |  |  | overall |  | 2 | | 3 | 2 | 6.00 | 5.90 | 6.10 |  |
|  |  |  |  | overall |  | 2 | | 4 | 3 | 6.00 | 5.90 | 6.10 |  |
|  |  | Economic and Social Disadvantage | metabolic and inflammatory-related risk factors | overall | Geometric Mean (95% CI) | 3 | | 1 | 0 | 1.50 | 1.30 | 1.70 |  |
|  |  |  |  | overall |  | 3 | | 2 | 1 | 1.70 | 1.60 | 1.90 |  |
|  |  |  |  | overall |  | 3 | | 3 | 2 | 1.70 | 1.50 | 2.00 |  |
|  |  |  |  | overall |  | 3 | | 4 | 3 | 1.90 | 1.50 | 2.30 |  |
|  |  | Economic and Social Disadvantage | metabolic and inflammatory-related risk factors | overall | Geometric Mean (95% CI) | 4 | | 1 | high | 283.00 | 280.00 | 285.00 |  |
|  |  |  |  | overall |  | 4 | | 2 | middle | 287.00 | 285.00 | 289.00 |  |
|  |  |  |  | overall |  | 4 | | 3 | low | 293.00 | 291.00 | 295.00 |  |
|  |  | Economic and Social Disadvantage | metabolic and inflammatory-related risk factors | overall | Geometric Mean (95% CI) | 5 | | 1 | high | 5.80 | 5.70 | 5.90 |  |
|  |  |  |  | overall |  | 5 | | 2 | middle | 5.90 | 5.90 | 6.00 |  |
|  |  |  |  | overall |  | 5 | | 3 | low | 6.00 | 6.00 | 6.10 |  |
|  |  | Economic and Social Disadvantage | metabolic and inflammatory-related risk factors | overall | Geometric Mean (95% CI) | 6 | | 1 | high | 1.40 | 1.30 | 1.60 |  |
|  |  |  |  | overall |  | 6 | | 2 | middle | 1.60 | 1.40 | 1.80 |  |
|  |  |  |  | overall |  | 6 | | 3 | low | 1.90 | 1.70 | 2.20 |  |
|  |  | Economic and Social Disadvantage | metabolic and inflammatory-related risk factors | overall | Geometric Mean (95% CI) | 7 | | 1 | 0 | 305.00 | 300.00 | 310.00 |  |
|  |  |  |  | overall |  | 7 | | 2 | 1 | 305.00 | 301.00 | 310.00 |  |
|  |  |  |  | overall |  | 7 | | 3 | 2 | 311.00 | 306.00 | 317.00 |  |
|  |  |  |  | overall |  | 7 | | 4 | 3 | 312.00 | 305.00 | 320.00 |  |
|  |  | Economic and Social Disadvantage | metabolic and inflammatory-related risk factors | overall | Geometric Mean (95% CI) | 8 | | 1 | 0 | 5.30 | 5.10 | 5.40 |  |
|  |  |  |  | overall |  | 8 | | 2 | 1 | 5.20 | 5.10 | 5.30 |  |
|  |  |  |  | overall |  | 8 | | 3 | 2 | 5.30 | 5.10 | 5.40 |  |
|  |  |  |  | overall |  | 8 | | 4 | 3 | 5.30 | 5.10 | 5.50 |  |
|  |  | Economic and Social Disadvantage | metabolic and inflammatory-related risk factors | overall | Geometric Mean (95% CI) | 9 | | 1 | 0 | 1.80 | 1.30 | 2.60 |  |
|  |  |  |  | overall |  | 9 | | 2 | 1 | 2.40 | 1.70 | 3.30 |  |
|  |  |  |  | overall |  | 9 | | 3 | 2 | 2.10 | 1.40 | 3.30 |  |
|  |  |  |  | overall |  | 9 | | 4 | 3 | 2.10 | 1.30 | 3.50 |  |
|  |  | Economic and Social Disadvantage | metabolic and inflammatory-related risk factors | overall | Geometric Mean (95% CI) | 10 | | 1 | high | 305.00 | 300.00 | 309.00 |  |
|  |  |  |  | overall |  | 10 | | 2 | middle | 305.00 | 301.00 | 309.00 |  |
|  |  |  |  | overall |  | 10 | | 3 | low | 311.00 | 308.00 | 315.00 |  |
|  |  | Economic and Social Disadvantage | metabolic and inflammatory-related risk factors | overall | Geometric Mean (95% CI) | 11 | | 1 | high | 5.20 | 5.10 | 5.30 |  |
|  |  |  |  | overall |  | 11 | | 2 | middle | 5.20 | 5.10 | 5.30 |  |
|  |  |  |  | overall |  | 11 | | 3 | low | 5.30 | 5.20 | 5.40 |  |
|  |  | Economic and Social Disadvantage | metabolic and inflammatory-related risk factors | overall | Geometric Mean (95% CI) | 12 | | 1 | high | 1.70 | 1.30 | 2.40 |  |
|  |  |  |  | overall |  | 12 | | 2 | middle | 2.20 | 1.60 | 2.90 |  |
|  |  |  |  | overall |  | 12 | | 3 | low | 2.40 | 1.90 | 3.10 |  |
| [138] | Pollitt, 2007 | Economic and Social Disadvantage | metabolic and inflammatory-related risk factors | overall | Beta (95%CI) | 1 | | 1 |  | 1.07 | 0.85 | 1.35 |  |
|  |  |  |  | overall | Beta (95%CI) | 1 | | 2 |  | 1.12 | 0.91 | 1.38 |  |
|  |  | Economic and Social Disadvantage | metabolic and inflammatory-related risk factors | overall | Beta (95%CI) | 2 | | 1 |  | 1.01 | 1.00 | 1.03 |  |
|  |  |  |  | overall | Beta (95%CI) | 2 | | 2 |  | 1.00 | 0.99 | 1.02 |  |
|  |  | Economic and Social Disadvantage | metabolic and inflammatory-related risk factors | overall | Beta (95%CI) | 3 | | 1 |  | 1.00 | 0.97 | 1.03 |  |
|  |  |  |  | overall | Beta (95%CI) | 3 | | 2 |  | 1.01 | 0.99 | 1.03 |  |
|  |  | Economic and Social Disadvantage | metabolic and inflammatory-related risk factors | overall | Beta (95%CI) | 4 | | 1 |  | 1.05 | 0.86 | 1.27 |  |
|  |  |  |  | overall | Beta (95%CI) | 4 | | 2 |  | 1.24 | 1.03 | 1.50 |  |
|  |  | Economic and Social Disadvantage | metabolic and inflammatory-related risk factors | overall | Beta (95%CI) | 5 | | 1 |  | 1.01 | 1.00 | 1.02 |  |
|  |  |  |  | overall | Beta (95%CI) | 5 | | 2 |  | 1.03 | 1.02 | 1.04 |  |
|  |  | Economic and Social Disadvantage | metabolic and inflammatory-related risk factors | overall | Beta (95%CI) | 6 | | 1 |  | 1.02 | 1.00 | 1.04 |  |
|  |  |  |  | overall | Beta (95%CI) | 6 | | 2 |  | 1.02 | 1.01 | 1.04 |  |
|  |  | Economic and Social Disadvantage | metabolic and inflammatory-related risk factors | overall | Beta (95%CI) | 7 | | 1 |  | 1.05 | 0.83 | 1.33 |  |
|  |  |  |  | overall | Beta (95%CI) | 7 | | 2 |  | 1.07 | 0.88 | 1.30 |  |
|  |  | Economic and Social Disadvantage | metabolic and inflammatory-related risk factors | overall | Beta (95%CI) | 8 | | 1 |  | 1.01 | 0.99 | 1.02 |  |
|  |  |  |  | overall | Beta (95%CI) | 8 | | 2 |  | 1.00 | 0.99 | 1.01 |  |
|  |  | Economic and Social Disadvantage | metabolic and inflammatory-related risk factors | overall | Beta (95%CI) | 9 | | 1 |  | 0.99 | 0.97 | 1.02 |  |
|  |  |  |  | overall | Beta (95%CI) | 9 | | 2 |  | 1.00 | 0.98 | 1.02 |  |
|  |  | Economic and Social Disadvantage | metabolic and inflammatory-related risk factors | overall | Beta (95%CI) | 10 | | 1 |  | 1.01 | 0.84 | 1.21 |  |
|  |  |  |  | overall | Beta (95%CI) | 10 | | 2 |  | 1.16 | 0.97 | 1.40 |  |
|  |  | Economic and Social Disadvantage | metabolic and inflammatory-related risk factors | overall | Beta (95%CI) | 11 | | 1 |  | 1.01 | 1.00 | 1.02 |  |
|  |  |  |  | overall | Beta (95%CI) | 11 | | 2 |  | 1.03 | 1.01 | 1.04 |  |
|  |  | Economic and Social Disadvantage | metabolic and inflammatory-related risk factors | overall | Beta (95%CI) | 12 | | 1 |  | 1.01 | 0.99 | 1.03 |  |
|  |  |  |  | overall | Beta (95%CI) | 12 | | 2 |  | 1.01 | 0.99 | 1.03 |  |
|  |  | Economic and Social Disadvantage | metabolic and inflammatory-related risk factors | overall | Beta (95%CI) | 13 | | 1 |  | 1.46 | 0.84 | 2.54 |  |
|  |  |  |  | overall | Beta (95%CI) | 13 | | 2 |  | 1.41 | 0.81 | 2.45 |  |
|  |  | Economic and Social Disadvantage | metabolic and inflammatory-related risk factors | overall | Beta (95%CI) | 14 | | 1 |  | 1.00 | 0.98 | 1.03 |  |
|  |  |  |  | overall | Beta (95%CI) | 14 | | 2 |  | 1.00 | 0.98 | 1.03 |  |
|  |  | Economic and Social Disadvantage | metabolic and inflammatory-related risk factors | overall | Beta (95%CI) | 15 | | 1 |  | 1.02 | 0.98 | 1.06 |  |
|  |  |  |  | overall | Beta (95%CI) | 15 | | 2 |  | 1.00 | 0.96 | 1.04 |  |
|  |  | Economic and Social Disadvantage | metabolic and inflammatory-related risk factors | overall | Beta (95%CI) | 16 | | 1 |  | 1.28 | 0.82 | 2.00 |  |
|  |  |  |  | overall | Beta (95%CI) | 16 | | 2 |  | 1.31 | 0.85 | 2.03 |  |
|  |  | Economic and Social Disadvantage | metabolic and inflammatory-related risk factors | overall | Beta (95%CI) | 17 | | 1 |  | 1.00 | 0.98 | 1.02 |  |
|  |  |  |  | overall | Beta (95%CI) | 17 | | 2 |  | 1.02 | 1.00 | 1.04 |  |
|  |  | Economic and Social Disadvantage | metabolic and inflammatory-related risk factors | overall | Beta (95%CI) | 18 | | 1 |  | 1.00 | 0.97 | 1.03 |  |
|  |  |  |  | overall | Beta (95%CI) | 18 | | 2 |  | 1.01 | 0.98 | 1.04 |  |
|  |  | Economic and Social Disadvantage | metabolic and inflammatory-related risk factors | overall | Beta (95%CI) | 19 | | 1 |  | 1.33 | 0.78 | 2.26 |  |
|  |  |  |  | overall | Beta (95%CI) | 19 | | 2 |  | 1.24 | 0.73 | 2.10 |  |
|  |  | Economic and Social Disadvantage | metabolic and inflammatory-related risk factors | overall | Beta (95%CI) | 20 | | 1 |  | 1.01 | 0.98 | 1.03 |  |
|  |  |  |  | overall | Beta (95%CI) | 20 | | 2 |  | 1.00 | 0.98 | 1.02 |  |
|  |  | Economic and Social Disadvantage | metabolic and inflammatory-related risk factors | overall | Beta (95%CI) | 21 | | 1 |  | 1.02 | 0.98 | 1.05 |  |
|  |  |  |  | overall | Beta (95%CI) | 21 | | 2 |  | 0.99 | 0.96 | 1.03 |  |
|  |  | Economic and Social Disadvantage | metabolic and inflammatory-related risk factors | overall | Beta (95%CI) | 22 | | 1 |  | 1.23 | 0.79 | 1.92 |  |
|  |  |  |  | overall | Beta (95%CI) | 22 | | 2 |  | 1.35 | 0.87 | 2.09 |  |
|  |  | Economic and Social Disadvantage | metabolic and inflammatory-related risk factors | overall | Beta (95%CI) | 23 | | 1 |  | 1.00 | 0.98 | 1.02 |  |
|  |  |  |  | overall | Beta (95%CI) | 23 | | 2 |  | 1.01 | 0.99 | 1.03 |  |
|  |  | Economic and Social Disadvantage | metabolic and inflammatory-related risk factors | overall | Beta (95%CI) | 24 | | 1 |  | 1.00 | 0.97 | 1.03 |  |
|  |  |  |  | overall | Beta (95%CI) | 24 | | 2 |  | 1.01 | 0.98 | 1.05 |  |
|  |  | Economic and Social Disadvantage | metabolic and inflammatory-related risk factors | overall | Beta (95%CI) | 25 | | 1 |  | 0.07 | -0.05 | 0.20 |  |
|  |  |  |  | overall | Beta (95%CI) | 25 | | 2 |  | 0.03 | -0.07 | 0.11 |  |
|  |  | Economic and Social Disadvantage | metabolic and inflammatory-related risk factors | overall | Beta (95%CI) | 26 | | 1 |  | 0.09 | 0.00 | 0.19 |  |
|  |  |  |  | overall | Beta (95%CI) | 26 | | 2 |  | 0.17 | 0.08 | 0.26 |  |
|  |  | Economic and Social Disadvantage | metabolic and inflammatory-related risk factors | overall | Beta (95%CI) | 27 | | 1 |  | 0.15 | -0.13 | 0.43 |  |
|  |  |  |  | overall | Beta (95%CI) | 27 | | 2 |  | 0.08 | -0.20 | 0.36 |  |
|  |  | Economic and Social Disadvantage | metabolic and inflammatory-related risk factors | overall | Beta (95%CI) | 28 | | 1 |  | 0.14 | -0.09 | 0.36 |  |
|  |  |  |  | overall | Beta (95%CI) | 28 | | 2 |  | 0.31 | 0.08 | 0.53 |  |
|  |  | Economic and Social Disadvantage | metabolic and inflammatory-related risk factors | overall | Beta (95%CI) | 29 | | 1 |  | 0.06 | -0.07 | 0.18 |  |
|  |  |  |  | overall | Beta (95%CI) | 29 | | 2 |  | 0.00 | -0.10 | 0.10 |  |
|  |  | Economic and Social Disadvantage | metabolic and inflammatory-related risk factors | overall | Beta (95%CI) | 30 | | 1 |  | 0.09 | 0.01 | 0.18 |  |
|  |  |  |  | overall | Beta (95%CI) | 30 | | 2 |  | 0.16 | 0.08 | 0.24 |  |
|  |  | Economic and Social Disadvantage | metabolic and inflammatory-related risk factors | overall | Beta (95%CI) | 31 | | 1 |  | 0.11 | -0.16 | 0.38 |  |
|  |  |  |  | overall | Beta (95%CI) | 31 | | 2 |  | 0.01 | -0.26 | 0.28 |  |
|  |  | Economic and Social Disadvantage | metabolic and inflammatory-related risk factors | overall | Beta (95%CI) | 32 | | 1 |  | 0.16 | -0.07 | 0.38 |  |
|  |  |  |  | overall | Beta (95%CI) | 32 | | 2 |  | 0.34 | 0.12 | 0.57 |  |
| [139] | Riva, 2016 | Economic and Social Disadvantage | cardiovascular health-related risk factors | overall | Beta | 1 | | 1 | Town, mid socioeconomic status - reference | 1.00 | - | - |  |
|  |  |  |  | overall |  | 1 | | 2 | Town, high socioeconomic status | -3.61 | -5.24 | -1.98 |  |
|  |  |  |  | overall |  | 1 | | 3 | Village, low socioeconomic status | -3.34 | -5.45 | -1.22 |  |
|  |  |  |  | overall |  | 1 | | 4 | Village, middle/high socioeconomic status | -1.69 | -4.48 | 1.11 |  |
|  |  | Economic and Social Disadvantage | cardiovascular health-related risk factors | overall | Beta | 2 | | 1 | Town, mid socioeconomic status - reference | 1.00 | - | - |  |
|  |  |  |  | overall |  | 2 | | 2 | Town, high socioeconomic status | -4.11 | -6.71 | -1.51 |  |
|  |  |  |  | overall |  | 2 | | 3 | Village, low socioeconomic status | -4.91 | -7.62 | -2.19 |  |
|  |  |  |  | overall |  | 2 | | 4 | Village, middle/high socioeconomic status | -3.97 | -7.09 | -0.85 |  |
|  |  | Economic and Social Disadvantage | cardiovascular health-related risk factors | male | Beta | 3 | | 1 | Town, mid socioeconomic status - reference | 1.00 | - | - |  |
|  |  |  |  | male |  | 3 | | 2 | Town, high socioeconomic status | -5.49 | -9.19 | -1.80 |  |
|  |  |  |  | male |  | 3 | | 3 | Village, low socioeconomic status | -6.08 | -10.30 | -1.89 |  |
|  |  |  |  | male |  | 3 | | 4 | Village, middle/high socioeconomic status | -4.80 | -9.82 | 0.22 |  |
|  |  | Economic and Social Disadvantage | cardiovascular health-related risk factors | female | Beta | 4 | | 1 | Town, mid socioeconomic status - reference | 1.00 | - | - |  |
|  |  |  |  | female |  | 4 | | 2 | Town, high socioeconomic status | -2.28 | -4.65 | 0.09 |  |
|  |  |  |  | female |  | 4 | | 3 | Village, low socioeconomic status | -1.19 | -4.12 | 1.74 |  |
|  |  |  |  | female |  | 4 | | 4 | Village, middle/high socioeconomic status | 1.06 | -2.79 | 4.92 |  |
|  |  | Economic and Social Disadvantage | cardiovascular health-related risk factors | male | Beta | 5 | | 1 | Town, mid socioeconomic status - reference | 1.00 | - | - |  |
|  |  |  |  | male |  | 5 | | 2 | Town, high socioeconomic status | -4.69 | -8.28 | -1.10 |  |
|  |  |  |  | male |  | 5 | | 3 | Village, low socioeconomic status | -6.07 | -9.89 | -2.26 |  |
|  |  |  |  | male |  | 5 | | 4 | Village, middle/high socioeconomic status | -5.40 | -9.77 | -1.02 |  |
|  |  | Economic and Social Disadvantage | cardiovascular health-related risk factors | female | Beta | 6 | | 1 | Town, mid socioeconomic status - reference | 1.00 | - | - |  |
|  |  |  |  | female |  | 6 | | 2 | Town, high socioeconomic status | -3.89 | -5.64 | -2.14 |  |
|  |  |  |  | female |  | 6 | | 3 | Village, low socioeconomic status | -4.53 | -6.63 | -2.44 |  |
|  |  |  |  | female |  | 6 | | 4 | Village, middle/high socioeconomic status | -3.16 | -5.84 | -0.47 |  |
| [140] | Robinette, 2016 | Economic and Social Disadvantage | CVD risk scores | overall | Beta (SE) | 1 | | 1 |  | −0.05 | - | - | 0.01 |
| [141] | Robinette, 2020 | Social Cohesion and Social Capital | CVD risk scores | overall | Beta (95%CI) | 1 | | 1 |  | −0.02 | −0.08 | 0.05 |  |
| [142] | Rosvall, 2007 | Economic and Social Disadvantage | cardiovascular health-related risk factors | male | Mean difference | 1 | | 1 | tertile 1 - reference | - | - | - |  |
|  |  |  |  | male |  | 1 | | 2 | tertile 2 | -0.21 | - | - |  |
|  |  |  |  | male |  | 1 | | 3 | tertile 3 | -0.29 | - | - |  |
|  |  | Economic and Social Disadvantage | cardiovascular health-related risk factors | female | Mean difference | 2 | | 1 | tertile 1 - reference | - | - | - |  |
|  |  |  |  | female |  | 2 | | 2 | tertile 2 | -0.10 | - | - |  |
|  |  |  |  | female |  | 2 | | 3 | tertile 3 | -0.20 | - | - |  |
| [143] | Sörman, 2016 | Social Relationships and Norms | cardiovascular health-related risk factors | overall | Beta (SE) | 1 | | 1 |  | 0.16 | - | - | 1.59 |
|  |  | Social Relationships and Norms | cardiovascular health-related risk factors | overall | Beta (SE) | 2 | | 1 |  | -0.01 | - | - | 1.26 |
|  |  | Social Relationships and Norms | cardiovascular health-related risk factors | overall | Beta (SE) | 3 | | 1 |  | 0.11 | - | - | 0.98 |
|  |  | Social Relationships and Norms | cardiovascular health-related risk factors | overall | Beta (SE) | 4 | | 1 |  | 0.06 | - | - | 1.33 |
|  |  | Social Relationships and Norms | cardiovascular health-related risk factors | overall | Beta (SE) | 5 | | 1 |  | 0.11 | - | - | 0.46 |
|  |  | Social Relationships and Norms | cardiovascular health-related risk factors | overall | Beta (SE) | 6 | | 1 |  | 0.05 | - | - | 3.07 |
|  |  | Social Relationships and Norms | cardiovascular health-related risk factors | overall | Beta (SE) | 7 | | 1 |  | -0.04 | - | - | 2.44 |
|  |  | Social Relationships and Norms | cardiovascular health-related risk factors | overall | Beta (SE) | 8 | | 1 |  | -0.02 | - | - | 1.90 |
|  |  | Social Relationships and Norms | cardiovascular health-related risk factors | overall | Beta (SE) | 9 | | 1 |  | -0.03 | - | - | 2.58 |
|  |  | Social Relationships and Norms | cardiovascular health-related risk factors | overall | Beta (SE) | 10 | | 1 |  | -0.02 | - | - | 0.90 |
|  |  | Social Relationships and Norms | cardiovascular health-related risk factors | overall | Beta (SE) | 11 | | 1 |  | -0.07 | - | - | 1.02 |
|  |  | Social Relationships and Norms | cardiovascular health-related risk factors | overall | Beta (SE) | 12 | | 1 |  | 0.02 | - | - | 0.81 |
|  |  | Social Relationships and Norms | cardiovascular health-related risk factors | overall | Beta (SE) | 13 | | 1 |  | -0.07 | - | - | 0.63 |
|  |  | Social Relationships and Norms | cardiovascular health-related risk factors | overall | Beta (SE) | 14 | | 1 |  | -0.08 | - | - | 0.86 |
|  |  | Social Relationships and Norms | cardiovascular health-related risk factors | overall | Beta (SE) | 15 | | 1 |  | -0.11 | - | - | 0.30 |
|  |  | Social Relationships and Norms | cardiovascular health-related risk factors | overall | Beta (SE) | 16 | | 1 |  | -0.06 | - | - | 2.15 |
|  |  | Social Relationships and Norms | cardiovascular health-related risk factors | overall | Beta (SE) | 17 | | 1 |  | -0.01 | - | - | 1.71 |
|  |  | Social Relationships and Norms | cardiovascular health-related risk factors | overall | Beta (SE) | 18 | | 1 |  | 0.01 | - | - | 1.33 |
|  |  | Social Relationships and Norms | cardiovascular health-related risk factors | overall | Beta (SE) | 19 | | 1 |  | 0.00 | - | - | 1.80 |
|  |  | Social Relationships and Norms | cardiovascular health-related risk factors | overall | Beta (SE) | 20 | | 1 |  | -0.04 | - | - | 0.11 |
| [144] | Samuel, 2015 | Social Cohesion and Social Capital | cardiovascular health-related risk factors | overall | Beta (SE) | 1 | | 1 |  | 2.65 | - | - | 0.19 |
|  |  | Social Cohesion and Social Capital | cardiovascular health-related risk factors | overall | Beta (SE) | 2 | | 1 |  | 1.24 | - | - | 0.35 |
|  |  | Social Cohesion and Social Capital | cardiovascular health-related risk factors | overall | Beta (SE) | 3 | | 1 |  | 4.91 | - | - | 0.01 |
|  |  | Social Cohesion and Social Capital | cardiovascular health-related risk factors | overall | Beta (SE) | 4 | | 1 |  | 3.38 | - | - | 0.01 |
| [145] | Schulz, 2013 | Economic and Social Disadvantage | CVD risk scores | overall | Beta (SE) | 1 | | 1 | 1 (least disadvantaged) | -0.33 | - | - | 0.17 |
|  |  |  |  | overall |  | 1 | | 2 | 2 | -0.09 | - | - | 0.14 |
|  |  |  |  | overall |  | 1 | | 3 | 3 | -0.38 | - | - | 0.19 |
|  |  |  |  | overall |  | 1 | | 4 | 4 | -0.17 | - | - | 0.16 |
|  |  |  |  | overall |  | 1 | | 5 | 5 (most disadvantaged) | 1.00 | - | - |  |
| [146] | Seeman, 2014 | Social Relationships and Norms | CVD risk scores | overall | Beta (SE) | 1 | | 1 | Q1 (lowest) | 4.50 | - | - | 0.23 |
|  |  |  |  | overall |  | 1 | | 2 | Q2 | 4.46 | - | - | 0.22 |
|  |  |  |  | overall |  | 1 | | 3 | Q3 | 3.98 | - | - | 0.19 |
|  |  |  |  | overall |  | 1 | | 4 | Q4 | 3.75 | - | - | 0.16 |
|  |  | Social Relationships and Norms | CVD risk scores | overall | Beta (SE) | 2 | | 1 | Q1 (lowest) | 4.56 | - | - | 0.29 |
|  |  |  |  | overall |  | 2 | | 2 | Q2 | 4.50 | - | - | 0.21 |
|  |  |  |  | overall |  | 2 | | 3 | Q3 | 3.98 | - | - | 0.16 |
|  |  |  |  | overall |  | 2 | | 4 | Q4 | 3.76 | - | - | 0.18 |
|  |  | Social Relationships and Norms | CVD risk scores | overall | Beta (SE) | 3 | | 1 | Q1 (lowest) | 3.44 | - | - | 0.20 |
|  |  |  |  | overall |  | 3 | | 2 | Q2 | 4.15 | - | - | 0.15 |
|  |  |  |  | overall |  | 3 | | 3 | Q3 | 4.24 | - | - | 0.19 |
|  |  |  |  | overall |  | 3 | | 4 | Q4 | 5.40 | - | - | 0.38 |
| [147] | Smith, 1998 | Economic and Social Disadvantage | cardiovascular health-related risk factors | male | Mean | 1 | | 1 | **1** | 85.30 | - | - |  |
|  |  |  |  | male |  | 1 | | 2 | **3** | 87.00 | - | - |  |
|  |  |  |  | male |  | 1 | | 3 | 4 | 84.50 | - | - |  |
|  |  |  |  | male |  | 1 | | 4 | 5 | 87.10 | - | - |  |
|  |  |  |  | male |  | 1 | | 5 | 6 | 84.80 | - | - |  |
|  |  |  |  | male |  | 1 | | 6 | 7 | 87.90 | - | - |  |
|  |  | Economic and Social Disadvantage | metabolic and inflammatory-related risk factors | male | Mean | 2 | | 1 | **1** | 5.83 | - | - |  |
|  |  |  |  | male |  | 2 | | 2 | **3** | 5.97 | - | - |  |
|  |  |  |  | male |  | 2 | | 3 | 4 | 5.97 | - | - |  |
|  |  |  |  | male |  | 2 | | 4 | 5 | 5.80 | - | - |  |
|  |  |  |  | male |  | 2 | | 5 | 6 | 5.79 | - | - |  |
|  |  |  |  | male |  | 2 | | 6 | 7 | 5.92 | - | - |  |
|  |  | Economic and Social Disadvantage | cardiovascular health-related risk factors | female | Mean | 3 | | 1 | **1** | 85.00 | - | - |  |
|  |  |  |  | female |  | 3 | | 2 | **3** | 86.30 | - | - |  |
|  |  |  |  | female |  | 3 | | 3 | 4 | 83.10 | - | - |  |
|  |  |  |  | female |  | 3 | | 4 | 5 | 86.30 | - | - |  |
|  |  |  |  | female |  | 3 | | 5 | 6 | 84.50 | - | - |  |
|  |  |  |  | female |  | 3 | | 6 | 7 | 85.30 | - | - |  |
|  |  | Economic and Social Disadvantage | metabolic and inflammatory-related risk factors | female | Mean | 4 | | 1 | **1** | 6.36 | - | - |  |
|  |  |  |  | female |  | 4 | | 2 | **3** | 6.49 | - | - |  |
|  |  |  |  | female |  | 4 | | 3 | 4 | 6.50 | - | - |  |
|  |  |  |  | female |  | 4 | | 4 | 5 | 6.43 | - | - |  |
|  |  |  |  | female |  | 4 | | 5 | 6 | 6.35 | - | - |  |
|  |  |  |  | female |  | 4 | | 6 | 7 | 6.35 | - | - |  |
| [148] | Sprung, 2019 | Crime and Safety | glucose metabolism-related risk factors | female | Beta | 1 | | 1 |  | 0.17 | - | - |  |
|  |  | Crime and Safety | cardiovascular health-related risk factors | female | Beta | 2 | | 1 |  | 87.44 | - | - |  |
|  |  | Crime and Safety | cardiovascular health-related risk factors | female | Beta | 3 | | 1 |  | 60.79 | - | - |  |
| [149] | Steppuhn, 2019 | Economic and Social Disadvantage | metabolic and inflammatory-related risk factors | overall | Beta (95%CI) | 1 | | 1 |  | 1.50 | -6.90 | 10.60 |  |
|  |  |  |  | overall | Beta (95%CI) | 1 | | 2 |  | 13.20 | 3.10 | 24.40 |  |
|  |  | Economic and Social Disadvantage | metabolic and inflammatory-related risk factors | overall | Beta (95%CI) | 2 | | 1 |  | -1.10 | -8.40 | 6.90 |  |
|  |  |  |  | overall | Beta (95%CI) | 2 | | 2 |  | 9.00 | 0.20 | 18.50 |  |
| [150] | Strogatz, 1997 | Social Relationships and Norms | cardiovascular health-related risk factors | female | Beta (SE) | 1 | | 1 |  | -0.60 | - | - | 0.23 |
|  |  | Social Relationships and Norms | cardiovascular health-related risk factors | female | Beta (SE) | 2 | | 1 |  | -0.43 | - | - | 0.15 |
|  |  | Social Relationships and Norms | cardiovascular health-related risk factors | female | Beta (SE) | 3 | | 1 |  | -0.33 | - | - | 0.16 |
|  |  | Social Relationships and Norms | cardiovascular health-related risk factors | female | Beta (SE) | 4 | | 1 |  | -0.22 | - | - | 0.07 |
|  |  | Social Relationships and Norms | cardiovascular health-related risk factors | male | Beta (SE) | 5 | | 1 |  | -0.57 | - | - | 0.31 |
|  |  | Social Relationships and Norms | cardiovascular health-related risk factors | male | Beta (SE) | 6 | | 1 |  | -0.21 | - | - | 0.22 |
|  |  | Social Relationships and Norms | cardiovascular health-related risk factors | male | Beta (SE) | 7 | | 1 |  | -0.45 | - | - | 0.28 |
|  |  | Social Relationships and Norms | cardiovascular health-related risk factors | male | Beta (SE) | 8 | | 1 |  | -0.03 | - | - | 0.18 |
|  |  | Social Relationships and Norms | cardiovascular health-related risk factors | female | Beta (95%CI) | 9 | | 1 |  | 3.60 | 0.80 | 6.40 |  |
|  |  | Social Relationships and Norms | cardiovascular health-related risk factors | female | Beta (95%CI) | 10 | | 1 |  | 5.20 | 1.70 | 8.70 |  |
|  |  | Social Relationships and Norms | cardiovascular health-related risk factors | female | Beta (95%CI) | 11 | | 1 |  | 2.00 | 0.10 | 3.90 |  |
|  |  | Social Relationships and Norms | cardiovascular health-related risk factors | female | Beta (95%CI) | 12 | | 1 |  | 2.70 | 1.00 | 4.40 |  |
|  |  | Social Relationships and Norms | cardiovascular health-related risk factors | male | Beta (95%CI) | 13 | | 1 |  | 3.40 | -0.30 | 7.10 |  |
|  |  | Social Relationships and Norms | cardiovascular health-related risk factors | male | Beta (95%CI) | 14 | | 1 |  | 2.50 | -2.70 | 7.70 |  |
|  |  | Social Relationships and Norms | cardiovascular health-related risk factors | male | Beta (95%CI) | 15 | | 1 |  | 2.70 | -0.60 | 6.00 |  |
|  |  | Social Relationships and Norms | cardiovascular health-related risk factors | male | Beta (95%CI) | 16 | | 1 |  | 0.40 | -3.90 | 4.70 |  |
| [151] | Theorell, 1982 | Economic and Social Disadvantage | cardiovascular health-related risk factors | male | correlation | 1 | | 1 |  | -0.43 | - | - |  |
|  |  | Economic and Social Disadvantage | cardiovascular health-related risk factors | male | correlation | 2 | | 1 |  | -0.22 | - | - |  |
|  |  | Economic and Social Disadvantage | cardiovascular health-related risk factors | male | correlation | 3 | | 1 |  | -0.12 | - | - |  |
|  |  | Economic and Social Disadvantage | cardiovascular health-related risk factors | male | correlation | 4 | | 1 |  | 0.27 | - | - |  |
|  |  | Economic and Social Disadvantage | cardiovascular health-related risk factors | male | correlation | 5 | | 1 |  | -0.30 | - | - |  |
|  |  | Economic and Social Disadvantage | cardiovascular health-related risk factors | male | correlation | 6 | | 1 |  | 0.17 | - | - |  |
| [152] | Toms, 2020 | Economic and Social Disadvantage | glucose metabolism-related risk factors | overall | OR (95%CI) | 1 | | 1 | Q5 - reference | 1.00 | - | - |  |
|  |  |  |  | overall |  | 1 | | 2 | Q4 | 1.27 | 1.18 | 1.37 |  |
|  |  |  |  | overall |  | 1 | | 3 | Q3 | 1.49 | 1.39 | 1.61 |  |
|  |  |  |  | overall |  | 1 | | 4 | Q2 | 1.62 | 1.50 | 1.74 |  |
|  |  |  |  | overall |  | 1 | | 5 | Q1 most deprived | 2.11 | 1.96 | 2.26 |  |
|  |  | Economic and Social Disadvantage | glucose metabolism-related risk factors | overall | OR (95%CI) | 2 | | 1 | Q5 - reference | 1.00 | - | - |  |
|  |  |  |  | overall |  | 2 | | 2 | Q4 | 1.15 | 1.04 | 1.28 |  |
|  |  |  |  | overall |  | 2 | | 3 | Q3 | 1.39 | 1.26 | 1.54 |  |
|  |  |  |  | overall |  | 2 | | 4 | Q2 | 1.55 | 1.41 | 1.71 |  |
|  |  |  |  | overall |  | 2 | | 5 | Q1 most deprived | 2.02 | 1.84 | 2.22 |  |
|  |  | Economic and Social Disadvantage | metabolic and inflammatory-related risk factors | overall | OR (95%CI) | 3 | | 1 | Q5 - reference | 1.00 | - | - |  |
|  |  |  |  | overall |  | 3 | | 2 | Q4 | 0.94 | 0.90 | 0.98 |  |
|  |  |  |  | overall |  | 3 | | 3 | Q3 | 0.94 | 0.90 | 0.98 |  |
|  |  |  |  | overall |  | 3 | | 4 | Q2 | 0.98 | 0.87 | 0.94 |  |
|  |  |  |  | overall |  | 3 | | 5 | Q1 most deprived | 0.84 | 0.81 | 0.88 |  |
|  |  | Economic and Social Disadvantage | metabolic and inflammatory-related risk factors | overall | OR (95%CI) | 4 | | 1 | Q5 - reference | 1.00 | - | - |  |
|  |  |  |  | overall |  | 4 | | 2 | Q4 | 1.20 | 1.13 | 1.28 |  |
|  |  |  |  | overall |  | 4 | | 3 | Q3 | 1.32 | 1.24 | 1.41 |  |
|  |  |  |  | overall |  | 4 | | 4 | Q2 | 1.51 | 1.42 | 1.61 |  |
|  |  |  |  | overall |  | 4 | | 5 | Q1 most deprived | 1.90 | 1.78 | 2.02 |  |
|  |  | Economic and Social Disadvantage | metabolic and inflammatory-related risk factors | overall | OR (95%CI) | 5 | | 1 | Q5 - reference | 1.00 | - | - |  |
|  |  |  |  | overall |  | 5 | | 2 | Q4 | 1.25 | 1.04 | 1.50 |  |
|  |  |  |  | overall |  | 5 | | 3 | Q3 | 1.27 | 1.06 | 1.51 |  |
|  |  |  |  | overall |  | 5 | | 4 | Q2 | 1.45 | 1.23 | 1.72 |  |
|  |  |  |  | overall |  | 5 | | 5 | Q1 most deprived | 1.84 | 1.56 | 2.16 |  |
| [153] | Troxel, 2010 | Civic Participation and Engagement | cardiovascular health-related risk factors | overall | Beta (SE) | 1 | | 1 |  | −0.26 | - | - | 0.00 |
|  |  | Civic Participation and Engagement | cardiovascular health-related risk factors | overall | Beta (SE) | 2 | | 1 |  | −0.33 | - | - | 0.00 |
| [154] | Tung, 2019 | Crime and Safety | cardiovascular health-related risk factors | overall | Beta (SE) | 1 | | 1 |  | 0.20 | - | - | 0.08 |
|  |  | Crime and Safety | cardiovascular health-related risk factors | overall | Beta (SE) | 2 | | 1 |  | 0.04 | - | - | 0.15 |
|  |  | Crime and Safety | cardiovascular health-related risk factors | overall | Beta (SE) | 3 | | 1 |  | 0.06 | - | - | 0.07 |
|  |  | Crime and Safety | cardiovascular health-related risk factors | overall | Beta (SE) | 4 | | 1 |  | 0.01 | - | - | 0.12 |
|  |  | Crime and Safety | cardiovascular health-related risk factors | overall | OR (95%CI) | 5 | | 1 |  | 1.01 | 0.97 | 1.05 |  |
|  |  | Crime and Safety | cardiovascular health-related risk factors | overall | OR (95%CI) | 6 | | 1 |  | 1.01 | 0.92 | 1.11 |  |
| [155] | Uchino, 2013 | Social Relationships and Norms | cardiovascular health-related risk factors | overall | Beta (SE) | 1 | | 1 |  | -0.16 | - | - | 0.06 |
|  |  | Social Relationships and Norms | cardiovascular health-related risk factors | overall | Beta (SE) | 2 | | 1 |  | 1.75 | - | - | 0.39 |
|  |  | Social Relationships and Norms | cardiovascular health-related risk factors | overall | Beta (SE) | 3 | | 1 |  | 1.11 | - | - | 0.20 |
|  |  | Social Relationships and Norms | cardiovascular health-related risk factors | overall | Beta (SE) | 4 | | 1 |  | 0.18 | - | - | 0.10 |
| [156] | Unger, 2014 | Crime and Safety | CVD risk scores | overall | OR (95%CI) | 1 | | 1 |  | 1.02 | 0.93 | 1.12 |  |
|  |  |  |  | overall | OR (95%CI) | 1 | | 2 |  | 1.05 | 0.94 | 1.17 |  |
|  |  | Social Cohesion and Social Capital | CVD risk scores | overall | OR (95%CI) | 2 | | 1 |  | 1.01 | 0.91 | 1.12 |  |
|  |  |  |  | overall | OR (95%CI) | 2 | | 2 |  | 0.99 | 0.87 | 1.12 |  |
|  |  | Economic and Social Disadvantage | CVD risk scores | overall | OR (95%CI) | 3 | | 1 |  | 1.08 | 0.99 | 1.17 |  |
|  |  |  |  | overall | OR (95%CI) | 3 | | 2 |  | 1.22 | 1.11 | 1.33 |  |
|  |  | Crime and Safety | CVD risk scores | female | OR (95%CI) | 4 | | 1 |  | 1.09 | 0.98 | 1.22 |  |
|  |  |  |  | female | OR (95%CI) | 4 | | 2 |  | 1.17 | 1.02 | 1.33 |  |
|  |  | Social Cohesion and Social Capital | CVD risk scores | female | OR (95%CI) | 5 | | 1 |  | 1.04 | 0.92 | 1.17 |  |
|  |  |  |  | female | OR (95%CI) | 5 | | 2 |  | 1.06 | 0.92 | 1.23 |  |
|  |  | Economic and Social Disadvantage | CVD risk scores | female | OR (95%CI) | 6 | | 1 |  | 1.19 | 1.07 | 1.31 |  |
|  |  |  |  | female | OR (95%CI) | 6 | | 2 |  | 1.41 | 1.26 | 1.59 |  |
|  |  | Crime and Safety | CVD risk scores | male | OR (95%CI) | 7 | | 1 |  | 0.96 | 0.86 | 1.08 |  |
|  |  |  |  | male | OR (95%CI) | 7 | | 2 |  | 0.93 | 0.82 | 1.07 |  |
|  |  | Social Cohesion and Social Capital | CVD risk scores | male | OR (95%CI) | 8 | | 1 |  | 0.90 | 0.78 | 1.05 |  |
|  |  |  |  | male | OR (95%CI) | 8 | | 2 |  | 0.98 | 0.87 | 1.11 |  |
|  |  | Economic and Social Disadvantage | CVD risk scores | male | OR (95%CI) | 9 | | 1 |  | 0.98 | 0.89 | 1.09 |  |
|  |  |  |  | male | OR (95%CI) | 9 | | 2 |  | 1.04 | 0.92 | 1.17 |  |
| [157] | Wagner, 2016 | Economic and Social Disadvantage | cardiovascular health-related risk factors | overall | Beta | 1 | | 1 | >= 12 (reference) | - | - | - |  |
|  |  |  |  | overall | Beta (95%CI) | 1 | | 2 |  | 2.74 | -0.29 | 5.76 |  |
|  |  |  |  | overall | Beta (95%CI) | 1 | | 3 | <=8 | 3.97 | 0.50 | 7.44 |  |
| [158] | Whittaker, 2012 | Social Relationships and Norms | CVD risk scores | female | OR (95%CI) | 1 | | 1 |  | 0.90 | 0.76 | 1.10 |  |
| [159] | Willets, 2019 | Social Cohesion and Social Capital | cardiovascular health-related risk factors | overall | Beta (95%CI) | 1 | | 1 |  | -0.09 | -0.87 | 0.70 |  |
|  |  | Crime and Safety | cardiovascular health-related risk factors | overall | Beta (95%CI) | 2 | | 1 |  | -0.35 | -1.23 | 0.54 |  |
|  |  | Crime and Safety | cardiovascular health-related risk factors | overall | Beta (95%CI) | 3 | | 1 |  | -0.96 | -1.97 | 0.06 |  |
|  |  | Crime and Safety | cardiovascular health-related risk factors | overall | Beta (95%CI) | 4 | | 1 |  | 6.12 | -0.01 | 12.24 |  |
|  |  | Social Cohesion and Social Capital | cardiovascular health-related risk factors | male | Beta (95%CI) | 5 | | 1 |  | -0.70 | -1.95 | 0.55 |  |
|  |  | Crime and Safety | cardiovascular health-related risk factors | male | Beta (95%CI) | 6 | | 1 |  | -1.57 | -2.97 | -0.16 |  |
|  |  | Crime and Safety | cardiovascular health-related risk factors | male | Beta (95%CI) | 7 | | 1 |  | -0.64 | -2.24 | 0.96 |  |
|  |  | Crime and Safety | cardiovascular health-related risk factors | male | Beta (95%CI) | 8 | | 1 |  | 3.34 | -6.47 | 13.15 |  |
|  |  | Social Cohesion and Social Capital | cardiovascular health-related risk factors | female | Beta (95%CI) | 9 | | 1 |  | 0.79 | -0.11 | 1.68 |  |
|  |  | Crime and Safety | cardiovascular health-related risk factors | female | Beta (95%CI) | 10 | | 1 |  | 0.14 | -0.88 | 1.15 |  |
|  |  | Crime and Safety | cardiovascular health-related risk factors | female | Beta (95%CI) | 11 | | 1 |  | -0.48 | -1.65 | 0.69 |  |
|  |  | Crime and Safety | cardiovascular health-related risk factors | female | Beta (95%CI) | 12 | | 1 |  | 4.08 | -2.93 | 11.09 |  |
| [160] | Williams, 2012 | Economic and Social Disadvantage | cardiovascular health-related risk factors | overall | Mean | 1 | | 1 | 1 least disadvantaged - reference | - | - | - | - |
|  |  |  |  | overall |  | 1 | | 2 | 2 | 125.00 | - | - | 0.49 |
|  |  |  |  | overall |  | 1 | | 3 | 3 | 128.00 | - | - | 0.51 |
|  |  |  |  | overall |  | 1 | | 4 | 4 | 126.00 | - | - | 0.50 |
|  |  | Economic and Social Disadvantage | cardiovascular health-related risk factors | overall | Mean | 2 | | 1 | 1 least disadvantaged - reference | - | - | - | - |
|  |  |  |  | overall |  | 2 | | 2 | 2 | 69.00 | - | - | 0.33 |
|  |  |  |  | overall |  | 2 | | 3 | 3 | 69.00 | - | - | 0.34 |
|  |  |  |  | overall |  | 2 | | 4 | 4 | 69.00 | - | - | 0.34 |
|  |  | Economic and Social Disadvantage | metabolic and inflammatory-related risk factors | overall | Mean | 3 | | 1 | 1 least disadvantaged - reference | - | - | - | - |
|  |  |  |  | overall |  | 3 | | 2 | 2 | 5.60 | - | - | 0.03 |
|  |  |  |  | overall |  | 3 | | 3 | 3 | 5.60 | - | - | 0.03 |
|  |  |  |  | overall |  | 3 | | 4 | 4 | 5.60 | - | - | 0.03 |
|  |  | Economic and Social Disadvantage | metabolic and inflammatory-related risk factors | overall | Mean | 4 | | 1 | 1 least disadvantaged - reference | - | - | - | - |
|  |  |  |  | overall |  | 4 | | 2 | 2 | 1.50 | - | - | 0.44 |
|  |  |  |  | overall |  | 4 | | 3 | 3 | 1.40 | - | - | 0.45 |
|  |  |  |  | overall |  | 4 | | 4 | 4 | 1.40 | - | - | 0.45 |
|  |  | Economic and Social Disadvantage | metabolic and inflammatory-related risk factors | overall | Mean | 5 | | 1 | 1 least disadvantaged - reference | - | - | - | - |
|  |  |  |  | overall |  | 5 | | 2 | 2 | 1.40 | - | - | 0.03 |
|  |  |  |  | overall |  | 5 | | 3 | 3 | 1.40 | - | - | 0.03 |
|  |  |  |  | overall |  | 5 | | 4 | 4 | 1.50 | - | - | 0.03 |
|  |  | Economic and Social Disadvantage | glucose metabolism-related risk factors | overall | Mean | 6 | | 1 | 1 least disadvantaged - reference | - | - | - | - |
|  |  |  |  | overall |  | 6 | | 2 | 2 | 5.30 | - | - | 0.01 |
|  |  |  |  | overall |  | 6 | | 3 | 3 | 5.30 | - | - | 0.01 |
|  |  |  |  | overall |  | 6 | | 4 | 4 | 5.30 | - | - | 0.01 |
|  |  | Economic and Social Disadvantage | glucose metabolism-related risk factors | overall | Mean | 7 | | 1 | 1 least disadvantaged - reference | - | - | - | - |
|  |  |  |  | overall |  | 7 | | 2 | 2 | 5.40 | - | - | 0.03 |
|  |  |  |  | overall |  | 7 | | 3 | 3 | 5.50 | - | - | 0.03 |
|  |  |  |  | overall |  | 7 | | 4 | 4 | 5.60 | - | - | 0.03 |
|  |  | Economic and Social Disadvantage | glucose metabolism-related risk factors | overall | OR (95%CI) | 8 | | 1 | 1 least disadvantaged - reference | 1.00 | - | - | - |
|  |  |  |  | overall |  | 8 | | 2 | 3 | 1.13 | 0.79 | 1.62 |  |
|  |  |  |  | overall |  | 8 | | 3 | 2 | 1.50 | 1.06 | 2.13 |  |
|  |  |  |  | overall |  | 8 | | 4 | 1 | 1.53 | 1.07 | 2.18 |  |
| [161] | Wing, 2016 | Crime and Safety and Social Cohesion and Social Capital (combined) | cardiovascular health-related risk factors | overall | Mean (SE) | 1 | | 1 | Q1 (lowest) | 140.40 | - | - | 7.90 |
|  |  |  |  | overall |  | 1 | | 2 | Q2 | 161.90 | - | - | 7.50 |
|  |  |  |  | overall |  | 1 | | 3 | Q3 | 158.70 | - | - | 7.60 |
|  |  |  |  | overall |  | 1 | | 4 | Q4 | 158.30 | - | - | 7.60 |
|  |  | Crime and Safety and Social Cohesion and Social Capital (combined) | cardiovascular health-related risk factors | overall | Mean (SE) | 2 | | 1 | Q1 (lowest) | 1.58 | - | - | 2.27 |
|  |  |  |  | overall |  | 2 | | 2 | Q2 | -0.14 | - | - | 2.15 |
|  |  |  |  | overall |  | 2 | | 3 | Q3 | 0.41 | - | - | 2.23 |
|  |  |  |  | overall |  | 2 | | 4 | Q4 | 3.03 | - | - | 2.19 |
|  |  | Crime and Safety and Social Cohesion and Social Capital (combined) | cardiovascular health-related risk factors | overall | Percent of participants with outcome per exposure quartile | 3 | | 1 | Q1 (lowest) | 44.20 | - | - |  |
|  |  |  |  | overall |  | 3 | | 2 | Q2 | 49.20 | - | - |  |
|  |  |  |  | overall |  | 3 | | 3 | Q3 | 50.00 | - | - |  |
|  |  |  |  | overall |  | 3 | | 4 | Q4 | 50.70 | - | - |  |
|  |  | Crime and Safety and Social Cohesion and Social Capital (combined) | cardiovascular health-related risk factors | overall | Mean differences (95%CI) | 4 | | 1 |  | 7.86 | −0.74 | 16.45 |  |
| [162] | Yang, 2014 | Social Relationships and Norms | CVD risk scores | overall | OR (95%CI) | 1 | | 1 |  | 1.16 | 0.85 | 1.57 |  |
|  |  | Social Relationships and Norms | CVD risk scores | overall | OR (95%CI) | 2 | | 1 |  | 1.15 | 0.88 | 1.50 |  |
|  |  | Social Relationships and Norms | CVD risk scores | overall | OR (95%CI) | 3 | | 1 |  | 0.89 | 0.64 | 1.23 |  |
|  |  | Social Relationships and Norms | CVD risk scores | overall | OR (95%CI) | 4 | | 1 |  | 1.09 | 0.77 | 1.56 |  |
|  |  | Social Relationships and Norms | CVD risk scores | overall | OR (95%CI) | 5 | | 1 |  | 1.44 | 1.05 | 1.99 |  |
|  |  | Social Relationships and Norms | CVD risk scores | overall | OR (95%CI) | 6 | | 1 |  | 1.32 | 0.91 | 1.93 |  |
|  |  | Social Relationships and Norms | CVD risk scores | overall | OR (95%CI) | 7 | | 1 |  | 1.14 | 0.86 | 1.49 |  |
|  |  | Social Relationships and Norms | CVD risk scores | overall | OR (95%CI) | 8 | | 1 |  | 1.52 | 0.99 | 2.30 |  |
| [163] | Yang, 2016 | Social Relationships and Norms | metabolic and inflammatory-related risk factors | overall | OR (95%CI) | 1 | | 1 |  | 0.99 | 0.76 | 1.29 |  |
|  |  | Social Relationships and Norms | metabolic and inflammatory-related risk factors | overall | OR (95%CI) | 2 | | 1 |  | 0.79 | 0.62 | 1.00 |  |
|  |  | Social Relationships and Norms | metabolic and inflammatory-related risk factors | overall | OR (95%CI) | 3 | | 1 |  | 1.07 | 0.55 | 2.10 |  |
|  |  | Social Relationships and Norms | metabolic and inflammatory-related risk factors | overall | OR (95%CI) | 4 | | 1 |  | 0.89 | 0.81 | 0.98 |  |
|  |  | Social Relationships and Norms | metabolic and inflammatory-related risk factors | overall | OR (95%CI) | 5 | | 1 |  | 0.77 | 0.48 | 1.23 |  |
| [164] | Yang, 2015 | Social Relationships and Norms | cardiovascular health-related risk factors | overall | Beta (SE) | 1 | | 1 | low | −0.01 | - | - | 0.01 |
|  |  |  |  | overall |  | 1 | | 2 | moderate | 0.01 | - | - | 0.01 |
|  |  |  |  | overall |  | 1 | | 3 | High (reference) | - | - | - | - |
|  |  | Social Relationships and Norms | cardiovascular health-related risk factors | overall | Beta (SE) | 2 | | 1 | low | 0.03 | - | - | 0.02 |
|  |  |  |  | overall |  | 2 | | 2 | moderate | 0.02 | - | - | 0.01 |
|  |  |  |  | overall |  | 2 | | 3 | High (reference) | - | - | - | - |
| [165] | Yang, 2013 | Social Relationships and Norms | CVD risk scores | overall | OR (95%CI) | 1 | | 1 |  | 0.63 | 0.46 | 0.86 |  |
|  |  | Social Relationships and Norms | CVD risk scores | male | OR (95%CI) | 2 | | 1 |  | 0.58 | 0.33 | 1.02 |  |
|  |  | Social Relationships and Norms | CVD risk scores | female | OR (95%CI) | 3 | | 1 |  | 0.64 | 0.44 | 0.96 |  |
| [166] | Yao, 2019 | Economic and Social Disadvantage | cardiovascular health-related risk factors | overall | regression coefficient | 1 | | 1 | income polarization | 40.70 | - | - | 10.10 |
|  |  | Economic and Social Disadvantage | cardiovascular health-related risk factors | overall | regression coefficient | 2 | | 1 | income inequality | -6.84 | - | - | 2.10 |
| [167] | Zöller, 2012 | Economic and Social Disadvantage | cardiovascular health-related risk factors | male | OR (95%CI) | 1 | | 1 | low - reference | 1.00 | - | - | - |
|  |  |  |  | male |  | 1 | | 2 | moderate | 1.02 | 1.00 | 1.05 |  |
|  |  |  |  | male |  | 1 | | 3 | high | 1.03 | 1.00 | 1.06 |  |
|  |  | Economic and Social Disadvantage | cardiovascular health-related risk factors | female | OR (95%CI) | 2 | | 1 | low - reference | 1.00 | - | - | - |
|  |  |  |  | female |  | 2 | | 2 | moderate | 1.05 | 1.02 | 1.09 |  |
|  |  |  |  | female |  | 2 | | 3 | high | 1.12 | 1.08 | 1.16 |  |
| [168] | Zanelatto, 2019 | Crime and Safety | cardiovascular health-related risk factors | overall | Beta | 1 | | 1 | Lower tertile - reference | - | - | - | - |
|  |  |  |  | overall |  | 1 | | 2 | Intermediate tertile | 0.50 | -1.47 | 2.48 |  |
|  |  |  |  | overall |  | 1 | | 3 | Upper tertile | 0.49 | -1.96 | 2.93 |  |
|  |  | Crime and Safety | cardiovascular health-related risk factors | overall | Beta | 2 | | 1 | Lower tertile - reference | - | - | - | - |
|  |  |  |  | overall |  | 2 | | 2 | Intermediate tertile | -0.24 | -1.71 | 1.23 |  |
|  |  |  |  | overall |  | 2 | | 3 | Upper tertile | 0.81 | -0.86 | 2.48 |  |

1. Adams, R.J., et al., *Effects of area deprivation on health risks and outcomes: a multilevel, cross-sectional, Australian population study.* Int J Public Health, 2009. **54**(3): p. 183-92.

2. Adjaye-Gbewonyo, K., et al., *Income inequality and cardiovascular disease risk factors in a highly unequal country: a fixed-effects analysis from South Africa.* Int J Equity Health, 2018. **17**(1): p. 31.

3. Agyemang, C., et al., *Ethnic differences in the effect of environmental stressors on blood pressure and hypertension in the Netherlands.* BMC Public Health, 2007. **7**: p. 118.

4. Aliarzadeh, B., et al., *Association between socio-economic status and hemoglobin A1c levels in a Canadian primary care adult population without diabetes.* BMC Fam Pract, 2014. **15**: p. 7.

5. Altevers, J., et al., *Poor structural social support is associated with an increased risk of Type 2 diabetes mellitus: findings from the MONICA/KORA Augsburg cohort study.* Diabet Med, 2016. **33**(1): p. 47-54.

6. Andell, P., et al., *Neighborhood socioeconomic status and aortic stenosis: A Swedish study based on nationwide registries and an echocardiographic screening cohort.* Int J Cardiol, 2020. **318**: p. 153-159.

7. Andersen, A.F., et al., *Life-course socio-economic position, area deprivation and Type 2 diabetes: findings from the British Women's Heart and Health Study.* Diabet Med, 2008. **25**(12): p. 1462-8.

8. Auchincloss, A.H., et al., *Association of insulin resistance with distance to wealthy areas: the multi-ethnic study of atherosclerosis.* Am J Epidemiol, 2007. **165**(4): p. 389-97.

9. Bagheri, N., et al., *Community cardiovascular disease risk from cross-sectional general practice clinical data: a spatial analysis.* Prev Chronic Dis, 2015. **12**: p. E26.

10. Bagheri, N., et al., *Identifying hotspots of type 2 diabetes risk using general practice data and geospatial analysis: an approach to inform policy and practice.* Aust J Prim Health, 2019.

11. Bajaj, A., et al., *Daily social interactions, close relationships, and systemic inflammation in two samples: Healthy middle-aged and older adults.* Brain Behav Immun, 2016. **58**: p. 152-164.

12. Baldock, K.L., et al., *Gender-specific associations between perceived and objective neighbourhood crime and metabolic syndrome.* PLoS One, 2018. **13**(7): p. e0201336.

13. Baldock, K., et al., *Associations between resident perceptions of the local residential environment and metabolic syndrome.* J Environ Public Health, 2012. **2012**: p. 589409.

14. Barber, S., et al., *Neighborhood Disadvantage and Cumulative Biological Risk Among a Socioeconomically Diverse Sample of African American Adults: An Examination in the Jackson Heart Study.* Journal of Racial and Ethnic Health Disparities, 2016. **3**(3): p. 444-456.

15. Barber, S., et al., *Double-jeopardy: The joint impact of neighborhood disadvantage and low social cohesion on cumulative risk of disease among African American men and women in the Jackson Heart Study.* Soc Sci Med, 2016. **153**: p. 107-15.

16. Barber, S., et al., *Neighborhood Disadvantage, Poor Social Conditions, and Cardiovascular Disease Incidence Among African American Adults in the Jackson Heart Study.* Am J Public Health, 2016. **106**(12): p. 2219-2226.

17. Bhopal, R., et al., *Ethnic and socio-economic inequalities in coronary heart disease, diabetes and risk factors in Europeans and South Asians.* J Public Health Med, 2002. **24**(2): p. 95-105.

18. Bird, C.E., et al., *Neighbourhood socioeconomic status and biological 'wear and tear' in a nationally representative sample of US adults.* Journal of Epidemiology and Community Health, 2010. **64**(10): p. 860-865.

19. Bland, S.H., et al., *Long term relations between earthquake experiences and coronary heart disease risk factors.* Am J Epidemiol, 2000. **151**(11): p. 1086-90.

20. Bland, S.H., et al., *Social network and blood pressure: a population study.* Psychosom Med, 1991. **53**(6): p. 598-607.

21. Boylan, J.M. and S.A. Robert, *Neighborhood SES is particularly important to the cardiovascular health of low SES individuals.* Soc Sci Med, 2017. **188**: p. 60-68.

22. Breckenkamp, J., A. Mielck, and O. Razum, *Health inequalities in Germany: do regional-level variables explain differentials in cardiovascular risk?* BMC Public Health, 2007. **7**: p. 132.

23. Browning, C.R., K.A. Cagney, and J. Iveniuk, *Neighborhood stressors and cardiovascular health: crime and C-reactive protein in Dallas, USA.* Soc Sci Med, 2012. **75**(7): p. 1271-9.

24. Bu, F., A. Steptoe, and D. Fancourt, *Relationship between loneliness, social isolation and modifiable risk factors for cardiovascular disease: a latent class analysis.* J Epidemiol Community Health, 2021.

25. Carels, R.A., J.A. Blumenthal, and A. Sherwood, *Effect of satisfaction with social support on blood pressure in normotensive and borderline hypertensive men and women.* Int J Behav Med, 1998. **5**(1): p. 76-85.

26. Carson, A.P., et al., *Cumulative socioeconomic status across the life course and subclinical atherosclerosis.* Ann Epidemiol, 2007. **17**(4): p. 296-303.

27. Caspi, A., et al., *Socially isolated children 20 years later: risk of cardiovascular disease.* Arch Pediatr Adolesc Med, 2006. **160**(8): p. 805-11.

28. Cathorall, M.L., et al., *Neighborhood Disadvantage and Variations in Blood Pressure.* Am. J. Health Educ., 2015. **46**(5): p. 266-273.

29. Chaparro, M.P., et al., *Neighborhood deprivation and biomarkers of health in Britain: the mediating role of the physical environment.* BMC Public Health, 2018. **18**(1): p. 801.

30. Chaix, B., et al., *Individual/neighborhood social factors and blood pressure in the RECORD Cohort Study: which risk factors explain the associations?* Hypertension, 2010. **55**(3): p. 769-75.

31. Chaix, B., et al., *Residential environment and blood pressure in the PRIME Study: is the association mediated by body mass index and waist circumference?* J Hypertens, 2008. **26**(6): p. 1078-84.

32. Chichlowska, K.L., et al., *Individual and neighborhood socioeconomic status characteristics and prevalence of metabolic syndrome: the Atherosclerosis Risk in Communities (ARIC) Study.* Psychosom Med, 2008. **70**(9): p. 986-92.

33. Clark, C.R., et al., *Neighborhood disadvantage, neighborhood safety and cardiometabolic risk factors in African Americans: biosocial associations in the Jackson Heart study.* PLoS One, 2013. **8**(5): p. e63254.

34. Clark, C.R., et al., *Cardiovascular inflammation in healthy women: multilevel associations with state-level prosperity, productivity and income inequality.* BMC Public Health, 2012. **12**: p. 211.

35. Claudel, S.E., et al., *Association between neighborhood-level socioeconomic deprivation and incident hypertension: A longitudinal analysis of data from the Dallas heart study.* Am Heart J, 2018. **204**: p. 109-118.

36. Climie, R.E., et al., *Individual and Neighborhood Deprivation and Carotid Stiffness.* Hypertension, 2019. **73**(6): p. 1185-1194.

37. Cohn, T., et al., *Impact of Individual and Neighborhood Factors on Cardiovascular Risk in White Hispanic and Non-Hispanic Women and Men.* Res Nurs Health, 2017. **40**(2): p. 120-131.

38. Coulon, S.M., et al., *Multilevel Associations of Neighborhood Poverty, Crime, and Satisfaction With Blood Pressure in African-American Adults.* Am J Hypertens, 2016. **29**(1): p. 90-5.

39. Coulon, S.M., et al., *The Association of Neighborhood Gene-Environment Susceptibility with Cortisol and Blood Pressure in African-American Adults.* Ann Behav Med, 2016. **50**(1): p. 98-107.

40. Cozier, Y.C., et al., *Neighborhood Socioeconomic Status in Relation to Serum Biomarkers in the Black Women's Health Study.* J Urban Health, 2016. **93**(2): p. 279-91.

41. Creaven, A.M., S. Howard, and B.M. Hughes, *Social support and trait personality are independently associated with resting cardiovascular function in women.* Br J Health Psychol, 2013. **18**(3): p. 556-73.

42. Cross, R., et al., *Cross-sectional study of area-level disadvantage and glycaemic-related risk in community health service users in the Southern.IML Research (SIMLR) cohort.* Aust Health Rev, 2019. **43**(1): p. 85-91.

43. Cubbin, C. and M.A. Winkleby, *Protective and harmful effects of neighborhood-level deprivation on individual-level health knowledge, behavior changes, and risk of coronary heart disease.* Am J Epidemiol, 2005. **162**(6): p. 559-68.

44. De Moraes, A.C.F., et al., *Sex and ethnicity modify the associations between individual and contextual socioeconomic indicators and ideal cardiovascular health: MESA study.* J Public Health (Oxf), 2019. **41**(3): p. e237-e244.

45. Deans, K.A., et al., *Differences in atherosclerosis according to area level socioeconomic deprivation: cross sectional, population based study.* Bmj, 2009. **339**: p. b4170.

46. Diez Roux, A.V., et al., *Socioeconomic disadvantage and change in blood pressure associated with aging.* Circulation, 2002. **106**(6): p. 703-10.

47. Diez Roux, A.V., D.R. Jacobs, and C.I. Kiefe, *Neighborhood characteristics and components of the insulin resistance syndrome in young adults: the coronary artery risk development in young adults (CARDIA) study.* Diabetes Care, 2002. **25**(11): p. 1976-82.

48. Diez-Roux, A.V., et al., *Neighborhood environments and coronary heart disease: a multilevel analysis.* Am J Epidemiol, 1997. **146**(1): p. 48-63.

49. Djekic, D., et al., *Impact of socioeconomic status on coronary artery calcification.* Eur J Prev Cardiol, 2018. **25**(16): p. 1756-1764.

50. Do, D.P., et al., *Circadian rhythm of cortisol and neighborhood characteristics in a population-based sample: the Multi-Ethnic Study of Atherosclerosis.* Health Place, 2011. **17**(2): p. 625-32.

51. Dragano, N., et al., *Subclinical coronary atherosclerosis and neighbourhood deprivation in an urban region.* Eur J Epidemiol, 2009. **24**(1): p. 25-35.

52. Dubowitz, T., et al., *The Women's Health Initiative: The food environment, neighborhood socioeconomic status, BMI, and blood pressure.* Obesity (Silver Spring), 2012. **20**(4): p. 862-71.

53. Duncan, D.T., et al., *Perceived spatial stigma, body mass index and blood pressure: a global positioning system study among low-income housing residents in New York City.* Geospat Health, 2016. **11**(2): p. 399.

54. Dwane, N., N. Wabiri, and S. Manda, *Small-area variation  of cardiovascular diseases and select risk factors and their association to household and area poverty in South Africa: Capturing emerging trends in South Africa to better target local level interventions.* PLoS One, 2020. **15**(4): p. e0230564.

55. Eichinger, M., et al., *How are physical activity behaviors and cardiovascular risk factors associated with characteristics of the built and social residential environment?* PLoS One, 2015. **10**(6): p. e0126010.

56. Ellaway, A. and S. Macintyre, *Is social participation associated with cardiovascular disease risk factors?* Soc Sci Med, 2007. **64**(7): p. 1384-91.

57. Engström, G., et al., *Geographic distribution of stroke incidence within an urban population: Relations to socioeconomic circumstances and prevalence of cardiovascular risk factors.* Stroke, 2001. **32**(5): p. 1098-1103.

58. Ferguson, T.S., et al., *Neighbourhood socioeconomic characteristics and blood pressure among Jamaican youth: a pooled analysis of data from observational studies.* PeerJ, 2020. **8**: p. e10058.

59. Finch, B.K., et al., *Neighborhood effects on health: Concentrated advantage and disadvantage.* Health & Place, 2010. **16**(5): p. 1058-1060.

60. Foraker, R.E., et al., *Distribution of Cardiovascular Health by Individual- and Neighborhood-Level Socioeconomic Status: Findings From the Jackson Heart Study.* Glob Heart, 2019. **14**(3): p. 241-250.

61. Ford, E.S., E.B. Loucks, and L.F. Berkman, *Social integration and concentrations of C-reactive protein among US adults.* Ann Epidemiol, 2006. **16**(2): p. 78-84.

62. Ford, J., et al., *Social Integration and Quality of Social Relationships as Protective Factors for Inflammation in a Nationally Representative Sample of Black Women.* J Urban Health, 2019. **96**(Suppl 1): p. 35-43.

63. Fuller, K.C., et al., *ACE gene haplotypes and social networks: Using a biocultural framework to investigate blood pressure variation in African Americans.* Plos One, 2018. **13**(9).

64. Gallo, L.C., et al., *Individual and neighborhood socioeconomic status and inflammation in Mexican American women: what is the role of obesity?* Psychosom Med, 2012. **74**(5): p. 535-42.

65. Garcia, L., et al., *Influence of neighbourhood socioeconomic position on the transition to type II diabetes in older Mexican Americans: the Sacramento Area Longitudinal Study on Aging.* BMJ Open, 2016. **6**(8): p. e010905.

66. Garcia, L., et al., *The Impact of Neighborhood Socioeconomic Position on Prevalence of Diabetes and Prediabetes in Older Latinos: The Sacramento Area Latino Study on Aging.* Hisp Health Care Int, 2015. **13**(2): p. 77-85.

67. Gary-Webb, T.L., et al., *Changes in perceptions of neighborhood environment and Cardiometabolic outcomes in two predominantly African American neighborhoods.* BMC Public Health, 2020. **20**(1): p. 52.

68. Gebreab, S.Y., et al., *Geographic variations in cardiovascular health in the United States: contributions of state- and individual-level factors.* J Am Heart Assoc, 2015. **4**(6): p. e001673.

69. Grimaud, O., et al., *Gender differences in the association between socioeconomic status and subclinical atherosclerosis.* PLoS One, 2013. **8**(11): p. e80195.

70. Höfelmann, D.A., et al., *Is income area level associated with blood pressure in adults regardless of individual-level characteristics? A multilevel approach.* Health Place, 2012. **18**(5): p. 971-7.

71. Halonen, J.I., et al., *Childhood Psychosocial Adversity and Adult Neighborhood Disadvantage as Predictors of Cardiovascular Disease: A Cohort Study.* Circulation, 2015. **132**(5): p. 371-9.

72. Hamad, R., et al., *Association of Neighborhood Disadvantage With Cardiovascular Risk Factors and Events Among Refugees in Denmark.* JAMA Netw Open, 2020. **3**(8): p. e2014196.

73. Hanson, B.S., et al., *Social anchorage and blood pressure in elderly men--a population study.* J Hypertens, 1988. **6**(6): p. 503-10.

74. Helminen, A., et al., *Validity assessment of a social support index.* Scand J Soc Med, 1995. **23**(1): p. 66-74.

75. Helminen, A., et al., *Carotid atherosclerosis in middle-aged men. Relation to conjugal circumstances and social support.* Scand J Soc Med, 1995. **23**(3): p. 167-72.

76. Helminen, A., et al., *Social network in relation to plasma fibrinogen.* J Biosoc Sci, 1997. **29**(2): p. 129-39.

77. Henning, C.H., et al., *Identification of direct and indirect social network effects in the pathophysiology of insulin resistance in obese human subjects.* PLoS One, 2014. **9**(4): p. e93860.

78. Hickson, D.A., et al., *Socioeconomic position is positively associated with blood pressure dipping among African-American adults: the Jackson Heart Study.* Am J Hypertens, 2011. **24**(9): p. 1015-21.

79. Hilding, A., C. Shen, and C.G. Östenson, *Social network and development of prediabetes and type 2 diabetes in middle-aged Swedish women and men.* Diabetes Res Clin Pract, 2015. **107**(1): p. 166-77.

80. Holmes, L.M. and E.A. Marcelli, *Neighborhoods and systemic inflammation: High CRP among legal and unauthorized Brazilian migrants.* Health Place, 2012. **18**(3): p. 683-693.

81. Horsten, M., et al., *Social relations and the metabolic syndrome in middle-aged Swedish women.* J Cardiovasc Risk, 1999. **6**(6): p. 391-7.

82. Hosseini, Z., et al., *Social connections and hypertension in women and men: a population-based cross-sectional study of the Canadian Longitudinal Study on Aging.* J Hypertens, 2020.

83. Hughes, B.M., *Social support in ordinary life and laboratory measures of cardiovascular reactivity: gender differences in habituation-sensitization.* Ann Behav Med, 2007. **34**(2): p. 166-76.

84. Islam, S.J., et al., *Neighborhood Characteristics and Ideal Cardiovascular Health Among Black Adults: Results From the Morehouse-Emory Cardiovascular (MECA) Center for Health Equity.* Ann Epidemiol, 2020.

85. Jimenez, M.P., et al., *Longitudinal associations of neighborhood socioeconomic status with cardiovascular risk factors: A 46-year follow-up study.* Soc Sci Med, 2019. **241**: p. 112574.

86. Kakinami, L., et al., *Neighbourhood disadvantage and behavioural problems during childhood and the risk of cardiovascular disease risk factors and events from a prospective cohort.* Prev Med Rep, 2017. **8**: p. 294-300.

87. Keita, A.D., et al., *Associations of neighborhood area level deprivation with the metabolic syndrome and inflammation among middle- and older- age adults.* BMC Public Health, 2014. **14**: p. 1319.

88. Kelli, H.M., et al., *Association Between Living in Food Deserts and Cardiovascular Risk.* Circ Cardiovasc Qual Outcomes, 2017. **10**(9).

89. Kent de Grey, R.G., et al., *Enemies and friends in high-tech places: the development and validation of the Online Social Experiences Measure.* Digit Health, 2019. **5**: p. 2055207619878351.

90. Kershaw, K.N., et al., *Association of Changes in Neighborhood-Level Racial Residential Segregation With Changes in Blood Pressure Among Black Adults: The CARDIA Study.* JAMA Intern Med, 2017. **177**(7): p. 996-1002.

91. Kim, D., et al., *Do neighborhood socioeconomic deprivation and low social cohesion predict coronary calcification?: the CARDIA study.* Am J Epidemiol, 2010. **172**(3): p. 288-98.

92. Kim, K., et al., *Associations between social network properties and metabolic syndrome and the mediating effect of physical activity: findings from the Cardiovascular and Metabolic Diseases Etiology Research Center (CMERC) Cohort.* BMJ Open Diabetes Res Care, 2020. **8**(1).

93. King, K.E., J.D. Morenoff, and J.S. House, *Neighborhood context and social disparities in cumulative biological risk factors.* Psychosom Med, 2011. **73**(7): p. 572-9.

94. Kivimäki, M., et al., *Neighbourhood socioeconomic disadvantage, risk factors, and diabetes from childhood to middle age in the Young Finns Study: a cohort study.* Lancet Public Health, 2018. **3**(8): p. e365-e373.

95. Lawlor, D.A., et al., *Life-course socioeconomic position, area deprivation, and coronary heart disease: findings from the British Women's Heart and Health Study.* Am J Public Health, 2005. **95**(1): p. 91-7.

96. Lee, J.E. and K.E. Cichy, *Complex Role of Touch in Social Relationships for Older Adults' Cardiovascular Disease Risk.* Res Aging, 2020. **42**(7-8): p. 208-216.

97. Lei, M.K., S.R.H. Beach, and R.L. Simons, *Biological embedding of neighborhood disadvantage and collective efficacy: Influences on chronic illness via accelerated cardiometabolic age.* Dev Psychopathol, 2018. **30**(5): p. 1797-1815.

98. Lemelin, E.T., et al., *Life-course socioeconomic positions and subclinical atherosclerosis in the multi-ethnic study of atherosclerosis.* Soc Sci Med, 2009. **68**(3): p. 444-51.

99. Lewis, T.T., et al., *Race, psychosocial factors, and aortic pulse wave velocity: the Health, Aging, and Body Composition Study.* J Gerontol A Biol Sci Med Sci, 2010. **65**(10): p. 1079-85.

100. Li, K., M. Wen, and J.X. Fan, *Neighborhood Racial Diversity and Metabolic Syndrome: 2003-2008 National Health and Nutrition Examination Survey.* J Immigr Minor Health, 2019. **21**(1): p. 151-160.

101. Li, K., M. Wen, and K.A. Henry, *Ethnic density, immigrant enclaves, and Latino health risks: A propensity score matching approach.* Soc Sci Med, 2017. **189**: p. 44-52.

102. Linden, W., et al., *Sex differences in social support, self-deception, hostility, and ambulatory cardiovascular activity.* Health Psychol, 1993. **12**(5): p. 376-80.

103. Lippert, A.M., et al., *Associations of Continuity and Change in Early Neighborhood Poverty With Adult Cardiometabolic Biomarkers in the United States: Results From the National Longitudinal Study of Adolescent to Adult Health, 1995-2008.* Am J Epidemiol, 2017. **185**(9): p. 765-776.

104. Loose, F., et al., *Blood pressure and psychological distress among North Africans in France: The role of perceived personal/group discrimination and gender.* Am J Hum Biol, 2017. **29**(5).

105. Loucks, E.B., et al., *Relation of social integration to inflammatory marker concentrations in men and women 70 to 79 years.* Am J Cardiol, 2006. **97**(7): p. 1010-6.

106. Loucks, E.B., et al., *Social integration is associated with fibrinogen concentration in elderly men.* Psychosom Med, 2005. **67**(3): p. 353-8.

107. Loucks, E.B., et al., *Social networks and inflammatory markers in the Framingham Heart Study.* J Biosoc Sci, 2006. **38**(6): p. 835-42.

108. Maki, K.G., *Social support, strain, and glycemic control: A path analysis.* Pers. Relat., 2020. **27**(3): p. 592-612.

109. Marley, T.L. and M.W. Metzger, *A longitudinal study of structural risk factors for obesity and diabetes among American Indian young adults, 1994-2008.* Prev Chronic Dis, 2015. **12**: p. E69.

110. Martin, C.L., et al., *Neighborhood disadvantage across the transition from adolescence to adulthood and risk of metabolic syndrome.* Health Place, 2019. **57**: p. 131-138.

111. Matricciani, L.A., et al., *Investigating individual- and area-level socioeconomic gradients of pulse pressure among normotensive and hypertensive participants.* Int J Environ Res Public Health, 2013. **10**(2): p. 571-89.

112. Mayne, S.L., et al., *Neighbourhood racial/ethnic residential segregation and cardiometabolic risk: the multiethnic study of atherosclerosis.* J Epidemiol Community Health, 2019. **73**(1): p. 26-33.

113. Mayne, S.L., et al., *Longitudinal Associations of Neighborhood Crime and Perceived Safety With Blood Pressure: The Multi-Ethnic Study of Atherosclerosis (MESA).* Am J Hypertens, 2018. **31**(9): p. 1024-1032.

114. McKenzie, J.A., et al., *Ideal cardiovascular health in urban Jamaica: prevalence estimates and relationship to community property value, household assets and educational attainment: a cross-sectional study.* BMJ Open, 2020. **10**(12): p. e040664.

115. Mellman, T.A., et al., *Blood Pressure Dipping and Urban Stressors in Young Adult African Americans.* Ann Behav Med, 2015. **49**(4): p. 622-7.

116. Merkin, S.S., et al., *Neighborhoods and cumulative biological risk profiles by race/ethnicity in a national sample of U.S. adults: NHANES III.* Ann Epidemiol, 2009. **19**(3): p. 194-201.

117. Merkin, S.S., et al., *Race/ethnicity, neighborhood socioeconomic status and cardio-metabolic risk.* SSM Popul Health, 2020. **11**: p. 100634.

118. Merlo, J., et al., *Diastolic blood pressure and area of residence: multilevel versus ecological analysis of social inequity.* J Epidemiol Community Health, 2001. **55**(11): p. 791-8.

119. Metcalf, P.A., et al., *Comparison of different markers of socioeconomic status with cardiovascular disease and diabetes risk factors in the Diabetes, Heart and Health Survey.* N Z Med J, 2008. **121**(1269): p. 45-56.

120. Meza, B.P.L., et al., *Social network factors and cardiovascular health among baltimore public housing residents.* Prev Med Rep, 2020. **20**: p. 101192.

121. Mobley, L.R., et al., *Environment, obesity, and cardiovascular disease risk in low-income women.* Am J Prev Med, 2006. **30**(4): p. 327-332.

122. Murakami, K., et al., *Neighborhood socioeconomic status in relation to dietary intake and insulin resistance syndrome in female Japanese dietetic students.* Nutrition, 2010. **26**(5): p. 508-14.

123. Murray, E.T., et al., *Trajectories of neighborhood poverty and associations with subclinical atherosclerosis and associated risk factors: the multi-ethnic study of atherosclerosis.* Am J Epidemiol, 2010. **171**(10): p. 1099-108.

124. Naimi, A.I., et al., *Associations between area-level unemployment, body mass index, and risk factors for cardiovascular disease in an urban area.* Int J Environ Res Public Health, 2009. **6**(12): p. 3082-96.

125. Nazmi, A., et al., *Cross-sectional and longitudinal associations of neighborhood characteristics with inflammatory markers: findings from the multi-ethnic study of atherosclerosis.* Health Place, 2010. **16**(6): p. 1104-12.

126. Neergheen, V.L., et al., *Neighborhood social cohesion is associated with lower levels of interleukin-6 in African American women.* Brain Behav Immun, 2019. **76**: p. 28-36.

127. Ngo, A.D., et al., *Area-level socioeconomic characteristics and incidence of metabolic syndrome: a prospective cohort study.* BMC Public Health, 2013. **13**: p. 681.

128. Ngo, A.D., et al., *Area-level socioeconomic characteristics, prevalence and trajectories of cardiometabolic risk.* Int J Environ Res Public Health, 2014. **11**(1): p. 830-48.

129. Nikulina, V. and C.S. Widom, *Do race, neglect, and childhood poverty predict physical health in adulthood? A multilevel prospective analysis.* Child Abuse Negl, 2014. **38**(3): p. 414-24.

130. Nordstrom, C.K., et al., *The association of personal and neighborhood socioeconomic indicators with subclinical cardiovascular disease in an elderly cohort. The cardiovascular health study.* Soc Sci Med, 2004. **59**(10): p. 2139-47.

131. Ribeiro, A.I., et al., *Neighbourhood socioeconomic deprivation and allostatic load: a multi-cohort study.* Sci Rep, 2019. **9**(1): p. 8790.

132. Pedersen, J.M., et al., *Psychosocial risk factors for the metabolic syndrome: A prospective cohort study.* Int J Cardiol, 2016. **215**: p. 41-6.

133. Petersen, K.L., et al., *Community socioeconomic status is associated with circulating interleukin-6 and C-reactive protein.* Psychosomatic Medicine, 2008. **70**(6): p. 646-652.

134. Piferi, R.L. and K.A. Lawler, *Social support and ambulatory blood pressure: an examination of both receiving and giving.* Int J Psychophysiol, 2006. **62**(2): p. 328-36.

135. Pollack, C.E., et al., *Neighborhood socioeconomic status and coronary heart disease risk prediction in a nationally representative sample.* Public Health, 2012. **126**(10): p. 827-35.

136. Pollard, T.M., et al., *Social networks and coronary heart disease risk factors in South Asians and Europeans in the UK.* Ethn Health, 2003. **8**(3): p. 263-75.

137. Pollitt, R.A., et al., *Cumulative life course and adult socioeconomic status and markers of inflammation in adulthood.* J Epidemiol Community Health, 2008. **62**(6): p. 484-91.

138. Pollitt, R.A., et al., *Early-life and adult socioeconomic status and inflammatory risk markers in adulthood.* Eur J Epidemiol, 2007. **22**(1): p. 55-66.

139. Riva, M., C.V. Larsen, and P. Bjerregaard, *Association between individual-level and community-level socio-economic status and blood pressure among Inuit in Greenland.* Int J Circumpolar Health, 2016. **75**: p. 32757.

140. Robinette, J.W., et al., *Neighborhood features and physiological risk: An examination of allostatic load.* Health & Place, 2016. **41**: p. 110-118.

141. Robinette, J.W., J.D. Boardman, and E. Crimmins, *Perceived neighborhood social cohesion and cardiometabolic risk: a gene × environment study.* Biodemography Soc Biol, 2020. **65**(1): p. 1-15.

142. Rosvall, M., et al., *Area social characteristics and carotid atherosclerosis.* Eur. J. Public Health, 2007. **17**(4): p. 333-339.

143. Sörman, D.E., P. Hansson, and M. Rönnlund, *Blood pressure levels and longitudinal changes in relation to social network factors.* Psihologijske Teme, 2016. **25**(1): p. 59-73.

144. Samuel, L.J., et al., *Community Characteristics are Associated with Blood Pressure Levels in a Racially Integrated Community.* J Urban Health, 2015. **92**(3): p. 403-14.

145. Schulz, A.J., et al., *Do observed or perceived characteristics of the neighborhood environment mediate associations between neighborhood poverty and cumulative biological risk?* Health & Place, 2013. **24**: p. 147-156.

146. Seeman, T.E., et al., *Social relationships and their biological correlates: Coronary Artery Risk Development in Young Adults (CARDIA) study.* Psychoneuroendocrinology, 2014. **43**: p. 126-38.

147. Smith, G.D., et al., *Individual social class, area-based deprivation, cardiovascular disease risk factors, and mortality: the Renfrew and Paisley Study.* J Epidemiol Community Health, 1998. **52**(6): p. 399-405.

148. Sprung, M.R., et al., *Neighborhood crime is differentially associated with cardiovascular risk factors as a function of race and sex.* J Public Health Res, 2019. **8**(3): p. 1643.

149. Steppuhn, H., et al., *Individual and area-level determinants associated with C-reactive protein as a marker of cardiometabolic risk among adults: Results from the German National Health Interview and Examination Survey 2008-2011.* PLoS One, 2019. **14**(2): p. e0211774.

150. Strogatz, D.S., et al., *Social support, stress, and blood pressure in black adults.* Epidemiology, 1997. **8**(5): p. 482-7.

151. Theorell, T., et al., *Blood pressure variations across areas in the greater Stockholm region: analysis of 74,000 18-year-old men.* Soc Sci Med, 1982. **16**(4): p. 469-73.

152. Toms, R., et al., *Geographic variation in cardiometabolic risk factor prevalence explained by area-level disadvantage in the Illawarra-Shoalhaven region of the NSW, Australia.* Sci Rep, 2020. **10**(1): p. 12770.

153. Troxel, W.M., et al., *Social integration, social contacts, and blood pressure dipping in African-Americans and whites.* J Hypertens, 2010. **28**(2): p. 265-71.

154. Tung, E.L., et al., *Association of Rising Violent Crime With Blood Pressure and Cardiovascular Risk: Longitudinal Evidence From Chicago, 2014-2016.* Am J Hypertens, 2019. **32**(12): p. 1192-1198.

155. Uchino, B.N., et al., *The quality of spouses' social networks contributes to each other's cardiovascular risk.* PLoS One, 2013. **8**(8): p. e71881.

156. Unger, E., et al., *Association of neighborhood characteristics with cardiovascular health in the multi-ethnic study of atherosclerosis.* Circ Cardiovasc Qual Outcomes, 2014. **7**(4): p. 524-31.

157. Wagner, K.J., et al., *Effects of neighborhood socioeconomic status on blood pressure in older adults.* Rev Saude Publica, 2016. **50**: p. 78.

158. Whittaker, K.S., et al., *Combining psychosocial data to improve prediction of cardiovascular disease risk factors and events: The National Heart, Lung, and Blood Institute--sponsored Women's Ischemia Syndrome Evaluation study.* Psychosom Med, 2012. **74**(3): p. 263-70.

159. Willets, C., et al., *Association Between Perceived Neighborhood Characteristics and Carotid Artery Intima-Media Thickness: Cross-Sectional Results From the ELSA-Brasil Study.* Glob Heart, 2019. **14**(4): p. 379-385.

160. Williams, E.D., et al., *Area-level socioeconomic status and incidence of abnormal glucose metabolism: the Australian Diabetes, Obesity and Lifestyle (AusDiab) study.* Diabetes Care, 2012. **35**(7): p. 1455-61.

161. Wing, J.J., et al., *Change in Neighborhood Characteristics and Change in Coronary Artery Calcium: A Longitudinal Investigation in the MESA (Multi-Ethnic Study of Atherosclerosis) Cohort.* Circulation, 2016. **134**(7): p. 504-13.

162. Yang, Y.C., K. Schorpp, and K.M. Harris, *Social support, social strain and inflammation: Evidence from a national longitudinal study of U.S. adults.* Social Science & Medicine, 2014. **107**: p. 124-135.

163. Yang, Y.C., et al., *Social relationships and physiological determinants of longevity across the human life span.* Proc Natl Acad Sci U S A, 2016. **113**(3): p. 578-83.

164. Yang, Y.C., C. Boen, and K. Mullan Harris, *Social relationships and hypertension in late life: evidence from a nationally representative longitudinal study of older adults.* J Aging Health, 2015. **27**(3): p. 403-31.

165. Yang, Y.C., T. Li, and Y. Ji, *Impact of social integration on metabolic functions: evidence from a nationally representative longitudinal study of US older adults.* BMC Public Health, 2013. **13**: p. 1210.

166. Yao, Y., G. Wan, and D. Meng, *Income distribution and health: can polarization explain health outcomes better than inequality?* Eur J Health Econ, 2019. **20**(4): p. 543-557.

167. Zöller, B., et al., *Neighborhood deprivation and hospitalization for venous thromboembolism in Sweden.* J Thromb Thrombolysis, 2012. **34**(3): p. 374-82.

168. Zanelatto, C., et al., *Perception of neighborhood disorder and blood pressure in adults: a multilevel population-based study.* Cad Saude Publica, 2019. **35**(2): p. e00016418.
