# Supplementary material for: Associations between dimensions of the social environment and cardiometabolic risk factors: Systematic review and meta-analysis
Source: SSM Popul Health. 2023 Nov 25;25:101559. doi: 10.1016/j.ssmph.2023.101559 (PMC10749911; doi:10.1016/j.ssmph.2023.101559)
Supplement: Multimedia component 4 [file mmc4.docx]

**Supplementary Table 2.** Summary pooled effects and between-study variance estimates with 95% confidence intervals from sex-specific meta-analyses covering the social environmental determinants of cardiometabolic biomarkers

| **Exposure** | **Outcome** | **N** | **Odds ratio (95% CI)** |  | **Total I^2^** |
| --- | --- | --- | --- | --- | --- |
| Women |  |  |  |  |  |
| *Economic and Social Disadvantage* | *CVD risk scores* | 3 | 0.98 (0.34-2.78) |  | 86% |
| *Economic and Social Disadvantage* | *Cardiovascular health-related risk factors* | 3 | 1.12 (1.03-1.22) | * | 0% |
| *Social Relationships and Norms* | *CVD risk scores* | 6 | 1.26 (0.88-1.80) |  | 9% |
| *Social Relationships and Norms* | *Metabolic and inflammatory-related risk factors* | 5 | 0.92 (0.63-1.34) |  | 9% |
| Men |  |  |  |  |  |
| *Economic and Social Disadvantage* | *CVD risk scores* | 3 | 0.59 (0.05-6.58) |  | 97% |
| *Economic and Social Disadvantage* | *Cardiovascular health-related risk factors* | 3 | 1.12 (0.67-1.87) |  | 37% |
| *Social Relationships and Norms* | *CVD risk scores* | 5 | 0.91 (0.52-1.60) |  | 60% |
| *Social Relationships and Norms* | *Metabolic and inflammatory-related risk factors* | 5 | 1.21 (0.65-2.26) |  | 17% |
| ‘***’= p <= 0.001; ‘**’ = p <= 0.01 ; ‘*’ = p <= 0.05 | |  |  |  |  |
