# Supplementary material for: Associations between dimensions of the social environment and cardiometabolic risk factors: Systematic review and meta-analysis
Source: SSM Popul Health. 2023 Nov 25;25:101559. doi: 10.1016/j.ssmph.2023.101559 (PMC10749911; doi:10.1016/j.ssmph.2023.101559)

Supplementary Figure 1. Meta-analyses of social environment dimensions and cardiometabolic risk factors

Economic and Social Disadvantage and CVD risk scores

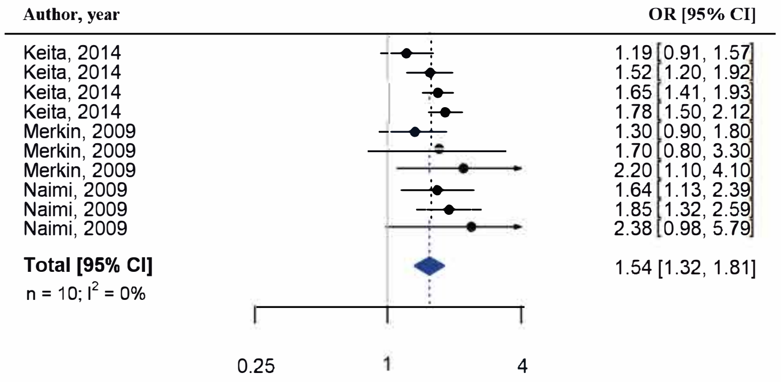

Economic and Social Disadvantage and Cardiovascular health-related risk factors

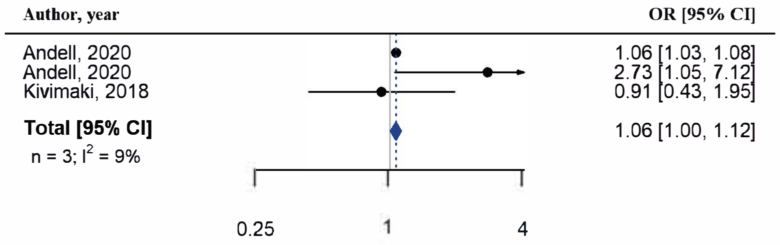

Economic and Social Disadvantage and Metabolic and inflammatory-related risk factors

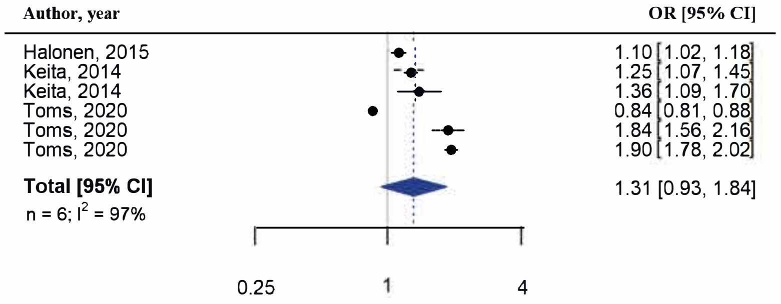

Economic and Social Disadvantage and Glucose metabolism-related risk factors

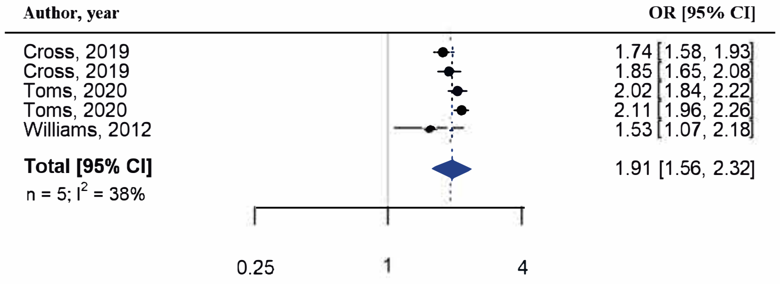

Supplement: Multimedia component 5 [file mmc5.pdf]
