# Supplementary material for: Associations between dimensions of the social environment and cardiometabolic risk factors: Systematic review and meta-analysis
Source: SSM Popul Health. 2023 Nov 25;25:101559. doi: 10.1016/j.ssmph.2023.101559 (PMC10749911; doi:10.1016/j.ssmph.2023.101559)
Supplement: Multimedia component 6 [file mmc6.docx]

**Supplementary File 3 – List of Articles Assessed for Full-Text Eligibility Following Title and Abstract Screening (n = 555)**

|  | **Title** | | **Authors** | | **Journal** | | **Year** | | **Volume** | | **Issue number** | | **Pages** | | **DOI** | |  |
| --- | --- | --- | --- | --- | --- | --- | --- | --- | --- | --- | --- | --- | --- | --- | --- | --- | --- |
| 1 | A social-network behavioral health program on sustained long-term body weight and glycemic outcomes: 2-year follow-up of a 4-month Microclinic Health Program in Jordan | | D. E. Zoughbie; J. A. Rushakoff; K. T. Watson; N. Bui; A. Ireifij; R. S. Farraj; E. L. Ding | | Prev Med Rep | | 2019 | | 13 | |  | | 160-165 | | 10.1016/j.pmedr.2018.12.002 | |  |
| 2 | | Neighbourhood deprivation and hospitalization for atrial fibrillation in Sweden | | B. Zöller; X. Li; J. Sundquist; K. Sundquist | | Europace | | 2013 | | 15 | | 8 | | 1119-27 | | 10.1093/europace/eut019 | |
| 3 | | Neighborhood deprivation and hospitalization for venous thromboembolism in Sweden | | B. Zöller; X. Li; J. Sundquist; K. Sundquist | | J Thromb Thrombolysis | | 2012 | | 34 | | 3 | | 374-82 | | 10.1007/s11239-012-0728-4 | |
| 4 | | Geographical variation in diabetes prevalence and detection in china: multilevel spatial analysis of 98,058 adults | | M. Zhou; T. Astell-Burt; Y. Bi; X. Feng; Y. Jiang; Y. Li; A. Page; L. Wang; Y. Xu; W. Zhao; G. Ning | | Diabetes Care | | 2015 | | 38 | | 1 | | 72-81 | | 10.2337/dc14-1100 | |
| 5 | | Using integrated visualization techniques to investigate associations between cardiovascular health outcomes and residential migration in Auckland, New Zealand | | J. F. Zhao; D. J. Exeter; G. Hanham; A. C. L. Lee; M. Browne; C. Grey; S. Wells | | Cartogr. Geogr. Inf. Sci. | | 2015 | | 42 | | 5 | | 381-397 | | 10.1080/15230406.2015.1013567 | |
| 6 | | Type 2 diabetes mellitus and neighborhood deprivation index: A spatial analysis in Zhejiang, China | | X. Zhang; X. Chen; W. Gong | | J Diabetes Investig | | 2019 | | 10 | | 2 | | 272-282 | | 10.1111/jdi.12899 | |
| 7 | | Examining trends in type 2 diabetes incidence, prevalence and mortality in the UK between 2004 and 2014 | | S. S. Zghebi; D. T. Steinke; M. J. Carr; M. K. Rutter; R. A. Emsley; D. M. Ashcroft | | Diabetes Obes Metab | | 2017 | | 19 | | 11 | | 1537-1545 | | 10.1111/dom.12964 | |
| 8 | | Perception of neighborhood disorder and blood pressure in adults: a multilevel population-based study | | C. Zanelatto; D. A. Höfelmann; M. W. C. Giehl; W. Nishida; J. L. Bastos | | Cad Saude Publica | | 2019 | | 35 | | 2 | |  | | 10.1590/0102-311x00016418 | |
| 9 | | Associations of overweight/obesity and socioeconomic status with hypertension prevalence across racial and ethnic groups | | D. R. Young; H. Fischer; D. Arterburn; D. Bessesen; L. Cromwell; M. F. Daley; J. Desai; A. Ferrara; S. L. Fitzpatrick; M. A. Horberg; C. Koebnick; C. L. Nau; C. Oshiro; B. Waitzfelder; A. Yamamoto | | J Clin Hypertens (Greenwich) | | 2018 | | 20 | | 3 | | 532-540 | | 10.1111/jch.13217 | |
| 10 | | Association between Social Relationship and Glycemic Control among Older Japanese: JAGES Cross-Sectional Study | | K. Yokobayashi; I. Kawachi; K. Kondo; N. Kondo; Y. Nagamine; Y. Tani; K. Shirai; S. Tazuma | | PLoS One | | 2017 | | 12 | | 1 | |  | | 10.1371/journal.pone.0169904 | |
| 11 | | Religious involvement and health outcomes among older persons in Taiwan | | D. M. Yeager; D. A. Glei; M. Au; H. S. Lin; R. P. Sloan; M. Weinstein | | Soc Sci Med | | 2006 | | 63 | | 8 | | 2228-41 | | 10.1016/j.socscimed.2006.05.007 | |
| 12 | | Association between social participation and hypertension among older people in Japan: the JAGES Study | | A. Yazawa; Y. Inoue; T. Fujiwara; A. Stickley; K. Shirai; A. Amemiya; N. Kondo; C. Watanabe; K. Kondo | | Hypertens. Res. | | 2016 | | 39 | | 11 | | 818-824 | | 10.1038/hr.2016.78 | |
| 13 | | Income distribution and health: can polarization explain health outcomes better than inequality? | | Y. Yao; G. Wan; D. Meng | | Eur J Health Econ | | 2019 | | 20 | | 4 | | 543-557 | | 10.1007/s10198-018-1016-9 | |
| 14 | | Impact of social integration on metabolic functions: evidence from a nationally representative longitudinal study of US older adults | | Y. C. Yang; T. Li; Y. Ji | | BMC Public Health | | 2013 | | 13 | |  | |  | | 10.1186/1471-2458-13-1210 | |
| 15 | | Social relationships and hypertension in late life: evidence from a nationally representative longitudinal study of older adults | | Y. C. Yang; C. Boen; K. Mullan Harris | | J Aging Health | | 2015 | | 27 | | 3 | | 403-31 | | 10.1177/0898264314551172 | |
| 16 | | Social relationships and physiological determinants of longevity across the human life span | | Y. C. Yang; C. Boen; K. Gerken; T. Li; K. Schorpp; K. M. Harris | | Proc Natl Acad Sci U S A | | 2016 | | 113 | | 3 | | 578-83 | | 10.1073/pnas.1511085112 | |
| 17 | | Exploring psychosocial pathways between neighbourhood characteristics and stroke in older adults: the cardiovascular health study | | T. Yan; J. J. Escarce; L. J. Liang; W. T. Longstreth, Jr.; S. S. Merkin; B. Ovbiagele; S. D. Vassar; T. Seeman; C. Sarkisian; A. F. Brown | | Age Ageing | | 2013 | | 42 | | 3 | | 391-7 | | 10.1093/ageing/afs179 | |
| 18 | | Cardiovascular Diseases and Risk-Factor Burden in Urban and Rural Communities in High-, Middle-, and Low-Income Regions of China: A Large Community-Based Epidemiological Study | | R. Yan; W. Li; L. Yin; Y. Wang; J. Bo | | J Am Heart Assoc | | 2017 | | 6 | | 2 | |  | | 10.1161/jaha.116.004445 | |
| 19 | | [Study on health-related behaviors among males in rural and urban residents from Guangdong province] | | H. Y. Xie; Y. H. Zhang; W. J. Ma; Y. J. Xu; X. J. Xu; Q. M. Cai; X. L. Song; H. F. Xu; S. P. Nie | | Zhonghua Liu Xing Bing Xue Za Zhi | | 2010 | | 31 | | 12 | | 1363-7 | |  | |
| 20 | | Inequities in CHD incidence and case fatality by neighborhood deprivation | | M. Winkleby; K. Sundquist; C. Cubbin | | Am J Prev Med | | 2007 | | 32 | | 2 | | 97-106 | | 10.1016/j.amepre.2006.10.002 | |
| 21 | | Change in Neighborhood Characteristics and Change in Coronary Artery Calcium: A Longitudinal Investigation in the MESA (Multi-Ethnic Study of Atherosclerosis) Cohort | | J. J. Wing; E. August; S. D. Adar; A. L. Dannenberg; A. Hajat; B. N. Sánchez; J. H. Stein; M. C. Tattersall; A. V. Diez Roux | | Circulation | | 2016 | | 134 | | 7 | | 504-13 | | 10.1161/circulationaha.115.020534 | |
| 22 | | Area-level socioeconomic status and incidence of abnormal glucose metabolism: the Australian Diabetes, Obesity and Lifestyle (AusDiab) study | | E. D. Williams; D. J. Magliano; P. Z. Zimmet; A. M. Kavanagh; C. E. Stevenson; B. F. Oldenburg; J. E. Shaw | | Diabetes Care | | 2012 | | 35 | | 7 | | 1455-61 | | 10.2337/dc11-1410 | |
| 23 | | Association Between Perceived Neighborhood Characteristics and Carotid Artery Intima-Media Thickness: Cross-Sectional Results From the ELSA-Brasil Study | | C. Willets; I. S. Santos; P. A. Lotufo; I. M. Benseñor; C. K. Suemoto | | Glob Heart | | 2019 | | 14 | | 4 | | 379-385 | | 10.1016/j.gheart.2019.09.002 | |
| 24 | | A multilevel analysis of urban neighborhood socioeconomic disadvantage and health in late life | | R. G. Wight; J. R. Cummings; D. Miller-Martinez; A. S. Karlamangla; T. E. Seeman; C. S. Aneshensel | | Soc Sci Med | | 2008 | | 66 | | 4 | | 862-72 | | 10.1016/j.socscimed.2007.11.002 | |
| 25 | | Combining psychosocial data to improve prediction of cardiovascular disease risk factors and events: The National Heart, Lung, and Blood Institute--sponsored Women's Ischemia Syndrome Evaluation study | | K. S. Whittaker; D. S. Krantz; T. Rutledge; B. D. Johnson; A. J. Wawrzyniak; V. Bittner; J. A. Eastwood; W. Eteiba; C. E. Cornell; C. J. Pepine; D. A. Vido; E. Handberg; C. N. Merz | | Psychosom Med | | 2012 | | 74 | | 3 | | 263-70 | | 10.1097/PSY.0b013e31824a58ff | |
| 26 | | Racial/ethnic residential segregation and self-reported hypertension among US- and foreign-born blacks in New York City | | K. White; L. N. Borrell; D. W. Wong; S. Galea; G. Ogedegbe; M. M. Glymour | | Am J Hypertens | | 2011 | | 24 | | 8 | | 904-10 | | 10.1038/ajh.2011.69 | |
| 27 | | Long-term effects of neighbourhood deprivation on diabetes risk: quasi-experimental evidence from a refugee dispersal policy in Sweden | | J. S. White; R. Hamad; X. Li; S. Basu; H. Ohlsson; J. Sundquist; K. Sundquist | | Lancet Diabetes Endocrinol | | 2016 | | 4 | | 6 | | 517-24 | | 10.1016/s2213-8587(16)30009-2 | |
| 28 | | Cardiovascular disease occurrence in two close but different social environments | | C. Wennerholm; B. Grip; A. Johansson; H. Nilsson; M. L. Honkasalo; T. Faresjo | | Int. J. Health Geogr. | | 2011 | | 10 | |  | |  | | 10.1186/1476-072X-10-5 | |
| 29 | | Social relationships and myocardial infarction: a case-control study | | C. L. Welin; A. Rosengren; L. W. Wilhelmsen | | J Cardiovasc Risk | | 1996 | | 3 | | 2 | | 183-90 | |  | |
| 30 | | Can a sustainable community intervention reduce the health gap?--10-year evaluation of a Swedish community intervention program for the prevention of cardiovascular disease | | L. Weinehall; G. Hellsten; K. Boman; G. Hallmans; K. Asplund; S. Wall | | Scand J Public Health Suppl | | 2001 | | 56 | |  | | 59-68 | |  | |
| 31 | | The effect of neighborhood, socioeconomic status and a community-based program on multi-disease health screening in an Asian population: a controlled intervention study | | L. E. Wee; G. C. Koh | | Prev Med | | 2011 | | 53 | |  | | 64-9 | | 10.1016/j.ypmed.2011.05.005 | |
| 32 | | Park green green spaces, public health and social inequalities: Understanding the interrelationships for policy implications | | Q. Wang; Z. L. Lan | | Land Use Pol. | | 2019 | | 83 | |  | | 66-74 | | 10.1016/j.landusepol.2019.01.026 | |
| 33 | | Association of the Robert Wood Johnson Foundations' social determinants of health and Medicare hospitalisations for ischaemic strokes: a cross-sectional data analysis | | A. Wang; A. N. Kho; D. D. French | | Open Heart | | 2020 | | 7 | | 1 | |  | | 10.1136/openhrt-2019-001189 | |
| 34 | | The weight of networks: the role of social ties and ethnic media in mitigating obesity and hypertension among Latinas | | N. Walter; C. Robbins; S. T. Murphy; S. J. Ball-Rokeach | | Ethn Health | | 2019 | | 24 | | 7 | | 790-803 | | 10.1080/13557858.2017.1373071 | |
| 35 | | Classifying high-prevalence neighborhoods for cardiovascular disease in Texas | | K. E. Walker; S. M. Crotty | | Appl. Geogr. | | 2015 | | 57 | |  | | 22-31 | | 10.1016/j.apgeog.2014.11.011 | |
| 36 | | Effects of neighborhood socioeconomic status on blood pressure in older adults | | K. J. Wagner; A. F. Boing; S. V. Subramanian; D. A. Höfelmann; E. D'Orsi | | Rev Saude Publica | | 2016 | | 50 | |  | |  | | 10.1590/s1518-8787.2016050006595 | |
| 37 | | Social networks as predictors of ischemic heart disease, cancer, stroke and hypertension: incidence, survival and mortality | | T. M. Vogt; J. P. Mullooly; D. Ernst; C. R. Pope; J. F. Hollis | | J Clin Epidemiol | | 1992 | | 45 | | 6 | | 659-66 | | 10.1016/0895-4356(92)90138-d | |
| 38 | | Unfavorable and favorable changes in modifiable risk factors and incidence of coronary heart disease: The Whitehall II cohort study | | M. Virtanen; J. Vahtera; A. Singh-Manoux; M. Elovainio; J. E. Ferrie; M. Kivimäki | | Int J Cardiol | | 2018 | | 269 | |  | |  | | 10.1016/j.ijcard.2018.07.005 | |
| 39 | | Geographical variation of diabetic emergencies attended by prehospital Emergency Medical Services is associated with measures of ethnicity and socioeconomic status | | M. Villani; A. Earnest; K. Smith; B. de Courten; S. Zoungas | | Sci Rep | | 2018 | | 8 | | 1 | |  | | 10.1038/s41598-018-23457-5 | |
| 40 | | [The connection between the socioeconomic status and stroke in Budapest] | | I. Vastagh; I. Szőcs; F. Oberfrank; A. Ajtay; D. Bereczki | | Ideggyogy Sz | | 2020 | | 73 | |  | | 389-397 | | 10.18071/isz.73.0389 | |
| 41 | | Socioeconomic Status and Incidence of Hospitalization With Lower-Extremity Peripheral Artery Disease: Atherosclerosis Risk in Communities Study | | P. Vart; J. Coresh; L. Kwak; S. H. Ballew; G. Heiss; K. Matsushita | | J Am Heart Assoc | | 2017 | | 6 | | 8 | |  | | 10.1161/jaha.116.004995 | |
| 42 | | Population density is beneficially associated with 12-year diabetes risk marker change among residents of lower socio-economic neighborhoods | | J. Van Cauwenberg; D. Dunstan; E. Cerin; M. J. Koohsari; T. Sugiyama; N. Owen | | Health Place | | 2019 | | 57 | |  | | 74-81 | | 10.1016/j.healthplace.2019.02.006 | |
| 43 | | Loneliness, social isolation and risk of cardiovascular disease in the English Longitudinal Study of Ageing | | N. K. Valtorta; M. Kanaan; S. Gilbody; B. Hanratty | | Eur J Prev Cardiol | | 2018 | | 25 | | 13 | | 1387-1396 | | 10.1177/2047487318792696 | |
| 44 | | Residential Segregation and Hypertension Prevalence in Black and White Older Adults | | T. Usher; D. J. Gaskin; K. Bower; C. Rohde; R. J. Thorpe, Jr. | | J Appl Gerontol | | 2018 | | 37 | | 2 | | 177-202 | | 10.1177/0733464816638788 | |
| 45 | | Association of neighborhood characteristics with cardiovascular health in the multi-ethnic study of atherosclerosis | | E. Unger; A. V. Diez-Roux; D. M. Lloyd-Jones; M. S. Mujahid; J. A. Nettleton; A. Bertoni; S. E. Badon; H. Ning; N. B. Allen | | Circ Cardiovasc Qual Outcomes | | 2014 | | 7 | | 4 | | 524-31 | | 10.1161/circoutcomes.113.000698 | |
| 46 | | Cardiovascular effects of social support in the work place: twenty-four-hour ECG monitoring of men and women | | A. L. Undén; K. Orth-Gomér; S. Elofsson | | Psychosom Med | | 1991 | | 53 | | 1 | | 50-60 | | 10.1097/00006842-199101000-00005 | |
| 47 | | The quality of spouses' social networks contributes to each other's cardiovascular risk | | B. N. Uchino; T. W. Smith; M. Carlisle; W. C. Birmingham; K. C. Light | | PLoS One | | 2013 | | 8 | | 8 | |  | | 10.1371/journal.pone.0071881 | |
| 48 | | The quality of social networks predicts age-related changes in cardiovascular reactivity to stress | | B. N. Uchino; R. G. Kent de Grey; S. Cronan | | Psychol Aging | | 2016 | | 31 | | 4 | | 321-6 | | 10.1037/pag0000092 | |
| 49 | | Heterogeneity in the social networks of young and older adults: prediction of mental health and cardiovascular reactivity during acute stress | | B. N. Uchino; J. Holt-Lunstad; D. Uno; J. B. Flinders | | J Behav Med | | 2001 | | 24 | | 4 | | 361-82 | | 10.1023/a:1010634902498 | |
| 50 | | Geographical pattern of female deaths from myocardial infarction in an urban population: Fatal outcome out-of-hospital related to socio-economic deprivation | | P. Tydén; G. Engström; O. Hansen; B. Hedblad; L. Janzon | | J. Intern. Med. (GBR) | | 2001 | | 250 | | 3 | | 201-207 | | 10.1046/j.1365-2796.2001.00877.x | |
| 51 | | Police-Recorded Crime and Disparities in Obesity and Blood Pressure Status in Chicago | | E. L. Tung; K. E. Wroblewski; K. Boyd; J. A. Makelarski; M. E. Peek; S. T. Lindau | | J Am Heart Assoc | | 2018 | | 7 | | 7 | |  | | 10.1161/jaha.117.008030 | |
| 52 | | Association of Rising Violent Crime With Blood Pressure and Cardiovascular Risk: Longitudinal Evidence From Chicago, 2014-2016 | | E. L. Tung; R. F. M. Chua; S. A. Besser; S. T. Lindau; M. Kolak; E. C. Anyanwu; J. K. Liao; C. E. Tabit | | Am J Hypertens | | 2019 | | 32 | | 12 | | 1192-1198 | | 10.1093/ajh/hpz134 | |
| 53 | | Social integration, social contacts, and blood pressure dipping in African-Americans and whites | | W. M. Troxel; D. J. Buysse; M. Hall; T. W. Kamarck; P. J. Strollo; J. F. Owens; S. E. Reis; K. A. Matthews | | J Hypertens | | 2010 | | 28 | | 2 | | 265-71 | | 10.1097/HJH.0b013e328333ab01 | |
| 54 | | Geographic variation in cardiometabolic risk factor prevalence explained by area-level disadvantage in the Illawarra-Shoalhaven region of the NSW, Australia | | R. Toms; D. J. Mayne; X. Feng; A. Bonney | | Sci Rep | | 2020 | | 10 | | 1 | |  | | 10.1038/s41598-020-69552-4 | |
| 55 | | The geography of diabetes in London, Canada: the need for local level policy for prevention and management | | J. W. Tompkins; I. N. Luginaah; G. L. Booth; S. B. Harris | | Int J Environ Res Public Health | | 2010 | | 7 | | 5 | | 2407-22 | | 10.3390/ijerph7052407 | |
| 56 | | A comparison of Australian rural and metropolitan cardiovascular risk and mortality: the Greater Green Triangle and North West Adelaide population surveys | | P. Tideman; A. W. Taylor; E. Janus; B. Philpot; R. Clark; E. Peach; T. Laatikainen; E. Vartiainen; R. Tirimacco; A. Montgomerie; J. Grant; V. Versace; J. A. Dunbar | | BMJ Open | | 2013 | | 3 | | 8 | |  | | 10.1136/bmjopen-2013-003203 | |
| 57 | | Greater incidence of both fatal and nonfatal strokes in disadvantaged areas: the Northeast Melbourne Stroke Incidence Study | | A. G. Thrift; H. M. Dewey; J. W. Sturm; S. L. Paul; A. K. Gilligan; V. K. Srikanth; R. A. Macdonell; J. J. McNeil; M. R. Macleod; G. A. Donnan | | Stroke | | 2006 | | 37 | | 3 | | 877-82 | | 10.1161/01.STR.0000202588.95876.a7 | |
| 58 | | Reducing diabetes risk in American Indian women | | J. L. Thompson; P. Allen; D. L. Helitzer; C. Qualls; A. N. Whyte; V. K. Wolfe; C. J. Herman | | Am J Prev Med | | 2008 | | 34 | | 3 | | 192-201 | | 10.1016/j.amepre.2007.11.014 | |
| 59 | | Blood pressure variations across areas in the greater Stockholm region: analysis of 74,000 18-year-old men | | T. Theorell; J. Svensson; S. Knox; B. Ahlborg | | Soc Sci Med | | 1982 | | 16 | | 4 | | 469-73 | | 10.1016/0277-9536(82)90055-7 | |
| 60 | | A small-area analysis of inequalities in chronic disease prevalence across urban and non-urban communities in the Province of Nova Scotia, Canada, 2007-2011 | | M. Terashima; D. G. Rainham; A. R. Levy | | BMJ Open | | 2014 | | 4 | | 5 | |  | | 10.1136/bmjopen-2013-004459 | |
| 61 | | Stress-Associated Neurobiological Pathway Linking Socioeconomic Disparities to Cardiovascular Disease | | A. Tawakol; M. T. Osborne; Y. Wang; B. Hammed; B. Tung; T. Patrich; B. Oberfeld; A. Ishai; L. M. Shin; M. Nahrendorf; E. T. Warner; J. Wasfy; Z. A. Fayad; K. Koenen; P. M. Ridker; R. K. Pitman; K. A. Armstrong | | J Am Coll Cardiol | | 2019 | | 73 | | 25 | | 3243-3255 | | 10.1016/j.jacc.2019.04.042 | |
| 62 | | Neighborhood socioeconomic status and the prevalence of stroke and coronary heart disease in rural China: a population-based study | | X. Tang; D. T. Laskowitz; L. He; T. Østbye; J. P. Bettger; Y. Cao; N. Li; J. Li; Z. Zhang; J. Liu; L. Yu; H. Xu; Y. Hu; L. B. Goldstein | | Int J Stroke | | 2015 | | 10 | | 3 | | 388-95 | | 10.1111/ijs.12343 | |
| 63 | | Socioeconomic gap between neighborhoods of Budapest: Striking impact on stroke and possible explanations | | I. Szőcs; D. Bereczki; A. Ajtay; F. Oberfrank; I. Vastagh | | PLoS One | | 2019 | | 14 | | 2 | |  | | 10.1371/journal.pone.0212519 | |
| 64 | | Association between Area-Level Socio-Economic Status and Hypertension in Eag States of India : An Insight from Nfhs-Iv 2015-16 | | P. K. Swain; B. Behera; D. Das | | Int. J. Agric. Stat. Sci. | | 2019 | | 15 | | 1 | | 39-52 | |  | |
| 65 | | Neighborhood socioeconomic environment and incidence of coronary heart disease: A follow-up study of 25,319 women and men in Sweden | | K. Sundquist; M. Winkleby; H. Ahlen; S. E. Johansson | | Am. J. Epidemiol. | | 2004 | | 159 | | 7 | | 655-662 | | 10.1093/aje/kwh096 | |
| 66 | | Neighborhood violent crime and unemployment increase the risk of coronary heart disease: a multilevel study in an urban setting | | K. Sundquist; H. Theobald; M. Yang; X. Li; S. E. Johansson; J. Sundquist | | Soc Sci Med | | 2006 | | 62 | | 8 | | 2061-71 | | 10.1016/j.socscimed.2005.08.051 | |
| 67 | | Neighbourhood deprivation and incidence of coronary heart disease: a multilevel study of 2.6 million women and men in Sweden | | K. Sundquist; M. Malmström; S. E. Johansson | | J Epidemiol Community Health | | 2004 | | 58 | | 1 | | 71-7 | | 10.1136/jech.58.1.71 | |
| 68 | | Low linking social capital as a predictor of coronary heart disease in Sweden: a cohort study of 2.8 million people | | J. Sundquist; S. E. Johansson; M. Yang; K. Sundquist | | Soc Sci Med | | 2006 | | 62 | | 4 | | 954-63 | | 10.1016/j.socscimed.2005.06.049 | |
| 69 | | Individual and contextual correlates of cardiovascular diseases among adults in the United States: a geospatial and multilevel analysis | | W. J. Sun; F. Gong; J. Xu | | GeoJournal | | 2020 | | 85 | | 6 | | 1685-1700 | | 10.1007/s10708-019-10049-7 | |
| 70 | | Social support, stress, and blood pressure in black adults | | D. S. Strogatz; J. B. Croft; S. A. James; N. L. Keenan; S. R. Browning; J. M. Garrett; A. B. Curtis | | Epidemiology | | 1997 | | 8 | | 5 | | 482-7 | | 10.1097/00001648-199709000-00002 | |
| 71 | | Neighborhood socioeconomic context, individual income and myocardial infarction | | M. K. Stjärne; J. Fritzell; A. P. De Leon; J. Hallqvist | | Epidemiology | | 2006 | | 17 | | 1 | | 14-23 | | 10.1097/01.ede.0000187178.51024.a7 | |
| 72 | | Diabetes and the socioeconomic and built environment: geovisualization of disease prevalence and potential contextual associations using ring maps | | J. E. Stewart; S. E. Battersby; A. Lopez-De Fede; K. C. Remington; J. W. Hardin; K. Mayfield-Smith | | Int J Health Geogr | | 2011 | | 10 | |  | |  | | 10.1186/1476-072x-10-18 | |
| 73 | | Individual and area-level determinants associated with C-reactive protein as a marker of cardiometabolic risk among adults: Results from the German National Health Interview and Examination Survey 2008-2011 | | H. Steppuhn; D. Laußmann; J. Baumert; L. Kroll; T. Lampert; D. Plaß; C. Scheidt-Nave; C. Heidemann | | PLoS One | | 2019 | | 14 | | 2 | |  | | 10.1371/journal.pone.0211774 | |
| 74 | | The hidden cost of moving up: type 2 diabetes and the escape from persistent poverty in the American South | | R. H. Steckel | | Am J Hum Biol | | 2013 | | 25 | | 4 | | 508-15 | | 10.1002/ajhb.22399 | |
| 75 | | Seven-year follow-up of blood pressure in the Healthy Old People in Edinburgh (HOPE) cohort | | J. M. Starr; S. Inch; S. Cross; I. J. Deary | | J Hum Hypertens | | 2000 | | 14 | | 12 | | 773-8 | | 10.1038/sj.jhh.1001039 | |
| 76 | | Neighborhood crime is differentially associated with cardiovascular risk factors as a function of race and sex | | M. R. Sprung; L. M. D. Faulkner; M. K. Evans; A. B. Zonderman; S. R. Waldstein | | J Public Health Res | | 2019 | | 8 | | 3 | |  | | 10.4081/jphr.2019.1643 | |
| 77 | | The Effects of Anger Management and Social Contact on Risk of Myocardial-Infarction in Type-as and Type-Bs | | J. Spicer; R. Jackson; R. Scragg | | Psychol. Health | | 1993 | | 8 | | 4 | | 243-255 | | 10.1080/08870449308401919 | |
| 78 | | Blood pressure levels and longitudinal changes in relation to social network factors | | D. E. Sörman; P. Hansson; M. Rönnlund | | Psihologijske Teme | | 2016 | | 25 | | 1 | | 59-73 | |  | |
| 79 | | Using Geographical Convergence of Obesity, Cardiovascular Disease, and Type 2 Diabetes at the Neighborhood Level to Inform Policy and Practice | | K. Smurthwaite; N. Bagheri | | Prev Chronic Dis | | 2017 | | 14 | |  | |  | | 10.5888/pcd14.170170 | |
| 80 | | The psychosocial work environment and incident diabetes in Ontario, Canada | | P. M. Smith; R. H. Glazier; H. Lu; C. A. Mustard | | Occup Med (Lond) | | 2012 | | 62 | | 6 | | 413-9 | | 10.1093/occmed/kqs128 | |
| 81 | | Individual social class, area-based deprivation, cardiovascular disease risk factors, and mortality: the Renfrew and Paisley Study | | G. D. Smith; C. Hart; G. Watt; D. Hole; V. Hawthorne | | J Epidemiol Community Health | | 1998 | | 52 | | 6 | | 399-405 | | 10.1136/jech.52.6.399 | |
| 82 | | Childhood adversity, adult neighborhood context, and cumulative biological risk for chronic diseases in adulthood | | N. Slopen; A. Non; D. R. Williams; A. L. Roberts; M. A. Albert | | Psychosom Med | | 2014 | | 76 | | 7 | | 481-9 | | 10.1097/psy.0000000000000081 | |
| 83 | | Early origins of inflammation: An examination of prenatal and childhood social adversity in a prospective cohort study | | N. Slopen; E. B. Loucks; A. A. Appleton; I. Kawachi; L. D. Kubzansky; A. L. Non; S. Buka; S. E. Gilman | | Psychoneuroendocrinology | | 2015 | | 51 | |  | | 403-13 | | 10.1016/j.psyneuen.2014.10.016 | |
| 84 | | The complex relationship with health: Rural and urban poor' women" | | S. Singh; R. Zhou; X. Li; L. P. Tong | | Int. Soc. Work | | 2016 | | 59 | | 1 | | 32-46 | | 10.1177/0020872813503862 | |
| 85 | | Social Determinants of Health in the United States: Addressing Major Health Inequality Trends for the Nation, 1935-2016 | | G. K. Singh; G. P. Daus; M. Allender; C. T. Ramey; E. K. Martin; C. Perry; A. A. L. Reyes; I. P. Vedamuthu | | Int J MCH AIDS | | 2017 | | 6 | | 2 | | 139-164 | | 10.21106/ijma.236 | |
| 86 | | Individual Income, Area Deprivation, and Health: Do Income-Related Health Inequalities Vary by Small Area Deprivation? | | M. Siegel; A. Mielck; W. Maier | | Health Econ | | 2015 | | 24 | | 11 | | 1523-30 | | 10.1002/hec.3102 | |
| 87 | | Association of socioeconomic status with functional capacity, heart rate recovery, and all-cause mortality | | M. H. Shishehbor; D. Litaker; C. E. Pothier; M. S. Lauer | | Jama | | 2006 | | 295 | | 7 | | 784-92 | | 10.1001/jama.295.7.784 | |
| 88 | | Relation of Neighborhood Disadvantage to Heart Failure Symptoms and Hospitalizations | | T. E. Shirey; Y. Hu; Y. A. Ko; A. Nayak; E. Udeshi; S. Patel; A. A. Morris | | Am J Cardiol | | 2021 | | 140 | |  | | 83-90 | | 10.1016/j.amjcard.2020.10.057 | |
| 89 | | Relationship between social engagement and diabetes incidence in a middle-aged population: Results from a longitudinal nationwide survey in Japan | | T. Shibayama; H. Noguchi; H. Takahashi; N. Tamiya | | J Diabetes Investig | | 2018 | | 9 | | 5 | | 1060-1066 | | 10.1111/jdi.12820 | |
| 90 | | The Effect of Neighborhood Disadvantage on Diabetes Prevalence | | L. Sheets; G. F. Petroski; J. Jaddoo; Y. Barnett; C. Barnett; L. E. H. Kelley; V. Raman; A. J. H. Kind; J. C. Parker | | AMIA Annu Symp Proc | | 2017 | | 2017 | |  | | 1547-1553 | |  | |
| 91 | | Incorporating socio-economic and risk factor diversity into the development of an African-American community blood pressure control program | | B. Shakoor-Abdullah; J. M. Kotchen; W. E. Walker; T. H. Chelius; R. G. Hoffmann | | Ethn Dis | | 1997 | | 7 | | 3 | | 175-83 | |  | |
| 92 | | Disparities in Socioeconomic Context and Association With Blood Pressure Control and Cardiovascular Outcomes in ALLHAT | | A. Shahu; J. Herrin; S. S. Dhruva; N. R. Desai; B. R. Davis; H. M. Krumholz; E. S. Spatz | | J Am Heart Assoc | | 2019 | | 8 | | 15 | |  | | 10.1161/jaha.119.012277 | |
| 93 | | Social relationships and their biological correlates: Coronary Artery Risk Development in Young Adults (CARDIA) study | | T. E. Seeman; T. L. Gruenewald; S. Cohen; D. R. Williams; K. A. Matthews | | Psychoneuroendocrinology | | 2014 | | 43 | |  | | 126-38 | | 10.1016/j.psyneuen.2014.02.008 | |
| 94 | | Association of community types and features in a case-control analysis of new onset type 2 diabetes across a diverse geography in Pennsylvania | | B. S. Schwartz; J. Pollak; M. N. Poulsen; K. Bandeen-Roche; K. Moon; J. DeWalle; K. Siegel; C. Mercado; G. Imperatore; A. G. Hirsch | | BMJ Open | | 2021 | | 11 | | 1 | |  | | 10.1136/bmjopen-2020-043528 | |
| 95 | | Tracking stroke hospitalization clusters over time and associations with county-level socioeconomic and healthcare characteristics | | L. J. Schieb; L. R. Mobley; M. George; M. Casper | | Stroke | | 2013 | | 44 | | 1 | | 146-52 | | 10.1161/strokeaha.112.669705 | |
| 96 | | Community Characteristics are Associated with Blood Pressure Levels in a Racially Integrated Community | | L. J. Samuel; R. J. Thorpe, Jr.; K. M. Bower; T. A. LaVeist | | J Urban Health | | 2015 | | 92 | | 3 | | 403-14 | | 10.1007/s11524-015-9936-5 | |
| 97 | | Associations between social relationship measures, serum brain-derived neurotrophic factor, and risk of stroke and dementia | | J. Salinas; A. Beiser; J. J. Himali; C. L. Satizabal; H. J. Aparicio; G. Weinstein; F. J. Mateen; L. F. Berkman; J. Rosand; S. Seshadri | | Alzheimers Dement (N Y) | | 2017 | | 3 | | 2 | | 229-237 | | 10.1016/j.trci.2017.03.001 | |
| 98 | | Association between area-level socioeconomic status, accessibility and diabetes-related hospitalisations: a cross-sectional analysis of data from Western Victoria, Australia | | M. A. Sajjad; K. L. Holloway-Kew; M. Mohebbi; M. A. Kotowicz; L. L. F. de Abreu; P. M. Livingston; M. Khasraw; S. Hakkennes; T. L. Dunning; S. Brumby; R. S. Page; A. G. Sutherland; S. Venkatesh; L. J. Williams; S. L. Brennan-Olsen; J. A. Pasco | | BMJ Open | | 2019 | | 9 | | 5 | |  | | 10.1136/bmjopen-2018-026880 | |
| 99 | | Explaining income-related inequalities in cardiovascular risk factors in Tunisian adults during the last decade: comparison of sensitivity analysis of logistic regression and Wagstaff decomposition analysis | | O. Saidi; N. Zoghlami; K. E. Bennett; P. A. Mosquera; D. Malouche; S. Capewell; H. B. Romdhane; M. O'Flaherty | | Int J Equity Health | | 2019 | | 18 | | 1 | |  | | 10.1186/s12939-019-1047-6 | |
| 100 | | Number of Social Determinants of Health and Fatal and Nonfatal Incident Coronary Heart Disease in the REGARDS Study | | M. M. Safford; E. Reshetnyak; M. R. Sterling; J. S. Richman; P. M. Muntner; R. W. Durant; J. Booth; L. C. Pinheiro | | Circulation | | 2021 | | 143 | | 3 | | 244-253 | | 10.1161/circulationaha.120.048026 | |
| 101 | | Environmental determinants of cardiovascular diseases risk factors: a qualitative directed content analysis | | L. Sabzmakan; E. Mohammadi; M. A. Morowatisharifabad; A. Afaghi; M. H. Naseri; M. Mirzaei | | Iran Red Crescent Med J | | 2014 | | 16 | | 5 | |  | | 10.5812/ircmj.11573 | |
| 102 | | Social networks are associated with lower mortality rates among women with suspected coronary disease: the National Heart, Lung, and Blood Institute-Sponsored Women's Ischemia Syndrome Evaluation study | | T. Rutledge; S. E. Reis; M. Olson; J. Owens; S. F. Kelsey; C. J. Pepine; S. Mankad; W. J. Rogers; C. N. Bairey Merz; G. Sopko; C. E. Cornell; B. Sharaf; K. A. Matthews | | Psychosom Med | | 2004 | | 66 | | 6 | | 882-8 | | 10.1097/01.psy.0000145819.94041.52 | |
| 103 | | Social networks and incident stroke among women with suspected myocardial ischemia | | T. Rutledge; S. E. Linke; M. B. Olson; J. Francis; B. D. Johnson; V. Bittner; K. York; C. McClure; S. F. Kelsey; S. E. Reis; C. E. Cornell; V. Vaccarino; D. S. Sheps; L. J. Shaw; D. S. Krantz; S. Parashar; C. N. Merz | | Psychosom Med | | 2008 | | 70 | | 3 | | 282-7 | | 10.1097/PSY.0b013e3181656e09 | |
| 104 | | The community need index. A new tool pinpoints health care disparities in communities throughout the nation | | R. Roth; E. Barsi | | Health Prog | | 2005 | | 86 | | 4 | | 32-8 | |  | |
| 105 | | Area social characteristics and carotid atherosclerosis | | M. Rosvall; G. Engström; B. Hedblad; L. Janzon; G. Berglund | | Eur. J. Public Health | | 2007 | | 17 | | 4 | | 333-339 | | 10.1093/eurpub/ckl239 | |
| 106 | | Coronary disease in relation to social support and social class in Swedish men. A 15 year follow-up in the study of men born in 1933 | | A. Rosengren; L. Wilhelmsen; K. Orth-Gomér | | Eur Heart J | | 2004 | | 25 | | 1 | | 56-63 | | 10.1016/j.ehj.2003.10.005 | |
| 107 | | Neighborhood disparities in incident hospitalized myocardial infarction in four U.S. communities: the ARIC surveillance study | | K. M. Rose; C. M. Suchindran; R. E. Foraker; E. A. Whitsel; W. D. Rosamond; G. Heiss; J. L. Wood | | Ann Epidemiol | | 2009 | | 19 | | 12 | | 867-74 | | 10.1016/j.annepidem.2009.07.092 | |
| 108 | | Sociodemographic disparities in hypertension prevalence: Results from the first Portuguese National Health Examination Survey | | A. P. Rodrigues; V. Gaio; I. Kislaya; S. Graff-Iversen; E. Cordeiro; A. C. Silva; S. Namorado; M. Barreto; A. P. Gil; L. Antunes; A. Santos; J. P. Miguel; B. Nunes; C. M. Dias | | Rev Port Cardiol | | 2019 | | 38 | | 8 | | 547-555 | | 10.1016/j.repc.2018.10.012 | |
| 109 | | Psychosocial risk factors and heart failure hospitalization: a prospective cohort study | | N. H. Rod; I. Andersen; E. Prescott | | Am J Epidemiol | | 2011 | | 174 | | 6 | | 672-80 | | 10.1093/aje/kwr144 | |
| 110 | | Perceived neighborhood social cohesion and cardiometabolic risk: a gene × environment study | | J. W. Robinette; J. D. Boardman; E. Crimmins | | Biodemography Soc Biol | | 2020 | | 65 | | 1 | |  | | 10.1080/19485565.2019.1568672 | |
| 111 | | Perceived neighborhood social cohesion and cardiometabolic risk: a gene × environment study | | J. W. Robinette; J. D. Boardman; E. Crimmins | | Biodemography Soc Biol | | 2018 | | 64 | |  | | 173-186 | | 10.1080/19485565.2019.1579084 | |
| 112 | | The influence of socioeconomic status on future risk for developing Type 2 diabetes in the Canadian population between 2011 and 2022: differential associations by sex | | L. A. Rivera; M. Lebenbaum; L. C. Rosella | | Int J Equity Health | | 2015 | | 14 | |  | |  | | 10.1186/s12939-015-0245-0 | |
| 113 | | Association between individual-level and community-level socio-economic status and blood pressure among Inuit in Greenland | | M. Riva; C. V. Larsen; P. Bjerregaard | | Int J Circumpolar Health | | 2016 | | 75 | |  | |  | | 10.3402/ijch.v75.32757 | |
| 114 | | Heart failure hospitalisations and deaths in New Zealand: patterns by deprivation and ethnicity | | T. Riddell | | N Z Med J | | 2004 | | 118 | |  | |  | |  | |
| 115 | | Neighbourhood socioeconomic deprivation and allostatic load: a multi-cohort study | | A. I. Ribeiro; S. Fraga; M. Kelly-Irving; C. Delpierre; S. Stringhini; M. Kivimaki; S. Joost; I. Guessous; M. Gandini; P. Vineis; H. Barros | | Sci Rep | | 2019 | | 9 | | 1 | |  | | 10.1038/s41598-019-45432-4 | |
| 116 | | Impact of Multiple Social Determinants of Health on Incident Stroke | | E. Reshetnyak; M. Ntamatungiro; L. C. Pinheiro; V. J. Howard; A. P. Carson; K. D. Martin; M. M. Safford | | Stroke | | 2020 | | 51 | | 8 | | 2445-2453 | | 10.1161/strokeaha.120.028530 | |
| 117 | | Social networks and coronary heart disease among Japanese men in Hawaii | | D. Reed; D. McGee; K. Yano; M. Feinleib | | Am J Epidemiol | | 1983 | | 117 | | 4 | | 384-96 | | 10.1093/oxfordjournals.aje.a113557 | |
| 118 | | Psychosocial processes and general susceptibility to chronic disease | | D. Reed; D. McGee; K. Yano | | Am J Epidemiol | | 1984 | | 119 | | 3 | | 356-70 | | 10.1093/oxfordjournals.aje.a113754 | |
| 119 | | [Relationship between social network and hypertension in older people in Spain] | | A. Redondo-Sendino; P. Guallar-Castillón; J. R. Banegas; F. Rodríguez-Artalejo | | Rev Esp Cardiol | | 2005 | | 58 | | 11 | | 1294-301 | |  | |
| 120 | | Provider and recipient factors that may moderate the effectiveness of received support: examining the effects of relationship quality and expectations for support on behavioral and cardiovascular reactions | | M. Reblin; B. N. Uchino; T. W. Smith | | J Behav Med | | 2010 | | 33 | | 6 | | 423-31 | | 10.1007/s10865-010-9270-z | |
| 121 | | Individual and Neighbourhood-Level Socioeconomic Factors and Incidence of Type 2 Diabetes in Older Age: Results from a 14 Year Follow-up of a Cohort of Older British Men | | S. E. Ramsay; D. Roberts; A. O. Papacosta; L. T. Lennon; P. H. Whincup; S. G. Wannamethee | | J. Epidemiol. Community Health | | 2017 | | 71 | |  | |  | | 10.1136/jech-2017-SSMAbstracts.78 | |
| 122 | | Association between socioeconomic status and incident atrial fibrillation | | S. Ramkumar; A. Ochi; H. Yang; N. Nerlekar; N. D'Elia; E. L. Potter; I. C. Murray; N. Nattraj; Y. Wang; T. H. Marwick | | Intern Med J | | 2019 | | 49 | | 10 | | 1244-1251 | | 10.1111/imj.14214 | |
| 123 | | Incident Type 2 Diabetes Risk is Influenced by Obesity and Diabetes in Social Contacts: a Social Network Analysis | | S. Raghavan; M. C. Pachucki; Y. Chang; B. Porneala; C. S. Fox; J. Dupuis; J. B. Meigs | | J Gen Intern Med | | 2016 | | 31 | | 10 | | 1127-33 | | 10.1007/s11606-016-3723-1 | |
| 124 | | Neighbourhood disadvantage and self-reported type 2 diabetes, heart disease and comorbidity: a cross-sectional multilevel study | | J. N. Rachele; B. Giles-Corti; G. Turrell | | Ann Epidemiol | | 2016 | | 26 | | 2 | | 146-150 | | 10.1016/j.annepidem.2015.11.008 | |
| 125 | | Cardiovascular disease risk prediction equations in 400 000 primary care patients in New Zealand: a derivation and validation study | | R. Pylypchuk; S. Wells; A. Kerr; K. Poppe; T. Riddell; M. Harwood; D. Exeter; S. Mehta; C. Grey; B. P. Wu; P. Metcalf; J. Warren; J. Harrison; R. Marshall; R. Jackson | | Lancet | | 2018 | | 391 | |  | | 1897-1907 | | 10.1016/s0140-6736(18)30664-0 | |
| 126 | | Positive Psychosocial Factors in Childhood Predicting Lower Risk for Adult Type 2 Diabetes: The Cardiovascular Risk in Young Finns Study, 1980-2012 | | L. Pulkki-Råback; M. Elovainio; C. Hakulinen; J. Lipsanen; L. D. Kubzansky; M. Hintsanen; K. Savelieva; A. Serlachius; C. G. Magnussen; M. A. Sabin; D. P. Burgner; T. Lehtimäki; E. Jokinen; T. Rönnemaa; V. Mikkilä; A. Jula; N. Hutri-Kähönen; J. Viikari; L. Keltikangas-Järvinen; O. Raitakari; M. Juonala | | Am J Prev Med | | 2017 | | 52 | | 6 | |  | | 10.1016/j.amepre.2017.01.042 | |
| 127 | | Socioeconomic deprivation and the incidence of 12 cardiovascular diseases in 1.9 million women and men: implications for risk prediction and prevention | | M. Pujades-Rodriguez; A. Timmis; D. Stogiannis; E. Rapsomaniki; S. Denaxas; A. Shah; G. Feder; M. Kivimaki; H. Hemingway | | PLoS One | | 2014 | | 9 | | 8 | |  | | 10.1371/journal.pone.0104671 | |
| 128 | | Local Area Variation in Morbidity Among Low-Income, Older Adults in the United States: A Cross-sectional Study | | M. Polyakova; L. M. Hua | | Ann Intern Med | | 2019 | | 171 | | 7 | | 464-473 | | 10.7326/m18-2800 | |
| 129 | | Cumulative life course and adult socioeconomic status and markers of inflammation in adulthood | | R. A. Pollitt; J. S. Kaufman; K. M. Rose; A. V. Diez-Roux; D. Zeng; G. Heiss | | J Epidemiol Community Health | | 2008 | | 62 | | 6 | | 484-91 | | 10.1136/jech.2006.054106 | |
| 130 | | Early-life and adult socioeconomic status and inflammatory risk markers in adulthood | | R. A. Pollitt; J. S. Kaufman; K. M. Rose; A. V. Diez-Roux; D. Zeng; G. Heiss | | Eur J Epidemiol | | 2007 | | 22 | | 1 | | 55-66 | | 10.1007/s10654-006-9082-1 | |
| 131 | | Social networks and coronary heart disease risk factors in South Asians and Europeans in the UK | | T. M. Pollard; L. E. Carlin; R. Bhopal; N. Unwin; M. White; C. Fischbacher | | Ethn Health | | 2003 | | 8 | | 3 | | 263-75 | | 10.1080/1355785032000136452 | |
| 132 | | Neighborhood socioeconomic status and coronary heart disease risk prediction in a nationally representative sample | | C. E. Pollack; M. E. Slaughter; B. A. Griffin; T. Dubowitz; C. E. Bird | | Public Health | | 2012 | | 126 | | 10 | | 827-35 | | 10.1016/j.puhe.2012.05.028 | |
| 133 | | Multiple Vulnerabilities to Health Disparities and Incident Heart Failure Hospitalization in the REGARDS Study | | L. C. Pinheiro; E. Reshetnyak; M. R. Sterling; E. B. Levitan; M. M. Safford; P. Goyal | | Circ Cardiovasc Qual Outcomes | | 2020 | | 13 | | 8 | |  | | 10.1161/circoutcomes.119.006438 | |
| 134 | | Social support and ambulatory blood pressure: an examination of both receiving and giving | | R. L. Piferi; K. A. Lawler | | Int J Psychophysiol | | 2006 | | 62 | | 2 | | 328-36 | | 10.1016/j.ijpsycho.2006.06.002 | |
| 135 | | Comparing individual and area-based income measures: impact on analysis of inequality in smoking, obesity, and diabetes rates in Canadians 2003-2013 | | E. Pichora; J. Y. Polsky; C. Catley; N. Perumal; J. Jin; S. Allin | | Can. J. Public Health-Rev. Can. Sante Publ. | | 2018 | | 109 | | 3 | | 410-418 | | 10.17269/s41997-018-0062-5 | |
| 136 | | Associations of area based deprivation status and individual educational attainment with incidence, treatment, and prognosis of first coronary event in Rome, Italy | | S. Picciotto; F. Forastiere; M. Stafoggia; D. D'Ippoliti; C. Ancona; C. A. Perucci | | J Epidemiol Community Health | | 2006 | | 60 | | 1 | | 37-43 | | 10.1136/jech.2005.037846 | |
| 137 | | Social comparisons and health: can having richer friends and neighbors make you sick? | | G. Pham-Kanter | | Soc Sci Med | | 2009 | | 69 | | 3 | | 335-44 | | 10.1016/j.socscimed.2009.05.017 | |
| 138 | | Socioeconomic inequalities in coronary heart disease in Italy: a multilevel population-based study | | A. Petrelli; R. Gnavi; C. Marinacci; G. Costa | | Soc Sci Med | | 2006 | | 63 | | 2 | | 446-56 | | 10.1016/j.socscimed.2006.01.018 | |
| 139 | | Social network, social support, and loneliness in older persons with different chronic diseases | | B. W. Penninx; T. van Tilburg; D. M. Kriegsman; A. J. Boeke; D. J. Deeg; J. T. van Eijk | | J Aging Health | | 1999 | | 11 | | 2 | | 151-68 | | 10.1177/089826439901100202 | |
| 140 | | Psychosocial risk factors for the metabolic syndrome: A prospective cohort study | | J. M. Pedersen; R. Lund; I. Andersen; A. J. Clark; E. Prescott; N. H. Rod | | Int J Cardiol | | 2016 | | 215 | |  | | 41-6 | | 10.1016/j.ijcard.2016.04.076 | |
| 141 | | Association of Social and Behavioral Risk Factors With Earlier Onset of Adult Hypertension and Diabetes | | M. S. Pantell; A. A. Prather; J. M. Downing; N. P. Gordon; N. E. Adler | | JAMA Netw Open | | 2019 | | 2 | | 5 | |  | | 10.1001/jamanetworkopen.2019.3933 | |
| 142 | | Social determinants of health score: does it help identify those at higher cardiovascular risk? | | A. Palacio; R. Mansi; D. Seo; M. Suarez; S. Garay; H. Medina; F. Tang; L. Tamariz | | Am J Manag Care | | 2020 | | 26 | | 10 | |  | | 10.37765/ajmc.2020.88504 | |
| 143 | | Social relations and extent and severity of coronary artery disease. The Stockholm Female Coronary Risk Study | | K. Orth-Gomér; M. Horsten; S. P. Wamala; M. A. Mittleman; R. Kirkeeide; B. Svane; L. Rydén; K. Schenck-Gustafsson | | Eur Heart J | | 1998 | | 19 | | 11 | | 1648-56 | | 10.1053/euhj.1998.1190 | |
| 144 | | Neighbourhood immigrant concentration and hospitalization: a multilevel analysis of cardiovascular-related admissions in Ontario using linked data | | D. W. Omariba; N. A. Ross; C. Sanmartin; J. V. Tu | | Can J Public Health | | 2014 | | 105 | | 6 | |  | | 10.17269/cjph.105.4616 | |
| 145 | | Egocentric Health Networks and Cardiovascular Risk Factors in the ECHORN Cohort Study | | C. R. Oladele; T. A. Thompson; K. Wang; D. Galusha; E. Tran; J. L. Martinez-Brockman; O. P. Adams; R. G. Maharaj; C. M. Nazario; M. Nunez; M. Nunez-Smith | | J Gen Intern Med | | 2020 | | 35 | | 3 | | 784-791 | | 10.1007/s11606-019-05550-1 | |
| 146 | | Social determinants of hypertension and diabetes among African immigrants: the African immigrants health study | | O. Ogungbe; R. A. Turkson-Ocran; M. Nkimbeng; J. Cudjoe; H. N. Miller; D. Baptiste; C. D. Himmelfarb; P. Davidson; L. A. Cooper; Y. Commodore-Mensah | | Ethn Health | | 2021 | |  | |  | |  | | 10.1080/13557858.2021.1879026 | |
| 147 | | Sociodemographic Determinants of Acute Myocardial Infarction Hospitalization Risks in Florida | | E. W. Odoi; N. Nagle; R. Zaretzki; M. Jordan; C. DuClos; K. W. Kintziger | | J Am Heart Assoc | | 2020 | | 9 | | 11 | |  | | 10.1161/jaha.119.012712 | |
| 148 | | Role of support networks in maintenance of improved cardiovascular health status | | P. O'Reilly; H. E. Thomas | | Soc Sci Med | | 1989 | | 28 | | 3 | | 249-60 | | 10.1016/0277-9536(89)90268-2 | |
| 149 | | Inequalities and Deteriorations in Cardiovascular Health in Premenopausal US Women, 1990-2016 | | A. O'Neil; K. Thompson; J. D. Russell; R. Norton | | Am J Public Health | | 2020 | | 110 | | 8 | | 1175-1181 | | 10.2105/ajph.2020.305702 | |
| 150 | | Longitudinal analysis of large social networks: estimating the effect of health traits on changes in friendship ties | | A. J. O'Malley; N. A. Christakis | | Stat Med | | 2011 | | 30 | | 9 | | 950-64 | | 10.1002/sim.4190 | |
| 151 | | Neighborhood and individual socioeconomic status and early atherosclerosis: The Los Angeles atherosclerosis study | | C. K. Nordstrom; K. M. Dwyer; N. B. Merz; J. H. Dwyer | | Circulation | | 2002 | | 105 | | 14 | |  | |  | |
| 152 | | The association of personal and neighborhood socioeconomic indicators with subclinical cardiovascular disease in an elderly cohort. The cardiovascular health study | | C. K. Nordstrom; A. V. Diez Roux; S. A. Jackson; J. M. Gardin | | Soc Sci Med | | 2004 | | 59 | | 10 | | 2139-47 | | 10.1016/j.socscimed.2004.03.017 | |
| 153 | | Childhood social disadvantage, cardiometabolic risk, and chronic disease in adulthood | | A. L. Non; M. Rewak; I. Kawachi; S. E. Gilman; E. B. Loucks; A. A. Appleton; J. C. Román; S. L. Buka; L. D. Kubzansky | | Am J Epidemiol | | 2014 | | 180 | | 3 | | 263-71 | | 10.1093/aje/kwu127 | |
| 154 | | Social Network Structure and Atherosclerotic Cardiovascular Disease | | N. Nishi | | J Atheroscler Thromb | | 2018 | | 25 | | 2 | | 124-125 | | 10.5551/jat.ED087 | |
| 155 | | Do race, neglect, and childhood poverty predict physical health in adulthood? A multilevel prospective analysis | | V. Nikulina; C. S. Widom | | Child Abuse Negl | | 2014 | | 38 | | 3 | | 414-24 | | 10.1016/j.chiabu.2013.09.007 | |
| 156 | | Psychosocial work environment and cardiovascular risk factors in an occupational cohort in France | | I. Niedhammer; M. Goldberg; A. Leclerc; S. David; I. Bugel; M. F. Landre | | J Epidemiol Community Health | | 1998 | | 52 | | 2 | | 93-100 | | 10.1136/jech.52.2.93 | |
| 157 | | Socioeconomic Disadvantage Is Associated with a Higher Incidence of Aneurysmal Subarachnoid Hemorrhage | | L. Nichols; C. Stirling; P. Otahal; J. Stankovich; S. Gall | | J Stroke Cerebrovasc Dis | | 2018 | | 27 | | 3 | | 660-668 | | 10.1016/j.jstrokecerebrovasdis.2017.09.055 | |
| 158 | | Area-level socioeconomic characteristics, prevalence and trajectories of cardiometabolic risk | | A. D. Ngo; C. Paquet; N. J. Howard; N. T. Coffee; A. W. Taylor; R. J. Adams; M. Daniel | | Int J Environ Res Public Health | | 2014 | | 11 | | 1 | | 830-48 | | 10.3390/ijerph110100830 | |
| 159 | | Area-level socioeconomic characteristics and incidence of metabolic syndrome: a prospective cohort study | | A. D. Ngo; C. Paquet; N. J. Howard; N. T. Coffee; R. Adams; A. Taylor; M. Daniel | | BMC Public Health | | 2013 | | 13 | |  | |  | | 10.1186/1471-2458-13-681 | |
| 160 | | Trends of blood pressure levels and management in Västerbotten County, Sweden, during 1990-2010 | | N. Ng; B. Carlberg; L. Weinehall; M. Norberg | | Glob Health Action | | 2012 | | 5 | |  | |  | | 10.3402/gha.v5i0.18195 | |
| 161 | | Geographical variations in the prevalence of hypertension in France: Cross-sectional analysis of the CONSTANCES cohort | | L. Neufcourt; S. Deguen; S. Bayat; F. Paillard; M. Zins; O. Grimaud | | Eur J Prev Cardiol | | 2019 | | 26 | | 12 | | 1242-1251 | | 10.1177/2047487319842229 | |
| 162 | | An analytic approach for describing and prioritizing health inequalities at the local level in Canada: a descriptive study | | C. Neudorf; D. Fuller; J. Cushon; R. Glew; H. Turner; C. Ugolini | | CMAJ Open | | 2015 | | 3 | | 4 | |  | | 10.9778/cmajo.20150049 | |
| 163 | | Neighborhood social cohesion is associated with lower levels of interleukin-6 in African American women | | V. L. Neergheen; M. Topel; M. E. Van Dyke; S. Sullivan; P. E. Pemu; G. H. Gibbons; V. Vaccarino; A. A. Quyyumi; T. T. Lewis | | Brain Behav Immun | | 2019 | | 76 | |  | | 28-36 | | 10.1016/j.bbi.2018.10.008 | |
| 164 | | Educational Inequalities in Cardiovascular Risk Factor and Blood Pressure Control in the Elderly: Comparison of MESA Cohort and Chilean NHS Survey Outcome Measures | | C. Nazzal; S. Shea; C. Castro-Diehl; T. Alfaro; P. Frenz; C. J. Rodriguez | | Glob Heart | | 2018 | | 13 | | 1 | | 19-26 | | 10.1016/j.gheart.2017.09.001 | |
| 165 | | Cross-sectional and longitudinal associations of neighborhood characteristics with inflammatory markers: findings from the multi-ethnic study of atherosclerosis | | A. Nazmi; A. Diez Roux; N. Ranjit; T. E. Seeman; N. S. Jenny | | Health Place | | 2010 | | 16 | | 6 | | 1104-12 | | 10.1016/j.healthplace.2010.07.001 | |
| 166 | | Association Between Community-Level Social Participation and Self-reported Hypertension in Older Japanese: A JAGES Multilevel Cross-sectional Study | | A. Nakagomi; T. Tsuji; M. Hanazato; Y. Kobayashi; K. Kondo | | Am J Hypertens | | 2019 | | 32 | | 5 | | 503-514 | | 10.1093/ajh/hpz028 | |
| 167 | | Associations between area-level unemployment, body mass index, and risk factors for cardiovascular disease in an urban area | | A. I. Naimi; C. Paquet; L. Gauvin; M. Daniel | | Int J Environ Res Public Health | | 2009 | | 6 | | 12 | | 3082-96 | | 10.3390/ijerph6123082 | |
| 168 | | Social network, social support, and risk of incident stroke: Atherosclerosis Risk in Communities study | | M. Nagayoshi; S. A. Everson-Rose; H. Iso; T. H. Mosley, Jr.; K. M. Rose; P. L. Lutsey | | Stroke | | 2014 | | 45 | | 10 | | 2868-73 | | 10.1161/strokeaha.114.005815 | |
| 169 | | Trajectories of neighborhood poverty and associations with subclinical atherosclerosis and associated risk factors: the multi-ethnic study of atherosclerosis | | E. T. Murray; A. V. Diez Roux; M. Carnethon; P. L. Lutsey; H. Ni; E. S. O'Meara | | Am J Epidemiol | | 2010 | | 171 | | 10 | | 1099-108 | | 10.1093/aje/kwq044 | |
| 170 | | Neighborhood socioeconomic status in relation to dietary intake and insulin resistance syndrome in female Japanese dietetic students | | K. Murakami; S. Sasaki; Y. Takahashi; K. Uenishi | | Nutrition | | 2010 | | 26 | | 5 | | 508-14 | | 10.1016/j.nut.2009.08.025 | |
| 171 | | Regional and neighborhood disparities in the odds of type 2 diabetes: results from 5 population-based studies in Germany (DIAB-CORE consortium) | | G. Müller; A. Kluttig; K. H. Greiser; S. Moebus; U. Slomiany; S. Schipf; H. Völzke; W. Maier; C. Meisinger; T. Tamayo; W. Rathmann; K. Berger | | Am J Epidemiol | | 2013 | | 178 | | 2 | | 221-30 | | 10.1093/aje/kws466 | |
| 172 | | Gender differences in the association of individual social class and neighbourhood unemployment rate with prevalent type 2 diabetes mellitus: a cross-sectional study from the DIAB-CORE consortium | | G. Müller; S. Hartwig; K. H. Greiser; S. Moebus; N. Pundt; S. Schipf; H. Völzke; W. Maier; C. Meisinger; T. Tamayo; W. Rathmann; K. Berger | | BMJ Open | | 2013 | | 3 | | 6 | |  | | 10.1136/bmjopen-2013-002601 | |
| 173 | | [Neighbourhood deprivation and type 2 diabetes: results from the Dortmund Health Study (DHS)] | | G. Müller; K. Berger | | Gesundheitswesen | | 2013 | | 75 | | 12 | | 797-802 | | 10.1055/s-0033-1333737 | |
| 174 | | Neighborhood characteristics and hypertension | | M. S. Mujahid; A. V. Diez Roux; J. D. Morenoff; T. E. Raghunathan; R. S. Cooper; H. Ni; S. Shea | | Epidemiology | | 2008 | | 19 | | 4 | | 590-8 | | 10.1097/EDE.0b013e3181772cb2 | |
| 175 | | The influence of neighbourhood deprivation on the prevalence of diabetes in 25- to 74-year-old individuals: first results from the Dortmund Health Study | | G. Mueller; K. Berger | | Diabet Med | | 2012 | | 29 | | 6 | | 831-3 | | 10.1111/j.1464-5491.2011.03526.x | |
| 176 | | Neighborhood-level racial/ethnic residential segregation and incident cardiovascular disease: The multi-ethnic study of atherosclerosis Kershaw KN, Osypuk TL, Do DP, et al. Circulation 2015;131:141-8 | | E. A. Morse | | J. Emerg. Med. | | 2015 | | 49 | | 1 | |  | | 10.1016/j.jemermed.2015.05.020 | |
| 177 | | A multinomial model for comorbidity in England of long-standing cardiovascular disease, diabetes and obesity | | K. Morrissey; F. Espuny; P. Williamson | | Health Soc Care Community | | 2016 | | 24 | | 6 | | 717-727 | | 10.1111/hsc.12251 | |
| 178 | | Do socioeconomic characteristics of neighbourhood of residence independently influence incidence of coronary heart disease and all-cause mortality in older British men? | | R. W. Morris; G. Wannamethee; L. T. Lennon; M. C. Thomas; P. H. Whincup | | Eur J Cardiovasc Prev Rehabil | | 2008 | | 15 | | 1 | | 19-25 | | 10.1097/HJR.0b013e3282f11f81 | |
| 179 | | Socioeconomic Status and Long-Term Stroke Mortality, Recurrence and Disability in Iran: The Mashhad Stroke Incidence Study | | N. Morovatdar; A. G. Thrift; S. Stranges; M. Kapral; R. Behrouz; A. Amiri; A. Heshmati; A. Ghahremani; M. T. Farzadfard; N. Mokhber; M. R. Azarpazhooh | | Neuroepidemiology | | 2019 | | 53 | |  | | 27-31 | | 10.1159/000494885 | |
| 180 | | Understanding social disparities in hypertension prevalence, awareness, treatment, and control: the role of neighborhood context | | J. D. Morenoff; J. S. House; B. B. Hansen; D. R. Williams; G. A. Kaplan; H. E. Hunte | | Soc Sci Med | | 2007 | | 65 | | 9 | | 1853-66 | | 10.1016/j.socscimed.2007.05.038 | |
| 181 | | Is the status of diabetes socioeconomic inequality changing in Kurdistan province, west of Iran? A comparison of two surveys | | G. Moradi; R. Majdzadeh; K. Mohammad; H. Malekafzali; S. Jafari; K. Holakouie-Naieni | | Med J Islam Repub Iran | | 2016 | | 30 | |  | |  | |  | |
| 182 | | Decomposing social capital inequalities in health | | S. Moore; S. Stewart; A. Teixeira | | J Epidemiol Community Health | | 2014 | | 68 | | 3 | | 233-8 | | 10.1136/jech-2013-202996 | |
| 183 | | [Social status and cardiovascular risk factors in Danish males] | | L. F. Møller; T. S. Kristensen; H. Hollnagel | | Ugeskr Laeger | | 1991 | | 154 | | 1 | |  | |  | |
| 184 | | Social class and cardiovascular risk factors in Danish men | | L. Møller; T. S. Kristensen; H. Hollnagel | | Scand J Soc Med | | 1991 | | 19 | | 2 | | 116-26 | | 10.1177/140349489101900207 | |
| 185 | | Area-based socioeconomic status and mortality: the Ludwigshafen Risk and Cardiovascular Health study | | A. P. Moissl; G. E. Delgado; B. K. Krämer; W. März; M. E. Kleber; T. B. Grammer | | Clin Res Cardiol | | 2020 | | 109 | | 1 | | 103-114 | | 10.1007/s00392-019-01494-y | |
| 186 | | Environment, obesity, and cardiovascular disease risk in low-income women | | L. R. Mobley; E. D. Root; E. A. Finkelstein; O. Khavjou; R. P. Farris; J. C. Will | | Am J Prev Med | | 2006 | | 30 | | 4 | | 327-332 | | 10.1016/j.amepre.2005.12.001 | |
| 187 | | Personality type and coronary heart disease | | N. Mitaishvili; M. Danelia | | Georgian Med News | | 2006 | |  | |  | | 58-60 | |  | |
| 188 | | Depression, neighborhood deprivation and risk of type 2 diabetes | | B. Mezuk; Å. Chaikiat; X. Li; J. Sundquist; K. S. Kendler; K. Sundquist | | Health Place | | 2013 | | 23 | |  | | 63-9 | | 10.1016/j.healthplace.2013.05.004 | |
| 189 | | Immigrant enclaves and risk of diabetes: a prospective study | | B. Mezuk; K. Cederin; X. Li; K. Rice; K. S. Kendler; J. Sundquist; K. Sundquist | | BMC Public Health | | 2014 | | 14 | |  | |  | | 10.1186/1471-2458-14-1093 | |
| 190 | | Social network factors and cardiovascular health among baltimore public housing residents | | B. P. L. Meza; M. Chatrathi; C. E. Pollack; D. M. Levine; C. A. Latkin; J. M. Clark; L. A. Cooper; C. T. Yuan; N. M. Maruthur; K. A. Gudzune | | Prev Med Rep | | 2020 | | 20 | |  | |  | | 10.1016/j.pmedr.2020.101192 | |
| 191 | | Comparison of different markers of socioeconomic status with cardiovascular disease and diabetes risk factors in the Diabetes, Heart and Health Survey | | P. A. Metcalf; R. R. Scragg; D. Schaaf; L. Dyall; P. N. Black; R. T. Jackson | | N Z Med J | | 2008 | | 121 | |  | | 45-56 | |  | |
| 192 | | Diastolic blood pressure and area of residence: multilevel versus ecological analysis of social inequity | | J. Merlo; P. O. Ostergren; O. Hagberg; M. Lindström; A. Lindgren; A. Melander; L. Råstam; G. Berglund | | J Epidemiol Community Health | | 2001 | | 55 | | 11 | | 791-8 | | 10.1136/jech.55.11.791 | |
| 193 | | Revisiting causal neighborhood effects on individual ischemic heart disease risk: a quasi-experimental multilevel analysis among Swedish siblings | | J. Merlo; H. Ohlsson; B. Chaix; P. Lichtenstein; I. Kawachi; S. V. Subramanian | | Soc Sci Med | | 2013 | | 76 | | 1 | | 39-46 | | 10.1016/j.socscimed.2012.08.034 | |
| 194 | | Race/ethnicity, neighborhood socioeconomic status and cardio-metabolic risk | | S. S. Merkin; A. Karlamangla; A. D. Roux; S. Shrager; K. Watson; T. Seeman | | SSM Popul Health | | 2020 | | 11 | |  | |  | | 10.1016/j.ssmph.2020.100634 | |
| 195 | | Neighborhoods and cumulative biological risk profiles by race/ethnicity in a national sample of U.S. adults: NHANES III | | S. S. Merkin; R. Basurto-Dávila; A. Karlamangla; C. E. Bird; N. Lurie; J. Escarce; T. Seeman | | Ann Epidemiol | | 2009 | | 19 | | 3 | | 194-201 | | 10.1016/j.annepidem.2008.12.006 | |
| 196 | | Does the relationship between neighborhood socioeconomic status and health outcomes persist into very old age? A population-based study | | V. H. Menec; S. Shooshtari; S. Nowicki; S. Fournier | | J Aging Health | | 2010 | | 22 | | 1 | | 27-47 | | 10.1177/0898264309349029 | |
| 197 | | Community as a source of health in three racial/ethnic communities in Oregon: a qualitative study | | C. A. Mendez-Luck; J. W. Bethel; R. T. Goins; M. B. Schure; E. McDermott | | BMC Public Health | | 2015 | | 15 | |  | |  | | 10.1186/s12889-015-1462-6 | |
| 198 | | Blood Pressure Dipping and Urban Stressors in Young Adult African Americans | | T. A. Mellman; T. S. Brown; I. Kobayashi; S. H. Abu-Bader; J. Lavela; D. Altaee; L. McLaughlin; O. S. Randall | | Ann Behav Med | | 2015 | | 49 | | 4 | | 622-7 | | 10.1007/s12160-014-9684-x | |
| 199 | | The contribution of housing and neighbourhood conditions to educational inequalities in non-communicable diseases in Europe: findings from the European Social Survey (2014) special module on the social determinants of health | | C. L. McNamara; M. Balaj; K. H. Thomson; T. A. Eikemo; C. Bambra | | Eur J Public Health | | 2017 | | 27 | | suppl_1 | | 102-106 | | 10.1093/eurpub/ckw224 | |
| 200 | | Ideal cardiovascular health in urban Jamaica: prevalence estimates and relationship to community property value, household assets and educational attainment: a cross-sectional study | | J. A. McKenzie; N. O. Younger; M. K. Tulloch-Reid; I. Govia; N. R. Bennett; S. McFarlane; R. Walters; D. K. Francis; K. Webster-Kerr; A. Grant; T. Davidson; R. Wilks; D. R. Williams; T. S. Ferguson | | BMJ Open | | 2020 | | 10 | | 12 | |  | | 10.1136/bmjopen-2020-040664 | |
| 201 | | Late life socioeconomic status and hypertension in an aging cohort: the Atherosclerosis Risk in Communities Study | | M. M. McDoom; P. Palta; P. Vart; S. P. Juraschek; A. Kucharska-Newton; A. V. Diez Roux; J. Coresh | | J Hypertens | | 2018 | | 36 | | 6 | | 1382-1390 | | 10.1097/hjh.0000000000001696 | |
| 202 | | Association of Social Adversity with Comorbid Diabetes and Depression Symptoms in the Hispanic Community Health Study/Study of Latinos Sociocultural Ancillary Study: A Syndemic Framework | | J. L. McCurley; A. P. Gutierrez; J. I. Bravin; N. Schneiderman; S. A. Reina; T. Khambaty; S. F. Castañeda; S. Smoller; M. L. Daviglus; M. J. O'Brien; M. R. Carnethon; C. R. Isasi; K. M. Perreira; G. A. Talavera; M. Yang; L. C. Gallo | | Ann Behav Med | | 2019 | | 53 | | 11 | | 975-987 | | 10.1093/abm/kaz009 | |
| 203 | | Social Disadvantage and Social Isolation Are Associated With a Higher Resting Heart Rate: Evidence From The Irish Longitudinal Study on Ageing | | C. McCrory; C. Finucane; C. O'Hare; J. Frewen; H. Nolan; R. Layte; P. M. Kearney; R. A. Kenny | | J Gerontol B Psychol Sci Soc Sci | | 2016 | | 71 | | 3 | | 463-73 | | 10.1093/geronb/gbu163 | |
| 204 | | Longitudinal Associations of Neighborhood Crime and Perceived Safety With Blood Pressure: The Multi-Ethnic Study of Atherosclerosis (MESA) | | S. L. Mayne; K. A. Moore; T. M. Powell-Wiley; K. R. Evenson; R. Block; K. N. Kershaw | | Am J Hypertens | | 2018 | | 31 | | 9 | | 1024-1032 | | 10.1093/ajh/hpy066 | |
| 205 | | Racial residential segregation, racial discrimination, and diabetes: The Coronary Artery Risk Development in Young Adults study | | S. L. Mayne; L. Loizzo; M. P. Bancks; M. R. Carnethon; S. Barber; P. Gordon-Larsen; A. P. Carson; P. J. Schreiner; A. E. Bantle; K. M. Whitaker; K. N. Kershaw | | Health Place | | 2020 | | 62 | |  | |  | | 10.1016/j.healthplace.2020.102286 | |
| 206 | | Neighbourhood racial/ethnic residential segregation and cardiometabolic risk: the multiethnic study of atherosclerosis | | S. L. Mayne; M. T. Hicken; S. S. Merkin; T. E. Seeman; K. N. Kershaw; D. P. Do; A. Hajat; A. V. Diez Roux | | J Epidemiol Community Health | | 2019 | | 73 | | 1 | | 26-33 | | 10.1136/jech-2018-211159 | |
| 207 | | Income inequality and health outcomes in the United States: An empirical analysis | | P. Matthew; D. M. Brodersen | | Soc. Sci. J. | | 2018 | | 55 | | 4 | | 432-442 | | 10.1016/j.soscij.2018.05.001 | |
| 208 | | Investigating individual- and area-level socioeconomic gradients of pulse pressure among normotensive and hypertensive participants | | L. A. Matricciani; C. Paquet; N. J. Howard; R. Adams; N. T. Coffee; A. W. Taylor; M. Daniel | | Int J Environ Res Public Health | | 2013 | | 10 | | 2 | | 571-89 | | 10.3390/ijerph10020571 | |
| 209 | | Neighbourhood chronic stress and gender inequalities in hypertension among Canadian adults: a multilevel analysis | | F. I. Matheson; H. L. White; R. Moineddin; J. R. Dunn; R. H. Glazier | | J Epidemiol Community Health | | 2010 | | 64 | | 8 | | 705-13 | | 10.1136/jech.2008.083303 | |
| 210 | | Environmental factors and cardiovascular diseases: the association of income inequality and green spaces in elderly residents of São Paulo, Brazil | | K. H. Massa; R. Pabayo; M. L. Lebrão; A. D. Chiavegatto Filho | | BMJ Open | | 2016 | | 6 | | 9 | |  | | 10.1136/bmjopen-2016-011850 | |
| 211 | | [Hypertension in impoverished social segments in the state of São Paulo] | | I. S. Martins; D. C. de Oliveira; S. P. Marinho; E. A. de Araújo | | Cien Saude Colet | | 2008 | | 13 | | 2 | | 477-86 | | 10.1590/s1413-81232008000200023 | |
| 212 | | Neighborhood disadvantage across the transition from adolescence to adulthood and risk of metabolic syndrome | | C. L. Martin; J. B. Kane; G. L. Miles; A. E. Aiello; K. M. Harris | | Health Place | | 2019 | | 57 | |  | | 131-138 | | 10.1016/j.healthplace.2019.03.002 | |
| 213 | | A longitudinal study of structural risk factors for obesity and diabetes among American Indian young adults, 1994-2008 | | T. L. Marley; M. W. Metzger | | Prev Chronic Dis | | 2015 | | 12 | |  | |  | | 10.5888/pcd12.140469 | |
| 214 | | Social capital and hypertension in rural Haitian women | | C. Malino; T. Kershaw; M. Angley; R. Frederic; M. Small | | Matern Child Health J | | 2014 | | 18 | | 10 | | 2253-60 | | 10.1007/s10995-013-1351-5 | |
| 215 | | Social support, strain, and glycemic control: A path analysis | | K. G. Maki | | Pers. Relat. | | 2020 | | 27 | | 3 | | 592-612 | | 10.1111/pere.12333 | |
| 216 | | Socioeconomic deprivation, mortality and health of within-city migrants: A population cohort study | | R. Maheswaran; M. Strong; P. Clifford; L. Brewins | | J. Epidemiol. Community Health | | 2018 | | 72 | | 6 | | 519-525 | | 10.1136/jech-2017-210166 | |
| 217 | | Socio-economic deprivation and excess winter mortality and emergency hospital admissions in the South Yorkshire Coalfields Health Action Zone, UK | | R. Maheswaran; D. Chan; P. T. Fryers; C. McManus; H. McCabe | | Public Health | | 2004 | | 118 | | 3 | | 167-76 | | 10.1016/j.puhe.2003.09.004 | |
| 218 | | Ischaemic manifestations in giant cell arteritis are associated with area level socio-economic deprivation, but not cardiovascular risk factors | | S. L. Mackie; B. Dasgupta; L. Hordon; A. Gough; M. Green; J. Hollywood; S. Dutta; V. Bejarano; S. Jarrett; A. W. Morgan; C. T. Pease | | Rheumatology (Oxford) | | 2011 | | 50 | | 11 | | 2014-22 | | 10.1093/rheumatology/ker265 | |
| 219 | | Geographical and socioeconomic disparity analysis - An empirical study of hypertension and its comorbidities in China | | J. Ma; J. Wang; J. Liu; Z. Cao | | IEEE Computer Society | | 2016 | |  | |  | | 3318-3327 | | 10.1109/HICSS.2016.414 | |
| 220 | | Negative aspects of close social relations and 10-year incident ischaemic heart disease hospitalization among middle-aged Danes | | R. Lund; N. H. Rod; K. Thielen; C. J. Nilsson; U. Christensen | | Eur J Prev Cardiol | | 2014 | | 21 | | 10 | | 1249-56 | | 10.1177/2047487313486041 | |
| 221 | | Do Demands and Worries from Close Social Relations Increase the Risk of Subsequent Incident Ihd Hospitalization? A 7 Year Longitudinal Study of Middle-Aged Danish Men and Women | | R. Lund; N. H. Rod; K. Thielen; U. Christensen | | J. Epidemiol. Community Health | | 2012 | | 66 | |  | |  | | 10.1136/jech-2012-201753.059 | |
| 222 | | Are negative aspects of social relations predictive of angina pectoris? A 6-year follow-up study of middle-aged Danish women and men | | R. Lund; N. H. Rod; U. Christensen | | J Epidemiol Community Health | | 2012 | | 66 | | 4 | | 359-65 | | 10.1136/jech.2009.106153 | |
| 223 | | Sex differences in the association of social network satisfaction and the risk for type 2 diabetes | | K. Lukaschek; J. Baumert; J. Kruse; C. Meisinger; K. H. Ladwig | | BMC Public Health | | 2017 | | 17 | | 1 | |  | | 10.1186/s12889-017-4323-7 | |
| 224 | | Structural racism and myocardial infarction in the United States | | A. Lukachko; M. L. Hatzenbuehler; K. M. Keyes | | Soc Sci Med | | 2014 | | 103 | |  | | 42-50 | | 10.1016/j.socscimed.2013.07.021 | |
| 225 | | Neighborhoods, obesity, and diabetes--a randomized social experiment | | J. Ludwig; L. Sanbonmatsu; L. Gennetian; E. Adam; G. J. Duncan; L. F. Katz; R. C. Kessler; J. R. Kling; S. T. Lindau; R. C. Whitaker; T. W. McDade | | N Engl J Med | | 2011 | | 365 | | 16 | | 1509-19 | | 10.1056/NEJMsa1103216 | |
| 226 | | The Association Between Perceived Stress and Hypertension Among Asian Americans: Does Social Support and Social Network Make a Difference? | | X. Lu; H. S. Juon; X. He; C. M. Dallal; M. Q. Wang; S. Lee | | J Community Health | | 2019 | | 44 | | 3 | | 451-462 | | 10.1007/s10900-018-00612-7 | |
| 227 | | Evaluating options for measurement of neighborhood socioeconomic context: evidence from a myocardial infarction case-control study | | G. S. Lovasi; A. V. Moudon; N. L. Smith; T. Lumley; E. B. Larson; D. W. Sohn; D. S. Siscovick; B. M. Psaty | | Health Place | | 2008 | | 14 | | 3 | | 453-67 | | 10.1016/j.healthplace.2007.09.004 | |
| 228 | | Social networks and inflammatory markers in the Framingham Heart Study | | E. B. Loucks; L. M. Sullivan; R. B. D'Agostino, Sr.; M. G. Larson; L. F. Berkman; E. J. Benjamin | | J Biosoc Sci | | 2006 | | 38 | | 6 | | 835-42 | | 10.1017/s0021932005001203 | |
| 229 | | Relation of social integration to inflammatory marker concentrations in men and women 70 to 79 years | | E. B. Loucks; L. F. Berkman; T. L. Gruenewald; T. E. Seeman | | Am J Cardiol | | 2006 | | 97 | | 7 | | 1010-6 | | 10.1016/j.amjcard.2005.10.043 | |
| 230 | | Social integration is associated with fibrinogen concentration in elderly men | | E. B. Loucks; L. F. Berkman; T. L. Gruenewald; T. E. Seeman | | Psychosom Med | | 2005 | | 67 | | 3 | | 353-8 | | 10.1097/01.psy.0000160482.89163.e8 | |
| 231 | | Blood pressure and psychological distress among North Africans in France: The role of perceived personal/group discrimination and gender | | F. Loose; M. Tiboulet; C. Maisonneuve; A. Taillandier-Schmitt; M. Dambrun | | Am J Hum Biol | | 2017 | | 29 | | 5 | |  | | 10.1002/ajhb.23026 | |
| 232 | | Accumulated neighbourhood deprivation and coronary heart disease: a nationwide cohort study from Sweden | | S. L. Lönn; O. Melander; C. Crump; K. Sundquist | | BMJ Open | | 2019 | | 9 | | 9 | |  | | 10.1136/bmjopen-2019-029248 | |
| 233 | | Using multiple sources of data to assess the prevalence of diabetes at the subcounty level, Duval County, Florida, 2007 | | W. C. Livingood; L. Razaila; E. Reuter; R. Filipowicz; R. C. Butterfield; K. Lukens-Bull; L. Edwards; C. Palacio; D. L. Wood | | Prev Chronic Dis | | 2010 | | 7 | | 5 | |  | |  | |
| 234 | | Neighborhood environment and risk of ischemic stroke: the brain attack surveillance in Corpus Christi (BASIC) Project | | L. D. Lisabeth; A. V. Diez Roux; J. D. Escobar; M. A. Smith; L. B. Morgenstern | | Am J Epidemiol | | 2007 | | 165 | | 3 | | 279-87 | | 10.1093/aje/kwk005 | |
| 235 | | Associations of Continuity and Change in Early Neighborhood Poverty With Adult Cardiometabolic Biomarkers in the United States: Results From the National Longitudinal Study of Adolescent to Adult Health, 1995-2008 | | A. M. Lippert; C. R. Evans; F. Razak; S. V. Subramanian | | Am J Epidemiol | | 2017 | | 185 | | 9 | | 765-776 | | 10.1093/aje/kww206 | |
| 236 | | Do the Chinese Keep up with the Jones"?: Implications of peer effects, growing economic disparities and relative deprivation on health outcomes among older adults in China" | | D. C. Ling | | China Econ. Rev. | | 2009 | | 20 | | 1 | | 65-81 | | 10.1016/j.chieco.2008.10.010 | |
| 237 | | A Population-Based Approach to Mapping Vulnerability to Diabetes | | S. Linder; D. Marko; Y. Tian; T. Wisniewski | | Int J Environ Res Public Health | | 2018 | | 15 | | 10 | |  | | 10.3390/ijerph15102167 | |
| 238 | | Sex differences in social support, self-deception, hostility, and ambulatory cardiovascular activity | | W. Linden; L. Chambers; J. Maurice; J. W. Lenz | | Health Psychol | | 1993 | | 12 | | 5 | | 376-80 | | 10.1037//0278-6133.12.5.376 | |
| 239 | | Neighborhood characteristics, food deserts, rurality, and type 2 diabetes in youth: Findings from a case-control study | | A. D. Liese; A. P. Lamichhane; S. C. A. Garzia; R. C. Puett; D. E. Porter; D. Dabelea; R. B. D'Agostino, Jr.; D. Standiford; L. Liu | | Health Place | | 2018 | | 50 | |  | | 81-88 | | 10.1016/j.healthplace.2018.01.004 | |
| 240 | | Living in Cold Spot" Communities Is Associated with Poor Health and Health Quality" | | W. Liaw; A. H. Krist; S. T. Tong; R. Sabo; C. Hochheimer; J. Rankin; D. Grolling; J. Grandmont; A. W. Bazemore | | J Am Board Fam Med | | 2018 | | 31 | | 3 | | 342-350 | | 10.3122/jabfm.2018.03.170421 | |
| 241 | | Ethnic density, immigrant enclaves, and Latino health risks: A propensity score matching approach | | K. Li; M. Wen; K. A. Henry | | Soc Sci Med | | 2017 | | 189 | |  | | 44-52 | | 10.1016/j.socscimed.2017.07.019 | |
| 242 | | Neighborhood Racial Diversity and Metabolic Syndrome: 2003-2008 National Health and Nutrition Examination Survey | | K. Li; M. Wen; J. X. Fan | | J Immigr Minor Health | | 2019 | | 21 | | 1 | | 151-160 | | 10.1007/s10903-018-0728-3 | |
| 243 | | Socioeconomic gradients in the prevalence of cardiovascular disease in Scotland: the roles of composition and context | | A. H. Leyland | | J Epidemiol Community Health | | 2005 | | 59 | | 9 | | 799-803 | | 10.1136/jech.2005.034017 | |
| 244 | | Race, psychosocial factors, and aortic pulse wave velocity: the Health, Aging, and Body Composition Study | | T. T. Lewis; K. Sutton-Tyrrell; B. W. Penninx; N. Vogelzangs; T. B. Harris; G. D. Vaidean; H. N. Ayonayon; L. Kim; E. G. Lakatta; A. B. Newman | | J Gerontol A Biol Sci Med Sci | | 2010 | | 65 | | 10 | | 1079-85 | | 10.1093/gerona/glq089 | |
| 245 | | Health disparity by neighbourhood income | | M. Lemstra; C. Neudorf; J. Opondo | | Can J Public Health | | 2006 | | 97 | | 6 | | 435-9 | | 10.1007/bf03405223 | |
| 246 | | Life-course socioeconomic positions and subclinical atherosclerosis in the multi-ethnic study of atherosclerosis | | E. T. Lemelin; A. V. Diez Roux; T. G. Franklin; M. Carnethon; P. L. Lutsey; H. Ni; E. O'Meara; S. Shrager | | Soc Sci Med | | 2009 | | 68 | | 3 | | 444-51 | | 10.1016/j.socscimed.2008.10.038 | |
| 247 | | [Prevalence and factors associated with metabolic syndrome in users of primary healthcare units in São Paulo--SP, Brazil] | | M. P. Leitão; I. S. Martins | | Rev Assoc Med Bras (1992) | | 2012 | | 58 | | 1 | | 60-9 | |  | |
| 248 | | Biological embedding of neighborhood disadvantage and collective efficacy: Influences on chronic illness via accelerated cardiometabolic age | | M. K. Lei; S. R. H. Beach; R. L. Simons | | Dev Psychopathol | | 2018 | | 30 | | 5 | | 1797-1815 | | 10.1017/s0954579418000937 | |
| 249 | | Social support and networks: cardiovascular responses following recall on immigration stress among Chinese Americans | | Y. S. Lee; S. Suchday; J. Wylie-Rosett | | J Immigr Minor Health | | 2015 | | 17 | | 2 | | 543-52 | | 10.1007/s10903-013-9955-9 | |
| 250 | | Prevalence of Non-valvular Atrial Fibrillation Based on Geographical Distribution and Socioeconomic Status in the Entire Korean Population | | S. R. Lee; E. K. Choi; K. Han; M. J. Cha; S. Oh | | Korean Circ J | | 2018 | | 48 | | 7 | | 622-634 | | 10.4070/kcj.2017.0362 | |
| 251 | | Complex Role of Touch in Social Relationships for Older Adults' Cardiovascular Disease Risk | | J. E. Lee; K. E. Cichy | | Res Aging | | 2020 | | 42 | |  | | 208-216 | | 10.1177/0164027520915793 | |
| 252 | | Geographic Variation in Morbidity and Mortality of Cerebrovascular Diseases in Korea during 2011-2015 | | J. Lee; J. Bahk; I. Kim; Y. Y. Kim; S. C. Yun; H. Y. Kang; J. H. Park; S. A. Shin; Y. H. Khang | | J Stroke Cerebrovasc Dis | | 2018 | | 27 | | 3 | | 747-757 | | 10.1016/j.jstrokecerebrovasdis.2017.10.011 | |
| 253 | | Threats to security and ischaemic heart disease deaths: the case of homicides in Mexico | | E. H. Lee; T. A. Bruckner | | Int J Epidemiol | | 2017 | | 46 | | 1 | | 173-179 | | 10.1093/ije/dyw110 | |
| 254 | | Using Indirect Measures to Identify Geographic Hot Spots of Poor Glycemic Control: Cross-sectional Comparisons With an A1C Registry | | D. C. Lee; Q. Jiang; B. P. Tabaei; B. Elbel; C. A. Koziatek; K. J. Konty; W. Y. Wu | | Diabetes Care | | 2018 | | 41 | | 7 | | 1438-1447 | | 10.2337/dc18-0181 | |
| 255 | | Identifying Geographic Disparities in Diabetes Prevalence Among Adults and Children Using Emergency Claims Data | | D. C. Lee; M. P. Gallagher; A. Gopalan; M. Osorio; A. J. Vinson; S. P. Wall; J. E. Ravenell; M. A. Sevick; B. Elbel | | J Endocr Soc | | 2018 | | 2 | | 5 | | 460-470 | | 10.1210/js.2018-00001 | |
| 256 | | The Association between Social Network Betweenness and Coronary Calcification: A Baseline Study on Patients with a High Risk of Cardiovascular Disease | | C. J. Lee; W. T. Joo; J. Oh; I. C. Kim; S. H. Lee; S. M. Kang; H. C. Kim; S. Park; Y. Youm | | Atherosclerosis | | 2017 | | 263 | |  | |  | | 10.1016/j.atherosclerosis.2017.06.297 | |
| 257 | | Life-course socioeconomic position, area deprivation, and coronary heart disease: findings from the British Women's Heart and Health Study | | D. A. Lawlor; G. Davey Smith; R. Patel; S. Ebrahim | | Am J Public Health | | 2005 | | 95 | | 1 | | 91-7 | | 10.2105/ajph.2003.035592 | |
| 258 | | Social relations, depressive symptoms, and incident type 2 diabetes mellitus: The English Longitudinal Study of Ageing | | K. R. Laursen; A. Hulman; D. R. Witte; H. Terkildsen Maindal | | Diabetes Res Clin Pract | | 2017 | | 126 | |  | | 86-94 | | 10.1016/j.diabres.2017.01.006 | |
| 259 | | Socio-economic inequalities in the prevalence of Type 2 diabetes, cardiovascular risk factors and chronic diabetic complications in the Basque Country, Spain | | I. Larrañaga; J. M. Arteagoitia; J. L. Rodriguez; F. Gonzalez; S. Esnaola; J. A. Piniés | | Diabet Med | | 2005 | | 22 | | 8 | | 1047-53 | | 10.1111/j.1464-5491.2005.01598.x | |
| 260 | | Neighborhood Social Cohesion and Prevalence of Hypertension and Diabetes in a South Asian Population | | P. A. Lagisetty; M. Wen; H. Choi; M. Heisler; A. M. Kanaya; N. R. Kandula | | J Immigr Minor Health | | 2016 | | 18 | | 6 | | 1309-1316 | | 10.1007/s10903-015-0308-8 | |
| 261 | | Geographic Variations of Stroke Hospitalization across France: A Diachronic Cluster Analysis | | Y. Lachkhem; É. Minvielle; S. Rican | | Stroke Res Treat | | 2018 | | 2018 | |  | |  | | 10.1155/2018/1897569 | |
| 262 | | Socioeconomic status and incidence of type 2 diabetes: results from the Black Women's Health Study | | S. Krishnan; Y. C. Cozier; L. Rosenberg; J. R. Palmer | | Am J Epidemiol | | 2010 | | 171 | | 5 | | 564-70 | | 10.1093/aje/kwp443 | |
| 263 | | Overcoming the absence of socioeconomic data in medical records: validation and application of a census-based methodology | | N. Krieger | | Am J Public Health | | 1992 | | 82 | | 5 | | 703-10 | | 10.2105/ajph.82.5.703 | |
| 264 | | How is neighborhood social disorganization associated with diabetes outcomes? A multilevel investigation of glycemic control and self-reported use of acute or emergency health care services | | S. D. Kowitt; K. E. Donahue; E. B. Fisher; M. Mitchell; L. A. Young | | Clin Diabetes Endocrinol | | 2018 | | 4 | |  | |  | | 10.1186/s40842-018-0069-0 | |
| 265 | | Relationship between neighborhood socioeconomic status and venous thromboembolism: results from a population-based study | | D. Kort; N. van Rein; F. J. M. van der Meer; H. W. Vermaas; N. Wiersma; S. C. Cannegieter; W. M. Lijfering | | J Thromb Haemost | | 2017 | | 15 | | 12 | | 2352-2360 | | 10.1111/jth.13868 | |
| 266 | | Socioeconomic variation in incidence of primary and secondary major cardiovascular disease events: an Australian population-based prospective cohort study | | R. J. Korda; K. Soga; G. Joshy; B. Calabria; J. Attia; D. Wong; E. Banks | | Int J Equity Health | | 2016 | | 15 | | 1 | |  | | 10.1186/s12939-016-0471-0 | |
| 267 | | Social network and coronary artery calcification in asymptomatic individuals | | W. J. Kop; D. S. Berman; H. Gransar; N. D. Wong; R. Miranda-Peats; M. D. White; M. Shin; M. Bruce; D. S. Krantz; A. Rozanski | | Psychosom Med | | 2005 | | 67 | | 3 | | 343-52 | | 10.1097/01.psy.0000161201.45643.8d | |
| 268 | | Neighbourhood socioeconomic inequalities in incidence of acute myocardial infarction: a cohort study quantifying age- and gender-specific differences in relative and absolute terms | | C. Koopman; A. A. van Oeffelen; M. L. Bots; P. M. Engelfriet; W. M. Verschuren; L. van Rossem; I. van Dis; S. Capewell; I. Vaartjes | | BMC Public Health | | 2012 | | 12 | |  | |  | | 10.1186/1471-2458-12-617 | |
| 269 | | Exploring the social and neighbourhood predictors of diabetes: a comparison between Toronto and Chicago | | P. Kolpak; L. Wang | | Prim Health Care Res Dev | | 2017 | | 18 | | 3 | | 291-299 | | 10.1017/s1463423617000044 | |
| 270 | | Geographical clustering of incident acute myocardial infarction in Denmark: A spatial analysis approach | | T. M. Kjærulff; A. K. Ersbøll; G. Gislason; J. Schipperijn | | Spat Spatiotemporal Epidemiol | | 2016 | | 19 | |  | | 46-59 | | 10.1016/j.sste.2016.05.001 | |
| 271 | | Geographical inequalities in acute myocardial infarction beyond neighbourhood-level and individual-level sociodemographic characteristics: a Danish 10-year nationwide population-based cohort study | | T. M. Kjærulff; K. Bihrmann; I. Andersen; G. H. Gislason; M. L. Larsen; A. K. Ersbøll | | BMJ Open | | 2019 | | 9 | | 2 | |  | | 10.1136/bmjopen-2018-024207 | |
| 272 | | Neighbourhood socioeconomic disadvantage, risk factors, and diabetes from childhood to middle age in the Young Finns Study: a cohort study | | M. Kivimäki; J. Vahtera; A. G. Tabák; J. I. Halonen; P. Vineis; J. Pentti; K. Pahkala; S. Rovio; J. Viikari; M. Kähönen; M. Juonala; J. E. Ferrie; S. Stringhini; O. T. Raitakari | | Lancet Public Health | | 2018 | | 3 | | 8 | |  | | 10.1016/s2468-2667(18)30111-7 | |
| 273 | | Neighborhood context and social disparities in cumulative biological risk factors | | K. E. King; J. D. Morenoff; J. S. House | | Psychosom Med | | 2011 | | 73 | | 7 | | 572-9 | | 10.1097/PSY.0b013e318227b062 | |
| 274 | | Associations between social network properties and metabolic syndrome and the mediating effect of physical activity: findings from the Cardiovascular and Metabolic Diseases Etiology Research Center (CMERC) Cohort | | K. Kim; S. J. Jung; J. M. Baek; H. W. Yim; H. Jeong; D. J. Kim; S. Park; Y. Youm; H. C. Kim | | BMJ Open Diabetes Res Care | | 2020 | | 8 | | 1 | |  | | 10.1136/bmjdrc-2020-001272 | |
| 275 | | Identification of Resilient and At-Risk Neighborhoods for Cardiovascular Disease Among Black Residents: the Morehouse-Emory Cardiovascular (MECA) Center for Health Equity Study | | J. H. Kim; T. T. Lewis; M. L. Topel; M. Mubasher; C. Li; V. Vaccarino; M. S. Mujahid; M. Sims; A. A. Quyyumi; H. A. Taylor, Jr.; P. T. Baltrus | | Prev Chronic Dis | | 2019 | | 16 | |  | |  | | 10.5888/pcd16.180505 | |
| 276 | | Perceived neighborhood social cohesion and stroke | | E. S. Kim; N. Park; C. Peterson | | Soc Sci Med | | 2013 | | 97 | |  | | 49-55 | | 10.1016/j.socscimed.2013.08.001 | |
| 277 | | Perceived neighbourhood social cohesion and myocardial infarction | | E. S. Kim; A. M. Hawes; J. Smith | | J Epidemiol Community Health | | 2014 | | 68 | | 11 | | 1020-6 | | 10.1136/jech-2014-204009 | |
| 278 | | Neighbourhood socioeconomic position and risks of major chronic diseases and all-cause mortality: a quasi-experimental study | | D. Kim; R. H. Glazier; B. Zagorski; I. Kawachi; P. Oreopoulos | | BMJ Open | | 2018 | | 8 | | 5 | |  | | 10.1136/bmjopen-2017-018793 | |
| 279 | | Do neighborhood socioeconomic deprivation and low social cohesion predict coronary calcification?: the CARDIA study | | D. Kim; A. V. Diez Roux; C. I. Kiefe; I. Kawachi; K. Liu | | Am J Epidemiol | | 2010 | | 172 | | 3 | | 288-98 | | 10.1093/aje/kwq098 | |
| 280 | | Developing a data-driven spatial approach to assessment of neighbourhood influences on the spatial distribution of myocardial infarction | | W. Kihal-Talantikite; C. Weber; G. Pedrono; C. Segala; D. Arveiler; C. E. Sabel; S. Deguen; D. Bard | | Int J Health Geogr | | 2017 | | 16 | | 1 | |  | | 10.1186/s12942-017-0094-8 | |
| 281 | | Association of Changes in Neighborhood-Level Racial Residential Segregation With Changes in Blood Pressure Among Black Adults: The CARDIA Study | | K. N. Kershaw; W. R. Robinson; P. Gordon-Larsen; M. T. Hicken; D. C. Goff, Jr.; M. R. Carnethon; C. I. Kiefe; S. Sidney; A. V. Diez Roux | | JAMA Intern Med | | 2017 | | 177 | | 7 | | 996-1002 | | 10.1001/jamainternmed.2017.1226 | |
| 282 | | Neighborhood-level racial/ethnic residential segregation and incident cardiovascular disease: the multi-ethnic study of atherosclerosis | | K. N. Kershaw; T. L. Osypuk; D. P. Do; P. J. De Chavez; A. V. Diez Roux | | Circulation | | 2015 | | 131 | | 2 | | 141-8 | | 10.1161/circulationaha.114.011345 | |
| 283 | | Metropolitan-level racial residential segregation and black-white disparities in hypertension | | K. N. Kershaw; A. V. Diez Roux; S. A. Burgard; L. D. Lisabeth; M. S. Mujahid; A. J. Schulz | | Am J Epidemiol | | 2011 | | 174 | | 5 | | 537-45 | | 10.1093/aje/kwr116 | |
| 284 | | Enemies and friends in high-tech places: the development and validation of the Online Social Experiences Measure | | R. G. Kent de Grey; B. N. Uchino; B. R. Baucom; T. W. Smith; A. E. Holton; E. F. Diener | | Digit Health | | 2019 | | 5 | |  | |  | | 10.1177/2055207619878351 | |
| 285 | | Association Between Living in Food Deserts and Cardiovascular Risk | | H. M. Kelli; M. Hammadah; H. Ahmed; Y. A. Ko; M. Topel; A. Samman-Tahhan; M. Awad; K. Patel; K. Mohammed; L. S. Sperling; P. Pemu; V. Vaccarino; T. Lewis; H. Taylor; G. Martin; G. H. Gibbons; A. A. Quyyumi | | Circ Cardiovasc Qual Outcomes | | 2017 | | 10 | | 9 | |  | | 10.1161/circoutcomes.116.003532 | |
| 286 | | Associations of neighborhood area level deprivation with the metabolic syndrome and inflammation among middle- and older- age adults | | A. D. Keita; S. E. Judd; V. J. Howard; A. P. Carson; J. D. Ard; J. R. Fernandez | | BMC Public Health | | 2014 | | 14 | |  | |  | | 10.1186/1471-2458-14-1319 | |
| 287 | | A prospective study of social networks in relation to total mortality and cardiovascular disease in men in the USA | | I. Kawachi; G. A. Colditz; A. Ascherio; E. B. Rimm; E. Giovannucci; M. J. Stampfer; W. C. Willett | | J Epidemiol Community Health | | 1996 | | 50 | | 3 | | 245-51 | | 10.1136/jech.50.3.245 | |
| 288 | | Exploring the small-scale spatial distribution of hypertension and its association to area deprivation based on health insurance claims in Northeastern Germany | | B. Kauhl; W. Maier; J. Schweikart; A. Keste; M. Moskwyn | | BMC Public Health | | 2018 | | 18 | | 1 | |  | | 10.1186/s12889-017-5017-x | |
| 289 | | Blood pressure change in Africa: case study from Nigeria | | J. S. Kaufman; E. E. Owoaje; C. N. Rotimi; R. S. Cooper | | Hum Biol | | 1999 | | 71 | | 4 | | 641-57 | |  | |
| 290 | | Relationships among socioeconomic status, stress induced changes in cortisol, and blood pressure in African American males | | G. L. Kapuku; F. A. Treiber; H. C. Davis | | Ann Behav Med | | 2002 | | 24 | | 4 | | 320-5 | | 10.1207/s15324796abm2404_08 | |
| 291 | | Neighbourhood disadvantage and behavioural problems during childhood and the risk of cardiovascular disease risk factors and events from a prospective cohort | | L. Kakinami; L. A. Serbin; D. M. Stack; S. C. Karmaker; J. E. Ledingham; A. E. Schwartzman | | Prev Med Rep | | 2017 | | 8 | |  | | 294-300 | | 10.1016/j.pmedr.2017.10.003 | |
| 292 | | Neighborhood Environments and Incident Hypertension in the Multi-Ethnic Study of Atherosclerosis | | P. Kaiser; A. V. Diez Roux; M. Mujahid; M. Carnethon; A. Bertoni; S. D. Adar; S. Shea; R. McClelland; L. Lisabeth | | Am J Epidemiol | | 2016 | | 183 | | 11 | | 988-97 | | 10.1093/aje/kwv296 | |
| 293 | | The Australian Aboriginal Birth Cohort study: socio-economic status at birth and cardiovascular risk factors to 25 years of age | | M. Juonala; P. Sjöholm; K. Pahkala; S. Ellul; N. Kartiosuo; B. Davison; G. R. Singh | | Med J Aust | | 2019 | | 211 | | 6 | | 265-270 | | 10.5694/mja2.50285 | |
| 294 | | Childhood Psychosocial Factors and Coronary Artery Calcification in Adulthood: The Cardiovascular Risk in Young Finns Study | | M. Juonala; L. Pulkki-Råback; M. Elovainio; C. Hakulinen; C. G. Magnussen; M. A. Sabin; D. P. Burgner; D. L. Hare; O. Hartiala; H. Ukkonen; A. Saraste; S. Kajander; N. Hutri-Kähönen; M. Kähönen; I. Rinta-Kiikka; T. Laitinen; S. Kainulainen; J. S. Viikari; O. T. Raitakari | | JAMA Pediatr | | 2016 | | 170 | | 5 | | 466-72 | | 10.1001/jamapediatrics.2015.4121 | |
| 295 | | Nationally representative household survey data for studying the interaction between district-level development and individual-level socioeconomic gradients of cardiovascular disease risk factors in India | | L. Jung; J. W. De Neve; S. Chen; J. Manne-Goehler; L. M. Jaacks; D. J. Corsi; A. Awasthi; S. V. Subramanian; S. Vollmer; T. Bärnighausen; P. Geldsetzer | | Data Brief | | 2019 | | 27 | |  | |  | | 10.1016/j.dib.2019.104486 | |
| 296 | | The Role of Neighborhood Deprivation in Stroke Risk in Two Countries: France and the United States | | S. E. Judd; G. Howard; V. J. Howard; E. Z. Soliman; P. J. Clarke; N. Colabianchi; N. Letellier; C. Helmer; C. Feart; C. Tzourio; C. Berr; S. Lefevre; D. T. Lackland; C. Samieri | | Stroke | | 2019 | | 50 | |  | |  | |  | |
| 297 | | The Association between Social Network Betweenness and Coronary Calcium: A Baseline Study of Patients with a High Risk of Cardiovascular Disease | | W. T. Joo; C. J. Lee; J. Oh; I. C. Kim; S. H. Lee; S. M. Kang; H. C. Kim; S. Park; Y. Youm | | J Atheroscler Thromb | | 2018 | | 25 | | 2 | | 131-141 | | 10.5551/jat.40469 | |
| 298 | | Relationship between socioeconomic status and incidence of out-of-hospital cardiac arrest is dependent on age | | M. Jonsson; P. Ljungman; J. Härkönen; B. Van Nieuwenhuizen; S. Møller; M. Ringh; P. Nordberg | | J Epidemiol Community Health | | 2020 | | 74 | | 9 | | 726-731 | | 10.1136/jech-2019-213296 | |
| 299 | | Job strain, work place social support, and cardiovascular disease: a cross-sectional study of a random sample of the Swedish working population | | J. V. Johnson; E. M. Hall | | Am J Public Health | | 1988 | | 78 | | 10 | | 1336-42 | | 10.2105/ajph.78.10.1336 | |
| 300 | | Longitudinal associations of neighborhood socioeconomic status with cardiovascular risk factors: A 46-year follow-up study | | M. P. Jimenez; G. A. Wellenius; S. V. Subramanian; S. Buka; C. Eaton; S. E. Gilman; E. B. Loucks | | Soc Sci Med | | 2019 | | 241 | |  | |  | | 10.1016/j.socscimed.2019.112574 | |
| 301 | | Neighborhood characteristics and lifestyle intervention outcomes: Results from the Special Diabetes Program for Indians | | L. Jiang; J. Chang; J. Beals; A. Bullock; S. M. Manson | | Prev Med | | 2018 | | 111 | |  | | 216-224 | | 10.1016/j.ypmed.2018.03.009 | |
| 302 | | Social Identity Correlates of Minority Workers Health | | K. James; C. Lovato; G. Khoo | | Acad. Manage. J. | | 1994 | | 37 | | 2 | | 383-396 | | 10.2307/256834 | |
| 303 | | Residential mobility and chronic disease among World Trade Center Health Registry enrollees, 2004-2016 | | M. Jacobson; A. Crossa; S. Y. Liu; S. Locke; E. Poirot; C. Stein; S. Lim | | Health Place | | 2020 | | 61 | |  | |  | | 10.1016/j.healthplace.2019.102270 | |
| 304 | | Effects of race, sex, and socioeconomic status upon cardiovascular stress responsivity and recovery in youth | | R. W. Jackson; F. A. Treiber; J. R. Turner; H. Davis; W. B. Strong | | Int J Psychophysiol | | 1999 | | 31 | | 2 | | 111-9 | | 10.1016/s0167-8760(98)00044-0 | |
| 305 | | Studying place effects on health by synthesising individual and area-level outcomes | | C. H. Jackson; S. Richardson; N. G. Best | | Soc Sci Med | | 2008 | | 67 | | 12 | | 1995-2006 | | 10.1016/j.socscimed.2008.09.041 | |
| 306 | | Estimating the changing nature of Scotland's health inequalities by using a multivariate spatiotemporal model | | E. Jack; D. Lee; N. Dean | | J R Stat Soc Ser A Stat Soc | | 2019 | | 182 | | 3 | | 1061-1080 | | 10.1111/rssa.12447 | |
| 307 | | Neighborhood Characteristics and Ideal Cardiovascular Health Among Black Adults: Results From the Morehouse-Emory Cardiovascular (MECA) Center for Health Equity | | S. J. Islam; J. H. Kim; P. Baltrus; M. L. Topel; C. Liu; Y. A. Ko; M. S. Mujahid; V. Vaccarino; M. Sims; M. Mubasher; A. Khan; K. Ejaz; C. Searles; S. Dunbar; P. Pemu; H. A. Taylor; A. A. Quyyumi; T. T. Lewis | | Ann Epidemiol | | 2020 | |  | |  | |  | | 10.1016/j.annepidem.2020.11.009 | |
| 308 | | Association between social trust and the risk of cardiovascular disease in older adults in Korea: a nationwide retrospective cohort study | | S. E. Hwang; S. Choi; K. Kim; J. K. Lee; J. Oh; S. M. Park | | BMC Public Health | | 2020 | | 20 | | 1 | |  | | 10.1186/s12889-020-09964-z | |
| 309 | | Unequal Exposure or Unequal Vulnerability? Contributions of Neighborhood Conditions and Cardiovascular Risk Factors to Socioeconomic Inequality in Incident Cardiovascular Disease in the Multi-Ethnic Study of Atherosclerosis | | M. Hussein; A. V. Diez Roux; M. S. Mujahid; T. A. Hastert; K. N. Kershaw; A. G. Bertoni; A. Baylin | | Am J Epidemiol | | 2018 | | 187 | | 7 | | 1424-1437 | | 10.1093/aje/kwx363 | |
| 310 | | Social support reduces resting cardiovascular function in women | | B. M. Hughes; S. Howard | | Anxiety Stress Coping | | 2009 | | 22 | | 5 | | 537-48 | | 10.1080/10615800902814614 | |
| 311 | | Social support in ordinary life and laboratory measures of cardiovascular reactivity: gender differences in habituation-sensitization | | B. M. Hughes | | Ann Behav Med | | 2007 | | 34 | | 2 | | 166-76 | | 10.1007/bf02872671 | |
| 312 | | Quality and quantity of social support as differential predictors of cardiovascular reactivity | | B. Hughes; R. Curtis | | Ir. J. Psychol. | | 2000 | | 21 | |  | | 16-31 | | 10.1080/03033910.2000.10558237 | |
| 313 | | Neighborhood Deprivation, Obesity, and Diabetes in Residents of the US Gulf Coast | | M. D. Hu; K. G. Lawrence; M. R. Bodkin; R. K. Kwok; L. S. Engel; D. P. Sandler | | Am J Epidemiol | | 2021 | | 190 | | 2 | | 295-304 | | 10.1093/aje/kwaa206 | |
| 314 | | Quantile Regression Forests to Identify Determinants of Neighborhood Stroke Prevalence in 500 Cities in the USA: Implications for Neighborhoods with High Prevalence | | L. Hu; J. Ji; Y. Li; B. Liu; Y. Zhang | | J Urban Health | | 2020 | |  | |  | |  | | 10.1007/s11524-020-00478-y | |
| 315 | | Neighborhood socioeconomic index and stroke incidence in a national cohort of blacks and whites | | V. J. Howard; L. A. McClure; D. O. Kleindorfer; S. A. Cunningham; A. G. Thrift; A. V. Diez Roux; G. Howard | | Neurology | | 2016 | | 87 | | 22 | | 2340-2347 | | 10.1212/wnl.0000000000003299 | |
| 316 | | Social connections and hypertension in women and men: a population-based cross-sectional study of the Canadian Longitudinal Study on Aging | | Z. Hosseini; G. Veenstra; N. A. Khan; A. I. Conklin | | J Hypertens | | 2020 | |  | |  | |  | | 10.1097/hjh.0000000000002688 | |
| 317 | | Social relations and the metabolic syndrome in middle-aged Swedish women | | M. Horsten; M. A. Mittleman; S. P. Wamala; K. Schenck-Gustafsson; K. Orth-Gomér | | J Cardiovasc Risk | | 1999 | | 6 | | 6 | | 391-7 | | 10.1177/204748739900600606 | |
| 318 | | Impact of neighborhood socioeconomic conditions on the risk of stroke in Japan | | K. Honjo; H. Iso; T. Nakaya; T. Hanibuchi; A. Ikeda; M. Inoue; N. Sawada; S. Tsugane | | J Epidemiol | | 2015 | | 25 | | 3 | | 254-60 | | 10.2188/jea.JE20140117 | |
| 319 | | Development and Validation of a County-Level Social Determinants of Health Risk Assessment Tool for Cardiovascular Disease | | Y. R. Hong; A. G. Mainous, 3rd | | Ann Fam Med | | 2020 | | 18 | | 4 | | 318-325 | | 10.1370/afm.2534 | |
| 320 | | Is social capital a protective factor against obesity and diabetes? Findings from an exploratory study | | D. R. Holtgrave; R. Crosby | | Ann Epidemiol | | 2006 | | 16 | | 5 | | 406-8 | | 10.1016/j.annepidem.2005.04.017 | |
| 321 | | Social relationships and ambulatory blood pressure: structural and qualitative predictors of cardiovascular function during everyday social interactions | | J. Holt-Lunstad; B. N. Uchino; T. W. Smith; C. Olson-Cerny; J. B. Nealey-Moore | | Health Psychol | | 2003 | | 22 | | 4 | | 388-97 | | 10.1037/0278-6133.22.4.388 | |
| 322 | | Higher prevalence of heart failure in rural regions: a population-based study covering 87% of German inhabitants | | J. Holstiege; M. K. Akmatov; S. Störk; A. Steffen; J. Bätzing | | Clin Res Cardiol | | 2019 | | 108 | | 10 | | 1102-1106 | | 10.1007/s00392-019-01444-8 | |
| 323 | | Neighborhoods and systemic inflammation: High CRP among legal and unauthorized Brazilian migrants | | L. M. Holmes; E. A. Marcelli | | Health Place | | 2012 | | 18 | | 3 | | 683-693 | | 10.1016/j.healthplace.2011.11.006 | |
| 324 | | Is income area level associated with blood pressure in adults regardless of individual-level characteristics? A multilevel approach | | D. A. Höfelmann; J. L. Antunes; D. A. Santos Silva; M. A. Peres | | Health Place | | 2012 | | 18 | | 5 | | 971-7 | | 10.1016/j.healthplace.2012.06.010 | |
| 325 | | Spatial analysis and correlates of county-level diabetes prevalence, 2009-2010 | | J. A. Hipp; N. Chalise | | Prev Chronic Dis | | 2015 | | 12 | |  | |  | | 10.5888/pcd12.140404 | |
| 326 | | Connecting social environment variables to the onset of major specific health outcomes | | P. L. Hill; S. J. Weston; J. J. Jackson | | Psychol Health | | 2014 | | 29 | | 7 | | 753-67 | | 10.1080/08870446.2014.884221 | |
| 327 | | Social network and development of prediabetes and type 2 diabetes in middle-aged Swedish women and men | | A. Hilding; C. Shen; C. G. Östenson | | Diabetes Res Clin Pract | | 2015 | | 107 | | 1 | | 166-77 | | 10.1016/j.diabres.2014.09.057 | |
| 328 | | Socioeconomic position is positively associated with blood pressure dipping among African-American adults: the Jackson Heart Study | | D. A. Hickson; A. V. Diez Roux; S. B. Wyatt; S. Y. Gebreab; G. Ogedegbe; D. F. Sarpong; H. A. Taylor; M. R. Wofford | | Am J Hypertens | | 2011 | | 24 | | 9 | | 1015-21 | | 10.1038/ajh.2011.98 | |
| 329 | | Implications of supermarket access, neighbourhood walkability and poverty rates for diabetes risk in an employee population | | C. J. Herrick; B. W. Yount; A. A. Eyler | | Public Health Nutr | | 2016 | | 19 | | 11 | | 2040-8 | | 10.1017/s1368980015003328 | |
| 330 | | Association between social determinants of health and trends in prevalence of hypertension in patients of the Peruvian Ministry of Health | | P. Herrera-Añazco; E. Amaya; N. Atamari-Anahui; M. Ccorahua-Rios; A. V. Hernandez | | Trop Med Int Health | | 2019 | | 24 | | 12 | | 1434-1441 | | 10.1111/tmi.13318 | |
| 331 | | Structural social support and cardiovascular disease risk factors in Hispanic/Latino adults with diabetes: results from the Hispanic Community Health Study/Study of Latinos (HCHS/SOL) | | R. Hernandez; M. Carnethon; A. L. Giachello; F. J. Penedo; D. Wu; O. Birnbaum-Weitzman; R. E. Giacinto; L. C. Gallo; C. R. Isasi; N. Schneiderman; Y. Teng; D. Zeng; M. L. Daviglus | | Ethn Health | | 2018 | | 23 | | 7 | | 737-751 | | 10.1080/13557858.2017.1294660 | |
| 332 | | Relationship between psychosocial aspects of work environment and cardiovascular heart risk in men | | D. Hernandez; A. Salazar; V. Gomez | | Rev. Latinoam. Psicol. | | 2004 | | 36 | | 1 | | 107-123 | |  | |
| 333 | | Associations between income inequality at municipality level and health depend on context - a multilevel analysis on myocardial infarction in Sweden | | G. Henriksson; G. R. Weitoft; P. Allebeck | | Soc Sci Med | | 2010 | | 71 | | 6 | | 1141-9 | | 10.1016/j.socscimed.2010.05.044 | |
| 334 | | Identification of direct and indirect social network effects in the pathophysiology of insulin resistance in obese human subjects | | C. H. Henning; N. Zarnekow; J. Hedtrich; S. Stark; K. Türk; M. Laudes | | PLoS One | | 2014 | | 9 | | 4 | |  | | 10.1371/journal.pone.0093860 | |
| 335 | | Social Relationships and Risk of Type 2 Diabetes Among Postmenopausal Women | | M. Hendryx; W. Nicholson; J. E. Manson; C. H. Kroenke; J. Lee; J. C. Weitlauf; L. Garcia; J. M. Jonasson; J. Wactawski-Wende; J. Luo | | J Gerontol B Psychol Sci Soc Sci | | 2020 | | 75 | | 7 | | 1597-1608 | | 10.1093/geronb/gbz047 | |
| 336 | | Relationship between social network, social support and health behaviour in people with type 1 and type 2 diabetes: cross-sectional studies | | N. F. Hempler; L. E. Joensen; I. Willaing | | BMC Public Health | | 2016 | | 16 | |  | |  | | 10.1186/s12889-016-2819-1 | |
| 337 | | Social network in relation to plasma fibrinogen | | A. Helminen; T. Rankinen; S. Väisänen; R. Rauramaa | | J Biosoc Sci | | 1997 | | 29 | | 2 | | 129-39 | | 10.1017/s0021932097001296 | |
| 338 | | Carotid atherosclerosis in middle-aged men. Relation to conjugal circumstances and social support | | A. Helminen; T. Rankinen; M. Mercuri; R. Rauramaa | | Scand J Soc Med | | 1995 | | 23 | | 3 | | 167-72 | | 10.1177/140349489502300306 | |
| 339 | | Validity assessment of a social support index | | A. Helminen; P. Halonen; T. Rankinen; A. Nissinen; R. Rauramaa | | Scand J Soc Med | | 1995 | | 23 | | 1 | | 66-74 | | 10.1177/140349489502300112 | |
| 340 | | Social inequities in cardiovascular disease risk factors in East and West Germany | | U. Helmert; A. Mielck; E. Classen | | Soc Sci Med | | 1992 | | 35 | | 10 | | 1283-92 | | 10.1016/0277-9536(92)90181-o | |
| 341 | | Social group and risk factors for coronary heart diseases: Results of a health survey in Western Germany | | U. Helmert; E. Greiser | | Soz Präventivmed | | 1988 | | 33 | |  | | 233-240 | | 10.1007/BF02083579 | |
| 342 | | Social network composition of vascular patients and its associations with health behavior and clinical risk factors | | N. Heijmans; J. van Lieshout; M. Wensing | | PLoS One | | 2017 | | 12 | | 9 | |  | | 10.1371/journal.pone.0185341 | |
| 343 | | Socioeconomic disparities in stroke rates and outcome: pooled analysis of stroke incidence studies in Australia and New Zealand | | E. L. Heeley; J. W. Wei; K. Carter; M. S. Islam; A. G. Thrift; G. J. Hankey; A. Cass; C. S. Anderson | | Med J Aust | | 2011 | | 195 | | 1 | |  | | 10.5694/j.1326-5377.2011.tb03180.x | |
| 344 | | Influence of social support on cardiac event rate in men with ischaemic type ST segment depression during ambulatory 24-h long-term ECG recording. The prospective population study 'Men born in 1914', Malmö, Sweden | | B. Hedblad; P. O. Ostergren; B. S. Hanson; L. Janzon; B. W. Johansson; S. Juul-Möller | | Eur Heart J | | 1992 | | 13 | | 4 | | 433-9 | | 10.1093/oxfordjournals.eurheartj.a060193 | |
| 345 | | Individual and Neighborhood Stressors, Air Pollution and Cardiovascular Disease | | M. F. Hazlehurst; P. S. Nurius; A. Hajat | | Int J Environ Res Public Health | | 2018 | | 15 | | 3 | |  | | 10.3390/ijerph15030472 | |
| 346 | | Community care in England: reducing socioeconomic inequalities in heart failure | | N. M. Hawkins; S. Scholes; M. Bajekal; H. Love; M. O'Flaherty; R. Raine; S. Capewell | | Circulation | | 2012 | | 126 | | 9 | | 1050-7 | | 10.1161/circulationaha.111.088047 | |
| 347 | | Socioeconomic and behavioral determinants of cardiovascular diseases among older adults in Belgium and France: A longitudinal analysis from the SHARE study | | H. Y. Hassen; H. Bastiaens; K. Van Royen; S. Abrams | | PLoS One | | 2020 | | 15 | | 12 | |  | | 10.1371/journal.pone.0243422 | |
| 348 | | People, places and coronary heart disease risk factors: a multilevel analysis of the Scottish Heart Health Study archive | | C. Hart; R. Ecob; G. D. Smith | | Soc Sci Med | | 1997 | | 45 | | 6 | | 893-902 | | 10.1016/s0277-9536(96)00431-5 | |
| 349 | | Myocardial infarction and heart failure hospitalization rates in Maine, USA - variability along the urban-rural continuum | | D. E. Harris; A. M. Aboueissa; D. Hartley | | Rural Remote Health | | 2008 | | 8 | | 2 | |  | |  | |
| 350 | | Social anchorage and blood pressure in elderly men--a population study | | B. S. Hanson; S. O. Isacsson; L. Janzon; S. E. Lindell; L. Råstam | | J Hypertens | | 1988 | | 6 | | 6 | | 503-10 | | 10.1097/00004872-198806000-00011 | |
| 351 | | Relationship between county-level crime and diabetes: Mediating effect of physical inactivity | | M. Hanigan; M. Heisler; H. Choi | | Prev Med Rep | | 2020 | | 20 | |  | |  | | 10.1016/j.pmedr.2020.101220 | |
| 352 | | Impact of scale of aggregation on associations of cardiovascular hospitalization and socio-economic disadvantage | | I. C. Hanigan; T. Cochrane; R. Davey | | PLoS One | | 2017 | | 12 | | 11 | |  | | 10.1371/journal.pone.0188161 | |
| 353 | | Social Gradients in Myocardial Infarction and Stroke Diagnoses in Emergency Medicine | | C. Hanefeld; A. Haschemi; T. Lampert; H. J. Trampisch; A. Mügge; J. Miebach; C. Kloppe; R. Klaaßen-Mielke | | Dtsch Arztebl Int | | 2018 | | 115 | | 4 | | 41-48 | | 10.3238/arztebl.2018.0041 | |
| 354 | | Social Activities, Incident Cardiovascular Disease, and Mortality | | S. H. Han; J. L. Tavares; M. Evans; J. Saczynski; J. A. Burr | | J Aging Health | | 2017 | | 29 | | 2 | | 268-288 | | 10.1177/0898264316635565 | |
| 355 | | Job strain, social support at work, and incidence of myocardial infarction | | N. Hammar; L. Alfredsson; J. V. Johnson | | Occup Environ Med | | 1998 | | 55 | | 8 | | 548-53 | | 10.1136/oem.55.8.548 | |
| 356 | | Association of Neighborhood Disadvantage With Cardiovascular Risk Factors and Events Among Refugees in Denmark | | R. Hamad; B. Öztürk; E. Foverskov; L. Pedersen; H. T. Sørensen; H. E. Bøtker; J. S. White | | JAMA Netw Open | | 2020 | | 3 | | 8 | |  | | 10.1001/jamanetworkopen.2020.14196 | |
| 357 | | Childhood Psychosocial Adversity and Adult Neighborhood Disadvantage as Predictors of Cardiovascular Disease: A Cohort Study | | J. I. Halonen; S. Stenholm; J. Pentti; I. Kawachi; S. V. Subramanian; M. Kivimäki; J. Vahtera | | Circulation | | 2015 | | 132 | | 5 | | 371-9 | | 10.1161/circulationaha.115.015392 | |
| 358 | | Work life, relationship, and policy determinants of health and well-being among Filipino domestic Workers in China: a qualitative study | | B. J. Hall; M. R. Garabiles; C. A. Latkin | | BMC Public Health | | 2019 | | 19 | | 1 | |  | | 10.1186/s12889-019-6552-4 | |
| 359 | | Community-level socioeconomic inequality in the incidence of ischemic heart disease: a nationwide cohort study | | J. G. Gwon; J. Choi; Y. J. Han | | BMC Cardiovasc Disord | | 2020 | | 20 | | 1 | |  | | 10.1186/s12872-020-01389-1 | |
| 360 | | Do peer relations in adolescence influence health in adulthood? Peer problems in the school setting and the metabolic syndrome in middle-age | | P. E. Gustafsson; U. Janlert; T. Theorell; H. Westerlund; A. Hammarström | | PLoS One | | 2012 | | 7 | | 6 | |  | | 10.1371/journal.pone.0039385 | |
| 361 | | Area deprivation and the prevalence of type 2 diabetes and obesity: analysis at the municipality level in Germany | | N. Grundmann; A. Mielck; M. Siegel; W. Maier | | BMC Public Health | | 2014 | | 14 | |  | |  | | 10.1186/1471-2458-14-1264 | |
| 362 | | Association of social relationships with incident cardiovascular events and all-cause mortality | | J. Gronewold; R. Kropp; N. Lehmann; B. Schmidt; S. Weyers; J. Siegrist; N. Dragano; K. H. Jöckel; R. Erbel; D. M. Hermann | | Heart | | 2020 | | 106 | | 17 | | 1317-1323 | | 10.1136/heartjnl-2019-316250 | |
| 363 | | Gender differences in the association between socioeconomic status and subclinical atherosclerosis | | O. Grimaud; A. Lapostolle; C. Berr; C. Helmer; C. Dufouil; W. Kihal; A. Alpérovitch; P. Chauvin | | PLoS One | | 2013 | | 8 | | 11 | |  | | 10.1371/journal.pone.0080195 | |
| 364 | | Incidence of stroke and socioeconomic neighborhood characteristics: an ecological analysis of Dijon stroke registry | | O. Grimaud; Y. Béjot; Z. Heritage; J. Vallée; J. Durier; E. Cadot; M. Giroud; P. Chauvin | | Stroke | | 2011 | | 42 | | 5 | | 1201-6 | | 10.1161/strokeaha.110.596429 | |
| 365 | | Social vulnerability and its possible relation to the principal causes of morbidity and mortality in the Mexican state of Oaxaca | | A. M. González-Villoria; R. A. Abeldaño Zuñiga | | Int J Equity Health | | 2018 | | 17 | | 1 | |  | | 10.1186/s12939-018-0849-2 | |
| 366 | | All in the family: The link between kin network bridging and cardiovascular risk among older adults | | A. W. Goldman | | Soc Sci Med | | 2016 | | 166 | |  | | 137-149 | | 10.1016/j.socscimed.2016.07.035 | |
| 367 | | Sex differences in the association of psychosocial resources with prevalent type 2 diabetes among African Americans: The Jackson Heart Study | | L. M. Glover; A. G. Bertoni; S. H. Golden; P. Baltrus; Y. I. Min; M. R. Carnethon; H. Taylor; M. Sims | | J Diabetes Complications | | 2019 | | 33 | | 2 | | 113-117 | | 10.1016/j.jdiacomp.2018.11.005 | |
| 368 | | Neighborhood social and physical environments and type 2 diabetes mellitus in African Americans: The Jackson Heart Study | | S. Y. Gebreab; D. A. Hickson; M. Sims; S. B. Wyatt; S. K. Davis; A. Correa; A. V. Diez-Roux | | Health Place | | 2017 | | 43 | |  | | 128-137 | | 10.1016/j.healthplace.2016.12.001 | |
| 369 | | Geographic variations in cardiovascular health in the United States: contributions of state- and individual-level factors | | S. Y. Gebreab; S. K. Davis; J. Symanzik; G. A. Mensah; G. H. Gibbons; A. V. Diez-Roux | | J Am Heart Assoc | | 2015 | | 4 | | 6 | |  | | 10.1161/jaha.114.001673 | |
| 370 | | Disparities in diabetes: the nexus of race, poverty, and place | | D. J. Gaskin; R. J. Thorpe, Jr.; E. E. McGinty; K. Bower; C. Rohde; J. H. Young; T. A. LaVeist; L. Dubay | | Am J Public Health | | 2014 | | 104 | | 11 | | 2147-55 | | 10.2105/ajph.2013.301420 | |
| 371 | | Changes in perceptions of neighborhood environment and Cardiometabolic outcomes in two predominantly African American neighborhoods | | T. L. Gary-Webb; N. S. Egnot; A. Nugroho; T. Dubowitz; W. M. Troxel | | BMC Public Health | | 2020 | | 20 | | 1 | |  | | 10.1186/s12889-019-8119-9 | |
| 372 | | Associations of anger, vital exhaustion, anti-depressant use, and poor social ties with incident atrial fibrillation: The Atherosclerosis Risk in Communities Study | | P. K. Garg; J. S. Claxton; E. Z. Soliman; L. Y. Chen; T. T. Lewis; T. Mosley, Jr.; A. Alonso | | Eur J Prev Cardiol | | 2020 | |  | |  | |  | | 10.1177/2047487319897163 | |
| 373 | | Influence of neighbourhood socioeconomic position on the transition to type II diabetes in older Mexican Americans: the Sacramento Area Longitudinal Study on Aging | | L. Garcia; A. Lee; A. Zeki Al Hazzouri; J. M. Neuhaus; S. Moyce; A. Aiello; T. Elfassy; M. N. Haan | | BMJ Open | | 2016 | | 6 | | 8 | |  | | 10.1136/bmjopen-2015-010905 | |
| 374 | | The Impact of Neighborhood Socioeconomic Position on Prevalence of Diabetes and Prediabetes in Older Latinos: The Sacramento Area Latino Study on Aging | | L. Garcia; A. Lee; A. Zeki Al Hazzouri; J. Neuhaus; M. Epstein; M. Haan | | Hisp Health Care Int | | 2015 | | 13 | | 2 | | 77-85 | | 10.1891/1540-4153.13.2.77 | |
| 375 | | Measuring the habitat as an indicator of socioeconomic position: methodology and its association with hypertension | | B. Galobardes; A. Morabia | | J Epidemiol Community Health | | 2003 | | 57 | | 4 | | 248-53 | | 10.1136/jech.57.4.248 | |
| 376 | | Individual and neighborhood socioeconomic status and inflammation in Mexican American women: what is the role of obesity? | | L. C. Gallo; A. L. Fortmann; K. E. de Los Monteros; P. J. Mills; E. Barrett-Connor; S. C. Roesch; K. A. Matthews | | Psychosom Med | | 2012 | | 74 | | 5 | | 535-42 | | 10.1097/PSY.0b013e31824f5f6d | |
| 377 | | Social identity influences stress appraisals and cardiovascular reactions to acute stress exposure | | S. Gallagher; S. Meaney; O. T. Muldoon | | Br J Health Psychol | | 2014 | | 19 | | 3 | | 566-79 | | 10.1111/bjhp.12056 | |
| 378 | | Social support and stroke risk: An epidemiological study of a population aged 25–64 years in Russia/Siberia (the WHO MONICA-psychosocial program) | | A. V. Gafarova; E. A. Gromova; D. O. Panov; I. V. Gagulin; E. A. Krymov; V. V. Gafarov | | Nevrol. Neiropsikhiatr. Psikhosomat. | | 2019 | | 11 | | 1 | |  | | 10.14412/2074-2711-2019-1-12-20 | |
| 379 | | The influence of social support on risk of acute cardiovascular diseases in female population aged 25-64 in Russia | | V. V. Gafarov; D. O. Panov; E. A. Gromova; I. V. Gagulin; A. V. Gafarova | | Int J Circumpolar Health | | 2013 | | 72 | |  | |  | | 10.3402/ijch.v72i0.21210 | |
| 380 | | [A study of the risk factors of stroke development in the framework of WHO program MONICA-psychosocial"]" | | V. V. Gafarov; E. A. Gromova; I. V. Gagulin; P. I. Pilipenko | | Zh Nevrol Psikhiatr Im S S Korsakova | | 2005 | |  | | Suppl 13 | | 36-41 | |  | |
| 381 | | The risk of myocardial infarction, and social support among the population of 25-64 years in Russia/Siberia | | V. V. Gafarov; E. A. Gromova; I. V. Gagulin; D. O. Panov; E. A. Krymov; R. R. Suleymanov; A. V. Gafarova | | Russ. J. Cardiol. | | 2019 | | 24 | | 6 | | 34-41 | | 10.15829/1560-4071-2019-6-34-41 | |
| 382 | | [Risk of arterial hypertension and social support] | | V. V. Gafarov; E. A. Gromova; A. V. Gafarova; I. V. Gagulin; S. Ekimova Iu | | Klin Med (Mosk) | | 2005 | | 83 | | 8 | | 59-62 | |  | |
| 383 | | ACE gene haplotypes and social networks: Using a biocultural framework to investigate blood pressure variation in African Americans | | K. C. Fuller; C. McCarty; C. Seaborn; C. C. Gravlee; C. J. Mulligan | | PLoS One | | 2018 | | 13 | | 9 | |  | | 10.1371/journal.pone.0204127 | |
| 384 | | Individual- and area-level socioeconomic inequalities in diabetes mellitus in Saskatchewan between 2007 and 2012: a cross-sectional analysis | | D. Fuller; J. Neudorf; S. Lockhart; C. Plante; H. Roberts; T. Bandara; C. Neudorf | | CMAJ Open | | 2019 | | 7 | | 1 | |  | | 10.9778/cmajo.20180042 | |
| 385 | | Neighborhoods and chronic disease onset in later life | | V. A. Freedman; I. B. Grafova; J. Rogowski | | Am J Public Health | | 2011 | | 101 | | 1 | | 79-86 | | 10.2105/ajph.2009.178640 | |
| 386 | | Is job-related stress the link between cardiovascular disease and the law enforcement profession? | | W. D. Franke; M. L. Kohut; D. W. Russell; H. L. Yoo; P. Ekkekakis; S. P. Ramey | | J Occup Environ Med | | 2010 | | 52 | | 5 | | 561-5 | | 10.1097/JOM.0b013e3181dd086b | |
| 387 | | Causal nature of neighborhood deprivation on individual risk of coronary heart disease or ischemic stroke: A prospective national Swedish co-relative control study in men and women | | P. O. Forsberg; H. Ohlsson; K. Sundquist | | Health Place | | 2018 | | 50 | |  | |  | | 10.1016/j.healthplace.2017.12.006 | |
| 388 | | Accelerated aging: A marker for social factors resulting in cardiovascular events? | | S. N. Forrester; R. Zmora; P. J. Schreiner; D. R. Jacobs, Jr.; V. L. Roger; R. J. Thorpe, Jr.; C. I. Kiefe | | SSM Popul Health | | 2021 | | 13 | |  | |  | | 10.1016/j.ssmph.2021.100733 | |
| 389 | | Social Integration and Quality of Social Relationships as Protective Factors for Inflammation in a Nationally Representative Sample of Black Women | | J. Ford; C. Anderson; S. Gillespie; C. Giurgescu; T. Nolan; A. Nowak; K. P. Williams | | J Urban Health | | 2019 | | 96 | | Suppl 1 | | 35-43 | | 10.1007/s11524-018-00337-x | |
| 390 | | Social integration and concentrations of C-reactive protein among US adults | | E. S. Ford; E. B. Loucks; L. F. Berkman | | Ann Epidemiol | | 2006 | | 16 | | 2 | | 78-84 | | 10.1016/j.annepidem.2005.08.005 | |
| 391 | | Distribution of Cardiovascular Health by Individual- and Neighborhood-Level Socioeconomic Status: Findings From the Jackson Heart Study | | R. E. Foraker; C. Bush; M. A. Greiner; M. Sims; K. Henderson; S. Smith; A. Bidulescu; A. B. Shoben; N. C. Hardy; E. O'Brien | | Glob Heart | | 2019 | | 14 | | 3 | | 241-250 | | 10.1016/j.gheart.2019.04.007 | |
| 392 | | Social patterning of chronic disease risk factors in a Latin American city | | N. L. Fleischer; A. V. Diez Roux; M. Alazraqui; H. Spinelli | | J Urban Health | | 2008 | | 85 | | 6 | | 923-37 | | 10.1007/s11524-008-9319-2 | |
| 393 | | Outcomes and inequalities in diabetes from 2004/2005 to 2011/2012: English longitudinal study | | R. Fleetcroft; M. Asaria; S. Ali; R. Cookson | | Br J Gen Pract | | 2017 | | 67 | |  | |  | | 10.3399/bjgp16X688381 | |
| 394 | | Chronic Disease, the Built Environment, and Unequal Health Risks in the 500 Largest U.S. Cities | | K. M. Fitzpatrick; D. Willis | | Int J Environ Res Public Health | | 2020 | | 17 | | 8 | |  | | 10.3390/ijerph17082961 | |
| 395 | | The Prevalence of Diabetes Among Overweight and Obese Individuals is Higher in Poorer than in Richer Neighbourhoods | | M. M. Finkelstein | | Can. J. Diabetes | | 2008 | | 32 | | 3 | | 190-197 | | 10.1016/S1499-2671(08)23009-1 | |
| 396 | | Neighbourhood socioeconomic characteristics and blood pressure among Jamaican youth: a pooled analysis of data from observational studies | | T. S. Ferguson; N. O. M. Younger-Coleman; J. Mullings; D. Francis; L. G. Greene; P. Lyew-Ayee; R. Wilks | | PeerJ | | 2020 | | 8 | |  | |  | | 10.7717/peerj.10058 | |
| 397 | | Subgroup Variation and Neighborhood Social Gradients-an Analysis of Hypertension and Diabetes Among Asian Patients (New York City, 2014-2017) | | J. M. Feldman; S. Conderino; N. S. Islam; L. E. Thorpe | | J Racial Ethn Health Disparities | | 2021 | | 8 | | 1 | | 256-263 | | 10.1007/s40615-020-00779-7 | |
| 398 | | Demographics of cardiac arrest: association with residence in a low-income area | | S. Feero; J. R. Hedges; P. Stevens | | Acad Emerg Med | | 1995 | | 2 | | 1 | |  | | 10.1111/j.1553-2712.1995.tb03071.x | |
| 399 | | Association of Socio-Environmental Determinants with Diabetes Prevalence in the Athens Metropolitan Area, Greece: A Spatial Analysis | | A. Faka; C. Chalkias; D. Montano; E. N. Georgousopoulou; A. Tripitsidis; E. Koloverou; D. Tousoulis; C. Pitsavos; D. B. Panagiotakos | | Rev Diabet Stud | | 2018 | | 14 | | 4 | | 381-389 | | 10.1900/rds.2017.14.381 | |
| 400 | | Race and perceived racism, education, and hypertension among Brazilian civil servants: the Pró-Saúde Study | | E. Faerstein; D. Chor; G. L. Werneck; S. Lopes Cde; G. Kaplan | | Rev Bras Epidemiol | | 2014 | | 17 Suppl 2 | |  | | 81-7 | | 10.1590/1809-4503201400060007 | |
| 401 | | Movers and stayers: The geography of residential mobility and CVD hospitalisations in Auckland, New Zealand | | D. J. Exeter; C. E. Sabel; G. Hanham; A. C. Lee; S. Wells | | Soc Sci Med | | 2015 | | 133 | |  | | 331-9 | | 10.1016/j.socscimed.2014.11.056 | |
| 402 | | Individual and area-based indicators of acculturation and the metabolic syndrome among low-income Mexican American women living in a border region | | K. Espinosa de Los Monteros; L. C. Gallo; J. P. Elder; G. A. Talavera | | Am J Public Health | | 2008 | | 98 | | 11 | | 1979-86 | | 10.2105/ajph.2008.141903 | |
| 403 | | Neighborhood context and mortality among older Mexican Americans: is there a barrio advantage? | | K. Eschbach; G. V. Ostir; K. V. Patel; K. S. Markides; J. S. Goodwin | | Am J Public Health | | 2004 | | 94 | | 10 | | 1807-12 | | 10.2105/ajph.94.10.1807 | |
| 404 | | Geographic distribution of stroke incidence within an urban population: Relations to socioeconomic circumstances and prevalence of cardiovascular risk factors | | G. Engström; I. Jerntorp; H. Pessah-Rasmussen; B. Hedblad; G. Berglund; L. Janzon | | Stroke | | 2001 | | 32 | | 5 | | 1098-1103 | | 10.1161/01.STR.32.5.1098 | |
| 405 | | Distribution and determinants of ischaemic heart disease in an urban population. A study from the myocardial infarction register in Malmö, Sweden | | G. Engström; G. Berglund; M. Göransson; O. Hansen; B. Hedblad; J. Merlo; P. Tydén; L. Janzon | | J Intern Med | | 2000 | | 247 | | 5 | | 588-96 | | 10.1046/j.1365-2796.2000.00663.x | |
| 406 | | Social ties and change in social ties in relation to subsequent total and cause-specific mortality and coronary heart disease incidence in men | | P. M. Eng; E. B. Rimm; G. Fitzmaurice; I. Kawachi | | Am J Epidemiol | | 2002 | | 155 | | 8 | | 700-9 | | 10.1093/aje/155.8.700 | |
| 407 | | Is social participation associated with cardiovascular disease risk factors? | | A. Ellaway; S. Macintyre | | Soc Sci Med | | 2007 | | 64 | | 7 | | 1384-91 | | 10.1016/j.socscimed.2006.11.022 | |
| 408 | | Prevalence and determinants of self-reported high blood pressure among women of reproductive age in Benin: a population-based study | | M. Ekholuenetale; A. Barrow | | Clin Hypertens | | 2020 | | 26 | |  | |  | | 10.1186/s40885-020-00145-z | |
| 409 | | How are physical activity behaviors and cardiovascular risk factors associated with characteristics of the built and social residential environment? | | M. Eichinger; S. Titze; B. Haditsch; T. E. Dorner; W. J. Stronegger | | PLoS One | | 2015 | | 10 | | 6 | |  | | 10.1371/journal.pone.0126010 | |
| 410 | | Small-area variation  of cardiovascular diseases and select risk factors and their association to household and area poverty in South Africa: Capturing emerging trends in South Africa to better target local level interventions | | N. Dwane; N. Wabiri; S. Manda | | PLoS One | | 2020 | | 15 | | 4 | |  | | 10.1371/journal.pone.0230564 | |
| 411 | | Neighborhood disadvantage and chronic disease management | | S. N. M. Durfey; A. J. H. Kind; W. R. Buckingham; E. H. DuGoff; A. N. Trivedi | | Health Serv Res | | 2019 | | 54 Suppl 1 | | Suppl 1 | | 206-216 | | 10.1111/1475-6773.13092 | |
| 412 | | Perceived spatial stigma, body mass index and blood pressure: a global positioning system study among low-income housing residents in New York City | | D. T. Duncan; R. R. Ruff; B. Chaix; S. D. Regan; J. H. Williams; J. Ravenell; M. A. Bragg; G. Ogedegbe; B. Elbel | | Geospat Health | | 2016 | | 11 | | 2 | |  | | 10.4081/gh.2016.399 | |
| 413 | | The Women's Health Initiative: The food environment, neighborhood socioeconomic status, BMI, and blood pressure | | T. Dubowitz; M. Ghosh-Dastidar; C. Eibner; M. E. Slaughter; M. Fernandes; E. A. Whitsel; C. E. Bird; A. Jewell; K. L. Margolis; W. Li; Y. L. Michael; R. A. Shih; J. E. Manson; J. J. Escarce | | Obesity (Silver Spring) | | 2012 | | 20 | | 4 | | 862-71 | | 10.1038/oby.2011.141 | |
| 414 | | The geography of diabetes by census tract in a large sample of insured adults in King County, Washington, 2005-2006 | | A. Drewnowski; C. D. Rehm; A. V. Moudon; D. Arterburn | | Prev Chronic Dis | | 2014 | | 11 | |  | |  | | 10.5888/pcd11.140135 | |
| 415 | | Social support and arterial pressure in a central Mexican community | | W. W. Dressler; A. Mata; A. Chavez; F. E. Viteri; P. Gallagher | | Psychosom Med | | 1986 | | 48 | | 5 | | 338-50 | | 10.1097/00006842-198605000-00004 | |
| 416 | | Culture and the Immune System: Cultural Consonance in Social Support and C-reactive Protein in Urban Brazil | | W. W. Dressler; M. C. Balieiro; R. P. Ribeiro; J. E. Dos Santos | | Med Anthropol Q | | 2016 | | 30 | | 2 | | 259-77 | | 10.1111/maq.12213 | |
| 417 | | The cultural construction of social support in Brazil: associations with health outcomes | | W. W. Dressler; M. C. Balieiro; J. E. Dos Santos | | Cult Med Psychiatry | | 1997 | | 21 | | 3 | | 303-35 | | 10.1023/a:1005394416255 | |
| 418 | | Subclinical coronary atherosclerosis and neighbourhood deprivation in an urban region | | N. Dragano; B. Hoffmann; A. Stang; S. Moebus; P. E. Verde; S. Weyers; S. Möhlenkamp; A. Schmermund; K. Mann; K. H. Jöckel; R. Erbel; J. Siegrist | | Eur J Epidemiol | | 2009 | | 24 | | 1 | | 25-35 | | 10.1007/s10654-008-9292-9 | |
| 419 | | Neighbourhood socioeconomic status and cardiovascular risk factors: a multilevel analysis of nine cities in the Czech Republic and Germany | | N. Dragano; M. Bobak; N. Wege; A. Peasey; P. E. Verde; R. Kubinova; S. Weyers; S. Moebus; S. Möhlenkamp; A. Stang; R. Erbel; K. H. Jöckel; J. Siegrist; H. Pikhart | | BMC Public Health | | 2007 | | 7 | |  | |  | | 10.1186/1471-2458-7-255 | |
| 420 | | Risk factors of coronary artery disease and their relationships with dietetic and social variables | | J. E. dos Santos; W. W. Dressler; F. Viteri; R. Preto | | Arq. Bras. Cardiol. | | 1994 | | 63 | | 5 | | 371-375 | |  | |
| 421 | | Circadian rhythm of cortisol and neighborhood characteristics in a population-based sample: the Multi-Ethnic Study of Atherosclerosis | | D. P. Do; A. V. Diez Roux; A. Hajat; A. H. Auchincloss; S. S. Merkin; N. Ranjit; S. Shea; T. Seeman | | Health Place | | 2011 | | 17 | | 2 | | 625-32 | | 10.1016/j.healthplace.2010.12.019 | |
| 422 | | Impact of socioeconomic status on coronary artery calcification | | D. Djekic; O. Angerås; G. Lappas; E. Fagman; B. Fagerberg; G. Bergström; A. Rosengren | | Eur J Prev Cardiol | | 2018 | | 25 | | 16 | | 1756-1764 | | 10.1177/2047487318792103 | |
| 423 | | Neighborhood environments and coronary heart disease: a multilevel analysis | | A. V. Diez-Roux; F. J. Nieto; C. Muntaner; H. A. Tyroler; G. W. Comstock; E. Shahar; L. S. Cooper; R. L. Watson; M. Szklo | | Am J Epidemiol | | 1997 | | 146 | | 1 | | 48-63 | | 10.1093/oxfordjournals.aje.a009191 | |
| 424 | | A multilevel analysis of income inequality and cardiovascular disease risk factors | | A. V. Diez-Roux; B. G. Link; M. E. Northridge | | Soc. Sci. Med. | | 2000 | | 50 | | 5 | | 673-687 | | 10.1016/S0277-9536(99)00320-2 | |
| 425 | | Neighborhood of residence and incidence of coronary heart disease | | A. V. Diez Roux; S. S. Merkin; D. Arnett; L. Chambless; M. Massing; F. J. Nieto; P. Sorlie; M. Szklo; H. A. Tyroler; R. L. Watson | | N Engl J Med | | 2001 | | 345 | | 2 | | 99-106 | | 10.1056/nejm200107123450205 | |
| 426 | | Neighborhood characteristics and components of the insulin resistance syndrome in young adults: the coronary artery risk development in young adults (CARDIA) study | | A. V. Diez Roux; D. R. Jacobs; C. I. Kiefe | | Diabetes Care | | 2002 | | 25 | | 11 | | 1976-82 | | 10.2337/diacare.25.11.1976 | |
| 427 | | Socioeconomic disadvantage and change in blood pressure associated with aging | | A. V. Diez Roux; L. Chambless; S. S. Merkin; D. Arnett; M. Eigenbrodt; F. J. Nieto; M. Szklo; P. Sorlie | | Circulation | | 2002 | | 106 | | 6 | | 703-10 | | 10.1161/01.cir.0000025402.84600.cd | |
| 428 | | Social Network Trajectories in Myocardial Infarction Versus Ischemic Stroke | | A. Dhand; W. T. Longstreth, Jr.; P. H. M. Chaves; M. S. Dhamoon | | J Am Heart Assoc | | 2018 | | 7 | | 8 | |  | | 10.1161/jaha.117.008029 | |
| 429 | | Neighborhood and Network Disadvantage among Urban Renters | | M. Desmond; W. H. An | | Sociol. Sci. | | 2015 | | 2 | |  | | 329-349 | | 10.15195/v2.a16 | |
| 430 | | A small-area ecologic study of myocardial infarction, neighborhood deprivation, and sex: a Bayesian modeling approach | | S. Deguen; B. Lalloue; D. Bard; S. Havard; D. Arveiler; D. Zmirou-Navier | | Epidemiology | | 2010 | | 21 | | 4 | | 459-66 | | 10.1097/EDE.0b013e3181e09925 | |
| 431 | | Differences in atherosclerosis according to area level socioeconomic deprivation: cross sectional, population based study | | K. A. Deans; V. Bezlyak; I. Ford; G. D. Batty; H. Burns; J. Cavanagh; E. de Groot; A. McGinty; K. Millar; P. G. Shiels; C. Tannahill; Y. N. Velupillai; N. Sattar; C. J. Packard | | Bmj | | 2009 | | 339 | |  | |  | | 10.1136/bmj.b4170 | |
| 432 | | Social, cultural and economical determinants of diabetes mellitus in Kalutara district, Sri Lanka: a cross sectional descriptive study | | A. P. De Silva; S. H. P. De Silva; I. K. Liyanage; L. C. Rajapakse; K. S. A. Jayasinghe; P. Katulanda; C. N. Wijeratne; S. Wijeratne | | Int. J. Equity Health | | 2012 | | 11 | |  | |  | | 10.1186/1475-9276-11-76 | |
| 433 | | Sex and ethnicity modify the associations between individual and contextual socioeconomic indicators and ideal cardiovascular health: MESA study | | A. C. F. De Moraes; H. B. Carvalho; R. L. McClelland; A. V. Diez-Roux; M. Szklo | | J Public Health (Oxf) | | 2019 | | 41 | | 3 | |  | | 10.1093/pubmed/fdy145 | |
| 434 | | Perceived job stress and incidence of coronary events: 3-year follow-up of the Belgian Job Stress Project cohort | | D. De Bacquer; E. Pelfrene; E. Clays; R. Mak; M. Moreau; P. de Smet; M. Kornitzer; G. De Backer | | Am J Epidemiol | | 2005 | | 161 | | 5 | | 434-41 | | 10.1093/aje/kwi040 | |
| 435 | | Association of negative and positive social ties with fibrinogen levels in young women | | M. C. Davis; P. D. Swan | | Health Psychol | | 1999 | | 18 | | 2 | | 131-9 | | 10.1037//0278-6133.18.2.131 | |
| 436 | | Psychosocial work environment and coronary heart disease | | M. Danelia; D. Trapaidze | | Georgian Med News | | 2005 | |  | |  | | 56-8 | |  | |
| 437 | | Accuracy of Cardiovascular Risk Prediction Varies by Neighborhood Socioeconomic Position: A Retrospective Cohort Study | | J. E. Dalton; A. T. Perzynski; D. A. Zidar; M. B. Rothberg; C. J. Coulton; A. T. Milinovich; D. Einstadter; J. K. Karichu; N. V. Dawson | | Ann Intern Med | | 2017 | | 167 | | 7 | | 456-464 | | 10.7326/m16-2543 | |
| 438 | | Relationship between organizational support at work and risk of non-communicable chronic diseases in a health service | | É. S. M. Da Costa; A. Hyeda; E. M. C. P. Maluf | | Rev. Bras. Med. Trab. | | 2017 | | 15 | | 2 | | 134-141 | | 10.5327/Z1679443520176046 | |
| 439 | | Socioeconomic status and access to care and the incidence of a heart failure diagnosis in the inpatient and outpatient settings | | C. C. Cuthbertson; G. Heiss; J. D. Wright; R. Camplain; M. D. Patel; R. E. Foraker; K. Matsushita; N. Puccinelli-Ortega; A. M. Shah; A. M. Kucharska-Newton | | Ann Epidemiol | | 2018 | | 28 | | 6 | | 350-355 | | 10.1016/j.annepidem.2018.04.003 | |
| 440 | | Using GIS and secondary data to target diabetes-related public health efforts | | A. B. Curtis; C. Kothari; R. Paul; E. Connors | | Public Health Rep | | 2013 | | 128 | | 3 | | 212-20 | | 10.1177/003335491312800311 | |
| 441 | | County-level contextual factors associated with diabetes incidence in the United States | | S. A. Cunningham; S. A. Patel; G. L. Beckles; L. S. Geiss; N. Mehta; H. Xie; G. Imperatore | | Ann Epidemiol | | 2018 | | 28 | | 1 | | 20-25.e2 | | 10.1016/j.annepidem.2017.11.002 | |
| 442 | | Protective and harmful effects of neighborhood-level deprivation on individual-level health knowledge, behavior changes, and risk of coronary heart disease | | C. Cubbin; M. A. Winkleby | | Am J Epidemiol | | 2005 | | 162 | | 6 | | 559-68 | | 10.1093/aje/kwi250 | |
| 443 | | Neighborhood deprivation and cardiovascular disease risk factors: protective and harmful effects | | C. Cubbin; K. Sundquist; H. Ahlén; S. E. Johansson; M. A. Winkleby; J. Sundquist | | Scand J Public Health | | 2006 | | 34 | | 3 | | 228-37 | | 10.1080/14034940500327935 | |
| 444 | | Neighborhood context and cardiovascular disease risk factors: the contribution of material deprivation | | C. Cubbin; W. C. Hadden; M. A. Winkleby | | Ethn Dis | | 2001 | | 11 | | 4 | | 687-700 | |  | |
| 445 | | Cross-sectional study of area-level disadvantage and glycaemic-related risk in community health service users in the Southern.IML Research (SIMLR) cohort | | R. Cross; A. Bonney; D. J. Mayne; K. M. Weston | | Aust Health Rev | | 2019 | | 43 | | 1 | | 85-91 | | 10.1071/ah16298 | |
| 446 | | Social support and trait personality are independently associated with resting cardiovascular function in women | | A. M. Creaven; S. Howard; B. M. Hughes | | Br J Health Psychol | | 2013 | | 18 | | 3 | | 556-73 | | 10.1111/bjhp.12001 | |
| 447 | | Relation between neighborhood median housing value and hypertension risk among black women in the United States | | Y. C. Cozier; J. R. Palmer; N. J. Horton; L. Fredman; L. A. Wise; L. Rosenberg | | Am J Public Health | | 2007 | | 97 | | 4 | | 718-24 | | 10.2105/ajph.2005.074740 | |
| 448 | | Neighborhood Socioeconomic Status in Relation to Serum Biomarkers in the Black Women's Health Study | | Y. C. Cozier; M. A. Albert; N. Castro-Webb; P. F. Coogan; P. Ridker; H. W. Kaufman; J. R. Palmer; L. Rosenberg | | J Urban Health | | 2016 | | 93 | | 2 | | 279-91 | | 10.1007/s11524-016-0034-0 | |
| 449 | | Locality deprivation and Type 2 diabetes incidence: a local test of relative inequalities | | M. Cox; P. J. Boyle; P. G. Davey; Z. Feng; A. D. Morris | | Soc Sci Med | | 2007 | | 65 | | 9 | | 1953-64 | | 10.1016/j.socscimed.2007.05.043 | |
| 450 | | The Association of Neighborhood Gene-Environment Susceptibility with Cortisol and Blood Pressure in African-American Adults | | S. M. Coulon; D. K. Wilson; M. L. Van Horn; G. A. Hand; S. Kresovich | | Ann Behav Med | | 2016 | | 50 | | 1 | | 98-107 | | 10.1007/s12160-015-9737-9 | |
| 451 | | Multilevel Associations of Neighborhood Poverty, Crime, and Satisfaction With Blood Pressure in African-American Adults | | S. M. Coulon; D. K. Wilson; K. A. Alia; M. L. Van Horn | | Am J Hypertens | | 2016 | | 29 | | 1 | | 90-5 | | 10.1093/ajh/hpv060 | |
| 452 | | Measuring change in health care equity using small-area administrative data - evidence from the English NHS 2001-2008 | | R. Cookson; M. Laudicella; P. L. Donni | | Soc Sci Med | | 2012 | | 75 | | 8 | | 1514-22 | | 10.1016/j.socscimed.2012.05.033 | |
| 453 | | Neighbourhood property value and type 2 diabetes mellitus in the Maastricht study: A multilevel study | | D. Consolazio; A. Koster; S. Sarti; M. T. Schram; C. D. A. Stehouwer; E. J. Timmermans; A. Wesselius; H. Bosma | | PLoS One | | 2020 | | 15 | | 6 | |  | | 10.1371/journal.pone.0234324 | |
| 454 | | A diabetes risk index for small areas in England | | P. Congdon | | Health Place | | 2020 | | 63 | |  | |  | | 10.1016/j.healthplace.2020.102340 | |
| 455 | | Performance of the Atherosclerotic Cardiovascular Disease Pooled Cohort Risk Equations by Socioeconomic Status. The REasons for Geographic And Racial Differences in Stroke (REGARDS) Study | | L. D. Colantonio; A. P. Carson; D. M. Lloyd-Jones; J. S. Richman; G. Howard; V. J. Howard; M. M. Safford; P. Muntner; D. C. Goff | | Circulation | | 2016 | | 133 | |  | |  | |  | |
| 456 | | Impact of Individual and Neighborhood Factors on Cardiovascular Risk in White Hispanic and Non-Hispanic Women and Men | | T. Cohn; A. Miller; L. Fogg; L. T. Braun; L. Coke | | Res Nurs Health | | 2017 | | 40 | | 2 | | 120-131 | | 10.1002/nur.21778 | |
| 457 | | A Swiss paradox" in the United States? Level of spatial aggregation changes the association between income inequality and morbidity for older Americans" | | S. A. Cohen; M. L. Greaney; A. C. Klassen | | Int J Health Geogr | | 2019 | | 18 | | 1 | |  | | 10.1186/s12942-019-0192-x | |
| 458 | | Individual and Neighborhood Deprivation and Carotid Stiffness | | R. E. Climie; P. Boutouyrie; M. C. Perier; C. Guibout; T. T. van Sloten; F. Thomas; N. Danchin; J. E. Sharman; S. Laurent; X. Jouven; J. P. Empana | | Hypertension | | 2019 | | 73 | | 6 | | 1185-1194 | | 10.1161/hypertensionaha.118.12186 | |
| 459 | | Association between neighborhood-level socioeconomic deprivation and incident hypertension: A longitudinal analysis of data from the Dallas heart study | | S. E. Claudel; J. Adu-Brimpong; A. Banks; C. Ayers; M. A. Albert; S. R. Das; J. A. de Lemos; T. Leonard; I. J. Neeland; J. P. Rivers; T. M. Powell-Wiley | | Am Heart J | | 2018 | | 204 | |  | | 109-118 | | 10.1016/j.ahj.2018.07.005 | |
| 460 | | Cardiovascular inflammation in healthy women: multilevel associations with state-level prosperity, productivity and income inequality | | C. R. Clark; P. M. Ridker; M. J. Ommerborn; C. E. Huisingh; B. Coull; J. E. Buring; L. F. Berkman | | BMC Public Health | | 2012 | | 12 | |  | |  | | 10.1186/1471-2458-12-211 | |
| 461 | | Neighborhood disadvantage, neighborhood safety and cardiometabolic risk factors in African Americans: biosocial associations in the Jackson Heart study | | C. R. Clark; M. J. Ommerborn; D. A. Hickson; K. N. Grooms; M. Sims; H. A. Taylor; M. A. Albert | | PLoS One | | 2013 | | 8 | | 5 | |  | | 10.1371/journal.pone.0063254 | |
| 462 | | Latent constructs in psychosocial factors associated with cardiovascular disease: an examination by race and sex | | C. J. Clark; K. M. Henderson; C. F. de Leon; H. Guo; S. Lunos; D. A. Evans; S. A. Everson-Rose | | Front Psychiatry | | 2012 | | 3 | |  | |  | | 10.3389/fpsyt.2012.00005 | |
| 463 | | Neighborhood cohesion is associated with reduced risk of stroke mortality | | C. J. Clark; H. Guo; S. Lunos; N. T. Aggarwal; T. Beck; D. A. Evans; C. Mendes de Leon; S. A. Everson-Rose | | Stroke | | 2011 | | 42 | | 5 | | 1212-7 | | 10.1161/strokeaha.110.609164 | |
| 464 | | Cross-sectional associations between residential environmental exposures and cardiovascular diseases | | A. Chum; P. O'Campo | | BMC Public Health | | 2015 | | 15 | |  | |  | | 10.1186/s12889-015-1788-0 | |
| 465 | | Exposure to Neighborhood Foreclosures and Changes in Cardiometabolic Health: Results From MESA | | P. J. Christine; K. Moore; N. D. Crawford; T. Barrientos-Gutierrez; B. N. Sánchez; T. Seeman; A. V. Diez Roux | | Am J Epidemiol | | 2017 | | 185 | | 2 | | 106-114 | | 10.1093/aje/kww186 | |
| 466 | | Longitudinal Associations Between Neighborhood Physical and Social Environments and Incident Type 2 Diabetes Mellitus: The Multi-Ethnic Study of Atherosclerosis (MESA) | | P. J. Christine; A. H. Auchincloss; A. G. Bertoni; M. R. Carnethon; B. N. Sánchez; K. Moore; S. D. Adar; T. B. Horwich; K. E. Watson; A. V. Diez Roux | | JAMA Intern Med | | 2015 | | 175 | | 8 | | 1311-20 | | 10.1001/jamainternmed.2015.2691 | |
| 467 | | Individual and neighborhood socioeconomic status characteristics and prevalence of metabolic syndrome: the Atherosclerosis Risk in Communities (ARIC) Study | | K. L. Chichlowska; K. M. Rose; A. V. Diez-Roux; S. H. Golden; A. M. McNeill; G. Heiss | | Psychosom Med | | 2008 | | 70 | | 9 | | 986-92 | | 10.1097/PSY.0b013e318183a491 | |
| 468 | | Analysis of spatio-temporal variations in stroke incidence and case-fatality in Hong Kong | | P. H. Chau; J. Woo; W. B. Goggins; M. Wong; K. C. Chan; S. C. Ho | | Geospat Health | | 2011 | | 6 | | 1 | | 13-20 | | 10.4081/gh.2011.153 | |
| 469 | | Sociodemographic disparities in non-diabetic hyperglycaemia and the transition to type 2 diabetes: evidence from the English Longitudinal Study of Ageing | | G. Chatzi; T. Mason; T. Chandola; W. Whittaker; E. Howarth; S. Cotterill; R. Ravindrarajah; E. McManus; M. Sutton; P. Bower | | Diabet Med | | 2020 | | 37 | | 9 | | 1536-1544 | | 10.1111/dme.14343 | |
| 470 | | Neighborhood deprivation and biomarkers of health in Britain: the mediating role of the physical environment | | M. P. Chaparro; M. Benzeval; E. Richardson; R. Mitchell | | BMC Public Health | | 2018 | | 18 | | 1 | |  | | 10.1186/s12889-018-5667-3 | |
| 471 | | Social Integration and Reduced Risk of Coronary Heart Disease in Women: The Role of Lifestyle Behaviors | | S. C. Chang; M. Glymour; M. Cornelis; S. Walter; E. B. Rimm; E. Tchetgen Tchetgen; I. Kawachi; L. D. Kubzansky | | Circ Res | | 2017 | | 120 | | 12 | | 1927-1937 | | 10.1161/circresaha.116.309443 | |
| 472 | | Associations between psychological stress and smoking, drinking, obesity, and high blood pressure in an upper middle-income country in the African region | | T. Chamik; B. Viswanathan; J. Gedeon; P. Bovet | | Stress Health | | 2018 | | 34 | | 1 | | 93-101 | | 10.1002/smi.2766 | |
| 473 | | Socioeconomic determinants of hypertension and prehypertension in Peru: Evidence from the Peruvian Demographic and Health Survey | | D. Chambergo-Michilot; A. Rebatta-Acuña; C. J. Delgado-Flores; C. J. Toro-Huamanchumo | | PLoS One | | 2021 | | 16 | | 1 | |  | | 10.1371/journal.pone.0245730 | |
| 474 | | Neighborhood socioeconomic deprivation and residential instability: effects on incidence of ischemic heart disease and survival after myocardial infarction | | B. Chaix; M. Rosvall; J. Merlo | | Epidemiology | | 2007 | | 18 | | 1 | | 104-11 | | 10.1097/01.ede.0000249573.22856.9a | |
| 475 | | Income change at retirement, neighbourhood-based social support, and ischaemic heart disease: results from the prospective cohort study Men born in 1914"" | | B. Chaix; S. O. Isacsson; L. Råstam; M. Lindström; J. Merlo | | Soc Sci Med | | 2007 | | 64 | | 4 | | 818-29 | | 10.1016/j.socscimed.2006.10.018 | |
| 476 | | Residential environment and blood pressure in the PRIME Study: is the association mediated by body mass index and waist circumference? | | B. Chaix; P. Ducimetière; T. Lang; B. Haas; M. Montaye; J. B. Ruidavets; D. Arveiler; P. Amouyel; J. Ferrières; A. Bingham; P. Chauvin | | J Hypertens | | 2008 | | 26 | | 6 | | 1078-84 | | 10.1097/HJH.0b013e3282fd991f | |
| 477 | | Neighborhood effects on health: correcting bias from neighborhood effects on participation | | B. Chaix; N. Billaudeau; F. Thomas; S. Havard; D. Evans; Y. Kestens; K. Bean | | Epidemiology | | 2011 | | 22 | | 1 | | 18-26 | | 10.1097/EDE.0b013e3181fd2961 | |
| 478 | | Individual/neighborhood social factors and blood pressure in the RECORD Cohort Study: which risk factors explain the associations? | | B. Chaix; K. Bean; C. Leal; F. Thomas; S. Havard; D. Evans; B. Jégo; B. Pannier | | Hypertension | | 2010 | | 55 | | 3 | | 769-75 | | 10.1161/hypertensionaha.109.143206 | |
| 479 | | Social isolation, vital exhaustion, and incident heart failure: findings from the Atherosclerosis Risk in Communities Study | | C. W. Cené; L. Loehr; F. C. Lin; W. P. Hammond; R. E. Foraker; K. Rose; T. Mosley; G. Corbie-Smith | | Eur J Heart Fail | | 2012 | | 14 | | 7 | | 748-53 | | 10.1093/eurjhf/hfs064 | |
| 480 | | Geographic and statistic stability of deprivation aggregated measures at different spatial units in health research | | A. Cebrecos; M. F. Dominguez-Berjon; I. Duque; M. Franco; F. Escobar | | Appl. Geogr. | | 2018 | | 95 | |  | |  | | 10.1016/j.apgeog.2018.04.001 | |
| 481 | | Neighborhood Disadvantage and Variations in Blood Pressure | | M. L. Cathorall; H. B. Xin; A. Peachey; D. L. Bibeau; M. Schulz; R. Aronson | | Am. J. Health Educ. | | 2015 | | 46 | | 5 | | 266-273 | | 10.1080/19325037.2015.1055018 | |
| 482 | | Socially isolated children 20 years later: risk of cardiovascular disease | | A. Caspi; H. Harrington; T. E. Moffitt; B. J. Milne; R. Poulton | | Arch Pediatr Adolesc Med | | 2006 | | 160 | | 8 | | 805-11 | | 10.1001/archpedi.160.8.805 | |
| 483 | | Variation in the magnitude of black-white differences in stroke mortality by community occupational structure | | M. Casper; S. Wing; D. Strogatz | | J Epidemiol Community Health | | 1991 | | 45 | | 4 | | 302-6 | | 10.1136/jech.45.4.302 | |
| 484 | | Measures of SES for Electronic Health Record-based Research | | J. A. Casey; J. Pollak; M. M. Glymour; E. R. Mayeda; A. G. Hirsch; B. S. Schwartz | | Am J Prev Med | | 2018 | | 54 | | 3 | | 430-439 | | 10.1016/j.amepre.2017.10.004 | |
| 485 | | Designing Faith-Based Blood Pressure Interventions to Reach Young Black Men | | L. Carter-Edwards; R. Lindquist; N. Redmond; C. M. Turner; C. Harding; J. Oliver; L. B. West; J. Ravenell; J. M. Shikany | | Am J Prev Med | | 2018 | | 55 | | 5 Suppl 1 | |  | | 10.1016/j.amepre.2018.05.009 | |
| 486 | | Cumulative socioeconomic status across the life course and subclinical atherosclerosis | | A. P. Carson; K. M. Rose; D. J. Catellier; J. S. Kaufman; S. B. Wyatt; A. V. Diez-Roux; G. Heiss | | Ann Epidemiol | | 2007 | | 17 | | 4 | | 296-303 | | 10.1016/j.annepidem.2006.07.009 | |
| 487 | | Neighbourhood socioeconomic status and coronary heart disease in individuals between 40 and 50 years | | A. C. Carlsson; X. Li; M. J. Holzmann; P. Wändell; D. Gasevic; J. Sundquist; K. Sundquist | | Heart | | 2016 | | 102 | | 10 | | 775-82 | | 10.1136/heartjnl-2015-308784 | |
| 488 | | Neighborhood socioeconomic status at the age of 40 years and ischemic stroke before the age of 50 years: A nationwide cohort study from Sweden | | A. C. Carlsson; X. Li; M. J. Holzmann; J. Ärnlöv; P. Wändell; D. Gasevic; J. Sundquist; K. Sundquist | | Int J Stroke | | 2017 | | 12 | | 8 | | 815-826 | | 10.1177/1747493017702663 | |
| 489 | | Effect of satisfaction with social support on blood pressure in normotensive and borderline hypertensive men and women | | R. A. Carels; J. A. Blumenthal; A. Sherwood | | Int J Behav Med | | 1998 | | 5 | | 1 | | 76-85 | | 10.1207/s15327558ijbm0501_6 | |
| 490 | | Association between neighborhood disadvantage and hypertension prevalence, awareness, treatment, and control in older adults: results from the University of Alabama at Birmingham Study of Aging | | D. R. Buys; V. J. Howard; L. A. McClure; K. C. Buys; P. Sawyer; R. M. Allman; E. B. Levitan | | Am J Public Health | | 2015 | | 105 | | 6 | | 1181-8 | | 10.2105/ajph.2014.302048 | |
| 491 | | Use of measures of socioeconomic deprivation in planning primary health care workforce and defining health care need in Australia | | D. C. Butler; S. Petterson; A. Bazemore; K. A. Douglas | | Aust J Rural Health | | 2010 | | 18 | | 5 | | 199-204 | | 10.1111/j.1440-1584.2010.01154.x | |
| 492 | | Relationship between loneliness, social isolation and modifiable risk factors for cardiovascular disease: a latent class analysis | | F. Bu; A. Steptoe; D. Fancourt | | J Epidemiol Community Health | | 2021 | |  | |  | |  | | 10.1136/jech-2020-215539 | |
| 493 | | Neighborhood stressors and cardiovascular health: crime and C-reactive protein in Dallas, USA | | C. R. Browning; K. A. Cagney; J. Iveniuk | | Soc Sci Med | | 2012 | | 75 | | 7 | | 1271-9 | | 10.1016/j.socscimed.2012.03.027 | |
| 494 | | Individual socio-economic status, community socio-economic status and stroke in New Zealand: a case control study | | P. Brown; M. Guy; J. Broad | | Soc Sci Med | | 2005 | | 61 | | 6 | | 1174-88 | | 10.1016/j.socscimed.2005.02.003 | |
| 495 | | Neighborhood disadvantage and ischemic stroke: the Cardiovascular Health Study (CHS) | | A. F. Brown; L. J. Liang; S. D. Vassar; S. Stein-Merkin; W. T. Longstreth, Jr.; B. Ovbiagele; T. Yan; J. J. Escarce | | Stroke | | 2011 | | 42 | | 12 | | 3363-8 | | 10.1161/strokeaha.111.622134 | |
| 496 | | Socially isolated individuals are more prone to have newly diagnosed and prevalent type 2 diabetes mellitus - the Maastricht study | | S. Brinkhues; N. Dukers-Muijrers; C. Hoebe; C. J. H. van der Kallen; P. C. Dagnelie; A. Koster; R. M. A. Henry; S. J. S. Sep; N. C. Schaper; C. D. A. Stehouwer; H. Bosma; P. H. M. Savelkoul; M. T. Schram | | BMC Public Health | | 2017 | | 17 | | 1 | |  | | 10.1186/s12889-017-4948-6 | |
| 497 | | Health inequalities in Germany: do regional-level variables explain differentials in cardiovascular risk? | | J. Breckenkamp; A. Mielck; O. Razum | | BMC Public Health | | 2007 | | 7 | |  | |  | | 10.1186/1471-2458-7-132 | |
| 498 | | Socioeconomic disparities in first stroke incidence, quality of care, and survival: a nationwide registry-based cohort study of 44 million adults in England | | B. D. Bray; L. Paley; A. Hoffman; M. James; P. Gompertz; C. D. A. Wolfe; H. Hemingway; A. G. Rudd | | Lancet Public Health | | 2018 | | 3 | | 4 | |  | | 10.1016/s2468-2667(18)30030-6 | |
| 499 | | Residential Racial Isolation and Spatial Patterning of Hypertension in Durham, North Carolina | | M. A. Bravo; B. C. Batch; M. L. Miranda | | Prev Chronic Dis | | 2019 | | 16 | |  | |  | | 10.5888/pcd16.180445 | |
| 500 | | Residential Racial Isolation and Spatial Patterning of Type 2 Diabetes Mellitus in Durham, North Carolina | | M. A. Bravo; R. Anthopolos; R. T. Kimbro; M. L. Miranda | | Am J Epidemiol | | 2018 | | 187 | | 7 | | 1467-1476 | | 10.1093/aje/kwy026 | |
| 501 | | Changing social patterns of risk factors for cardiovascular disease in a Swedish community intervention programme | | I. Brännström; L. Weinehall; L. A. Persson; P. O. Wester; S. Wall | | Int J Epidemiol | | 1993 | | 22 | | 6 | | 1026-37 | | 10.1093/ije/22.6.1026 | |
| 502 | | Neighborhood SES is particularly important to the cardiovascular health of low SES individuals | | J. M. Boylan; S. A. Robert | | Soc Sci Med | | 2017 | | 188 | |  | | 60-68 | | 10.1016/j.socscimed.2017.07.005 | |
| 503 | | Low job control and risk of coronary heart disease in Whitehall II (prospective cohort) study | | H. Bosma; M. G. Marmot; H. Hemingway; A. C. Nicholson; E. Brunner; S. A. Stansfeld | | Bmj | | 1997 | | 314 | |  | | 558-65 | | 10.1136/bmj.314.7080.558 | |
| 504 | | High Inequalities Associated With Socioeconomic Deprivation in Cardiovascular Disease Burden and Antihypertensive Medication in Hungary | | K. Boruzs; A. Juhász; C. Nagy; Z. Szabó; M. Jakovljevic; K. Bíró; R. Ádány | | Front Pharmacol | | 2018 | | 9 | |  | |  | | 10.3389/fphar.2018.00839 | |
| 505 | | Higher Prevalence of Diabetes Mellitus and Impaired Glucose Tolerance Among the Rural Population in Bulgaria | | A. M. Borissova; A. Shinkov; J. Vlahov; L. Dakovska; T. Todorov | | J. Endocrinol. Metab. | | 2016 | | 6 | | 2 | | 52-58 | | 10.14740/jem334w | |
| 506 | | Prevalence of treated diabetes: Geographical variations at the small-area level and their association with area-level characteristics. A multilevel analysis in Southeastern France | | A. Bocquier; S. Cortaredona; S. Nauleau; M. Jardin; P. Verger | | Diabetes Metab | | 2011 | | 37 | | 1 | | 39-46 | | 10.1016/j.diabet.2010.07.004 | |
| 507 | | Preventable Hospitalization Rates and Neighborhood Poverty among New York City Residents, 2008-2013 | | A. Bocour; M. Tria | | J Urban Health | | 2016 | | 93 | | 6 | | 974-983 | | 10.1007/s11524-016-0090-5 | |
| 508 | | Social network and blood pressure: a population study | | S. H. Bland; V. Krogh; W. Winkelstein; M. Trevisan | | Psychosom Med | | 1991 | | 53 | | 6 | | 598-607 | | 10.1097/00006842-199111000-00002 | |
| 509 | | Long term relations between earthquake experiences and coronary heart disease risk factors | | S. H. Bland; E. Farinaro; V. Krogh; F. Jossa; A. Scottoni; M. Trevisan | | Am J Epidemiol | | 2000 | | 151 | | 11 | | 1086-90 | | 10.1093/oxfordjournals.aje.a010152 | |
| 510 | | How well does social variation mirror secular change in prevalence of cardiovascular risk factors in a country in transition? | | P. Bjerregaard; I. K. Dahl-Petersen | | Am J Hum Biol | | 2011 | | 23 | | 6 | | 774-9 | | 10.1002/ajhb.21209 | |
| 511 | | Social ties and cardiovascular function: an examination of relationship positivity and negativity during stress | | W. Birmingham; B. N. Uchino; T. W. Smith; K. C. Light; D. M. Sanbonmatsu | | Int J Psychophysiol | | 2009 | | 74 | | 2 | | 114-9 | | 10.1016/j.ijpsycho.2009.08.002 | |
| 512 | | Association of neighbourhood socioeconomic status and diabetes burden using electronic health records in Madrid (Spain): the HeartHealthyHoods study | | U. Bilal; F. Hill-Briggs; L. Sánchez-Perruca; I. Del Cura-González; M. Franco | | BMJ Open | | 2018 | | 8 | | 9 | |  | | 10.1136/bmjopen-2017-021143 | |
| 513 | | Ethnic and socio-economic inequalities in coronary heart disease, diabetes and risk factors in Europeans and South Asians | | R. Bhopal; L. Hayes; M. White; N. Unwin; J. Harland; S. Ayis; G. Alberti | | J Public Health Med | | 2002 | | 24 | | 2 | | 95-105 | | 10.1093/pubmed/24.2.95 | |
| 514 | | Prevalence and correlates of hypertension in Maharashtra, India: A multilevel analysis | | M. D. Bhise; S. Patra | | PLoS One | | 2018 | | 13 | | 2 | |  | | 10.1371/journal.pone.0191948 | |
| 515 | | Value of Neighborhood Socioeconomic Status in Predicting Risk of Outcomes in Studies That Use Electronic Health Record Data | | N. A. Bhavsar; A. Gao; M. Phelan; N. J. Pagidipati; B. A. Goldstein | | JAMA Netw Open | | 2018 | | 1 | | 5 | |  | | 10.1001/jamanetworkopen.2018.2716 | |
| 516 | | Multimorbidity and its associated factors among adults aged 50 and over: A cross-sectional study in 17 European countries | | D. L. Bezerra de Souza; A. Oliveras-Fabregas; A. Espelt; M. Bosque-Prous; M. de Camargo Cancela; E. Teixidó-Compañó; J. Jerez-Roig | | PLoS One | | 2021 | | 16 | | 2 | |  | | 10.1371/journal.pone.0246623 | |
| 517 | | Social network analysis and coronary heart disease | | L. F. Berkman | | Adv Cardiol | | 1982 | | 29 | |  | | 37-49 | | 10.1159/000406195 | |
| 518 | | Development of the cardiovascular morbidity and mortality in East Germany after the political change | | W. Barth; B. Classen; L. Heinemann; L. Gravens; G. Voigt; D. Quietzsch; S. Brasche; S. Bothig | | Z. Gesundheitswiss. | | 1998 | | 6 | | 2 | | 120-136 | | 10.1007/bf02956782 | |
| 519 | | Sex Differences in Life-Course Neighborhood Racial Composition and Adult Hypertension | | D. Barrington | | Circulation | | 2019 | | 140 | |  | |  | |  | |
| 520 | | Social network diversity and risks of ischemic heart disease and total mortality: findings from the Copenhagen City Heart Study | | J. C. Barefoot; M. Grønbaek; G. Jensen; P. Schnohr; E. Prescott | | Am J Epidemiol | | 2005 | | 161 | | 10 | | 960-7 | | 10.1093/aje/kwi128 | |
| 521 | | Neighborhood Disadvantage, Poor Social Conditions, and Cardiovascular Disease Incidence Among African American Adults in the Jackson Heart Study | | S. Barber; D. A. Hickson; X. Wang; M. Sims; C. Nelson; A. V. Diez-Roux | | Am J Public Health | | 2016 | | 106 | | 12 | | 2219-2226 | | 10.2105/ajph.2016.303471 | |
| 522 | | Double-jeopardy: The joint impact of neighborhood disadvantage and low social cohesion on cumulative risk of disease among African American men and women in the Jackson Heart Study | | S. Barber; D. A. Hickson; I. Kawachi; S. V. Subramanian; F. Earls | | Soc Sci Med | | 2016 | | 153 | |  | | 107-15 | | 10.1016/j.socscimed.2016.02.001 | |
| 523 | | At the intersection of place, race, and health in Brazil: Residential segregation and cardio-metabolic risk factors in the Brazilian Longitudinal Study of Adult Health (ELSA-Brasil) | | S. Barber; A. V. Diez Roux; L. Cardoso; S. Santos; V. Toste; S. James; S. Barreto; M. Schmidt; L. Giatti; D. Chor | | Soc Sci Med | | 2018 | | 199 | |  | | 67-76 | | 10.1016/j.socscimed.2017.05.047 | |
| 524 | | Association of Modifiable Risk Factors in Young Adulthood With Racial Disparity in Incident Type 2 Diabetes During Middle Adulthood | | M. P. Bancks; K. Kershaw; A. P. Carson; P. Gordon-Larsen; P. J. Schreiner; M. R. Carnethon | | Jama | | 2017 | | 318 | | 24 | | 2457-2465 | | 10.1001/jama.2017.19546 | |
| 525 | | Examining the effects of individual and neighbourhood socioeconomic status/wealth on hypertension among women in the Greater Accra Region of Ghana | | E. Banchani; E. Y. Tenkorang; W. Midodzi | | Health Soc Care Community | | 2020 | |  | |  | |  | | 10.1111/hsc.13185 | |
| 526 | | Social relations in late adolescence and incident coronary heart disease: a 38-year follow-up of the Swedish 1969-1970 Conscription Cohort | | P. Balog; I. Janszky; H. Chen; B. Rafael; T. Hemmingsson; K. D. László | | BMJ Open | | 2019 | | 9 | | 12 | |  | | 10.1136/bmjopen-2019-030880 | |
| 527 | | Gender-specific associations between perceived and objective neighbourhood crime and metabolic syndrome | | K. L. Baldock; C. Paquet; N. J. Howard; N. T. Coffee; A. W. Taylor; M. Daniel | | PLoS One | | 2018 | | 13 | | 7 | |  | | 10.1371/journal.pone.0201336 | |
| 528 | | Associations between resident perceptions of the local residential environment and metabolic syndrome | | K. Baldock; C. Paquet; N. Howard; N. Coffee; G. Hugo; A. Taylor; R. Adams; M. Daniel | | J Environ Public Health | | 2012 | | 2012 | |  | |  | | 10.1155/2012/589409 | |
| 529 | | Daily social interactions, close relationships, and systemic inflammation in two samples: Healthy middle-aged and older adults | | A. Bajaj; N. A. John-Henderson; J. M. Cundiff; A. L. Marsland; S. B. Manuck; T. W. Kamarck | | Brain Behav Immun | | 2016 | | 58 | |  | | 152-164 | | 10.1016/j.bbi.2016.06.004 | |
| 530 | | Undiagnosed diabetes from cross-sectional GP practice data: an approach to identify communities with high likelihood of undiagnosed diabetes | | N. Bagheri; I. McRae; P. Konings; D. Butler; K. Douglas; P. Del Fante; R. Adams | | BMJ Open | | 2014 | | 4 | | 7 | |  | | 10.1136/bmjopen-2014-005305 | |
| 531 | | Identifying hotspots of type 2 diabetes risk using general practice data and geospatial analysis: an approach to inform policy and practice | | N. Bagheri; P. Konings; K. Wangdi; A. Parkinson; S. Mazumdar; E. Sturgiss; A. Lal; K. Douglas; N. Glasgow | | Aust J Prim Health | | 2019 | |  | |  | |  | | 10.1071/py19043 | |
| 532 | | Community cardiovascular disease risk from cross-sectional general practice clinical data: a spatial analysis | | N. Bagheri; B. Gilmour; I. McRae; P. Konings; P. Dawda; P. Del Fante; C. van Weel | | Prev Chronic Dis | | 2015 | | 12 | |  | |  | | 10.5888/pcd12.140379 | |
| 533 | | Sex-specific effects of social networks on the prevalence, awareness, and control of hypertension among older Korean adults | | J. Baek; N. W. Hur; H. C. Kim; Y. Youm | | J Geriatr Cardiol | | 2016 | | 13 | | 7 | | 580-6 | | 10.11909/j.issn.1671-5411.2016.07.005 | |
| 534 | | Neighborhood psychosocial hazards and cardiovascular disease: the Baltimore Memory Study | | T. Augustin; T. A. Glass; B. D. James; B. S. Schwartz | | Am J Public Health | | 2008 | | 98 | | 9 | | 1664-70 | | 10.2105/ajph.2007.125138 | |
| 535 | | Association of insulin resistance with distance to wealthy areas: the multi-ethnic study of atherosclerosis | | A. H. Auchincloss; A. V. Diez Roux; D. G. Brown; E. S. O'Meara; T. E. Raghunathan | | Am J Epidemiol | | 2007 | | 165 | | 4 | | 389-97 | | 10.1093/aje/kwk028 | |
| 536 | | Health inequalities and the impact on the prevalence of cardiovascular risk factors and chronic complications in Argentina: a study on national risk factors surveys | | S. Asteazaran; J. J. Gagliardino; J. F. Elgart | | Medwave | | 2017 | | 17 | | 9 | |  | | 10.5867/medwave.2017.09.7083 | |
| 537 | | Effect of area-based deprivation on the severity, subtype, and outcome of ischemic stroke | | S. Aslanyan; C. J. Weir; K. R. Lees; J. L. Reid; G. T. McInnes | | Stroke | | 2003 | | 34 | | 11 | | 2623-8 | | 10.1161/01.Str.0000097610.12803.D7 | |
| 538 | | Geographic and demographic variation in the prevalence of the metabolic syndrome in Canada | | C. I. Ardern; P. T. Katzmarzyk | | Can. J. Diabetes | | 2007 | | 31 | | 1 | | 34-46 | | 10.1016/S1499-2671(07)11009-1 | |
| 539 | | Prevalence of diabetes and prediabetes in 15 states of India: results from the ICMR-INDIAB population-based cross-sectional study | | R. M. Anjana; M. Deepa; R. Pradeepa; J. Mahanta; K. Narain; H. K. Das; P. Adhikari; P. V. Rao; B. Saboo; A. Kumar; A. Bhansali; M. John; R. Luaia; T. Reang; S. Ningombam; L. Jampa; R. O. Budnah; N. Elangovan; R. Subashini; U. Venkatesan; R. Unnikrishnan; A. K. Das; S. V. Madhu; M. K. Ali; A. Pandey; R. S. Dhaliwal; T. Kaur; S. Swaminathan; V. Mohan | | Lancet Diabetes Endocrinol | | 2017 | | 5 | | 8 | | 585-596 | | 10.1016/s2213-8587(17)30174-2 | |
| 540 | | Income Inequality and Chronic Health Conditions: A Multilevel Analysis of the U.S. States | | K. F. Anderson; E. Bjorklund; S. Rambotti | | Sociol. Focus | | 2019 | | 52 | | 1 | | 65-85 | | 10.1080/00380237.2018.1484251 | |
| 541 | | Life-course socio-economic position, area deprivation and Type 2 diabetes: findings from the British Women's Heart and Health Study | | A. F. Andersen; C. Carson; H. C. Watt; D. A. Lawlor; K. Avlund; S. Ebrahim | | Diabet Med | | 2008 | | 25 | | 12 | | 1462-8 | | 10.1111/j.1464-5491.2008.02594.x | |
| 542 | | Neighborhood socioeconomic status and aortic stenosis: A Swedish study based on nationwide registries and an echocardiographic screening cohort | | P. Andell; X. Li; A. Martinsson; P. M. Nilsson; B. Zöller; J. G. Smith; K. Sundquist | | Int J Cardiol | | 2020 | | 318 | |  | | 153-159 | | 10.1016/j.ijcard.2020.06.034 | |
| 543 | | Poor structural social support is associated with an increased risk of Type 2 diabetes mellitus: findings from the MONICA/KORA Augsburg cohort study | | J. Altevers; K. Lukaschek; J. Baumert; J. Kruse; C. Meisinger; R. T. Emeny; K. H. Ladwig | | Diabet Med | | 2016 | | 33 | | 1 | | 47-54 | | 10.1111/dme.12951 | |
| 544 | | High risk neighbourhoods: The effect of neighbourhood level factors on cardiac arrest incidence | | K. S. Allan; J. G. Ray; P. Gozdyra; L. J. Morrison; A. Kiss; J. E. Buick; C. C. Zhan; P. Dorian | | Resuscitation | | 2020 | | 149 | |  | | 100-108 | | 10.1016/j.resuscitation.2020.02.002 | |
| 545 | | Association between socio-economic status and hemoglobin A1c levels in a Canadian primary care adult population without diabetes | | B. Aliarzadeh; M. Greiver; R. Moineddin; C. Meaney; D. White; A. Moazzam; K. M. Moore; P. Belanger | | BMC Fam Pract | | 2014 | | 15 | |  | |  | | 10.1186/1471-2296-15-7 | |
| 546 | | Evidence for affluence-related hypertension in urban Brazil | | L. Ala; G. Gill; R. Gurgel; L. Cuevas | | J Hum Hypertens | | 2004 | | 18 | | 11 | | 775-9 | | 10.1038/sj.jhh.1001750 | |
| 547 | | Relationship of strength of social support and frequency of social contact with hypertension and general health status among older adults in the mobile care unit in Kuwait | | Y. Y. Al-Kandari | | J Cross Cult Gerontol | | 2011 | | 26 | | 2 | | 175-87 | | 10.1007/s10823-011-9139-9 | |
| 548 | | Neighborhood perceptions and hypertension among low-income black women: a qualitative study | | M. Al-Bayan; N. Islam; S. Edwards; D. T. Duncan | | BMC Public Health | | 2016 | | 16 | | 1 | |  | | 10.1186/s12889-016-3741-2 | |
| 549 | | Neighborhood Deprivation Predicts Heart Failure Risk in a Low-Income Population of Blacks and Whites in the Southeastern United States | | E. A. Akwo; E. K. Kabagambe; F. E. Harrell, Jr.; W. J. Blot; J. M. Bachmann; T. J. Wang; D. K. Gupta; L. Lipworth | | Circ Cardiovasc Qual Outcomes | | 2018 | | 11 | | 1 | |  | | 10.1161/circoutcomes.117.004052 | |
| 550 | | Social capital and risk for chronic illnesses | | M. M. Ahern; M. S. Hendryx | | Chronic Illn | | 2005 | | 1 | | 3 | | 183-90 | | 10.1177/17423953050010030201 | |
| 551 | | Ethnic differences in the effect of environmental stressors on blood pressure and hypertension in the Netherlands | | C. Agyemang; C. van Hooijdonk; W. Wendel-Vos; J. K. Ujcic-Voortman; E. Lindeman; K. Stronks; M. Droomers | | BMC Public Health | | 2007 | | 7 | |  | |  | | 10.1186/1471-2458-7-118 | |
| 552 | | Income level and chronic ambulatory care sensitive conditions in adults: a multicity population-based study in Italy | | N. Agabiti; M. Pirani; P. Schifano; G. Cesaroni; M. Davoli; L. Bisanti; N. Caranci; G. Costa; F. Forastiere; C. Marinacci; A. Russo; T. Spadea; C. A. Perucci | | BMC Public Health | | 2009 | | 9 | |  | |  | | 10.1186/1471-2458-9-457 | |
| 553 | | Income inequality and cardiovascular disease risk factors in a highly unequal country: a fixed-effects analysis from South Africa | | K. Adjaye-Gbewonyo; I. Kawachi; S. V. Subramanian; M. Avendano | | Int J Equity Health | | 2018 | | 17 | | 1 | |  | | 10.1186/s12939-018-0741-0 | |
| 554 | | Effects of area deprivation on health risks and outcomes: a multilevel, cross-sectional, Australian population study | | R. J. Adams; N. Howard; G. Tucker; S. Appleton; A. W. Taylor; C. Chittleborough; T. Gill; R. E. Ruffin; D. H. Wilson | | Int J Public Health | | 2009 | | 54 | | 3 | | 183-92 | | 10.1007/s00038-009-7113-x | |
| 555 | | Neighborhood Environments and Coronary Heart Disease: A Multilevel Analysis | | A. V. Diez-Roux, F. J. Nieto, C. Muntaner, H. A. Tyroler, G. W. Comstock, E. Shahar, ... & M. Szklo. | | Am J Epidemiol | | 2017 | | 185 | | 11 | | 1187-1202 | | 10.1093/aje/kwx113 | |
