# Supplementary material for: Associations between dimensions of the social environment and cardiometabolic risk factors: Systematic review and meta-analysis
Source: SSM Popul Health. 2023 Nov 25;25:101559. doi: 10.1016/j.ssmph.2023.101559 (PMC10749911; doi:10.1016/j.ssmph.2023.101559)
Supplement: Multimedia component 7 — Supplementary File 2. Conceptual framework on social environment and cardiometabolic health. [file mmc7.pdf]

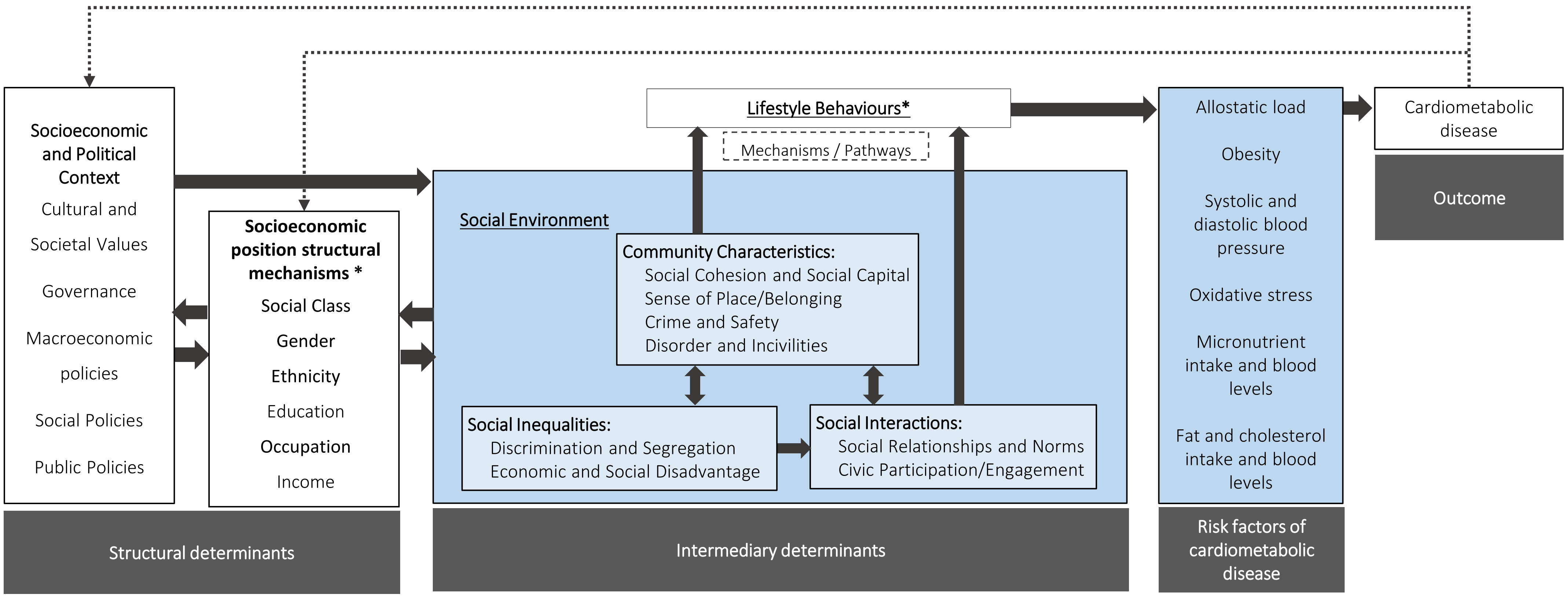

Supplemental File 2. Conceptual framework on social environment and cardiometabolic health

**there is an interplay between the elements within this box*
